# Comparative transcriptomic analysis of races 1, 2, 5 and 6 of *Fusarium oxysporum* f.sp. *pisi* in a susceptible pea host identifies differential pathogenicity profiles

**DOI:** 10.1186/s12864-021-08033-y

**Published:** 2021-10-09

**Authors:** Saidi R. Achari, Jacqueline Edwards, Ross C. Mann, Jatinder K. Kaur, Tim Sawbridge, Brett A. Summerell

**Affiliations:** 1 AgriBio, Agriculture Victoria Research, DJPR, Bundoora, Victoria Australia; 2 School of Applied Systems Biology, La Trobe University, Bundoora, Victoria Australia; 3 Australian Institute of Botanical Science, Royal Botanic Gardens & Domain Trust, Sydney, NSW Australia

**Keywords:** *Fusarium oxysporum* f.sp. *pisi*, Transcriptomics, *Pisum sativum*, Pathogenicity factors, Necrotrophic, Toxins

## Abstract

**Background:**

The fungal pathogen *Fusarium oxysporum* f.sp. *pisi* (Fop) causes *Fusarium* wilt in peas. There are four races globally: 1, 2, 5 and 6 and all of these races are present in Australia. Molecular infection mechanisms have been studied in a few other *F. oxysporum formae speciales;* however, there has been no transcriptomic Fop-pea pathosystem study.

**Results:**

A transcriptomic study was carried out to understand the molecular pathogenicity differences between the races. Transcriptome analysis at 20 days post-inoculation revealed differences in the differentially expressed genes (DEGs) in the Fop races potentially involved in fungal pathogenicity variations. Most of the DEGs in all the races were engaged in transportation, metabolism, oxidation-reduction, translation, biosynthetic processes, signal transduction, proteolysis, among others. Race 5 expressed the most virulence-associated genes. Most genes encoding for plant cell wall degrading enzymes, CAZymes and effector-like proteins were expressed in race 2. Race 6 expressed the least number of genes at this time point.

**Conclusion:**

Fop races deploy various factors and complex strategies to mitigate host defences to facilitate colonisation. This investigation provides an overview of the putative pathogenicity genes in different Fop races during the necrotrophic stage of infection. These genes need to be functionally characterised to confirm their pathogenicity/virulence roles and the race-specific genes can be further explored for molecular characterisation.

**Supplementary Information:**

The online version contains supplementary material available at 10.1186/s12864-021-08033-y.

## Introduction

*Fusarium oxysporum* (Fo), a soil-borne fungus, is a species complex of putatively non-pathogenic and pathogenic strains. Pathogenic strains, designated as *formae speciales* (ff.spp.), cause infection in more than 100 plant species of important agricultural crops such as cotton, tomato, banana, legumes, and others [1]. Some ff.spp. of Fo are further divided into several physiological races based on the host range of cultivars on which they cause disease. Fo *forma specialis* (f.sp.) *pisi* (Fop) causes vascular wilt in peas (*Pisum sativum*). Phylogenetic relationship study has shown that isolates of Fop races are polyphyletic and are present in clades 2 and 3 of the *Fusarium oxysporum* species complex (FOSC) [2].

Fop is present in nearly all pea growing regions globally [3]. The most common symptoms include chlorosis of the leaves beginning from the lower regions of the plant, extending upwards as the disease progresses [4]. The chlorotic leaflets curl downward and become flaccid [4]. There is also a red to brown discoloration of the vascular bundles, a characteristic symptom of *Fusarium* wilt [4]. Infected pea plants often develop dry, stunted and shrivelled pods hence unsuitable for harvest [4]. Fop race 2 causes secondary cortical decay in roots and stems and disease symptoms progress slowly and plant death occurs in the very late stages of the disease process [5].

As a soil-borne disease, control of *Fusarium* wilt is achieved by integrating different disease management techniques such as crop rotation, good field hygiene, on-farm biosecurity, and resistant cultivars. The use of resistant cultivars is the safest, most economical and most effective crop protection method [6]. Single dominant genes confer resistance to Fop races 1, 5 and 6 whereas resistance to race 2 is quantitative [5].

Recent histological studies on Fop race 2 infection by Bani et al. [7] showed direct penetration of the host root by a constricted hypha. Constriction of the penetrating hypha at the site of cell penetration was observed at the epidermis, exodermis and cortex. Similar cell penetration was previously reported for *Fusarium* wilt diseases of tomato and pea [8, 9]. Upon penetration, infective hyphae were seen growing in and out of the cortical cell via constricted hyphae [7, 9]. Similarly, intracellular colonisation of cortical cells was reported in cabbage and lentil infected with Fo [10, 11]. Degradation of cell layers surrounding the endodermis in susceptible cultivars was also visible [7]. Similar findings were reported for several other ff.spp. [9, 12]. The colonisation of xylem vessels and subsequent spread to the hypocotyl is the final step of Fo infection. In the Bani et al. [7] study, Fop entered immature and mature xylem vessels through direct penetration and elongation of the infective hyphae. Inter- and intra-cellular colonisation of fibre and parenchyma cells were also observed. In some instances, germinated microconidia entering through the xylem perforation plate contributed to colonisation and movement of Fop through the xylem [7]. Other Fo histological studies reported similar findings [13].

The molecular mechanisms of Fop pathogenicity and the genetic basis of host specificity are poorly understood. Currently, there are no known pathogenicity genes in Fop. However, some pathogenicity genes have been reported in other Fo ff.spp.. The genomes of Fo contain many pathogenic or virulence-associated genes, including effectors, transcription factors, G-proteins, protein kinases, transmembrane transporters, and CAZymes, among others [14–16]. One of the well documented group of effectors in Fo are Secreted in xylem (*SIX*) genes. A total of fourteen *SIX* genes (SIX1 to 14) have been identified in Fo f.sp. lycopersici (Fol) [15, 17, 18]. Homologs of these genes are also present in different combinations in other Fo [19–21]. These genes are expressed during the early stages of infection [22].

Previous studies have shown that phytopathogens secrete enormous amounts of proteins, toxins, secondary metabolites and hormones. These proteins either act as virulence factors, i.e., intensify disease symptoms, or act as pathogenicity factors, i.e., are exclusively responsible for developing disease symptoms [23]. Several categories of these factors, namely (i) proteins involved in signal transduction, (ii) proteins generating or detoxifying toxins, (iii) metabolic enzymes, (iv) proteins involved in forming infection structures, (v) transmembrane transporters, (vi) proteins involved in stress response and (vii) proteins involved in fungal development have been functionally characterised with pathogenicity roles in several phytopathogens [24–30].

High-throughput RNA sequencing (RNA-seq) technology presents a powerful and efficient method for transcriptome analysis, leading to the discovery of many novel genes. It has made it possible to relate gene expressions to physical disease symptoms. A transcriptomic analysis is one of the most effective ways to understand the molecular mechanisms of fungal pathogenesis. The main objective of this study was to identify putative effectors and pathogenicity genes expressed by Fop races during infection and colonisation. Such findings would facilitate understanding of the host-pathogen interactions involved in *Fusarium* wilt disease of peas and the molecular mechanism underlying the differences in the infection strategies between the different races of Fop.

## Results and discussion

### In vitro and in planta Fusarium RNA-sequencing

It was hypothesised that gene expression between the different races would vary but that there would be some common genes expressed in all the races, given they infect the same host plant species. We also hypothesised that there would be differences in the gene expression at different time points (5 dpi and 20 dpi) within a race.

**Fig. 1:**
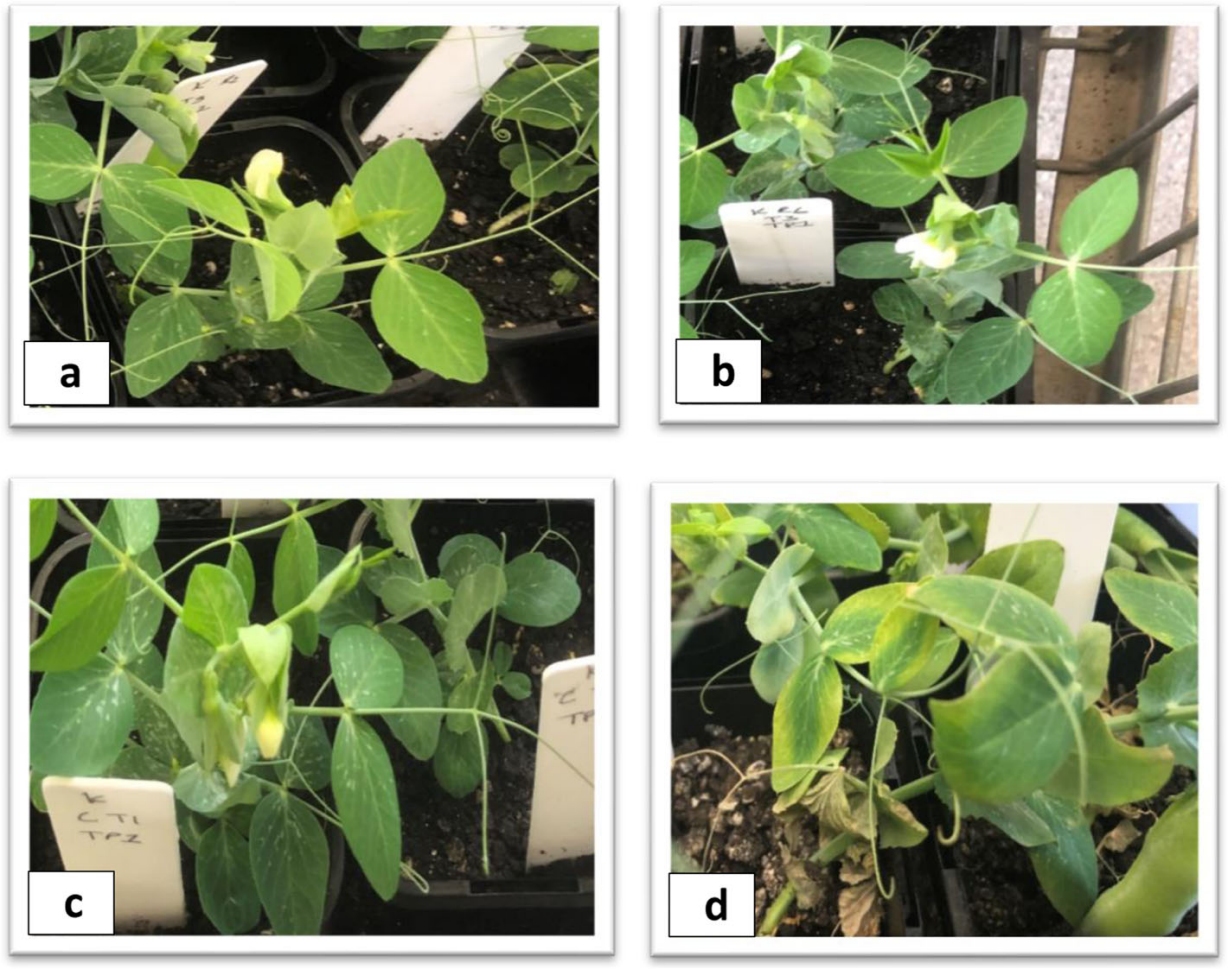
External Fop infection symptoms on the plants. **b** and **d** show external infection symptoms at 5 dpi, and at 20 dpi in a susceptible pea cultivar, respectively. **a** and **c** are control plants at 5 dpi and 20 dpi, respectively

**Fig. 2:**
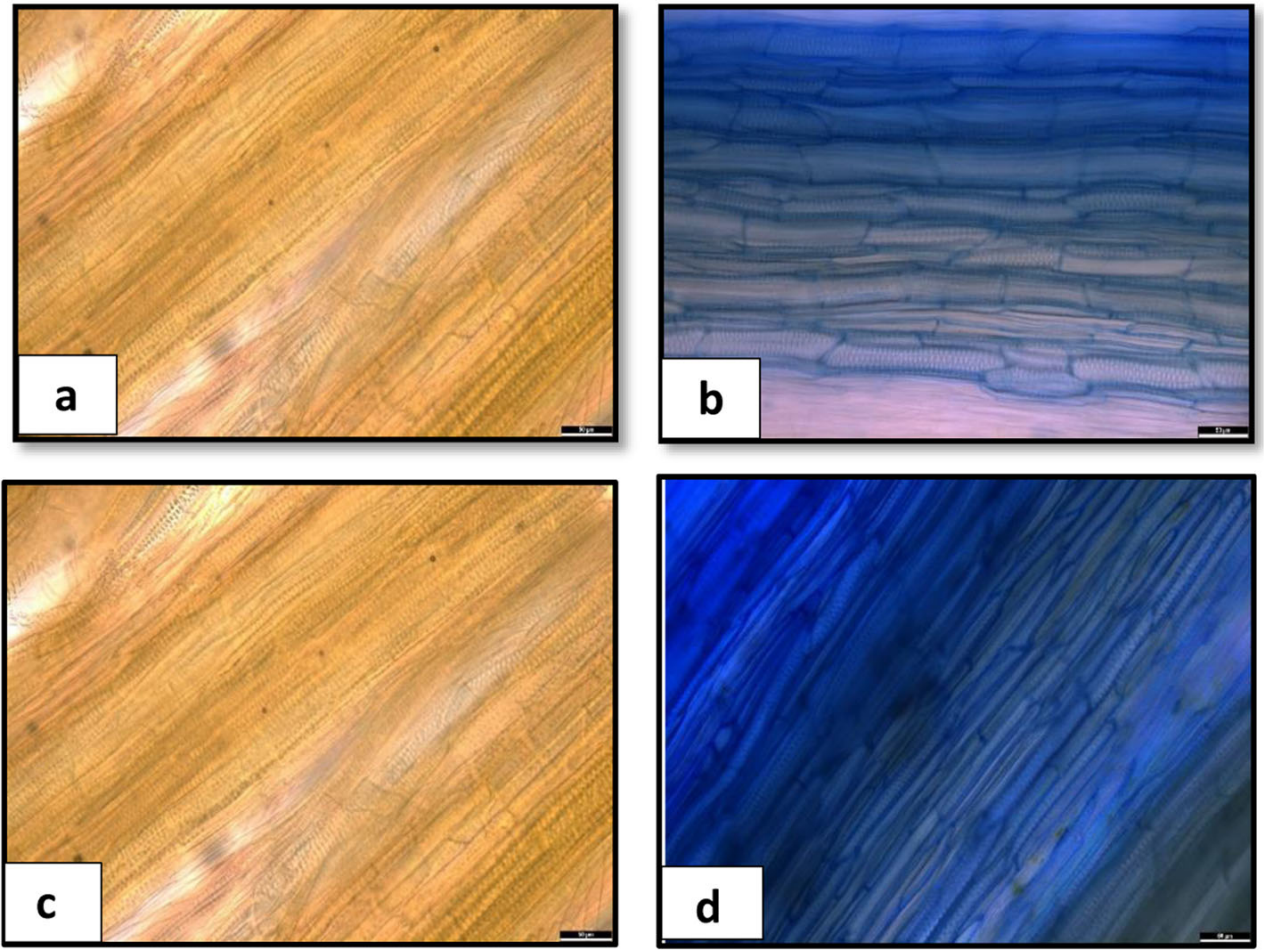
Comparison of Fop colonization within plant tissues at 5 dpi and at 20 dpi in a susceptible pea cultivar. Each picture shows a section of the plant’s basal stem superficially stained with the commercial ink Parker Blue, indicating fungal presence as a blue coloration. **a** and **c** are control plants at 5 dpi and 20 dpi, respectively, while **b** and **d** are Fop infected pea plants at 5 dpi and 20 dpi, respectively. The scale bar is 50 μm with 34X magnification

**Fig. 3:**
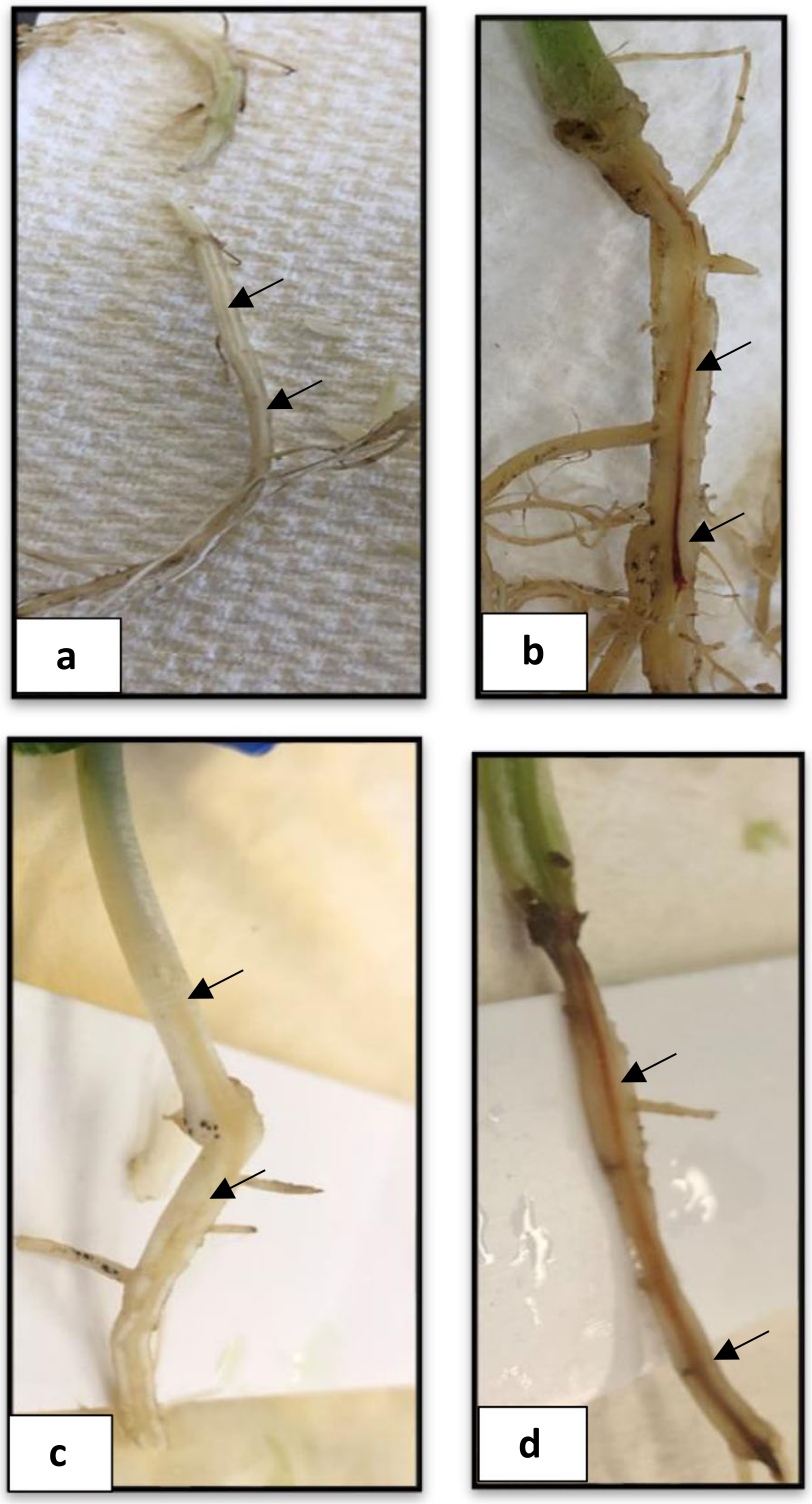
Fop-induced vascular discoloration in a susceptible pea cultivar. Each picture shows the main root up to the basal stem at 5 dpi and at 20 dpi. **a** and **c** are control plants at 5 dpi and 20 dpi, respectively while **b** and **d** are Fop infected pea plants at 5 dpi and 20 dpi, respectively. Black arrows indicate reddish vascular tissue

To capture the Fop genes expressed in the root and shoot samples and to compare and contrast their expression profiles, we generated high coverage RNA-Seq data from the infected root and shoot tissues of all the four races (race1 (R1), race 2 (R2), race 5 (R5) and race 6 (R6)) at 5 dpi when there were no visible exterior symptoms, and at 20 dpi when the disease symptoms were clearly visible (Fig. 1a-d). Infection inside the tissue was confirmed by staining the basal stem of the plants (Fig. 2a-d) and dissection of roots (Fig. 3a-d). Transcriptomic data from Fop mycelia grown in vitro was used to compare the levels of gene expression induced with host detection to facilitate identification of differentially expressed genes (DEGs) which may potentially be associated with pathogenicity Four biological replicates were used for all the *in planta* and in vitro samples for all the races.

The number of reads from the infected root and shoot samples that mapped to the respective Fop genome was far less at 5 dpi than 20 dpi (Table 1). The presence of Fop at 5 dpi was confirmed through the presence of the discoloured xylem vessel (Fig. 3a-d) and tissue staining (Fig. 2a-d). Because of the low number of fungal reads, 5 dpi data was unable to be further processed. At 20 dpi, the number of reads from the infected root samples that mapped to the respective Fop genomes was much higher than those from the infected shoot samples (Table 1). A principal component analysis was conducted to confirm the relatedness of the four biological replicates of all the *in planta* and in vitro samples for all the races and the accuracy of the RNA-Seq analysis. Individual replicates of each race (in vitro/*in planta*) clustered together, indicating a high degree of similarity in the expression profiles and low biological variability among the experimental replicates.

**Table 1:**
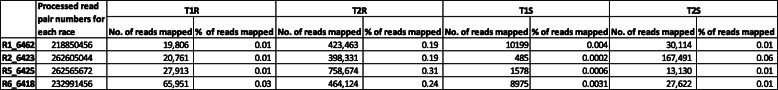
Mapping results of RNA-Seq data from four races of Fop infected root and shoot samples at 5 dpi and at 20 dpi

Out of all the shoot samples, R2 infected shoot samples had the highest number of reads mapping to the Fop R2 genome, while R5 infected root samples had the highest number of the reads mapping the Fop R5 genome out of all the infected root samples (Table 1).

### Comparison of DEGs in infected shoot and root tissues

There was a significant difference (Fisher’s LSD *p* ≤ 0.05) in the number of DEGs from the root and shoot samples between all the races with root tissues expressing more (Supplementary Fig. 1). However, some DEGs were uniquely expressed in the shoot tissues only. R1, R5 and R6 had five, three and 11 unique DEGs in the shoot tissues, respectively.

### The DEGs in different Fop races

**Fig. 4:**
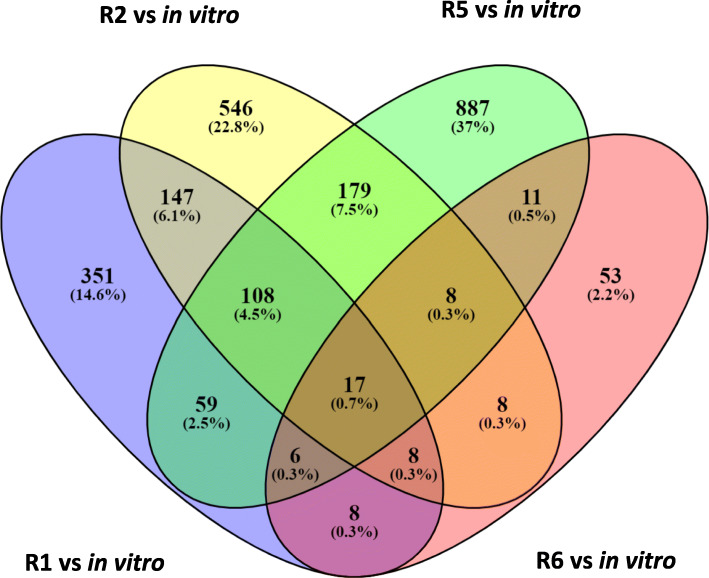
Venn diagram of unique and overlapping Fop genes differentially expressed between in vitro and *in planta* and upregulated in each race at 20 dpi

The DEGs found in Fop transcriptomes from the infected root and shoot tissues were combined to obtain the DEGs per race. There was a significant difference (Fisher’s LSD *p* ≤ 0.05) in the number of the DEGs between the races. The highest number of DEGs were from R5 (1275), followed by R2 (1021), R1 (704) and R6 (119) (Fig. 4). A large percentage of these DEGs encoded hypothetical proteins (HPs) and uncharacterised proteins ranging from 57% for R1 to 70% for R5 (Supplementary Tables 1, 2, 3 and 4).

### Putative effector proteins and their characteristics

Effectors are small secreted proteins that can alter host cell metabolism, inhibit or stimulate effector-triggered immune responses and facilitate infection [31]. DEGs were classified as effectors if they were predicted by Effector2P (http://effectorp.csiro.au/) [32] and had a secretory signal. The secretory proteins included proteins secreted through conventional and unconventional pathways but lacking transmembrane domains and the glycosylphosphatidylinositol (GPI)-anchored proteins. Proteins secreted through the conventional secretory pathway (endoplasmic reticulum and Golgi route) have a signal peptide at the N-terminus, while proteins secreted via the unconventional pathways do not have signal peptides [33].

**Fig. 5:**
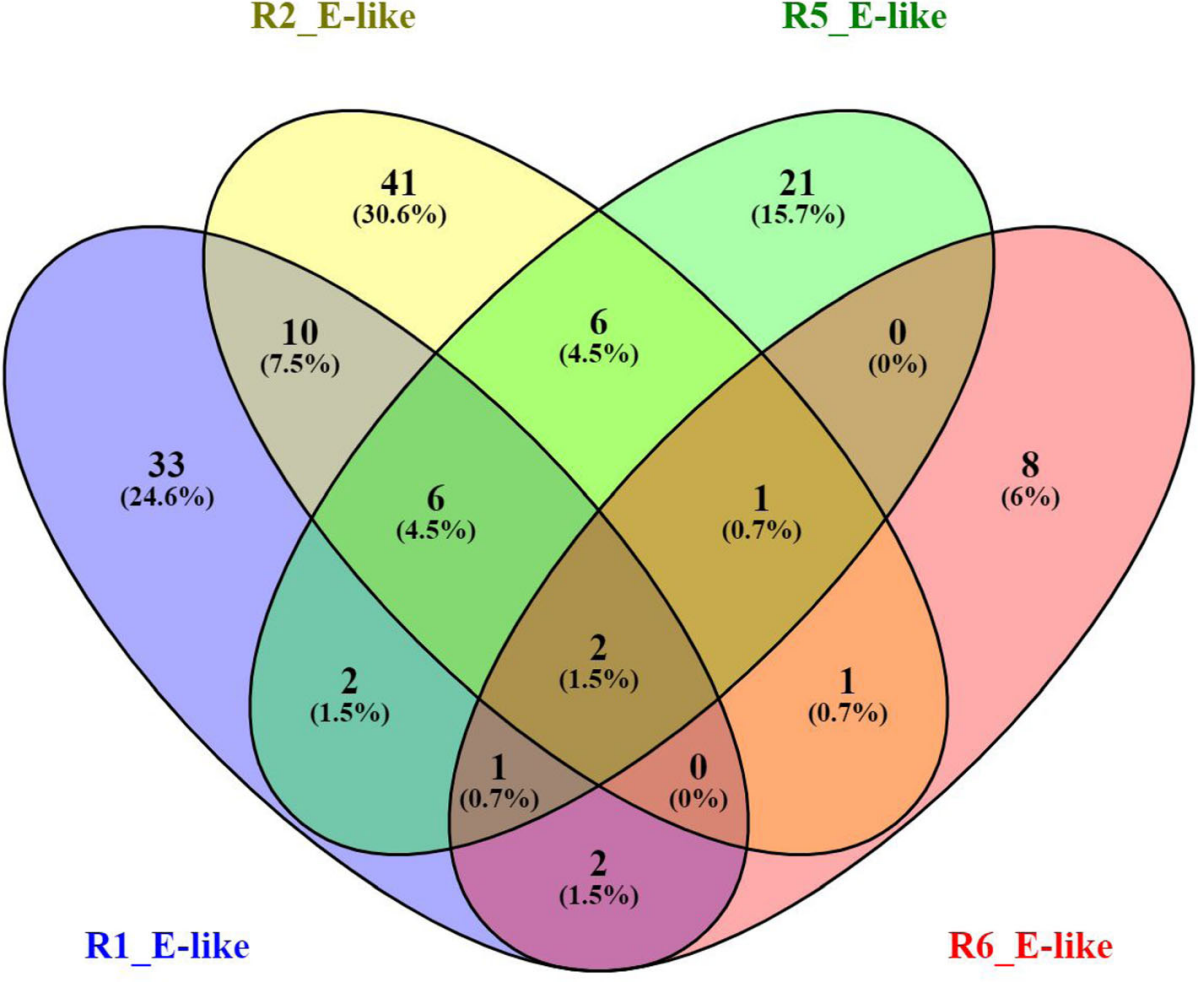
Venn diagram of unique and overlapping effector-like proteins differentially expressed between in vitro and *in planta* and upregulated in each race at 20 dpi

Most of the putative effector proteins were less than 300 amino acids in length (Supplementary Fig. 2) and did not possess a signal peptide (Supplementary Tables 1, 2, 3 and 4). The ratio of total putative effector proteins to putative effector proteins lacking a signal peptide for races 1, 2, 5 and 6 were 56:30, 67:50, 54:46 and 15:2, respectively (Supplementary Fig. 2). This finding suggests that putative effector proteins during the necrotrophic infection stage in Fop are mostly secreted through unconventional pathways. Previous studies have documented effectors in Fo and other filamentous fungi without a recognisable signal peptide [34–37]. Each race had some unique effector-like proteins and there were only two effector-like proteins common in all the races. There was a significant difference (Fisher’s LSD *p* ≤ 0.05) in the number of DEGs encoding effector-like proteins in all the races. Races 1, 2, 5 and 6 had 33, 41, 21 and eight race-specific effector-like proteins, respectively (Fig. 5).

Putative effector proteins containing a signal peptide were predicted to be only located in the extracellular space. In contrast, putative effector proteins without a signal peptide were predicted to be located in the extracellular space, cytoplasm, or associated with plant organelles. Extracellular localised putative effector proteins were dominant in all the races (Supplementary Fig. 2). All the races had putative effectors encoding putative effector proteins with sequence homology to virulence-associated genes on the Pathogen-Host Interactions (PHI-base) database (http://www.phi-base.org) [38] (Supplementary Tables 5, 6, 7 and 8).

### Gene ontology (GO) enrichment analysis of the DEGs in different Fop races

Gene ontology (GO) enrichment analysis for biological processes was used to predict the functions of the DEGs by classifying them according to the biological processes (BP) in which they are involved.

**Fig. 6:**
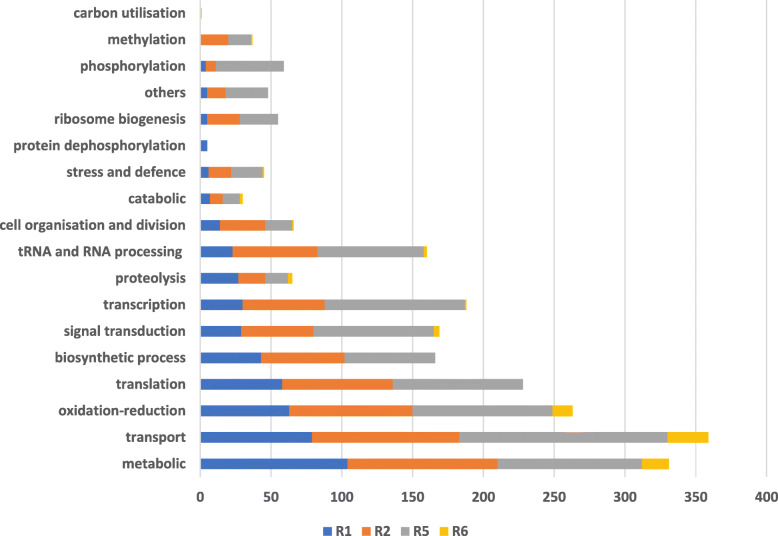
Percentage of DEGs involved in important biological processes in different races based on GO enrichment analyses

Seventy-one percent of the DEGs identified in R1 were associated with a BP. The top five BPs were metabolism (14.7%), transportation (11.2%), oxidation-reduction (8.9%), translation (8.2%) and cellular biosynthetic process (6.1%) (Fig. 6). Seventy-two percent of the DEGs in R2 were associated with a BP, and the top five were transportation (10.4%), metabolism (10.1%), oxidation-reduction (8.5%), translation (7.6%) and RNA processing (5.9%) (Fig. 6). R5 had 74% DEGs associated with a BP; the top five were transportation (11.5%), metabolism (8%), transcription (7.7%), oxidation-reduction (7.7%) and translation (7.2%) (Fig. 6). Sixty-six percent of the DEGs in R6 were associated with a BP, the top five of which were transportation (24.4%), metabolism (16%), oxidation-reduction (11.7%), signal transduction (3.3%) and proteolysis (2.5%) (Fig. 6).

The BPs in which the DEGs are involved are important processes for Fop pathogenicity. Transportation and metabolism were the most important BPs in all the races. Previous Fo-host interaction studies have also exhibited high expression of genes involved in metabolism and transportation [22, 39]. Fop is growing vigorously within the pea host during the necrotrophic stage and, as such, needs nutrients to facilitate further invasion and survival within the host, avoiding host defences. Genes encoding for transmembrane transporters are highly expressed during the necrotrophic infection stage in most phytopathogenic fungi [40], which mediates the selective uptake of nutrients and functions as an efflux pump for the removal of plant defence metabolites and toxins.

### The virulence-associated genes (VAGs) in Fop

A BLASTp analysis of the amino acid sequences of the transcripts encoded by the DEGs was conducted against the PHI-base database (http://www.phi-base.org) [38] to identify genes that are involved in pathogen-host interactions. The aim was to identify potential pathogenicity genes based on the amino acid sequence similarity in other phytopathogens. There was a significant difference (Fisher’s LSD *p* ≤ 0.05) in the putative virulence-associated genes (VAGs) expressed between all the races. Races 1, 2, 5 and 6 expressed 147, 198, 296 and 29 VAGs, respectively.

R1 transcriptomics search of the PHI-base database yielded VAGs encoding proteins identified as effectors (4 genes), lethal (4 genes), essential for pathogenicity (22 genes) and important for virulence (117 genes) (Supplementary Table 5). These VAGs encoded proteins were involved in plant cell wall degradation, cytochrome P450s, transmembrane transporters, peroxidases, protein kinases and transcription factors, among others (Supplementary Table 5). Included in the genes from the PHI-base database were six already characterised VAGs from Fo, including tom1 (PHI: 2364) [41], AreA (PHI:2283) [42], FGB1 (PHI:2826) [43], FOW1 (PHI:254) [44], FOW2 (PHI:734) [45] and GLX (PHI:5393) [46]. Most of the genes from Fop R1 that mapped onto the PHI-base database were from *Fusarium graminearum* (47 genes), followed by *Magnaporthe oryzae* (42 genes).

R2 transcriptomics search of the PHI-base database yielded VAGs designated as effectors (7 genes), lethal (11 genes), essential for pathogenicity (27 genes) and important for virulence (153 genes) (Supplementary Table 6). These VAGs encoded proteins with CFEM domain, cytochrome P450s, plant cell wall degrading enzymes, polyketide synthase, protein kinases, G-proteins and transcription factors, among others (Supplementary Table 6). Included in the genes from the PHI-base database were three already characterised VAGs from Fo, including FOW2 (PHI:734) [45], Dnj1 (PHI:5236) [47] and Hog1 (PHI:6317) [24]. Most of the genes from Fop R2 that mapped onto the PHI-base database were from *M. oryzae* (64 genes), followed by *F. graminearum* (56 genes).

R5 transcriptomics search of the PHI-base database yielded VAGs designated as effectors (1 gene), lethal (15 genes), essential for pathogenicity (44 genes) and important for virulence (236 genes) (Supplementary Table 7). Proteins encoded by these VAGs included transmembrane transporters, plant cell wall degrading enzymes, G-proteins, heat shock proteins, polyketide synthase, peptidases, protein kinases and transcription factors, among others (Supplementary Table 7). Included in the genes from the PHI-base database were four already characterised VAGs from Fo, including FOW2 (PHI:734) [45], AreA (PHI:2283) [42], Dnj1 (PHI:5236) [47] and FGA1 (PHI:251) [48]. Most of the genes from Fop R5 that mapped onto the PHI-base database were from *M. oryzae* (112 genes), followed by *F. graminearum* (81 genes).

R6 transcriptomics search of the PHI-base database yielded VAGs encoding proteins that were important for virulence (28 genes) and essential for pathogenicity (1 gene) (Supplementary Table 8). These VAGs encoded transporter proteins, cytochrome P450s, glycosyl hydrolases, transcription factors, among others (Supplementary Table 8). Included in the genes from the PHI-base database was one already characterised VAG from Fo, FTF2 (PHI:5482) [49]. Most of the genes from Fop R6 that mapped onto the PHI-base database were from *M. oryzae* (9 genes), followed by *F. graminearum* (7 genes).

The VAGs present in Fop transcripts are involved in roles such as cell adhesion [50], defence against plant phytoalexins [51], synthesis of secondary metabolites, signal transduction pathways [52–55], transcriptional factors [56], CAZymes [57] and development [58]. Genes involved in defence against plant phytoalexins included genes aiding detoxification, active exclusion, and alteration of the plant toxins [51]. Signal transduction enables the pathogen to respond appropriately to the host environment and this includes G-proteins, G-protein-coupled receptors [52, 53], F-box protein [54], cAMP-dependent protein kinase, mitogen-activated protein kinase (MAPK) and histidine kinase protein [55]. Transcriptional factors (TFs) are essential players in regulating diverse biological processes by activating or repressing gene expression [56]. TFs such as Zinc finger, basic-leucine zipper (bZIP) and homeobox protein domains were expressed by Fop races. These pathogenic processes have been shown to be important in Fo as genes involved in these processes were highly expressed in other Fo-host interaction studies [22, 39, 59–62].

One of the MAPK proteins expressed in Fop had sequence homology to the virulence-associated protein encoded by HOG1 (PHI:6317) gene in Fo [24] (Supplementary Table 6). There was 100% amino acid sequence identity between the two proteins. HOG1 gene plays an important role in fungal development, stress mitigation and virulence [24]. Additionally, three of the TF proteins in Fop transcripts had sequence homology to the virulence-associated proteins encoded by FOW2 (PHI:734) [45] and FTF2 (PHI:5482) [49] genes in Fo (Supplementary Tables 5, 6 and 8). FOW2 is conserved in pathogenic strains of Fo where it transcriptionally regulates the plant infection capabilities [45] while FTF2 regulates virulence and expression of *SIX* effectors in Fo [49].

Previously characterised Fo VAGs present in the Fop transcriptomics indicate conservation in VAGs and pathogenicity among the different ff.spp. of Fo. VAGs in Fop fell into various BPs (GO functional analysis), thereby highlighting the critical roles of these processes in Fop pathogenicity. R5 had the most VAGs.

### Genes encoding carbohydrate-active enzymes (CAZymes)

Fungi produce various CAZymes for the degradation of plant polysaccharide materials to facilitate infection and gain nutrition. The CAZymes have been grouped into six functional classes: glycoside hydrolases (GHs), glycosyltransferases (GTs), polysaccharide lyases (PLs), carbohydrate esterases (CEs), non-catalytic carbohydrate-binding modules (CBM) and auxiliary activities (AA) based on their structurally related catalytic modules or functional domains [57, 63]. Some of the CAZyme functional classes have been further subdivided into families and subfamilies. Families within a functional class are based on their similarity with the characterised models considered as functional anchors [63]. Subfamilies are defined according to their homology relationships between members of the family and are designated using the family name plus a suffix indicating the subfamily [63].

Identifying and comparing CAZymes from fungi with different nutritional modes or infection mechanisms may provide information for a better understanding of their lifestyles and infection models [57]. A comparative analysis of genes encoding CAZymes expressed in all the races showed diversity in the number, functional classes and families. There was a significant difference (Fisher’s LSD *p* ≤ 0.05) in the CAZymes expressed between the races except between races 1 and 2. Races 1, 2, 5 and 6 expressed 46, 50, 29 and 11 DEGs encoding CAZymes, respectively. The upregulation of many of these enzymes can be explained by the vital role that they play in infection. Many of these CAZymes are involved in the degradation of the plant cell wall, the majority are from the GH functional class. All the races shared only four CAZymes; CBM63, CE8, GH28, and AA3_2 (sub-family 2 of AA3).

Forty-six DEGs encoding CAZymes expressed in R1 were from all the six functional classes (Supplementary Table 9). Twenty-seven had secretion signals. Thirteen of the CAZymes were from AA families (AA1, AA3, AA5, AA9, AA11 and AA16), two from CBM families (CBM63), six from CE families (CE4, CE5, CE8, CE9 and CE12), 16 from GH families (GH1, GH10, GH18, GH28, GH31, GH32, GH43, GH54, GH74, GH105, GH131 and GH132), one from the GT family (GT3) and eight from PL families (PL1, PL3, PL4 and PL9). Forty-two CAZymes were involved in plant cell wall degradation.

Fifty DEGs encoding CAZymes expressed in R2 were also from all the six functional classes (Supplementary Table 10). Twenty-five had secretion signals. Fifteen of the CAZymes were from AA families (AA1, AA3, AA5, AA8, AA9, AA11 and AA16), one from the CBM family (CBM63), six from CE families (CE1, CE2, CE4, CE8, CE9 and CE12), 21 from GH families (GH1, GH3, GH6, GH7, GH10, GH11, GH13, GH28, GH31, GH35, GH43, GH51, GH74, GH81, GH88, GH105 and GH125), two from GT families (GT2 and GT3) and five from PL families (PL1, PL3 and PL9). Forty-seven CAZymes were involved in degrading plant cell walls.

The number of DEGs encoding CAZymes expressed in R5 were twenty-nine and they were also from all the six functional classes as well (Supplementary Table 11). Eight had a secretion signal. Six of the CAZymes were from AA families (AA1, AA3, AA9 and AA11), two from CBM families (CBM63), four from CE families (CE5, CE8 and CE9), ten from GH families (GH13, GH18, GH23, GH28, GH32, GH35, GH43 and GH54), five from GT families (GT1, GT3, GT15 and GT90) and two from PL families (PL3). Twenty CAZymes were involved in plant cell wall degradation.

Eleven DEGs encoding CAZymes expressed in R6 were from four functional classes (Supplementary Table 12). Eight had a secretion signal. Four of the CAZymes were from AA families (AA3, AA5, and AA9), one from the CBM family (CBM63), two from CE families (CE8) and four from GH families (GH5, GH18, GH28 and GH53). Ten CAZymes were involved in plant cell wall degradation.

All the races employed various CAZymes during the necrotrophic infection stage, with some that were unique to each race at this stage of infection. R1 only expressed genes encoding GH131, GH132 and PL4. Eleven genes encoding CAZymes expressed in R2 only were: CE1, CE2, GH3, GH6, GH7, GH11, GH51, GH81, GH88, GH125 and GT2. Genes encoding GH23, GT1, GT15 and GT90 were only expressed by R5, while genes encoding GH5 and GH53 were only expressed in R6. Since only a single time point and a single isolate per race were used in this study, we cannot state if the genes encoding for these CAZymes are Fop race-specific. The genes encoding CAZymes may be expressed at different times in different races as some genes encoding CAZymes were found in the genomes of other races but were not found to be expressed at 20 dpi.

### Protein families involved in degrading plant cell walls (based on CAZyme prediction using dbCAN)

The first barrier to pathogen invasion is the plant cuticle, composed of C:16 and C:18 fatty acids and their derivatives, forming the cutin and the waxy surfaces [64]. Cutinase is an important enzyme for pathogens attacking the plant’s aerial parts as it facilitates penetration of the first barrier, the cuticular layer [65]. The role of cutinase in *Fusarium* pathogenicity to pea remains controversial. Stahl et al. [66] found that the cutinase gene in *Fusarium solani* was not necessary for pathogenicity on a pea. A later study of the cutinase gene in *F. solani* by Rogers et al. [67] found that it was essential for virulence on pea seedlings 10–12 days post-inoculation. The zinc-finger transcription factor Ctf1 of *F. oxysporum* f.sp. *lycopersici* (Fol) is an orthologue of CTF1α, which controls cutinase gene expression in *F. solani* [65]. Disruption of Ctf1 in Fol prevented the activation and expression of the cutinase (cut1) and lipase (lip1) genes, and there was no difference between the mutant and the wild type Fol isolates in terms of virulence [65], supporting Stahl et al. [66] findings that cutinase may not be an essential pathogenicity factor in the root pathogens. Genes encoding for cutinase were only expressed in R1 and R5 (Supplementary Tables 13 and 14) with no significant difference (Fisher’s LSD *p* ≤ 0.05).

To break down C:16 and C:18 fatty acids and their derivatives present in the plant cuticle, pathogens need to secrete lipases. In *F. graminearum*, the FGL1 gene encoding a secreted lipase is vital for virulence on cereals [68], while its homologue in Fo, Lip1, a gene encoding a lipase in Fol, was highly expressed during infection but was dispensable for virulence [65]. Therefore, pathogenicity roles are different, even though there is an 80% amino acid sequence homology between these lipase proteins [68]. There was no expression of a gene encoding for Lip1 in any of the Fop races during the necrotrophic infection stage; however, a gene encoding for lipase 4 (lip4) was expressed in R5 (Supplementary Table 3). A homologue of lip4 encoded by gene FOC1_g10002228 is present in *F. oxysporum* f. sp. *cubense* (https://www.uniprot.org/uniprot).

*F. graminearum* also secretes phospholipase D (FgPLD), an important phospholipid hydrolase that plays a critical role in various BPs in eukaryotic cells [69]. There are three FgPLD proteins, FgPLD1, FgPLD2 and FgPLD3, which have the same subcellular localisation but with different roles [69]. Of these three phospholipases, only FgPLD1 plays a role in pathogenesis in cereals [69]. The genes encoding phospholipase D and phospholipase D1 expressed in Fop R1 and R2 with no significant difference (Fisher’s LSD *p* ≤ 0.05) are homologues of FgPLD1 (Supplementary Tables 1, 2, 5 and 6).

Since genes encoding cutinases were expressed in R1 and R5 and lipases were only expressed in R1 and R2 despite all the races causing infections; these proteins may not be important pathogenicity factors in Fop. Fop is a soil-borne pathogen hence it does not need to break the cutin layer for entering the host as do air-borne pathogens. Or it could have been expressed by all the races during the early infection stage when Fop entered the host. At 20 dpi, Fop is already established inside the host.

Once the phytopathogen enters the host, it secretes a range of other enzymes to physically degrade the plant cell wall components to allow colonisation and obtain nutrients [70]. The primary cell wall is composed mainly of cellulose, hemicellulose and pectin. Lignin, a highly cross-linked phenolic macromolecule, is the major component of the secondary cell wall. The pea plant cell wall is comprised of 27% cellulose, 32% hemicellulose and 41% pectin [71].

**Fig. 7:**
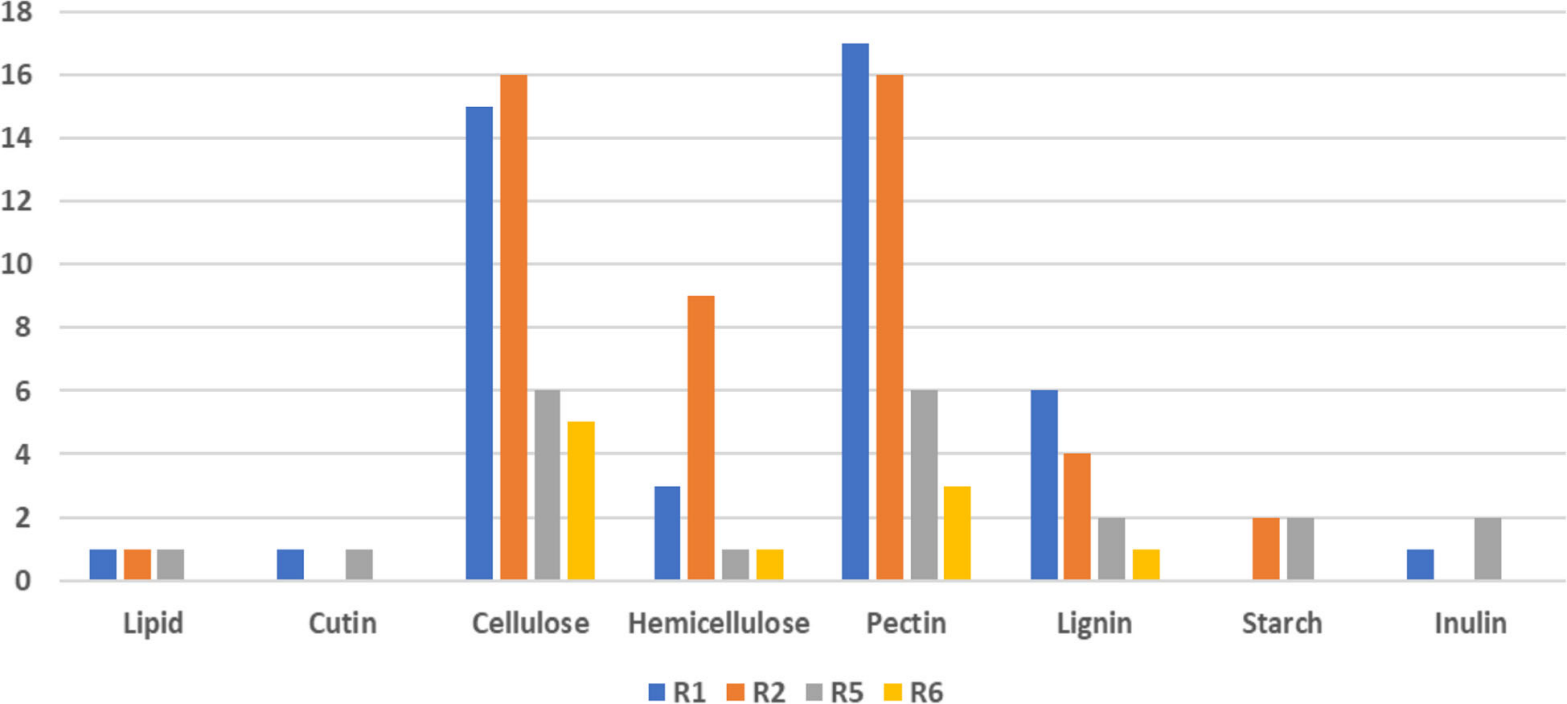
Number of genes encoding characterized and putative cell wall degrading enzymes in different races targeting different plant cell wall components

Cellulose is a homopolymer of beta-(1, 4)-linked D-glucose, which is sequentially hydrolysed into its component glucose by enzymes [72]. Genes encoding cellulose-degrading enzymes expressed by the different races of Fop included cellulose-binding domain proteins, glucose-methanol-choline oxidoreductases, exo/endo-glucanases, murien transglycosylase, glycosyl hydrolases (GH), lytic polysaccharides mono-oxygenase, xyloglucanase and β-glucosidase (Supplementary Tables 13, 14, 15 and 16). Genes encoding cellubiose dehydrogenase, responsible for degrading cellulose and lignin, were expressed only in R1. Races 1, 2, 5 and 6 expressed 15, 16, six and five genes encoding cellulose-degrading proteins, respectively (Fig. 7, Supplementary Tables 13, 14, 15 and 16). There was no significant difference (Fisher’s LSD *p* ≤ 0.05) in the number of DEGs encoding cellulose degrading proteins between R1 and R2 and R5 and R6. Nine of the cellulose degrading proteins had sequence homology to the virulence-associated proteins encoded by MoCDIP4 (PHI:3216) [27] and endo 1_4-beta-xylanase (PHI:2207) [73] genes in *M. oryzae* (Supplementary Tables 5, 6, 7 and 8). MoCDIP4 plays the role of a cell death-inducing effector in *M. oryzae* [27] and xylanase was essential for *in planta* expansion of *M. oryzae* in infected rice plants [73].

The hemicellulose component, which makes up 32% of the pea plant cell walls, consists of substantial amounts of glucose, xylose and xyloglucans [71]. Genes encoding hemicellulose degrading proteins in Fop included alpha-mannosidase, bifunctional xylanase/deacetylase, endo-xylanase, endoglucanase, glycosyl hydrolases, xyloglucanase, xylosidase and galactosidases. The number of DEGs encoding hemicellulose degrading enzymes expressed in races 1, 2, 5 and 6 were three, nine, one and one, respectively (Fig. 7, Supplementary Tables 13, 14, 15 and 16). There was a significant difference (Fisher’s LSD *p* ≤ 0.05) in the number of DEGs between R2 and the others.

Eight of the hemicellulose degrading proteins had sequence homology to the virulence-associated proteins encoded by tom1 (PHI:2364) gene in Fo [41], GAS1 (PHI:526) gene in *Ustilago maydis* [74], endo-1_4-beta-xylanase (PHI:2207) [73], CBP1 (PH:4639) [75] and MoGls2 (PHI:6739) genes in *M. oryzae* [76] and Fgleu1 (PHI:9357) gene in *F. graminearum* [77] (Supplementary Tables 5, 6, 7 and 8). Tomatinase enzyme, tom1, is not essential for pathogenicity in Fol but is necessary for full virulence [41]. Tom1 is secreted by Fol to degrade α-tomatine, a phytoalexin produced by tomato plants, to a less toxic derivative [41]. GAS1 and MoGls2 are involved in fungal growth and development in infected plants [76, 78]. CBP1 is essential for hydrophobic surface sensing during appressorium differentiation in *M. oryzae* [75]. Fgleu1 is involved in leucine biosynthesis and is essential for wheat head blight disease caused by *F. graminearum* [77]. There was a significant difference (Fisher’s LSD *p* ≤ 0.05) between R2 and the others with R1, R2, R5 and R6 expressing 3, 7, 1, 1, DEGs encoding hemicellulose degrading enzymes, respectively.

Pectin, the major component in the pea plant cell wall, contains uronic acid, a substantial amount of rhamnose, rhamnogalacturonan, and rhamnogalacturonan-associated galactose and arabinose polymers [71]. DEGs encoding pectin degrading proteins expressed by Fop races included endo/exo-polygalacturonases, endo-xylogalacturonan hydrolases, glycosyl hydrolases, pectate and pectin lyases, pectinesterase, rhamnogalacturonan lyases and hydrolases, unsaturated glucuronyl hydrolases, α-1,4-galacturonidases, carbohydrate esterases and arabinofuranosidases. Endo-polygalacturonases (PGs) and xylanases have been shown to play important roles during pathogenesis by some fungal pathogens of dicot plants [79], and this agrees with the fact that cell walls of dicots are composed of higher levels of pectin than monocots [80]. The number of DEGs encoding pectin degrading proteins expressed by races 1, 2, 5 and 6 were 17, 16, six and three, respectively (Fig. 7, Supplementary Tables 13, 14, 15 and 16). There was a significant difference (Fisher’s LSD *p* ≤ 0.05) in the number of DEGs encoding pectin degrading proteins between all the races except between R1 and R2. Thirteen of the pectin degrading proteins had sequence homology to the virulence-associated proteins encoded by PELA (PHI:179) and PELD (PHI:180) genes in *F. solani* [81], PELB (PHI:222) gene in *C. gloeosporioides* [82], Pg1 (PHI:7283) gene in *F. graminearum* [79], Pcipg2 (PHI:2343) gene in *Phytophthora capsici* [83], and PiGPB1 (PHI:316, PHI:4606) gene in *Phytophthora infestans* [84] (Supplementary Tables 5, 6, 7 and 8). All these mapped genes are involved in pectin degradation except PiGPB1 in *P. infestans,* which is involved in cell maintenance [79, 81–84].

Lignin in the plant cell walls provides defence against fungal pathogens [85]. A study of *F*. *graminearum* infection of wheat showed that lignin provided strength to the plant cell walls and played an essential role in restricting *Fusarium* development [86]. During *Fusarium* colonisation, the breakdown of cell walls surrounding the infected cells is required for toxin diffusion and enables the pathogen to obtain nutrients [87]. Lignin serves as the barrier against further infection progression [86]. A similar role of lignin has been reported in resistant pea cultivars against Fop in a histological study [7]. DEGs encoding lignin degrading proteins expressed in Fop races included laccases, glyoxal oxidases, cellobiose dehydrogenases, proteins with multicopper oxidase domain, galactose oxidases and glycoside hydrolases. Laccase has a three multicopper oxidase domain and is one of the first in the line of proteins expressed during the fungal catabolism of lignin [88]. DEGs encoding laccase proteins were only expressed in R1 and R5 (Supplementary Tables 13 and 14).

Races 1, 2, 5 and 6 had six, four, two and one DEGs encoding lignin degrading proteins, respectively (Fig. 7, Supplementary Tables 13, 14, 15 and 16) with a significant difference (Fisher’s LSD *p* ≤ 0.05) between all the races except between R5 and R6. Six of these proteins had sequence homology to the virulence-associated proteins encoded by the GLX (PHI:5393) gene in Fo, FET3–1 (PHI:2920, PHI:9074) and FET3–2 (PHI:2921, PHI:9075) genes in *Colletotrichum graminicola* [89], treZ gene (PHI:2746) in *Pseudomonas aeruginosa* [90] and XC_0423 gene (PHI:3958) in *Xanthomonas campestris* [91] (Supplementary Tables 5, 6 and 7). GLX, a glyoxal oxidase, has been found to play multiple roles in Fo. It is involved in mycotoxin production and lignin degradation pathways and provides defence against reactive oxygen species (ROS) [46]. The FET3–1 and FET3–2 in *C. graminicola* are related to iron availability during the infection process [89]. TreZ in *P. aeruginosa* is involved in acquiring nitrogen-containing nutrients by degrading the xyloglucan component of the plant cell wall, thereby allowing the bacteria to replicate in the intercellular spaces [90]. XC_0423 in *X. campestris* encodes for a protein within a group of proteins belonging to different functional categories, including biosynthesis and intermediary metabolism, regulation, oxidative stress, antibiotic resistance, and DNA replication [91].

Inulin is a naturally occurring polysaccharide that belongs to a class of carbohydrates known as fructans. Just as most plants store starch as reserve carbohydrates, about 15% of all flowering plant species store fructans [92]. Some of the plants that store fructans include cereals (e.g. barley, wheat and oat), vegetables (e.g. chicory, onion and lettuce), ornamentals (e.g. dahlia and tulip), forage grasses (e.g. *Lolium* and *Festuca*) [93] and peas [94]. DEGs encoding inulin degrading proteins included a hypothetical protein belonging to AA11 class, beta-fructofuranosidase and invertases, which were only expressed in R1 and R5 (Supplementary Tables 13 and 14), while DEGs encoding for 1,6 alpha-glucosidase, glycosyl hydrolase and 1,4 alpha-glucan-branching protein were expressed in R2 and R5 for starch degradation (Supplementary Tables 14 and 15) with no significant difference (Fisher’s LSD *p* ≤ 0.05).

In summary, fungal pathogens need to degrade plant cell walls to colonise host tissues and access cell nutrients. Plant cell wall degrading enzymes (PCWDEs) have been identified as crucial pathogenicity factors that contribute to successful Fo infection in tomato [95]. In several plant pathogenic fungi, cell wall degrading enzymes (CWDEs) such as pectinases and xylanases were demonstrated to be involved in pathogenicity or virulence [96, 97]. Since pectin is the main component of the pea plant cell walls, it would be expected that phytopathogens infecting pea would express more pectin degrading enzymes in their suite of PCWDEs. More DEGs encoding for pectin degrading proteins were expressed in R1 and R2; therefore, they would be better at degrading the pea plant cell walls, which may also contribute to their virulence. Alternatively, the differences between the races may also be due to different races expressing the pectin degrading genes at different time points during the necrotrophic infection stage.

All four races expressed genes encoding for proteins responsible for the degradation of cellulose, hemicellulose, pectin and lignin (Fig. 7), all components of pea cell walls suggesting that for pea colonisation, degradation of all these components are essential. However, virulence may be related to the number of different enzymes targeting the degradation of plant cell walls. R1 and R2, expressing 44 and 48 genes encoding PCWDEs, respectively, may be better at degrading cell walls than R5 and R6, which expressed 21 and 10 genes encoding PCWDEs, respectively. R6 secreted the least amount of PCWDEs out of all the races because its infection mechanism may be to avoid the plant defence response. Hence its strategy may be to produce enough enzymes to cause infection and get nutrients but still be safe from activating the plant’s defence responses. When the phytopathogens are secreting the PCWDEs, these enzymes or their degraded products may also act as elicitors for inducing plant defence responses [98]. However, the differences in the DEGs encoding PCWDEs between the races may also be due to the four races having different growth rates and therefore being at different stages of infection. Due to differences in growth rates, they may express the genes at different time points. R5 and R6 may have expressed more genes encoding for PCWDEs earlier than 20 dpi or may express at a later time point. This is supported by the fact that all the races exhibited the same external and internal symptoms at 20 dpi. Mc Phee et al. [5] had reported that disease symptoms in race 2 progress slowly and plant death occurs in the very late stages of the disease process. In this study, all the races had similar disease symptoms when the experiment was terminated at 20 dpi; the disease progression till the death of the plants were not studied.

## Conclusions

The aim of this study was to use a transcriptomics approach to study the Fop-pea pathosystem for the four Fop races 1, 2, 5 and 6 over the disease infection phase. The first time point was to be in the late biotrophic stage (5 dpi) and the second time point was to be in the necrotrophic stage (20 dpi). Both the shoot and root tissues were to be analysed for these time points. However, due to relatively fewer reads mapping to the fungal genome at 5 dpi, only the later time point of the necrotrophic stage could be studied in a susceptible pea host.

At this time point, all the races had shown similar levels of Fop colonisation in the basal stem region of the host plants (Fig. 2b), and they all had exhibited external necrotic symptoms (Fig. 1d) with grey-green discolouration and chlorosis of the lower leaves, which had started to extend upwards. The lower chlorotic leaves curled downwards and became flaccid. Internal red-brown discolouration of the xylem vessel was visible at five dpi in all the races. At 20 dpi, all the races had a more extensive section of the xylem vessel discoloured, but the discolouration remained in the primary root and the basal stem region. Most of the lateral roots and the root hairs in all the races had decayed at this stage of infection. There were many more fungal RNA Seq reads obtained from the root tissues than the shoot tissues for all the races.

Despite having similar internal and external infection symptoms, there were significant differences in the number of DEGs between the races, with R5 having the highest. Some DEGs were common to all the races. R2 and R5 had more common DEGs and effector-like proteins. R6 had the least number of DEGs, therefore portraying a “stealth” infection strategy, and this technique may be utilised to avoid detection and activation of the plant defence mechanisms. Alternatively, R6 may have a different time point for gene expression compared to the other races, and this time point has not been captured. It could also be that R6 can cause the same amount of disease symptoms but with fewer genes expressed. This needs to be confirmed with multiple isolates and time-series.

While there were genes shared between the races, others were specific to each race, accounting for their pathogenicity/virulence variation. Although we could state the DEGs present in the races, we could not state with certainty that they are not expressed in the other races, such as in R6, at a different stage of the infection, as only a single time point and single isolate per race were used for the study. This is further supported by the presence of the several DEGs uniquely expressed by different races in the genomes of other races, suggesting that different races may have different gene expression times. The differences in the DEGs per race could also be due to different races having different *in planta* growth rates and hence may not be at the same stage of infection despite all being at 20 dpi.

Since the study was at the necrotrophic infection stage, many CAZymes, proteases, nutrient transporters, and toxins were released to degrade the plant tissues and enhance further colonisation. This study revealed that multiple proteins are involved in Fop pathogenicity, with a high percentage of them being HPs. Functional characterisation of these Fop genes needs to be carried out to confirm pathogenicity or virulence roles, and race-specific genes can be further explored for molecular characterisation of the races.

## Methods

### Fungal isolates and cultural conditions

*Fusarium oxysporum* f.sp. *pisi* race 1 isolate RBG6462, race 2 isolate RBG6423, race 5 isolate RBG6425 and race 6 isolate RBG6418 were single spored as described by Burgess et al. [99] and grown on Potato Dextrose Agar (PDA; Diffco Laboratories, Detroit) under dark incubation for 5 days at 25 °C before using them for preparing inoculum. An earlier study on the phylogenetic relationships of these isolates has placed RBG6462 in clade 3 while the other three isolates were in clade 2 within the FOSC [2]. Further analysis later predicted RBG6462 as ‘species’ 3 while the other three isolates were assigned as ‘species’ 2 within the FOSC [2].

### Pea cultivar and growing conditions

*Pisum sativum* cultivar Kelvendon Wonder was used as it is susceptible to infection by all the four Fop races. Pea seeds were surface sterilised for 20 min in a 20% sodium hypochlorite solution and then rinsed three times with sterile water. The seeds were wrapped in wet filter paper in a Petri dish, stratified for 2 days at 4 °C in the dark and then incubated at 26 ± 2 °C until germination. Once germinated, the seedlings were transferred to pots containing standard potting mix (Bio-Gro, South Australia) with some additives to the 30 L bag of potting mix (0.5 L Vermiculite (coarse), 0.5 L Perlite (coarse), 35 g Macracote Coloniser Plus 4-months slow-release fertiliser (15 N:3P:9 K), 30 g Nitrogen slow-release fertiliser (40 N,0P:0 K), 25 g water-holding granules, 15 g Trace elements (6 Mg,6.5Fe:5.4S:1.5Mn:0.4Zn:0.14B:0.07Mo) and 5 g Garden lime). The plants were grown in a growth cabinet with controlled environmental conditions under a 16 / 8 h light-dark photoperiod at 26 ± 2 °C. Plants were watered with tap water every 3 days. There were four replications and four treatments (four races) and control with five plants per replication and two time points after inoculations.

### *F. Oxysporum* disease assays

For consistent infection between the replicate plants, millet grains (*Pennisetum glaucum*) pre-colonised with the different Fo f.sp. *pisi* races were used. The procedure is as described by Smith et al. [100]. To prepare the inoculum, millet grains were rinsed in distilled water and soaked in distilled water overnight. The grains were then rinsed with distilled water and drained of any excess water. Erlenmeyer flasks (2 L) containing 500 g millet seeds were sterilised in an autoclave for 30 min. When the grain was cold, each flask was inoculated with four plugs of 1 cm X 1 cm colonised PDA cut from a five-day-old culture plate. For inoculation of the control plants, uninoculated sterilised millet grains were used. The flasks with the millet grains were shaken once daily for 2 weeks for even colonisation of the fungus.

Seven-day-old Kelvendon Wonder seedlings (2–3 node stage) were uprooted carefully, 2 g of the inoculum (inoculated millet grains) were added and mixed with the soil, and the seedlings were re-potted. Millet grains without fungus were used for the control plants. The seedlings were returned to the same growing conditions as previously in the same growth cabinet.

### Detection of internal symptoms

Internal red-brown discolouration of the xylem vessel is associated with *F. oxysporum* infection in field peas [4]. To observe this red-brown discolouration within the pea plant tissue, the basal and middle part of the stem and the upper part of the root system of two plants from each treatment (races) and both the time points, 5 dpi and 20 dpi and control were dissected and examined.

### Plant staining

To detect fungal colonization *in planta*, the plants were prepared as described by Bani et al. [101]. Briefly, two of the plants per replicate at 5 dpi and 20 dpi and control were harvested, washed with sterile water to remove any unadhered Fop microconidia, cleared with 2.5% KOH at 90 °C for 1 h, rinsed twice with deionized water and incubated overnight at room temperature in a solution of 1% HCl. The samples were then stained in a 1% Parker blue Quink ink aqueous solution for 30 min at 60 °C and destained for 16 h at room temperature in lactoglycerol. The resulting stained tissues were stored at room temperature in 100% glycerol until observation under a microscope. Following this treatment, stained fungal structures were clearly visible.

### *In planta* and in vitro fusarium RNA extraction, library preparation and sequencing

Four biological replicates per race of *in planta* samples were used for RNA extraction for each time point. The plants were washed in tap water to remove any soil. Shoot and root tissues were collected and snap-frozen in liquid nitrogen and stored at − 80 °C.

Four biological replicates were also taken for in vitro fungal samples. The four isolates were grown as in section 1. Two culture plugs were cut out and transferred into 45 ml of Potato Dextrose Broth (PDB; Diffco Laboratories, Detroit). These tubes were placed onto a Ratek orbital shaker/mixer and gently shaken at 7RPM in the dark for 3 days. The resultant mycelia were harvested, snap-frozen in liquid nitrogen, and stored at − 80 °C.

Total RNA for all the samples was extracted using the Qiagen RNeasy mini kit (Qiagen, Mississauga, Canada). The RNA quality and quantity were accessed using a NanoDrop ND-1000 spectrophotometer (NanoDrop Technologies). RNA samples with 260/280 ratios of ~ 1.8 were used for downstream library preparation. NEXTFLEX® Poly(A) Beads 2.0 (PerkinElmer Applied Genomics) were used to extract mRNA from five μg of total RNA per sample. Individual barcoded cDNA libraries were prepared from 14 μl of mRNA (5–100 ng) using NEXTFLEX® Rapid Directional RNA-Seq Library Prep Kits (PerkinElmer Applied Genomics). Individual libraries were quantified using a Quantus™ Fluorometer (Promega), and the quality of the libraries (product insert size, primer and adapter dimers) were assessed on an Agilent 4200 Tape Station using Agilent High Sensitivity D1000 screen tape. The libraries were pooled together into a single library based on uniform molarity per sample to ensure even reads per sample. The final library was sequenced on an Illumina NovaSeq 6000.

### Identification of differentially expressed genes

Low-quality reads (<Q20) and adaptor sequences from the fastq sequence files were filtered using fastp [102]. After read processing (quality trimming and adaptor removal), an average of ~ 60 million paired-end reads were generated per sample. The genomic assemblies of Fop race 1 (RBG6462) (WGPO00000000.1), race 2 (RBG6423) (WGPE00000000.1), race 5 (RBG6425) (WGPF00000000.1) and race 6 (RBG6418) (WGOZ00000000.1) were part of an earlier study [2] and are available on NCBI GenBank. De novo assemblies of RNA-Seq data were also created using Trinity [103] to capture any genes that may be upregulated *in planta* but missing in the genomic assembly.

To determine the abundance of reads mapping to a gene in the genome, the RNA-Seq reads were mapped to the respective Fop race assemblies (genomic and transcript assemblies) using *Salmon* 1.3.0 (https://github.com/COMBINE-lab/salmon) [104]. Transcript abundance generated from Salmon (https://github.com/COMBINE-lab/salmon) [104] data was used by Sleuth v0.27.3 (http://pachterlab.github.io/sleuth) [105] in R with default settings to normalise and find the differentially expressed genes between each dataset (in vitro and infected tissue for each time point). The default filtering function in Sleuth (called basic_filter) requires at least five mapped reads per transcript in at least 47% of the samples. To identify DEGs with high potential for involvement in pathogenicity, we set out to identify Fop genes differentially expressed between in vitro and *in planta* and upregulated *in planta* with the premise that genes involved in Fop pathogenicity would be switched on or more highly upregulated in a suitable host [14, 22].

An adjusted *P* value < 0.05 and |log2(fold change)| > 1 [106] were chosen as the cut-off criteria to identify differentially expressed genes. The amino acid sequences of the differentially expressed genes were extracted from the respective assembled and protein annotated genomes. The transcripts were identified using their amino acid sequences against the NCBI protein database using Diamond BLASTX [107]. The Venn diagrams of the DEGs were created using Venny 2.1.0 [108].

### Gene expression profiling analyses

#### Identification of protein families

The protein families of all the transcripts were annotated using conserved domain information from InterProScan 5 [109] with E-value <E-10. CAZymes were identified and classified using the dbCAN meta server [110] (http://bcb.unl.edu/dbCAN2) [accessed on 08/2020] with the default settings and if predicted by more than two predicting tools.

#### Functional enrichment for biological process (BP)

The role of the proteins in the host-pathogen interactions was identified using Gene Ontology (GO) annotations for the BP using the eggNOG 5.0 http://eggnog-mapper.embl.de [111] [accessed on 17/08/2020] using the default settings and InterProScan 5 [109].

#### Prediction of secretory and putative effector proteins

The presence of a signal peptide was detected using the SignalP-5.0 program (http://www.cbs.dtu.dk/services) [112] [accessed on 25/08/2020]. The SecretomeP1.0 program (http://www.cbs.dtu.dk/services/SecretomeP-1.0/) [113] [accessed on 25/08/2020] was used to confirm the secretion of proteins through the unconventional pathways as used by Jain et al. [114]. The subcellular localisation of the secretory proteins was predicted using DeepLoc-1.0 (http://www.cbs.dtu.dk/services) [115] [accessed on 25/08/2020]. The transmembrane helices of the proteins were predicted using online TMHMM Server v. 2.0 (http://www.cbs.dtu.dk/services/TMHMM/) [accessed on 25/08/2020] to discriminate between soluble and membrane proteins. PredGPI [116] http://gpcr2.biocomp.unibo.it/predgpi/info.htm [accessed on 25/08/2020] was used for the prediction of GPI anchored proteins. Effector-like proteins were predicted from the secretory proteins using Effector2P (http://effectorp.csiro.au/) [32] [accessed on 07/2020].

#### Identification of VAGs and proteins with sequence homology

To identify the genes encoding for proteins that mapped to the virulence-associated proteins or that had a sequence homology to VAGs on the PHI-base database, a BLASTp analysis of the transcripts was carried out against the PHI base database [38] with identity > 25, E-value: 1e-10 as used by Jing et al. [117]. Only proteins involved in pathogenicity from phytopathogens were retained.

Statistical software OriginPro 2019 (www.originlab.com) was used to carry out Fisher’s Test for Least Significant Difference.

## Supplementary Information

**Additional file 1: Fig. S1.** Venn diagram of Fop genes differentially expressed between in vitro and *in planta* and upregulated in the root (R) and shoot tissues (S) at 20 dpi for the four races.

**Additional file 2: Supplementary Fig. S2.** Analysis of the effector-like proteins in all the races. 2A-amino acid sequence length, 2B- Effector-like proteins secretory pathways, 2C-localisation of the effector-like proteins.

**Additional file 3: Table S1.** Differentially expressed Fop genes detected in R1 at 20 dpi - column 1 with other analyses such as: predicted proteins - column 2, conserved domain - column 3, log_2_fold change - column 4, subcellular localisation of the effector-like proteins - column 5, protein length - column 6, and GO functional enrichment for biological processes (BP) - column 7. DEGs predicted to be effector-like are shaded yellow and they were all located on the adaptive genome.

**Additional file 4: Table S2.** Differentially expressed Fop genes detected in R2 at 20 dpi - column 1 with other analyses such as: predicted proteins - column 2, conserved domain - column 3, log_2_fold change - column 4, subcellular localisation of the effector-like proteins - column 5, protein length - column 6, and GO functional enrichment for biological processes (BP) - column 7. DEGs predicted to be effector-like are shaded yellow and they were all located on the adaptive genome.

**Additional file 5: Table S3.** Differentially expressed Fop genes detected in R5 at 20 dpi - column 1 with other analyses such as: predicted proteins - column 2, conserved domain - column 3, log_2_fold change - column 4, subcellular localisation of the effector-like proteins - column 5, protein length - column 6, and GO functional enrichment for biological processes (BP) - column 7. DEGs predicted to be effector-like are shaded yellow and they were all located on the adaptive genome.

**Additional file 6: Table S4.** Differentially expressed Fop genes detected in R6 at 20 dpi - column 1 with other analyses such as: predicted proteins - column 2, conserved domain - column 3, log_2_fold change - column 4, subcellular localisation of the effector-like proteins - column 5, protein length - column 6, and GO functional enrichment for biological processes (BP) - column 7. DEGs predicted to be effector-like are shaded yellow and they were all located on the adaptive genome.

**Additional file 7: Table S5.** Differentially expressed genes in R1 that mapped to the virulence-associated genes on the PHI-base database.

**Additional file 8: Table S6.** Differentially expressed genes in R2 that mapped to the virulence-associated genes on the PHI-base database.

**Additional file 9: Table S7.** Differentially expressed genes in R5 that mapped to the virulence-associated genes on the PHI-base database.

**Additional file 10: Table S8.** Differentially expressed genes in R6 that mapped to the virulence-associated genes on the PHI-base database.

**Additional file 11: Table S9.** CAZyme prediction of the differentially expressed genes in R1.

**Additional file 12: Table S10.** CAZyme prediction of the differentially expressed genes in R2.

**Additional file 13: Table S11.** CAZyme prediction of the differentially expressed genes in R5.

**Additional file 14: Table S12.** CAZyme prediction of the differentially expressed genes in R6.

**Additional file 15: Table S13.** Number of genes encoding characterized and putative cell wall degrading enzymes of the Fop R1 transcriptome. Note: Protein Ids are used under activities and where there was no name associated with the unigene, protein domain is provided and where there is no protein IDs and domains, HP is stated, denoting hypothetical protein with the CAZYme family associated with the amino acid sequences of the unigenes.

**Additional file 16: Table S14.** Number of genes encoding characterized and putative cell wall degrading enzymes of the Fop R5 transcriptome. Note: Protein Ids are used under activities and where there was no name associated with the unigene, protein domain is provided and where there is no protein IDs and domains, HP is stated, denoting hypothetical protein with the CAZYme family associated with the amino acid sequences of the unigenes.

**Additional file 17: Table S15.** Number of genes encoding characterized and putative cell wall degrading enzymes of the Fop R2 transcriptome. Note: Protein Ids are used under activities and where there was no name associated with the unigene, protein domain is provided and where there is no protein IDs and domains, HP is stated, denoting hypothetical protein with the CAZYme family associated with the amino acid sequences of the unigenes.

**Additional file 18: Table S16.** Number of genes encoding characterized and putative cell wall degrading enzymes of the Fop R6 transcriptome. Note: Protein Ids are used under activities and where there was no name associated with the unigene, protein domain is provided and where there is no protein IDs and domains, HP is stated, denoting hypothetical protein with the CAZYme family associated with the amino acid sequences of the unigenes.

## Data Availability

The data from this study are available from the NCBI Gene Expression Omnibus under accession number GSE159726.

## Abbreviations

AA: Auxiliary Activities
ABC: ATP-binding cassette
BP: Biological process
Bzip: Basic-leucine zipper
CAZymes: Carbohydrate-active enzymes
CBM: Carbohydrate-binding modules
CE: Carbohydrate esterase
CFEM: Common fungal extracellular membrane
CYP: Cytochrome P450s
DEGs: Differentially expressed genes
ff.spp.: *Formae speciales*
Fo: *Fusarium oxysporum*
Fol: *Fusarium oxysporum* f.sp. *lycopersici*
Fom: *Fusarium oxysporum* f.sp. *melonis*
Fop: *Fusarium oxysporum* f.sp. *pisi*
GH: Glycoside hydrolase
GO: Gene ontology
GTs: Glycosyltransferases
MAPKs: Mitogen-activated protein kinases
MFS: Major facilitator superfamily
PCWDE: Plant cell wall degrading enzymes
PLs: Polysaccharide lyases
RNS: Reactive nitrogen species
ROS: Reactive oxygen species
SMs: Secondary metabolites
TF: Transcription factor
TP: Timepoint
VAGs: Virulence-associated genes
