# Supplementary figures and images for: Comparative transcriptomic analysis of races 1, 2, 5 and 6 of Fusarium oxysporum f.sp. pisi in a susceptible pea host identifies differential pathogenicity profiles
Source: BMC Genomics. 2021 Oct 9;22:734. doi: 10.1186/s12864-021-08033-y (PMC8502283; doi:10.1186/s12864-021-08033-y)

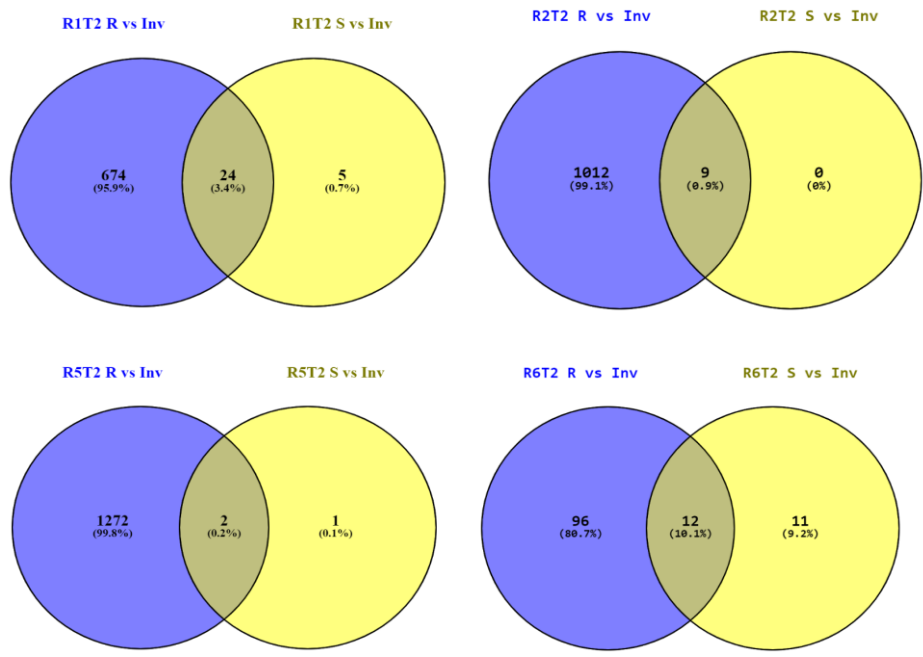

Supplement: Supplementary file 1 — Additional file 1: Fig. S1. Venn diagram of Fop genes differentially expressed between in vitro and in planta and upregulated in the root (R) and shoot tissues (S) at 20 dpi for the four races. [file 12864_2021_8033_MOESM1_ESM.pdf]

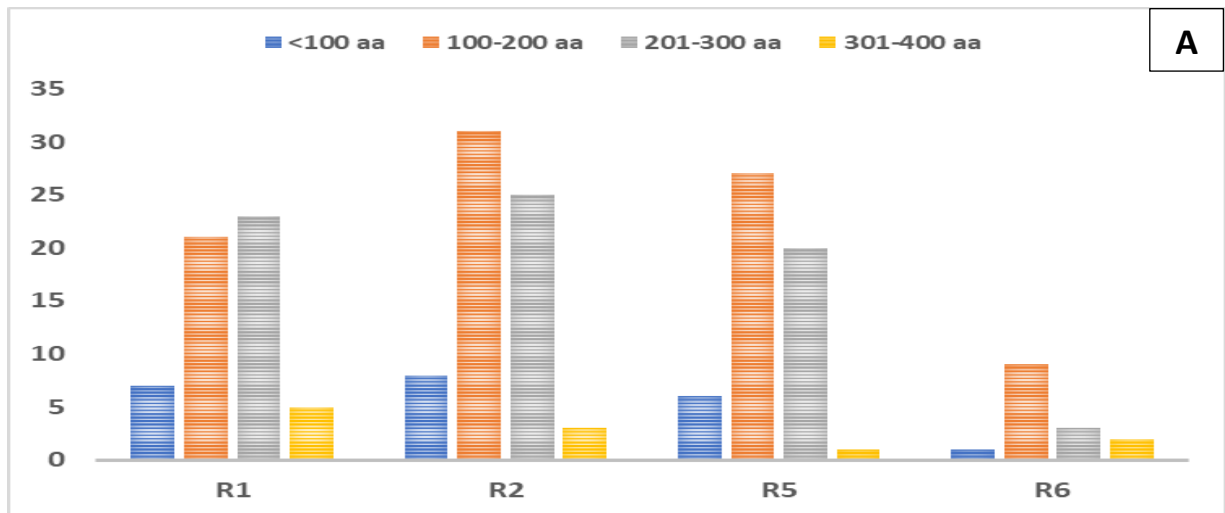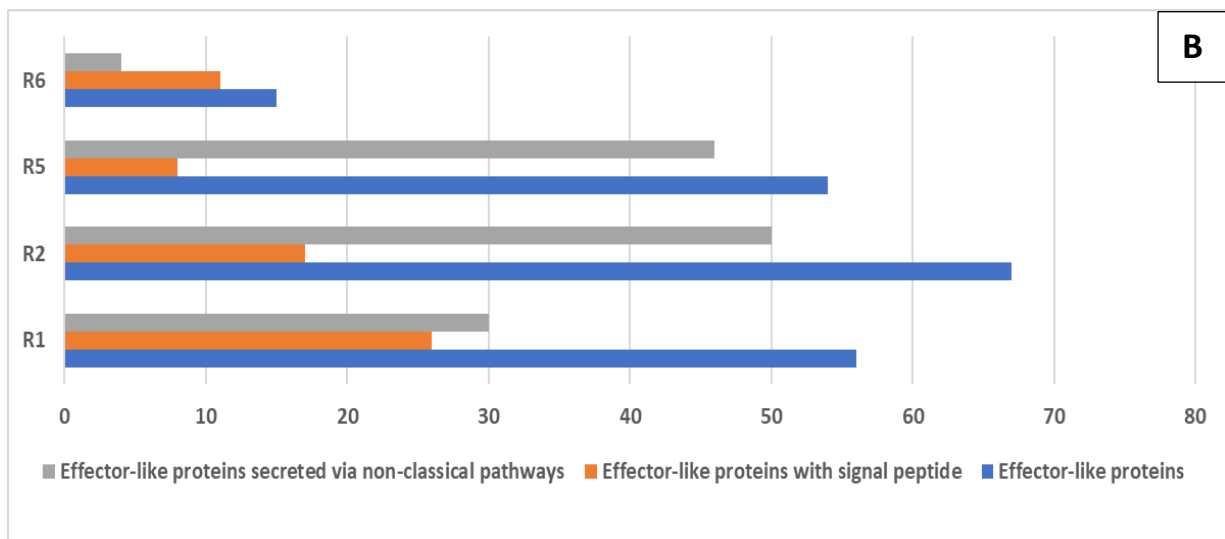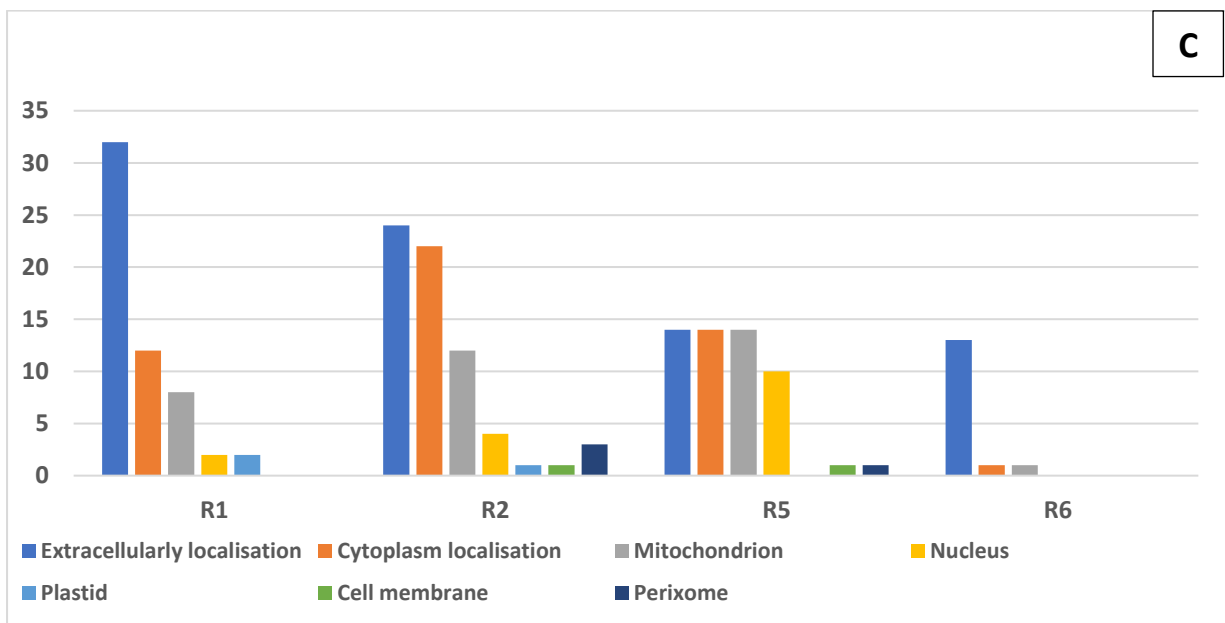

Supplement: Supplementary file 2 — Additional file 2: Supplementary Fig. S2. Analysis of the effector-like proteins in all the races. 2A-amino acid sequence length, 2B- Effector-like proteins secretory pathways, 2C-localisation of the effector-like proteins. [file 12864_2021_8033_MOESM2_ESM.pdf]
