# Supplementary material for: Comparative transcriptomic analysis of races 1, 2, 5 and 6 of Fusarium oxysporum f.sp. pisi in a susceptible pea host identifies differential pathogenicity profiles
Source: BMC Genomics. 2021 Oct 9;22:734. doi: 10.1186/s12864-021-08033-y (PMC8502283; doi:10.1186/s12864-021-08033-y)
Supplement: Supplementary file 3 — Additional file 3: Table S1. Differentially expressed Fop genes detected in R1 at 20 dpi - column 1 with other analyses such as: predicted proteins - column 2, conserved domain - column 3, log2fold change - column 4, subcellular localisation of the effector-like proteins - column 5, protein length - column 6, and GO functional enrichment for biological processes (BP) - column 7. DEGs predicted to be effector-like are shaded yellow and they were all located on the adaptive genome. [file 12864_2021_8033_MOESM3_ESM.docx]

**Supplementary Table 1**

| NODE_10.g470.t1 | hypothetical protein FOXG_18392 | Ribonuclease Z/Hydroxyacylglutathione hydrolase-like | 3.9 |  | 3515 | RNA processing |
| --- | --- | --- | --- | --- | --- | --- |
| NODE_101.g12451.t1 | uncharacterized protein FVRRES_11925 | Importin-alpha, importin-beta-binding domain | 1.3 |  | 552 | Transport |
| NODE_103.g12525.t1 | 50S ribosomal protein L2 | Ribosomal Proteins L2, RNA binding domain | 1.1 |  | 372 | Translation |
| NODE_103.g12546.t1 | saccharopine dehydrogenase | Saccharopine dehydrogenase, NADP binding domain | 1.1 |  | 450 | Oxidation-reduction |
| NODE_103.g12571.t1 | hypothetical protein FOCG_07937 | Lactonase, 7-bladed beta-propeller | 7.1 |  | 401 |  |
| NODE_103.g3079.t1 | Extracellular metalloproteinase MEP | Peptidase M36, fungalysin | 8.6 |  | 630 | Proteolysis |
| NODE_103.g3094.t1 | hypothetical protein BFJ65_g13442 | family decarboxylase | 4.4 |  | 384 |  |
| NODE_104.g3103.t1 | hypothetical protein FOC4_g10007778 | Phospholipase A2 domain superfamily | 4.0 | Extracellular | 183 | Metabolic |
| NODE_104.g3110.t1 | hypothetical protein BFJ65_g13082 | Galactose-binding-like domain superfamily | 6.2 |  | 261 |  |
| NODE_105.g12650.t1 | hypothetical protein FOC1_g10010447 | Zinc finger C2H2-type | 1.3 |  | 611 | Transcription |
| NODE_105.g12666.t1 | hypothetical protein FOXG_06223 | Pyridoxamine 5'-phosphate oxidase | 4.8 |  | 261 | Oxidation-reduction |
| NODE_106.g12698.t1 | Deoxyhypusine hydroxylase | L-lysine intermediate to form hypusine eIF-5A factor | 1.3 |  | 330 |  |
| NODE_107.g3183.t1 | Putative endo-beta-1,4-glucanase D | Cellulose-binding domain, fungal | 6.6 |  | 298 | Metabolic |
| NODE_107.g3193.t1 | gluconolactonase | SMP-30/Gluconolactonase/LRE-like region | 7.4 |  | 382 | Regulation of catalytic activity |
| NODE_108.g12769.t1 | probable adenylate kinase | Adenylate kinase/UMP-CMP kinase | 1.0 |  | 256 | Metabolic |
| NODE_108.g12789.t1 | hypothetical protein FOXG_11694 |  | 5.2 |  | 402 |  |
| NODE_108.g12792.t1 | hypothetical protein FOMG_14220 | Growth factor receptor cysteine-rich domain superfamily | 7.7 | Extracellular | 118 |  |
| NODE_108.g12808.t1 | hypothetical protein BFJ65_g16682 | Pectate lyase | 4.5 |  | 548 | Metabolic |
| NODE_109.g12879.t1 | Aldehyde dehydrogenase | Aldehyde dehydrogenase, C-terminal | 7.4 |  | 495 | Oxidation-reduction |
| NODE_111.g12948.t1 | related to triacylglycerol lipase | Alpha/beta hydrolase fold 3 | 2.2 |  | 386 | Metabolic |
| NODE_112.g12973.t1 | Putative 5-methyltetrahydropteroyltriglutamate--homocysteine methyltransferase | Cobalamin-independent methionine synthase MetE, C-terminal/archaeal | 2.4 |  | 766 | Biosynthetic |
| NODE_112.g12999.t1 | hypothetical protein FOTG_12292 | Cellulose-binding domain, fungal | 8.8 |  | 397 | Metabolic |
| NODE_114.g13081.t1 | adenylosuccinate synthetase | Adenylosuccinate synthetase | 2.5 |  | 409 | Biosynthetic |
| NODE_114.g13090.t1 | phosphatidylserine decarboxylase | Phosphatidylserine decarboxylase | 1.1 |  | 485 | Biosynthetic |
| NODE_114.g3330.t1 | citrate synthase, mitochondrial | Citrate synthase, eukaryotic type | 1.3 |  | 470 | Metabolic |
| NODE_114.g3331.t1 | Citrate/oxoglutarate carrier protein | Mitochondrial substrate/solute carrier | 1.4 |  | 318 | Transport |
| NODE_115.g13108.t1 | hypothetical protein BFJ69_g11872, partial | Ribosomal protein S19e | 2.1 |  | 139 | Translation |
| NODE_115.g13120.t1 | alpha-N-arabinofuranosidase B | Alpha-L-arabinofuranosidase B, arabinose-binding domain | 6.7 |  | 499 | Metabolic |
| NODE_115.g3357.t1 | hypothetical protein BFJ70_g2887 |  | 1.1 |  | 307 |  |
| NODE_116.g13162.t1 | ribose-phosphate pyrophosphokinase 3 | Ribose-phosphate pyrophosphokinase | 1.4 |  | 323 | Biosynthetic |
| NODE_116.g13177.t1 | related to 26S proteasome subunit RPN4 | Zinc finger C2H2-type | 3.0 |  | 622 | Transcription |
| NODE_116.g3376.t1 | hypothetical protein FOXG_01433 | Ribosomal RNA methyltransferase, Spb1, C-terminal | 1.5 |  | 794 | Methylation |
| NODE_116.g3382.t1 | putative nucleosome assembly protein | Nucleosome assembly protein (NAP) | 1.3 |  | 404 | Nucleosome assembly |
| NODE_116.g3394.t1 | hypothetical protein FPSE_09444 | Ribosomal protein S3Ae | 2.6 |  | 256 | Translation |
| NODE_117.g13208.t1 | related to DNA damage-responsive protein 48 |  | 5.5 |  | 109 |  |
| NODE_117.g3401.t1 | hypothetical protein FOC1_g10009972 |  | 3.3 |  | 331 |  |
| NODE_117.g3408.t1 | Putative glucose transporter rco-3 | Major facilitator, sugar transporter-like | 1.2 |  | 540 | Transport |
| NODE_118.g13250.t1 | hypothetical protein BFJ65_g8824 | Major facilitator superfamily | 6.3 |  | 434 | Transport |
| NODE_118.g13251.t1 | hypothetical protein BFJ69_g8042 | Pectin lyase fold | 6.2 | Extracellular | 391 | Metabolic |
| NODE_118.g3416.t1 | seryl-tRNA synthetase | Aminoacyl-tRNA synthetase, class II (G/ P/ S/T) | 1.5 |  | 477 | Translation |
| NODE_118.g3423.t1 | succinate-semialdehyde dehydrogenase (NADP+) | Aldehyde dehydrogenase, C-terminal | 2.3 |  | 494 | Oxidation-reduction |
| NODE_119.g13295.t1 | hypothetical protein BFJ65_g11457 | Ribonuclease Nob1, eukaryote | 1.2 |  | 452 | Ribosomal small subunit biogenesis |
| NODE_119.g13297.t1 | S-adenosylmethionine decarboxylase proenzyme | S-adenosylmethionine decarboxylase | 1.7 |  | 494 | Biosynthetic |
| NODE_119.g13324.t1 | hypothetical protein FPSE_08287 | Ribosomal protein S24e | 2.3 |  | 136 | Translation |
| NODE_12.g541.t1 | Uncharacterized protein Y057_6605 | Lytic polysaccharide monooxygenase, cellulose-degrading | 3.9 |  | 131 | Metabolic |
| NODE_120.g13344.t1 | hypothetical protein BFJ70_g12794 | PAS fold 3 | 2.6 |  | 658 |  |
| NODE_121.g13417.t1 | probable zuotin | Chaperone J-domain superfamily | 1.4 |  | 346 | Stress response |
| NODE_121.g3503.t1 | Minor extracellular protease vpr | Peptidase S8/S53 domain | 6.9 |  | 873 | Proteolysis |
| NODE_121.g3504.t1 | Laccase | Multicopper oxidase, type 2 | 6.3 |  | 658 | Oxidation-reduction |
| NODE_122.g13429.t1 | hypothetical protein BFJ71_g3584 | Cys/Met metabolism, pyridoxal phosphate-dependent enzyme | 6.7 |  | 406 | Transsulfuration |
| NODE_122.g13452.t1 | hypothetical protein BFJ69_g6430 | Haem peroxidase superfamily | 4.5 |  | 1426 | Proteolysis |
| NODE_122.g3508.t1 | Alkaline proteinase | Peptidase S8/S53 domain | 8.3 |  | 413 | Proteolysis |
| NODE_123.g13497.t1 | probable beta karyopherin | Importin repeat 6 | 1.7 |  | 1096 | Transport |
| NODE_125.g3578.t1 | hypothetical protein FOXG_12080 |  | 7.5 |  | 769 |  |
| NODE_125.g3579.t1 | hypothetical protein FOCG_15008 |  | 6.8 |  | 527 |  |
| NODE_126.g13617.t1 | hypothetical protein FOC4_g10004204 | RmlC-like cupin domain superfamily | 2.4 |  | 189 |  |
| NODE_126.g13618.t1 | hypothetical protein BFJ69_g6466 | integral membrane protein | 4.7 |  | 389 |  |
| NODE_127.g13681.t1 | S-(hydroxymethyl)glutathione synthase | Glutathione-dependent formaldehyde-activating enzyme | 1.3 |  | 227 | Catabolic |
| NODE_127.g13687.t1 | hypothetical protein FOCG_04532 | Haloacid dehalogenase-like hydrolase (HAD superfamily) | 2.7 |  | 425 |  |
| NODE_128.g3637.t1 | ribose-phosphate pyrophosphokinase | Ribose-phosphate pyrophosphokinase | 2.2 |  | 461 | Biosynthetic |
| NODE_13.g614.t1 | hypothetical protein FOC4_g10009946 |  | 7.4 |  | 195 |  |
| NODE_130.g13813.t1 | hypothetical protein FOC1_g10008301 | U3 small nucleolar RNA-associated protein 8 | 1.3 |  | 903 | rRNA processing |
| NODE_131.g13830.t1 | catalase-peroxidase | Haem peroxidase | 1.1 |  | 761 | Proteolysis |
| NODE_131.g13839.t1 | hypothetical protein FOXG_10535 | Phosphoribosylglycinamide synthetase, C-domain | 1.7 |  | 667 | Biosynthetic |
| NODE_132.g13872.t1 | hypothetical protein BFJ65_g7235 | serine-rich protein | 1.6 |  | 851 |  |
| NODE_135.g13955.t1 | pectate lyase | Pectate lyase PlyH/PlyE-like | 7.3 | Extracellular | 240 | Metabolic |
| NODE_135.g13958.t1 | hypothetical protein BFJ65_g11160 | Heat shock factor (HSF)-type, DNA-binding | 1.2 |  | 586 | Transcription |
| NODE_135.g3788.t1 | hypothetical protein FOQG_00510 | Sterile alpha motif/pointed domain superfamily | 2.5 |  | 827 |  |
| NODE_135.g3794.t1 | Pyruvate carboxylase | Pyruvate carboxylase | 2.9 |  | 1197 | Metabolic |
| NODE_136.g14003.t1 | hypothetical protein BFJ71_g7230 | High mobility group box domain | 1.7 |  | 93 |  |
| NODE_136.g14009.t1 | Xyloglucanase | Glycoside hydrolase family 74 | 7.5 |  | 733 | Metabolic |
| NODE_136.g14014.t1 | Adenylate cyclase | Adenylate cyclase G-alpha binding (cAMP) | 1.5 |  | 2264 | Biosynthetic |
| NODE_136.g14021.t1 | UDP-glucose 6-dehydrogenase | UDP-glucose/GDP-mannose dehydrogenase, dimerisation | 2.3 |  | 405 | Oxidation-reduction |
| NODE_137.g14050.t1 | hypothetical protein FLAG1_01408 |  | 2.7 |  | 69 |  |
| NODE_138.g14072.t1 | Phospho-2-dehydro-3-deoxyheptonate aldolase, tyrosine-inhibited | DHAP synthase, class 1 | 2.8 |  | 364 | Biosynthetic |
| NODE_138.g14074.t1 | hypothetical protein FOCG_09131 | Ribosomal protein L50, mitochondria | 1.2 |  | 380 | Translation |
| NODE_138.g14083.t1 | transcriptional regulator | Transcriptional regulator PAI 2-type | 7.5 |  | 253 | Transcription |
| NODE_139.g14117.t1 | Cytokinesis protein sepA | GTPase-binding domain (Formin) | 3.1 |  | 1022 | Actin cytoskeleton organization |
| NODE_14.g638.t1 | Putative pectate lyase F | Pectate lyase PlyH/PlyE-like | 3.9 | Extracellular | 233 | Metabolic |
| NODE_14.g646.t1 | hypothetical protein FOCG_08422 | Glyoxal oxidase, N-terminal | 4.8 |  | 902 | Oxidation-reduction |
| NODE_140.g3870.t1 | hypothetical protein BFJ66_g7752 | Growth factor receptor cysteine-rich domain superfamily | 8.7 | Extracellular | 297 |  |
| NODE_140.g3879.t1 | hypothetical protein BFJ71_g4060 | Aromatic amino acid beta-eliminating lyase/threonine aldolase | 3.9 |  | 353 | Metabolic |
| NODE_142.g14230.t1 | hypothetical protein BFJ67_g7392 | Calcium uniporter protein, C-terminal | 2.7 |  | 477 | Transport |
| NODE_143.g14261.t1 | serine hydroxymethyltransferase, mitochondrial | Serine hydroxymethyltransferase | 2.9 |  | 502 |  |
| NODE_143.g14262.t1 | probable GCV3-glycine decarboxylase, subunit H | Glycine cleavage system H-protein, subgroup | 1.7 |  | 172 | Glycine decarboxylation |
| NODE_145.g14316.t1 | hypothetical protein BFJ65_g4480 | Alkaline phosphatase | 4.1 |  | 655 | Protein dephosphorylation |
| NODE_145.g3964.t1 | hypothetical protein FLONG3_5678 |  | 3.8 |  | 120 |  |
| NODE_146.g14329.t1 | CAMKK protein kinase | Protein kinase domain-CAMKK | 1.8 |  | 1245 | Signal transduction |
| NODE_146.g3997.t1 | hypothetical protein FOMG_16806 |  | 5.1 |  | 360 |  |
| NODE_147.g14377.t1 | hypothetical protein FOXG_08482 | RGS domain | 1.8 |  | 713 | Signal transduction |
| NODE_149.g14416.t1 | probable YHM1 (mitochondrial carrier) | Mitochondrial substrate/solute carrier | 1.2 |  | 255 | Transport |
| NODE_149.g4059.t1 | Cytochrome c oxidase subunit 6, mitochondrial | Cytochrome c oxidase, subunit Va/VI | 1.4 |  | 146 | Oxidation-reduction |
| NODE_15.g691.t1 | murein transglycosylase | Glycoside hydrolase, family 61 | 6.1 |  | 349 | Metabolic |
| NODE_15.g696.t1 | related to nicotinamide mononucleotide permease | Major facilitator superfamily | 3.4 |  | 508 | Transport |
| NODE_15.g698.t1 | kynureninase 2 | Kynureninase | 2.8 |  | 493 | Biosynthetic |
| NODE_15.g706.t1 | hypothetical protein FOXG_05669 | SGNH hydrolase superfamily | 7.0 | Extracellular | 257 | Metabolic |
| NODE_15.g710.t1 | hypothetical protein BFJ68_g12732 |  | 7.5 |  | 304 |  |
| NODE_15.g714.t1 | hypothetical protein BFJ71_g6243 | Oligopeptide transporter, OPT superfamily | 7.2 |  | 742 | Transport |
| NODE_150.g14446.t1 | Mannitol-1-phosphate 5-dehydrogenase | Mannitol dehydrogenase, C-terminal | 2.4 |  | 391 | Oxidation-reduction |
| NODE_151.g4079.t1 | Putative helicase C6F12.16c | P-loop containing nucleoside triphosphate hydrolase | 1.4 |  | 1094 | Phosphorylation |
| NODE_153.g4124.t1 | probable woronin body major protein precursor | Ribosomal protein L2 | 1.4 |  | 606 | Translation |
| NODE_156.g4181.t1 | hypothetical protein FOXG_12843 | Nop domain | 1.1 |  | 593 | Ribosome biogenesis |
| NODE_157.g14665.t1 | hypothetical protein FOXG_14913 |  | 4.9 |  | 658 |  |
| NODE_158.g4218.t1 | probable iron inhibited ABC transporter 2 | ABC transporter-like | 1.1 |  | 618 | Transport |
| NODE_158.g4225.t1 | Calcium-dependent protein kinase 4 | Protein kinase domain | 5.2 |  | 542 | Signal transduction |
| NODE_159.g14710.t1 | probable IgE-dependent histamine-releasing factor | Translationally controlled tumour protein, conserved site | 2.5 |  | 170 | Translation |
| NODE_159.g14728.t1 | hypothetical protein FOTG_05904 | CCAAT-binding factor | 1.1 |  | 935 |  |
| NODE_159.g14731.t1 | probable MET14-ATP adenosine-5`-phosphosulfate 3`-phosphotransferase | Adenylyl-sulfate kinase | 2.6 | Plastid | 207 | Sulfate assimilation |
| NODE_16.g736.t1 | hypothetical protein BFJ71_g4155 |  | 4.9 |  | 392 |  |
| NODE_160.g4261.t1 | uncharacterized protein FMAN_11213 | CFEM domain | 1.1 |  | 195 |  |
| NODE_162.g14813.t1 | hypothetical protein BFJ69_g10229 | Arrestin, C-terminal | 1.8 |  | 421 |  |
| NODE_162.g4293.t1 | Galactose oxidase | Galactose oxidase | 6.0 |  | 679 | Oxidation-reduction |
| NODE_162.g4301.t1 | hypothetical protein FOC4_g10009509 | FAD dependent oxidoreductase | 2.2 |  | 564 | Oxidation-reduction |
| NODE_164.g14881.t1 | Cell wall synthesis protein psu1 | Beta-glucosidase (SUN family) | 2.2 |  | 439 | Metabolic |
| NODE_165.g14889.t1 | hypothetical protein BFJ68_g1045 | Zinc finger C2H2-type | 4.1 |  | 566 | Transcription |
| NODE_165.g14893.t1 | putative aminomethyltransferase, mitochondrial | Glycine cleavage system T protein | 1.3 |  | 439 | Catabolic |
| NODE_165.g14910.t1 | methionyl aminopeptidase | Peptidase M24A, methionine aminopeptidase, subfamily 2 | 1.1 |  | 385 | Proteolysis |
| NODE_166.g14923.t1 | Putative beta-glucosidase btgE | Glycoside hydrolase superfamily | 3.4 |  | 321 | Metabolic |
| NODE_166.g14935.t1 | hypothetical protein BFJ71_g675 | CFEM domain | 2.1 |  | 480 |  |
| NODE_167.g14952.t1 | hypothetical protein FPOA_07235 | S-adenosyl-L-homocysteine hydrolase, NAD binding domain | 1.3 |  | 449 | Metabolic |
| NODE_167.g14954.t1 | translation initiation factor 3 subunit J | Eukaryotic translation initiation factor 3 subunit J | 1.6 |  | 269 | Translation |
| NODE_167.g14967.t1 | hypothetical protein FOXG_15681 | Proton-dependent oligopeptide transporter family | 9.2 |  | 611 | Transport |
| NODE_168.g4399.t1 | hypothetical protein FOPG_03717 | Methyltransferase type 11 | 2.8 |  | 987 | Methylation |
| NODE_170.g15034.t1 | related to aldose 1-epimerase | Galactose mutarotase-like domain superfamily | 4.8 |  | 607 | Metabolic |
| NODE_170.g4444.t1 | hypothetical protein FPSE_00057 | Ribosomal protein S19/S15 | 3.0 |  | 152 | Translation |
| NODE_170.g4450.t1 | hypothetical protein FPSE_00059 | Alpha tubulin | 1.2 |  | 453 |  |
| NODE_170.g4451.t1 | Nucleolar protein 12 | RNA recognition motif domain | 1.8 |  | 562 | Cytokinesis |
| NODE_172.g4485.t1 | hypothetical protein FOMG_14594 | FAD linked oxidase, N-terminal | 7.6 |  | 573 | Oxidation-reduction |
| NODE_174.g15120.t1 | amidophosphoribosyltransferase | Amidophosphoribosyltransferase | 1.7 |  | 551 | Biosynthetic |
| NODE_175.g4533.t1 | uncharacterized protein FPRO_11845 | Zinc finger C2H2-type | 1.4 |  | 634 | Transcription |
| NODE_175.g4541.t1 | probable fibrillarin (NOP1) | Fibrillarin | 1.5 |  | 319 | rRNA processing |
| NODE_175.g4545.t1 | hypothetical protein BFJ69_g8254 | Nitroreductase Frm2/Hbn1-like | 2.2 | Mitochondrion | 205 | Stress response |
| NODE_176.g15157.t1 | hypothetical protein BFJ69_g10657 |  | 5.8 |  | 446 |  |
| NODE_176.g4568.t1 | hypothetical protein BFJ70_g5323 | Tetratricopeptide repeat | 2.6 |  | 1167 | RNA processing |
| NODE_178.g15190.t1 | hypothetical protein FPSE_02953 | Ribosomal protein S5/S7, eukaryotic/archaeal | 2.9 | Cytoplasm | 213 | Translation |
| NODE_178.g15207.t1 | hypothetical protein FOCG_13391 | FAD linked oxidase, N-terminal | 5.1 |  | 379 | Oxidation-reduction |
| NODE_180.g4632.t1 | HAL protein kinase | Protein kinase domain-HAL | 1.5 |  | 706 | Signal transduction |
| NODE_181.g4638.t1 | hypothetical protein FOMG_17002 | Haem peroxidase superfamily | 4.2 |  | 1455 | Proteolysis |
| NODE_182.g4648.t1 | hypothetical protein BFJ71_g2358 | Protein kinase domain-Histidine kinase, dimerisation/phosphoacceptor domain | 1.2 |  | 1228 | Signal transduction |
| NODE_183.g15276.t1 | hypothetical protein FOCG_04324 | CsbD-like domain | 1.8 |  | 181 |  |
| NODE_183.g4662.t1 | hypothetical protein FOCG_04805 |  | 8.0 |  | 921 |  |
| NODE_186.g4706.t1 | succinyl-CoA ligase | Succinate--CoA synthetase, beta subunit | 1.1 |  | 447 | Metabolic |
| NODE_186.g4709.t1 | hypothetical protein FOMG_08612 | Major facilitator superfamily | 2.1 |  | 502 | Transport |
| NODE_193.g15438.t1 | hypothetical protein BFJ68_g7612 | glycoside hydrolase family 17 protein | 4.2 |  | 99 | Metabolic |
| NODE_193.g4836.t1 | hypothetical protein FOTG_15008 | Pectinesterase, catalytic | 8.2 |  | 329 | Metabolic |
| NODE_193.g4838.t1 | hypothetical protein FOIG_14387 | Major facilitator, sugar transporter-like | 9.4 |  | 552 | Transport |
| NODE_197.g15488.t1 | hypothetical protein BFJ71_g15977 | Bestrophin, RFP-TM, chloride channel | 5.1 |  | 470 | Transport |
| NODE_198.g4908.t1 | related to F1F0-ATPase complex, subunit h | ATP synthase, F0 complex, subunit H | 1.1 |  | 128 | Transport |
| NODE_2.g88.t1 | hypothetical protein BFJ66_g5886 | Zn (2)-C6 fungal-type DNA-binding domain | 1.5 |  | 825 | Transcription |
| NODE_20.g878.t1 | hypothetical protein FOC1_g10005850 | Methyltransferase domain 25 | 1.7 |  | 622 | Methylation |
| NODE_20.g891.t1 | probable dihydroxy-acid dehydratase | Dihydroxy-acid dehydratase | 1.4 |  | 598 | Biosynthetic |
| NODE_200.g4940.t1 | hypothetical protein FOCG_08599 | Cellulose-binding domain, fungal | 6.2 |  | 837 | Metabolic |
| NODE_200.g4945.t1 | hypothetical protein FOCG_08606 | Aspartic peptidase A1 family | 3.5 |  | 384 | Proteolysis |
| NODE_201.g15537.t1 | Putative NADH-ubiquinone oxidoreductase C3A11.07, mitochondrial | Pyridine nucleotide-disulphide oxidoreductase | 1.5 |  | 561 | Oxidation-reduction |
| NODE_204.g15579.t1 | hypothetical protein FOXG_00490 | DNA polymerase V/Myb-binding protein 1A | 1.4 |  | 711 | Transcription |
| NODE_204.g15581.t1 | hypothetical protein FOXG_16600 |  | 3.5 |  | 164 |  |
| NODE_204.g5025.t1 | hypothetical protein FOXG_06225 |  | 2.8 |  | 1126 |  |
| NODE_205.g15593.t1 | related to quinate transport protein | Major facilitator, sugar transporter-like | 7.3 |  | 350 | Transport |
| NODE_206.g5056.t1 | hypothetical protein FOC1_g10016072 | Glycosyl hydrolase family 63, C-terminal | 2.6 |  | 1012 | Metabolic |
| NODE_207.g5069.t1 | hypothetical protein FOIG_12979 | Copper fist DNA-binding domain | 2.0 |  | 457 | Transcription |
| NODE_208.g5090.t1 | Ca2+-transporting ATPase | P-type ATPase, subfamily IIB | 3.1 |  | 1172 | Transport |
| NODE_210.g5106.t1 | uncharacterized protein FFUJ_04429 | Zinc finger, FYVE/PHD-type | 2.1 |  | 347 | Transcription |
| NODE_211.g5123.t1 | hypothetical protein FOCG_12216 |  | 2.7 |  | 640 |  |
| NODE_211.g5124.t1 | L-2-aminoadipate reductase large subunit | L-2-aminoadipate reductase | 1.1 |  | 1178 | Biosynthetic |
| NODE_211.g5127.t1 | hypothetical protein FOXG_11113 | Mitochondrial substrate/solute carrier | 2.9 |  | 321 | Transport |
| NODE_215.g5179.t1 | hypothetical protein FOTG_17235 | Cellobiose dehydrogenase, cytochrome domain | 4.8 |  | 385 | Metabolic |
| NODE_218.g15716.t1 | Serine/threonine-protein kinase psk1 | Protein kinase domain | 1.9 |  | 402 | Signal transduction |
| NODE_218.g5231.t1 | hypothetical protein BFJ66_g13254 | Galactose-binding-like domain superfamily | 7.0 |  | 483 |  |
| NODE_221.g15746.t1 | leucyl-tRNA synthetase, cytoplasmic | Methionyl/Leucyl tRNA synthetase | 1.0 |  | 477 | Translation |
| NODE_223.g5306.t1 | Inorganic phosphate transporter PHO84 | Major facilitator, sugar transporter-like | 3.4 |  | 567 | Transport |
| NODE_223.g5310.t1 | CTP synthase | CTP synthase GATase domain | 1.3 |  | 580 | Biosynthetic |
| NODE_225.g5355.t1 | F-type H+-transporting ATPase subunit B | ATP synthase, F0 complex, subunit B/MI25 | 1.7 |  | 242 | Transportation |
| NODE_226.g5363.t1 | Aromatic amino acid aminotransferase C56E4.03 | Aminotransferase, class I/classII | 4.9 |  | 532 | Biosynthetic |
| NODE_228.g5402.t1 | hypothetical protein BFJ70_g4004 | Eukaryotic molybdopterin oxidoreductase | 4.8 |  | 357 | Oxidation-reduction |
| NODE_232.g15822.t1 | hypothetical protein BFJ65_g10410 | CFEM domain | 3.7 |  | 397 |  |
| NODE_232.g5457.t1 | hypothetical protein BFJ68_g2259 | CFEM domain | 3.9 |  | 848 |  |
| NODE_234.g5483.t1 | DnaJ like subfamily A member 2 | Heat shock protein DnaJ, cysteine-rich domain | 2.2 |  | 434 | Stress response |
| NODE_234.g5487.t1 | NAD-specific glutamate dehydrogenase | Glutamate/phenylalanine/leucine/valine dehydrogenase, C-terminal | 1.4 |  | 1068 | Oxidation-reduction |
| NODE_235.g5493.t1 | hypothetical protein FOIG_07922 | guanine-nucleotide exchange factors catalytic domain (Ras-GTPase) | 1.5 |  | 1209 | Signal transduction |
| NODE_235.g5506.t1 | protein TIF31 | Tetratricopeptide repeat | 2.2 |  | 1263 | RNA processing |
| NODE_238.g15856.t1 | hypothetical protein BFJ65_g12047 |  | 8.5 |  | 115 |  |
| NODE_238.g5556.t1 | Peptidyl-prolyl cis-trans isomerase, mitochondrial | Cyclophilin-type peptidyl-prolyl cis-trans isomerase domain | 1.3 |  | 180 | Protein peptidyl-prolyl isomerization |
| NODE_24.g1048.t1 | hypothetical protein BFJ72_g11846 | Ribosomal protein L31e | 2.3 |  | 122 | Translation |
| NODE_242.g15872.t1 | hypothetical protein BFJ65_g4988 | Cellulose/chitin-binding protein, N-terminal | 4.8 | Extracellular | 117 | Metabolic |
| NODE_244.g15881.t1 | hypothetical protein BFJ65_g720 | glycosyl-phosphatidyl-inositol-anchored membrane family (GPI) | 2.5 |  | 212 | Proteolysis |
| NODE_247.g5700.t1 | hypothetical protein BFJ68_g11091 |  | 9.6 |  | 244 |  |
| NODE_248.g5723.t1 | hypothetical protein BFJ71_g14395 | GLEYA adhesin domain | 5.4 |  | 296 |  |
| NODE_249.g5729.t1 | hypothetical protein FOXG_01984 |  | 2.5 |  | 485 |  |
| NODE_249.g5730.t1 | hypothetical protein FAVG1_12529 | P-loop containing nucleoside triphosphate hydrolase | 2.1 |  | 396 | Phosphorylation |
| NODE_25.g1057.t1 | hypothetical protein BFJ68_g11938 | Class II aldolase/adducin N-terminal | 4.4 |  | 298 | Metabolic |
| NODE_254.g15920.t1 | hypothetical protein BFJ70_g2338 | Class I glutamine amidotransferase-like | 1.4 |  | 226 | Metabolic |
| NODE_254.g5803.t1 | Ketol-acid reductoisomerase, mitochondrial | Ketol-acid reductoisomerase, C-terminal | 1.6 |  | 405 | Oxidation-reduction |
| NODE_257.g5839.t1 | hypothetical protein FOCG_10316 | Carbohydrate-binding, CenC-like | 7.9 |  | 331 | Metabolic |
| NODE_258.g15931.t1 | hypothetical protein BFJ65_g14632 | Reverse transcriptase, RNA-dependent DNA polymerase | 2.0 |  | 407 | Biosynthetic |
| NODE_26.g1095.t1 | Putative importin subunit beta-4 | Importin-beta, N-terminal domain | 1.9 |  | 1098 | Transport |
| NODE_26.g1114.t1 | hypothetical protein BFJ65_g3552 | AMP-dependent synthetase/ligase | 6.3 |  | 1061 |  |
| NODE_260.g5879.t1 | hypothetical protein BFJ66_g7086 | Zinc finger C2H2-type | 1.7 |  | 531 | Transcription |
| NODE_261.g5891.t1 | Isocitrate dehydrogenase | Isocitrate dehydrogenase NADP-dependent | 2.6 |  | 462 | Oxidation-reduction |
| NODE_262.g5900.t1 | Phosphoribosylformylglycinamidine synthase | Phosphoribosylformylglycinamidine synthase, N-terminal | 1.7 |  | 1355 | Biosynthetic |
| NODE_27.g1127.t1 | Beta-glucosidase 1A | Glycoside hydrolase family 1 | 7.3 |  | 503 | Metabolic |
| NODE_27.g1129.t1 | hypothetical protein BFJ70_g4687 | Six-bladed beta-propeller, TolB-like | 4.6 |  | 633 |  |
| NODE_270.g6015.t1 | hypothetical protein FOC1_g10012848 | Zn (2)-C6 fungal-type DNA-binding domain superfamily | 2.7 |  | 1152 | Transcription |
| NODE_272.g6040.t1 | glycyl-tRNA synthetase | Glycyl-tRNA synthetase | 1.3 |  | 663 | Glycyl-tRNA aminoacylation |
| NODE_273.g6061.t1 | AGC/RSK/RSK-protein kinase | Protein kinase domain-AGC | 1.0 |  | 619 | Signal transduction |
| NODE_275.g6080.t1 | hypothetical protein FPSE_00523 | Ribosomal protein L13 | 3.0 |  | 202 | Translation |
| NODE_277.g6117.t1 | hypothetical protein FOXG_00622 | ATP synthase, F0 complex, subunit E, mitochondrial | 1.4 | Mitochondrion | 90 | Transport |
| NODE_277.g6122.t1 | Putative protein phosphatase 2C 80 | PPM-type phosphatase domain superfamily | 1.6 |  | 366 |  |
| NODE_279.g6151.t1 | probable heat shock protein 70 | Heat shock protein 70 family | 2.9 |  | 614 | Stress response |
| NODE_28.g1161.t1 | uncharacterized protein FFUJ_08611 | P-loop containing nucleoside triphosphate hydrolase | 3.6 |  | 303 | Phosphorylation |
| NODE_280.g6159.t1 | hypothetical protein BFJ70_g3149 |  | 5.1 |  | 599 |  |
| NODE_283.g6185.t1 | cytochrome heme mitochondrial | Cytochrome c1 | 1.6 |  | 322 | Oxidation-reduction |
| NODE_284.g6212.t1 | hypothetical protein FOCG_16049 | NADPH-dependent FMN reductase-like | 2.2 |  | 290 | Oxidation-reduction |
| NODE_288.g6252.t1 | hypothetical protein FOCG_11217 | Ankyrin repeat-containing domain superfamily | 6.1 |  | 1593 | Metabolic |
| NODE_290.g6289.t1 | hypothetical protein FOQG_04921 |  | 2.3 |  | 387 |  |
| NODE_294.g6342.t1 | cutinase | Cutinase | 7.3 |  | 234 |  |
| NODE_296.g6364.t1 | Inositol-3-phosphate synthase | Myo-inositol-1-phosphate synthase | 1.1 |  | 541 | Biosynthetic |
| NODE_299.g6406.t1 | hypothetical protein FOXG_12057 | Conserved hypothetical protein | 4.4 |  | 348 |  |
| NODE_3.g133.t1 | alpha-L-arabinofuranosidase II precursor | Glycoside hydrolase, family 43 | 3.9 | Extracellular | 323 | Metabolic |
| NODE_3.g163.t1 | hypothetical protein BFJ71_g12492 | FAD-binding domain | 4.3 |  | 415 |  |
| NODE_300.g6429.t1 | hypothetical protein BFJ65_g12933 | Major facilitator superfamily | 3.9 |  | 450 | Transport |
| NODE_302.g6440.t1 | MEAB protein | bZIP_YAP | 1.7 |  | 410 | Transcription |
| NODE_302.g6444.t1 | hypothetical protein BFJ69_g6142 | Stm1-like, N-terminal | 2.7 |  | 316 |  |
| NODE_303.g6449.t1 | hypothetical protein FPOA_00733 | Protein kinase domain-AGC-kinase | 1.6 |  | 1272 | Signal transduction |
| NODE_306.g6489.t1 | hypothetical protein FPSE_06440 | ATP synthase, F0 complex, subunit D, mitochondrial | 1.8 |  | 173 | Transport |
| NODE_306.g6490.t1 | probable nascent polypeptide-associated complex alpha chain | Nascent polypeptide-associated complex subunit alpha | 2.5 |  | 197 | Transport |
| NODE_311.g6558.t1 | hypothetical protein FOXG_05440 | U3 small nucleolar RNA-associated SSU processome protein 25 (Utp25) | 1.4 |  | 719 | rRNA processing |
| NODE_315.g6612.t1 | cystathionine beta-lyase | Cys/Met metabolism, pyridoxal phosphate-dependent enzyme | 1.4 |  | 428 | transsulfuration |
| NODE_318.g6664.t1 | hypothetical protein BFJ65_g5188 | Beta-lactamase-related | 4.6 |  | 751 |  |
| NODE_319.g6674.t1 | hypothetical protein FOC1_g10001023 |  | 2.1 |  | 425 |  |
| NODE_32.g1278.t1 | glycerol kinase | Carbohydrate kinase, FGGY, C-terminal | 1.9 |  | 516 | Metabolic |
| NODE_32.g1291.t1 | hypothetical protein FOXG_10013 |  | 2.6 |  | 370 |  |
| NODE_320.g6684.t1 | hypothetical protein FOCG_02613 | SH3 domain | 3.2 |  | 745 | Translation |
| NODE_321.g6694.t1 | hypothetical protein BFJ67_g12378 |  | 3.5 |  | 665 |  |
| NODE_321.g6698.t1 | related to aminopeptidase Y precursor, vacuolar | Peptidase M28, SGAP-like | 5.7 |  | 709 | Proteolysis |
| NODE_335.g6883.t1 | hypothetical protein BFJ65_g7571 | Ribosomal protein L10 | 1.1 |  | 333 | Translation |
| NODE_341.g6976.t1 | STE/STE20/PAKA protein kinase | Protein kinase domain | 1.8 |  | 863 | Signal transduction |
| NODE_341.g6979.t1 | hypothetical protein BFJ69_g4124 | Ubiquinol-cytochrome C reductase hinge domain superfamily | 2.0 |  | 135 | Oxidation-reduction |
| NODE_343.g6997.t1 | transporter sec23 | Zinc finger, Sec23/Sec24-type | 2.2 |  | 932 | Transcription |
| NODE_344.g7009.t1 | hypothetical protein BFJ68_g15668 | Glycoside hydrolase, family 32 | 4.5 |  | 674 | Metabolic |
| NODE_348.g7058.t1 | endoglucanase type F | Glycoside hydrolase family 10 domain | 9.0 |  | 384 | Metabolic |
| NODE_35.g1384.t1 | Proline-specific permease | Amino acid/polyamine transporter I | 1.3 |  | 536 | Transport |
| NODE_35.g1386.t1 | Metallocarboxypeptidase A | Peptidase M14, carboxypeptidase A | 6.0 |  | 413 | Proteolysis |
| NODE_350.g7094.t1 | putative 1-aminocyclopropane-1-carboxylate deaminase | 1-aminocyclopropane-1-carboxylate deaminase | 4.4 |  | 577 | Catabolic |
| NODE_352.g7127.t1 | hypothetical protein FOCG_15370 | BTB/POZ domain | 5.8 |  | 243 |  |
| NODE_355.g7162.t1 | Orotidine 5'-phosphate decarboxylase | Orotidine 5'-phosphate decarboxylase domain | 1.1 |  | 366 | Biosynthetic |
| NODE_359.g7213.t1 | hypothetical protein BFJ65_g3448 | Ribosomal protein L40e | 2.8 |  | 675 | Translation |
| NODE_359.g7214.t1 | hypothetical protein BFJ70_g12854 | Translation elongation factor, IF5A C-terminal | 1.9 |  | 164 | Translation |
| NODE_36.g1400.t1 | arginyl-tRNA synthetase | Arginyl tRNA synthetase N-terminal domain superfamily | 1.0 |  | 635 | Arginyl-tRNA aminoacylation |
| NODE_36.g1403.t1 | arsenical-resistance protein | Arsenical-resistance protein Acr3 | 2.3 |  | 383 | Transport |
| NODE_36.g1409.t1 | probable acetyl-CoA carboxylase | Acetyl-CoA carboxylase, central domain | 1.7 |  | 2284 | Biosynthetic |
| NODE_36.g1416.t1 | tRNA pseudouridine synthase 1 | Pseudouridine synthase, catalytic domain superfamily | 1.5 |  | 604 | RNA modification |
| NODE_362.g7241.t1 | hypothetical protein FPSE_06055 | Ribosomal protein S27a | 2.4 |  | 154 | Translation |
| NODE_364.g7273.t1 | isocitrate lyase | Isocitrate lyase | 3.5 | Nucleus | 160 | Metabolic |
| NODE_37.g1434.t1 | hypothetical protein BFJ69_g8756 | Linker histone H1/H5, domain H15 | 1.2 |  | 224 | Nucleosome assembly |
| NODE_370.g7336.t1 | putative pectate lyase C | Pectin lyase fold | 6.6 |  | 396 | Metabolism |
| NODE_370.g7338.t1 | Maltose permease MAL61 | Major facilitator, sugar transporter-like | 6.5 |  | 549 | Transport |
| NODE_371.g7351.t1 | alanine transaminase | Aminotransferase, class I/classII | 2.3 |  | 480 | Biosynthetic |
| NODE_373.g7376.t1 | Centromere/microtubule-binding protein cbf5 | Pseudouridine synthase, catalytic domain superfamily | 1.4 |  | 487 | RNA modification |
| NODE_376.g7409.t1 | uncharacterized protein FVRRES_05356 | Ribosomal protein S4e | 2.7 |  | 261 | Translation |
| NODE_376.g7411.t1 | fumarate hydratase, mitochondrial | Fumarate hydratase, class II | 1.3 |  | 529 | Metabolic |
| NODE_377.g7422.t1 | hypothetical protein FPSE_01306 | Ribosomal protein S23, eukaryotic/archaeal | 2.7 | Cytoplasm | 145 | Translation |
| NODE_38.g1450.t1 | ATP-citrate synthase subunit 2 | ATP-citrate synthase, citrate-binding domain | 1.2 |  | 489 | Metabolic |
| NODE_383.g7489.t1 | hypothetical protein FOMG_14709 | Glucose receptor Git3, N-terminal-G protein-coupled | 3.1 |  | 479 | Signal transduction |
| NODE_384.g7504.t1 | chromatin modification-like protein VID21 | Homeobox-like domain superfamily | 1.2 |  | 1404 |  |
| NODE_386.g7522.t1 | uncharacterized protein FMAN_02797 |  | 5.1 |  | 85 |  |
| NODE_388.g7545.t1 | asparaginyl-tRNA synthetase | Asparagine-tRNA ligase | 1.1 |  | 564 | Asparaginyl-tRNA aminoacylation |
| NODE_395.g7629.t1 | hypothetical protein BFJ69_g5063 | Transcription factor domain, fungi | 2.6 |  | 542 | Transcription |
| NODE_4.g196.t1 | hypothetical protein FOXG_04778 | Acetyltransferase (GNAT) domain | 3.8 |  | 228 | Biosynthetic |
| NODE_4.g221.t1 | hypothetical protein FOCG_03674 | Lipoxygenase, C-terminal | 6.9 |  | 744 | Oxidation-reduction |
| NODE_4.g222.t1 | catalase | Catalase core domain | 6.2 |  | 374 | Oxidation-reduction |
| NODE_4.g234.t1 | hypothetical protein BFJ71_g10841 | Peptidase M10, metallopeptidase | 9.9 |  | 266 | Proteolysis |
| NODE_4.g235.t1 | hypothetical protein FOCG_03694 |  | 8.0 |  | 353 |  |
| NODE_403.g7726.t1 | hypothetical protein FOCG_07260 | Conserved hypothetical protein | 6.8 |  | 368 |  |
| NODE_406.g7768.t1 | hypothetical protein FPSE_10374 | Ribosomal protein L5 | 2.7 |  | 173 | Translation |
| NODE_407.g7776.t1 | hypothetical protein FOXG_16638 | Aspartic peptidase A1 family | 1.5 |  | 413 | Proteolysis |
| NODE_407.g7784.t1 | Oligopeptide transporter 3 | Oligopeptide transporter, OPT superfamily | 7.8 |  | 784 | Transport |
| NODE_41.g1542.t1 | hypothetical protein FOC4_g10011786 |  | 2.2 |  | 809 |  |
| NODE_414.g7856.t1 | Uncharacterized protein LW93_7355 | Ribosomal protein S14 | 2.0 | Nucleus | 106 | Translation |
| NODE_414.g7857.t1 | probable translation elongation factor EF-Tu precursor, mitochondrial | GTP-binding domain | 1.4 |  | 445 | Signal transduction |
| NODE_416.g7879.t1 | cytochrome c | Cytochrome c-like domain | 1.9 |  | 106 | Oxidation-reduction |
| NODE_417.g7896.t1 | ATP synthase subunit mitochondrial | ATP synthase, F0 complex, subunit C | 2.6 |  | 146 | Transportation |
| NODE_421.g7934.t1 | hypothetical protein FPSE_00983 | Plectin/S10, N-terminal | 3.0 |  | 165 | Cytoskeleton organization |
| NODE_422.g7946.t1 | Isoleucyl-tRNA synthetase, cytoplasmic | Aminoacyl-tRNA synthetase, class Ia | 1.7 |  | 1071 | Translation |
| NODE_428.g8010.t1 | probable alpha-glucoside transport protein | Major facilitator, sugar transporter-like | 5.9 |  | 561 | Transport |
| NODE_429.g8017.t1 | malate synthase, glyoxysomal | Malate synthase | 1.1 |  | 542 | Glyoxylate cycle |
| NODE_43.g1592.t1 | hypothetical protein BFJ70_g9156 | Heterokaryon incompatibility | 1.7 |  | 775 |  |
| NODE_43.g1602.t1 | putative RNA-binding protein C17H9.04c | Zinc finger, RanBP2-type | 1.5 |  | 633 | Transcription |
| NODE_43.g1614.t1 | probable farnesyl-pyrophosphate synthetase | Polyprenyl synthetase | 1.3 |  | 347 | Biosynthetic |
| NODE_431.g8035.t1 | hypothetical protein FOMG_09129 |  | 6.9 |  | 391 |  |
| NODE_432.g8041.t1 | Methylenetetrahydrofolate dehydrogenase | Tetrahydrofolate dehydrogenase/cyclohydrolase, catalytic domain | 1.1 |  | 338 | Oxidation-reduction |
| NODE_434.g8073.t1 | hypothetical protein FOXG_13466 | S-adenosyl-L-methionine-dependent methyltransferase | 3.8 |  | 350 | Methylation |
| NODE_436.g8092.t1 | hypothetical protein FOXG_09247 | Mitochondrial ribosomal protein MRP51, fungi | 1.4 |  | 477 | Translation |
| NODE_436.g8093.t1 | hypothetical protein FAVG1_00109 | Ribosomal protein S17e | 3.0 |  | 147 | Translation |
| NODE_45.g1675.t1 | hypothetical protein FOMG_07095 | Nitronate monooxygenase | 2.3 |  | 364 | Oxidation-reduction |
| NODE_451.g8245.t1 | acid beta-fructofuranosidase precursor | Glycoside hydrolase, family 32 | 6.3 |  | 600 | Metabolic |
| NODE_458.g8310.t1 | hypothetical protein FOCG_09313 | Ferric reductase, NAD binding domain | 2.1 |  | 618 | Oxidation-reduction |
| NODE_46.g1682.t1 | hypothetical protein BFJ69_g13935 |  | 5.2 |  | 612 |  |
| NODE_46.g1695.t1 | hypothetical protein FOCG_16184 | Aspartic peptidase A1 family | 7.9 |  | 390 | Proteolysis |
| NODE_463.g8365.t1 | elongation factor G, mitochondrial | GTP-binding domain | 2.3 |  | 825 | Signal transduction |
| NODE_466.g8409.t1 | hypothetical protein FOCG_11808 |  | 6.9 | Extracellular | 154 |  |
| NODE_474.g8491.t1 | hypothetical protein BFJ66_g9621 |  | 1.4 |  | 108 |  |
| NODE_48.g1739.t1 | spermidine synthase | Spermidine/spermine synthases | 2.2 |  | 294 | Metabolic |
| NODE_48.g1742.t1 | hypothetical protein FPSE_03626 | Ribosomal protein L10e/L16 | 1.1 |  | 221 | Translation |
| NODE_48.g1752.t1 | Allergen Asp f 7 | RlpA-like domain superfamily | 1.9 |  | 292 |  |
| NODE_484.g8584.t1 | dihydrolipoamide acetyltransferase component pyruvate dehydrogenase complex | Peripheral subunit-binding domain | 1.9 |  | 457 |  |
| NODE_484.g8589.t1 | hypothetical protein FOMG_13818 | allergen asp | 2.1 |  | 316 |  |
| NODE_489.g8635.t1 | ubiquinol-cytochrome c reductase core subunit 2 | Metalloenzyme, LuxS/M16 peptidase-like | 1.5 |  | 454 | Proteolysis |
| NODE_493.g8684.t1 | Putative quinate permease | Major facilitator, sugar transporter-like | 2.6 |  | 505 | Transport |
| NODE_497.g8717.t1 | related to carboxylic acid transport protein JEN1 | Major facilitator, sugar transporter-like | 7.1 |  | 516 | Transport |
| NODE_5.g287.t1 | hypothetical protein BFJ71_g8483 | Tetratricopeptide repeat | 2.1 |  | 719 | RNA processing |
| NODE_50.g1801.t1 | hypothetical protein BFJ69_g9020 | Ribosomal protein L24e-related | 2.6 |  | 160 | Translation |
| NODE_503.g8768.t1 | hypothetical protein FGSG_05999 | Ribosomal protein L27e | 2.8 |  | 135 | Translation |
| NODE_503.g8777.t1 | Leucine--tRNA ligase, cytoplasmic | Aminoacyl-tRNA synthetase, class Ia | 3.3 |  | 669 | Translation |
| NODE_508.g8824.t1 | hypothetical protein FOCG_09634 | P-loop containing nucleoside triphosphate hydrolase | 1.1 |  | 2209 | Phosphorylation |
| NODE_509.g8836.t1 | hypothetical protein FOXG_01756 | Zinc finger C2H2-type | 1.6 |  | 348 | Transcription |
| NODE_51.g1818.t1 | 2,3-dihydroxybenzoate decarboxylase | Amidohydrolase-related | 7.8 |  | 336 | Proteolysis |
| NODE_51.g1820.t1 | related to salicylate 1-monooxygenase | FAD-binding domain | 7.9 |  | 452 |  |
| NODE_512.g8855.t1 | tyrosine 3-monooxygenase/tryptophan 5-monooxygenase activation protein | 14-3-3 domain | 2.0 |  | 145 |  |
| NODE_512.g8856.t1 | 14-3-3 protein | 14-3-3 domain | 1.8 |  | 141 |  |
| NODE_512.g8865.t1 | Threonine--tRNA ligase, cytoplasmic | Threonine-tRNA ligase, class IIa | 1.3 |  | 745 | Threonyl-tRNA aminoacylation |
| NODE_519.g8920.t1 | hypothetical protein CDV36_005045, partial | GPI anchored serine-rich protein | 1.7 |  | 100 | Proteolysis |
| NODE_525.g8964.t1 | hypothetical protein FOXG_04924 | Only prolin and serin are matching in the corresponding protein | 2.1 |  | 640 |  |
| NODE_526.g8974.t1 | hypothetical protein BFJ69_g1507 | Polyketide synthase, enoylreductase domain | 2.3 |  | 360 | Biosynthetic |
| NODE_529.g9001.t1 | Carbamoyl-phosphate synthase arginine-specific large chain | Carbamoyl-phosphate synthetase large subunit-like, ATP-binding domain | 1.5 |  | 1175 | Metabolic |
| NODE_531.g9023.t1 | hypothetical protein BFJ65_g833 | PTP type protein phosphatase | 2.0 |  | 524 | Protein dephosphorylation |
| NODE_532.g9027.t1 | phosphoenolpyruvate carboxykinase | Phosphoenolpyruvate carboxykinase, ATP-utilising | 2.9 |  | 589 | Metabolic |
| NODE_538.g9084.t1 | hypothetical protein FOXG_08208 |  | 3.6 |  | 209 |  |
| NODE_54.g1909.t1 | Putative branched-chain-amino-acid aminotransferase TOXF | Branched-chain amino acid aminotransferase II | 1.7 |  | 380 | Metabolic |
| NODE_54.g1911.t1 | hypothetical protein FOXG_08701 | WD domain, G-beta repeat | 1.3 |  | 515 | Signal transduction |
| NODE_540.g9103.t1 | hypothetical protein FOC1_g10003508 | integral membrane protein | 3.8 |  | 397 |  |
| NODE_544.g9152.t1 | probable hnRNP arginine N-methyltransferase | arginine N-methyltransferase | 1.9 |  | 345 | Methylation |
| NODE_549.g9201.t1 | GTPase | GTP binding domain | 1.6 |  | 394 | Signal transduction |
| NODE_556.g9273.t1 | endo-1,4-beta-xylanase C | Glycoside hydrolase family 10 domain | 6.4 | Extracellular | 328 | Metabolic |
| NODE_56.g1959.t1 | ornithine decarboxylase | Orn/DAP/Arg decarboxylase 2, C-terminal | 1.1 |  | 448 |  |
| NODE_561.g9307.t1 | hypothetical protein FOCG_04186 | tRNA/rRNA methyltransferase, SpoU type | 1.6 |  | 692 | RNA processing |
| NODE_566.g9354.t1 | hypothetical protein BFJ65_g17320 | Carbohydrate-binding, CenC-like | 3.1 |  | 902 | Metabolic |
| NODE_581.g9489.t1 | hypothetical protein FOIG_01343 | Ribosomal protein L15e | 2.5 |  | 450 | Translation |
| NODE_592.g9595.t1 | hypothetical protein FOCG_01584 | Multicopper oxidase, type 2 | 6.1 |  | 633 | Oxidation-reduction |
| NODE_592.g9596.t1 | hypothetical protein BFJ71_g12101 | Glycoside hydrolase family 31 | 8.4 |  | 1063 | Metabolic |
| NODE_596.g9634.t1 | Glutamate--cysteine ligase | Glutamate-cysteine ligase catalytic subunit | 1.3 |  | 716 | Biosynthetic |
| NODE_6.g315.t1 | translation initiation factor RLI1 | ABC transporter-like | 1.5 |  | 607 | Transport |
| NODE_6.g316.t1 | Chromodomain helicase hrp3 | SNF2-related, N-terminal domain | 1.5 |  | 1626 |  |
| NODE_60.g2063.t1 | hypothetical protein FOC1_g10002730 |  | 6.4 |  | 1079 |  |
| NODE_60.g2067.t1 | hypothetical protein BFJ65_g11036 | ATPase, AAA-type | 4.7 |  | 1429 |  |
| NODE_600.g9660.t1 | hypothetical protein BFJ71_g13304 | bZIP_ATF2 | 1.7 |  | 525 | Transcription |
| NODE_607.g9721.t1 | hypothetical protein BFJ68_g1924 |  | 2.5 |  | 449 |  |
| NODE_607.g9726.t1 | 54S ribosomal protein L3, mitochondrial | Ribonuclease III, endonuclease domain superfamily | 1.2 |  | 383 | RNA processing |
| NODE_611.g9752.t1 | hypothetical protein BFJ70_g4924 | Protein kinase domain | 4.4 |  | 1223 | Signal transduction |
| NODE_611.g9753.t1 | hypothetical protein FOMG_13400 | Prion-inhibition and propagation, HeLo domain | 4.2 |  | 573 |  |
| NODE_614.g9784.t1 | Unsaturated rhamnogalacturonyl hydrolase yteR | Glycosyl hydrolase, family 88 | 6.7 |  | 381 | Metabolic |
| NODE_617.g9818.t1 | putative exopolygalacturonase X | Glycoside hydrolase, family 28 | 6.3 |  | 450 | Metabolic |
| NODE_618.g9822.t1 | hypothetical protein BFJ65_g1649 |  | 1.2 |  | 1130 |  |
| NODE_619.g9829.t1 | T-complex protein 1 subunit epsilon | Chaperonin TCP-1, conserved site | 1.3 |  | 546 | Stress response |
| NODE_62.g2116.t1 | L-lactate dehydrogenase (cytochrome) | Cytochrome b5-like heme/steroid binding domain | 2.4 |  | 502 | Oxidation-reduction |
| NODE_623.g9857.t1 | hypothetical protein FOXG_05878 |  | 7.5 | Extracellular | 381 |  |
| NODE_624.g9869.t1 | 60S ribosomal protein L7 | Ribosomal protein L7, eukaryotic | 2.0 |  | 248 | Translation |
| NODE_630.g9918.t1 | CAMK/CAMKL/KIN4 protein kinase | Protein kinase domain-CAMK/CAMKL/KIN4 protein kinase | 1.6 |  | 1102 | Signal transduction |
| NODE_635.g9955.t1 | hypothetical protein FOQG_05573 | Phytocyanin domain | 3.4 |  | 215 | Transport |
| NODE_635.g9957.t1 | Multisite-specific tRNA:(cytosine-C (5))-methyltransferase | tRNA (C5-cytosine) methyltransferase, NCL1 | 1.3 |  | 845 | tRNA methylation |
| NODE_635.g9960.t1 | hypothetical protein BFJ69_g14912 | Nuclear segregation protein | 1.2 |  | 474 | Nuclear segregation protein |
| NODE_636.g9970.t1 | hypothetical protein FOCG_12257 | Uncharacterised domain NUC173 | 1.3 |  | 1240 |  |
| NODE_64.g2161.t1 | probable succinyl-CoA 3-ketoacid-coenzyme A transferase, mitochondrial precursor | Coenzyme A transferase family I | 3.4 |  | 1092 | Catabolic |
| NODE_644.g10030.t1 | hypothetical protein FOMG_08661 | cellulose-binding-like domain superfamily (Expansin) | 2.1 |  | 319 | Metabolic |
| NODE_648.g10055.t1 | hypothetical protein BFJ71_g16269 | Clr5 domain | 1.4 |  | 887 |  |
| NODE_650.g10075.t1 | ADP, ATP carrier protein | Mitochondrial carrier protein | 2.6 |  | 312 | Transport |
| NODE_651.g10085.t1 | hypothetical protein BFJ65_g17894 | NACHT nucleoside triphosphatase | 3.8 |  | 1184 | Metabolic |
| NODE_653.g10102.t1 | hypothetical protein FOCG_09092 | Protein Sls1 | 1.9 |  | 1265 | Translation |
| NODE_657.g10132.t1 | hypothetical protein FOXG_13238 | Conserved hypothetical protein | 6.7 |  | 340 |  |
| NODE_66.g2230.t1 | hypothetical protein FOCG_09412 | PH-like domain superfamily | 2.5 |  | 484 |  |
| NODE_660.g10163.t1 | hypothetical protein FOCG_11843 |  | 7.9 |  | 354 |  |
| NODE_660.g10164.t1 | hypothetical protein FOCG_11842 |  | 9.5 | Cytoplasm | 135 |  |
| NODE_661.g10166.t1 | glycogen | Glycogen synthase | 1.2 |  | 705 | Biosynthetic |
| NODE_664.g10193.t1 | aspartate aminotransferase, mitochondrial | Aspartate/ other aminotransferase | 2.5 |  | 424 | Metabolic |
| NODE_67.g2260.t1 | hypothetical protein BFJ70_g902 | Galactose-binding-like domain superfamily | 6.2 |  | 555 |  |
| NODE_671.g10244.t1 | phosphoribosylglycinamide formyltransferase | Formyl transferase, N-terminal | 2.3 |  | 225 | Biosynthetic |
| NODE_671.g10245.t1 | hypothetical protein BFJ68_g8916 | FAD dependent oxidoreductase | 1.2 |  | 473 | Oxidation-reduction |
| NODE_674.g10273.t1 | Zinc finger protein zpr1 | Zinc finger, ZPR1-type | 1.1 |  | 483 | Transcription |
| NODE_68.g2279.t1 | hypothetical protein BFJ65_g12542 |  | 6.7 |  | 678 |  |
| NODE_68.g2280.t1 | hypothetical protein BFJ65_g13029 |  | 8.4 |  | 2041 |  |
| NODE_68.g2281.t1 | hypothetical protein FOQG_10526 |  | 5.1 |  | 1046 |  |
| NODE_68.g2282.t1 | hypothetical protein BFJ71_g6375 |  | 5.6 |  | 1460 |  |
| NODE_68.g2285.t1 | hypothetical protein FLONG3_3149 | Peptidase S10, serine carboxypeptidase | 1.2 |  | 811 | Proteolysis |
| NODE_681.g10326.t1 | hypothetical protein BFJ71_g9053 | Polyketide synthase, enoylreductase domain | 4.3 |  | 818 | Biosynthetic |
| NODE_684.g10349.t1 | nucleolar GTP-binding protein | Nucleolar GTP-binding protein 1, Rossman-fold domain | 1.2 |  | 659 | Signal transduction |
| NODE_684.g10350.t1 | orotate phosphoribosyltransferase | Phosphoribosyltransferase domain | 1.8 |  | 234 | Metabolic |
| NODE_687.g10368.t1 | hypothetical protein FOMG_11258 | Cyclin PHO80-like | 1.1 |  | 690 | Signal transduction |
| NODE_688.g10378.t1 | choline dehydrogenase | Glucose-methanol-choline oxidoreductase, N-terminal | 4.6 |  | 673 | Oxidation-reduction |
| NODE_699.g10465.t1 | Protein BCP1 | BCP1 family | 1.3 |  | 285 |  |
| NODE_7.g350.t1 | hypothetical protein BFJ66_g8830 | Pyridine nucleotide-disulphide oxidoreductase | 2.1 |  | 456 | Oxidation-reduction |
| NODE_700.g10468.t1 | hypothetical protein FOQG_03281 |  | 4.0 |  | 433 |  |
| NODE_700.g10471.t1 | L-arabinitol 4-dehydrogenase | Alcohol dehydrogenase, C-terminal | 3.0 |  | 375 | Oxidation-reduction |
| NODE_702.g10486.t1 | hypothetical protein FOC1_g10005957 | Carbohydrate-binding domain, family 9 | 6.4 | Extracellular | 221 | Metabolic |
| NODE_702.g10487.t1 | probable DUF895 domain membrane protein | Major facilitator superfamily | 5.1 |  | 503 | Transport |
| NODE_702.g10488.t1 | hypothetical protein BFJ71_g10640 | Peptidase M20, dimerisation domain | 4.8 |  | 961 | Proteolysis |
| NODE_704.g10501.t1 | mitochondrial import receptor subunit tom-40 | Porin3_Tom40 | 1.1 |  | 356 | Transport |
| NODE_706.g10510.t1 | hypothetical protein BFJ69_g14025 | Dual specificity protein phosphatase domain | 1.4 |  | 1059 | Protein dephosphorylation |
| NODE_708.g10526.t1 | related to cellobiose dehydrogenase | Glucose-methanol-choline oxidoreductase, N-terminal | 1.6 |  | 543 | Oxidation-reduction |
| NODE_708.g10527.t1 | betaine-aldehyde dehydrogenase | Aldehyde dehydrogenase domain | 1.8 |  | 509 | Oxidation-reduction |
| NODE_71.g2345.t1 | Transcription factor steA | zinc fingers C2H2 - Ste12 | 1.2 |  | 689 | Transcription |
| NODE_72.g2355.t1 | hypothetical protein BFJ69_g1208 | Acyl-CoA N-acyltransferase | 5.2 |  | 286 | Biosynthetic |
| NODE_726.g10654.t1 | hypothetical protein BFJ70_g11612 | Glucose-methanol-choline oxidoreductase, N-terminal | 5.3 |  | 640 | Oxidation-reduction |
| NODE_728.g10666.t1 | guanine nucleotide-binding protein subunit beta | WD domain, G-beta repeat | 1.0 |  | 359 | Signal transduction |
| NODE_73.g2378.t1 | Protein gar2 | RNA recognition motif domain | 1.4 |  | 505 | Cytokinesis |
| NODE_73.g2389.t1 | probable EFB1-translation elongation factor eEF1beta | Translation elongation factor EF1B, beta/delta subunit, guanine nucleotide exchange domain | 1.8 |  | 231 | Translation |
| NODE_735.g10719.t1 | Adenylosuccinate lyase | Adenylosuccinate lyase C-terminal | 1.9 |  | 277 | Biosynthetic |
| NODE_74.g2420.t1 | Hexokinase-1 | Hexokinase, N-terminal | 2.3 |  | 549 | Metabolic |
| NODE_74.g2421.t1 | Glucosamine-6-phosphate isomerase 1 | Glucosamine-6-phosphate isomerase | 2.7 |  | 383 | Metabolic |
| NODE_740.g10767.t1 | Malate dehydrogenase, cytoplasmic | Lactate dehydrogenase/glycoside hydrolase, family 4, C-terminal | 2.6 |  | 335 | Oxidation-reduction |
| NODE_741.g10772.t1 | related to eukaryotic translation initiation factor 3 subunit 11 | Translation initiation factor 3, subunit 12, N-terminal, eukaryotic | 1.5 |  | 231 | Translation |
| NODE_742.g10788.t1 | hypothetical protein BFJ65_g17190 |  | 6.6 |  | 299 |  |
| NODE_743.g10792.t1 | hypothetical protein BFJ70_g12646 | Galactose-binding-like domain superfamily | 3.6 | Extracellular | 223 |  |
| NODE_745.g10813.t1 | hypothetical protein BFJ71_g9543 | Galactose-binding-like domain superfamily | 5.3 |  | 682 |  |
| NODE_750.g10848.t1 | hypothetical protein FOC4_g10007865 |  | 1.5 |  | 574 |  |
| NODE_750.g10851.t1 | hypothetical protein FOC4_g10007868 | BTB/POZ domain | 2.4 |  | 289 |  |
| NODE_758.g10897.t1 | probable translation elongation factor eEF-3 | ABC transporter-like | 2.0 |  | 1055 | Transport |
| NODE_762.g10919.t1 | 6-hydroxy-D-nicotine oxidase | FAD linked oxidase, N-terminal | 6.0 |  | 654 | Oxidation-reduction |
| NODE_770.g10974.t1 | PiT family inorganic phosphate transporter | Phosphate transporter | 9.3 |  | 607 | Transport |
| NODE_775.g11010.t1 | hypothetical protein BFJ65_g4569 | Eisosome protein 1 | 2.7 |  | 726 |  |
| NODE_779.g11040.t1 | hypothetical protein FPSE_03899 | Ribosomal protein L7Ae conserved site | 1.6 | Cytoplasm | 124 | Translation |
| NODE_78.g2489.t1 | hypothetical protein BFJ69_g7177 | Major facilitator superfamily | 4.8 |  | 499 | Transport |
| NODE_787.g11098.t1 | hypothetical protein FAVG1_05598 | Ribosomal protein L1 | 2.9 | Cytoplasm | 217 | Translation |
| NODE_789.g11111.t1 | Sulfite reductase | Nitrite/sulphite reductase 4Fe-4S domain | 1.7 |  | 918 | Oxidation-reduction |
| NODE_79.g2533.t1 | hypothetical protein BFJ71_g12165 |  | 5.6 |  | 260 |  |
| NODE_798.g11165.t1 | hypothetical protein FOCG_08451 | Major facilitator, sugar transporter-like | 5.1 |  | 507 | Transport |
| NODE_799.g11175.t1 | hypothetical protein BFJ69_g3127 | Transcription factor domain, fungi | 1.3 |  | 971 | Transcription |
| NODE_8.g373.t1 | endo-1,3(4)-beta-glucanase | Glycoside hydrolase family 16 | 6.6 |  | 286 | Metabolic |
| NODE_801.g11185.t1 | hypothetical protein FOCG_11444 | Cation/H+ exchanger | 3.2 |  | 851 | Transport |
| NODE_803.g11197.t1 | MICOS complex subunit MIC60 | Mitochondrial inner membrane protein Mitofilin | 1.4 |  | 636 |  |
| NODE_804.g11207.t1 | hypothetical protein BFJ71_g14823 | RGS domain superfamily | 1.7 |  | 322 | Signal transduction |
| NODE_807.g11226.t1 | Efflux pump roqT | Major facilitator superfamily | 1.7 |  | 581 | Transport |
| NODE_81.g2581.t1 | hypothetical protein FOCG_16099 | Major facilitator, sugar transporter-like | 7.3 |  | 499 | Transport |
| NODE_812.g11266.t1 | Glycine-rich RNA-binding protein 2, mitochondrial | RNA recognition motif domain | 2.8 |  | 114 | Cytokinesis |
| NODE_815.g11287.t1 | Bifunctional xylanase/deacetylase | NodB homology domain | 6.1 | Extracellular | 274 | Metabolic |
| NODE_82.g2611.t1 | hypothetical protein BFJ70_g1198 | Rossmann-like alpha/beta/alpha sandwich fold | 7.9 |  | 288 |  |
| NODE_823.g11344.t1 | Threonine synthase | Threonine synthase, N-terminal | 1.4 |  | 539 | Biosynthetic |
| NODE_825.g11355.t1 | hypothetical protein FOQG_13617 | Peptidase S8/S53 domain superfamily | 5.7 |  | 599 | Proteolysis |
| NODE_836.g11436.t1 | hypothetical protein FOMG_08164 | Sas10/Utp3/C1D | 1.4 |  | 616 |  |
| NODE_838.g11446.t1 | hypothetical protein FOXG_01776 | SET domain | 1.4 |  | 573 |  |
| NODE_838.g11449.t1 | hypothetical protein FOCG_00981 |  | 1.5 |  | 651 |  |
| NODE_847.g11520.t1 | hypothetical protein BFJ65_g15900 |  | 3.7 |  | 382 |  |
| NODE_847.g11525.t1 | hypothetical protein BFJ71_g12493 | Alpha/beta hydrolase fold 3 | 5.1 |  | 343 | Metabolic |
| NODE_852.g11557.t1 | Peroxisomal primary amine oxidase | Copper amine oxidase, N3-terminal | 5.6 |  | 702 | Oxidation-reduction |
| NODE_859.g11593.t1 | hypothetical protein FOC4_g10010274 | MICOS complex subunit MIC26/MIC27 | 1.3 |  | 249 |  |
| NODE_86.g2687.t1 | hypothetical protein FOCG_05557 |  | 3.6 |  | 480 |  |
| NODE_86.g2691.t1 | Argininosuccinate synthase | Argininosuccinate synthase | 3.2 |  | 422 | Biosynthetic |
| NODE_86.g2693.t1 | hypothetical protein BFJ68_g8568 | Pectinesterase, catalytic | 8.9 |  | 2638 | Metabolic |
| NODE_866.g11633.t1 | hypothetical protein BFJ69_g14759 |  | 5.3 |  | 523 |  |
| NODE_866.g11634.t1 | hypothetical protein FOIG_11146 | Rhodanese-like domain superfamily | 3.1 |  | 195 |  |
| NODE_877.g11700.t1 | MFS transporter, SP family, general alpha glucoside:H+ symporter | Major facilitator, sugar transporter-like | 7.5 |  | 541 | Transport |
| NODE_884.g11738.t1 | Pescadillo like protein | Pescadillo | 1.1 |  | 659 | Ribosome biogenesis |
| NODE_887.g11755.t1 | hypothetical protein BFJ67_g9277 | Cytochrome P450 superfamily | 2.9 |  | 702 | Oxidation-reduction |
| NODE_888.g11762.t1 | hypothetical protein FOIG_11752 | Major facilitator, sugar transporter-like | 5.1 |  | 556 | Transport |
| NODE_890.g11774.t1 | probable GAP1-General amino acid permease | Amino acid/polyamine transporter I | 2.9 |  | 536 | Transport |
| NODE_897.g11819.t1 | cytochrome b-c1 complex subunit Rieske, mitochondrial | Cytochrome b-c1 complex subunit Rieske, transmembrane domain | 1.0 | Mitochondrion | 235 | Oxidation-reduction |
| NODE_906.g11870.t1 | uncharacterized protein FPRN_04559 | Nucleic acid-binding, OB-fold | 4.0 |  | 58 |  |
| NODE_93.g2856.t1 | Aconitate hydratase | Aconitase, mitochondrial-like | 2.0 |  | 785 | Metabolic |
| NODE_930.g12005.t1 | Protein PRY1 | Allergen V5/Tpx-1-related, conserved site | 1.8 |  | 336 |  |
| NODE_937.g12042.t1 | hypothetical protein FOXG_08113 | Heat shock factor (HSF)-type, DNA-binding | 1.0 |  | 586 | Transcription |
| NODE_938.g12043.t1 | hypothetical protein FOC4_g10012609 | Amine oxidase | 2.7 |  | 519 | Oxidation-reduction |
| NODE_948.g12098.t1 | hypothetical protein BFJ68_g10016 | Protein kinase domain | 6.9 |  | 841 | Signal transduction |
| NODE_953.g12125.t1 | hypothetical protein BFJ70_g17240 | Cytochrome P450 | 3.2 |  | 523 | Oxidation-reduction |
| NODE_955.g12133.t1 | hypothetical protein BFJ65_g5397 | Proline rich extensin signature | 1.2 |  | 911 |  |
| NODE_956.g12140.t1 | Ankyrin repeat domain-containing protein 50 | Ankyrin repeat-containing domain superfamily | 2.2 |  | 1045 | Metabolic |
| NODE_959.g12152.t1 | hypothetical protein FOMG_09609 | Patatin-like phospholipase domain | 4.3 |  | 567 | Metabolic |
| NODE_963.g12174.t1 | hypothetical protein FOCG_08283 | Major facilitator, sugar transporter-like | 8.1 |  | 571 | Transport |
| NODE_964.g12178.t1 | Alternative oxidase, mitochondrial | Alternative oxidase | 2.2 |  | 353 | Oxidation-reduction |
| NODE_965.g12180.t1 | 60s ribosomal | Ribosomal protein L26/L24, eukaryotic/archaeal | 1.8 |  | 136 | Translation |
| NODE_969.g12201.t1 | probable GMP synthase | GMP synthase, glutamine amidotransferase | 1.8 |  | 544 | Biosynthetic |
| NODE_98.g2979.t1 | hypothetical protein BFJ71_g11224 | Transcription factor domain, fungi | 6.9 |  | 352 | Transcription |
| NODE_980.g12272.t1 | probable elongation factor 2 | Translation elongation factor EFG/EF2, domain IV | 2.0 |  | 844 | Translation |
| NODE_981.g12277.t1 | hypothetical protein FOMG_09287 | Major facilitator superfamily | 3.9 |  | 490 | Transport |
| NODE_997.g12366.t1 | uncharacterized protein LW93_15019 |  | 3.6 | Extracellular | 128 |  |
| DN0_c0_g1_i4.g10971.t1 | hypothetical protein FPSE_03202 | Ribosomal protein S14 | 2.5 | Cytoplasm | 56 | Translation |
| DN10437_c0_g1_i2.g7264.t1 | U3 small nucleolar RNA-associated protein 10 | HEAT repeat | 4.4 |  | 579 |  |
| DN10465_c0_g1_i1.g7257.t1 | Pectin lyase | Pectate lyase | 7.3 | Extracellular | 130 | Metabolic |
| DN10501_c0_g1_i1.g10711.t1 | hypothetical protein FOC4_g10006151 | Major facilitator superfamily | 5.6 |  | 373 | Transport |
| DN10631_c0_g1_i2.g19147.t1 | hypothetical protein FOC1_g10013697 | U3 snoRNA associated | 7.8 |  | 274 | rRNA processing |
| DN10666_c0_g1_i1.g19212.t1 | hypothetical protein FOXG_08912 | Amino acid permease/ SLC12A domain | 4.3 |  | 540 | Transport |
| DN10692_c0_g1_i1.g19136.t1 | hypothetical protein BFJ72_g1607 | Major facilitator, sugar transporter-like | 7.3 |  | 236 | Transport |
| DN10699_c0_g1_i1.g19153.t1 | hypothetical protein FOC4_g10012713 | Glycoside hydrolase, family 61 | 5.2 |  | 312 | Metabolic |
| DN10708_c0_g1_i1.g14326.t1 | hypothetical protein FOC1_g10002515 | Chaperone J-domain superfamily | 1.5 |  | 441 | Stress response |
| DN10792_c0_g1_i1.g14344.t1 | chitinase | Glycoside hydrolase family 18, catalytic domain | 4.2 |  | 432 | Metabolic |
| DN10900_c0_g1_i1.g13836.t1 | hypothetical protein FOXG_06328 |  | 5.7 |  | 257 |  |
| DN10926_c0_g1_i1.g13827.t1 | hypothetical protein BFJ65_g7877 | Glycoside hydrolase, family 61 | 7.4 |  | 178 | Metabolic |
| DN1099_c0_g1_i3.g12518.t1 | cellular nucleic acid-binding protein | Zinc finger, CCHC-type | 3.0 | Cytoplasm | 178 | Transcription |
| DN11040_c0_g1_i1.g26750.t1 | hypothetical protein FOC4_g10006219 |  | 4.0 | Extracellular | 220 |  |
| DN11052_c0_g1_i1.g26728.t1 | hypothetical protein BFJ69_g8711 |  | 3.7 |  | 213 |  |
| DN11170_c0_g1_i1.g23876.t1 | hypothetical protein FOXG_20813 |  | 3.3 |  | 74 |  |
| DN1138_c0_g1_i1.g23442.t1 | hypothetical protein BFJ65_g8669 | Ubiquinol-cytochrome C reductase hinge domain superfamily | 1.5 |  | 100 | Oxidation-reduction |
| DN11486_c0_g1_i1.g22387.t1 | hypothetical protein BFJ71_g5446 |  | 4.2 |  | 297 |  |
| DN1155_c0_g1_i1.g23823.t1 | hypothetical protein BFJ68_g4198 | Cellulose/chitin-binding protein, N-terminal | 4.4 |  | 404 | Metabolic |
| DN11782_c0_g1_i1.g18427.t1 | Phosphate-repressible phosphate permease | Sodium-phosphate symporter which plays a fundamental housekeeping role in phosphate transport | 7.3 |  | 122 | Transport |
| DN11802_c0_g1_i1.g23021.t1 | hypothetical protein BFJ68_g6908 | Glycoside hydrolase, family 61 | 6.5 | Extracellular | 86 | Metabolic |
| DN1193_c0_g1_i4.g23432.t1 | Nucleolar protein 56 | NOP5, N-terminal | 1.2 |  | 504 | Ribosome biogenesis |
| DN12_c0_g1_i1.g11115.t1 | hypothetical protein BFJ69_g6089 |  | 2.0 |  | 373 |  |
| DN12_c0_g2_i1.g11116.t1 | hypothetical protein BFJ71_g675 | CFEM domain | 2.5 |  | 937 |  |
| DN12051_c0_g1_i1.g25002.t1 | hypothetical protein BFJ68_g10659 | Protein kinase domain | 1.6 |  | 676 | Signal transduction |
| DN12100_c0_g1_i1.g25011.t1 | hypothetical protein FOXG_02375 | Acetyl-CoA carboxylase, central domain | 1.6 |  | 1648 | Biosynthetic |
| DN12134_c0_g1_i1.g7895.t1 | hypothetical protein FVEG_11508 |  | 4.1 |  | 154 |  |
| DN12257_c0_g1_i1.g5555.t1 | 2-methylcitrate synthase | Citrate synthase | 1.6 |  | 465 | Metabolic |
| DN1247_c0_g1_i1.g7325.t1 | hypothetical protein BFJ65_g6222 |  | 1.2 |  | 432 |  |
| DN12484_c0_g1_i1.g23049.t1 | hypothetical protein FOCG_00758 |  | 2.5 |  | 489 |  |
| DN12591_c0_g1_i1.g16960.t1 | hypothetical protein FOC4_g10003626 | Acyl-CoA N-acyltransferase | 4.7 | Mitochondrion | 174 | Biosynthetic |
| DN12672_c0_g1_i1.g17683.t1 | Bifunctional protein RIB2 | Pseudouridine synthase, RsuA/RluA | 1.2 |  | 514 | RNA modification |
| DN12793_c0_g1_i1.g11784.t1 | L-fuculose-phosphate aldolase | Class II aldolase/adducin N-terminal | 4.5 |  | 221 | Metabolic |
| DN12928_c0_g1_i1.g17060.t1 | pectate lyase E | Pectate lyase PlyH/PlyE-like | 6.6 | Extracellular | 205 | Metabolic |
| DN13145_c0_g1_i1.g21053.t1 | hypothetical protein FOC4_g10008998 | PAN/Apple domain | 6.1 |  | 181 |  |
| DN13196_c0_g1_i1.g21046.t1 | family inorganic phosphate transporter | Phosphate transporter | 8.0 |  | 75 | Transport |
| DN1320_c0_g1_i3.g3003.t1 | hypothetical protein FOC1_g10013863 | Conserved hypothetical protein | 4.4 |  | 349 |  |
| DN13278_c0_g1_i1.g25277.t1 | hypothetical protein BFJ71_g12436 |  | 3.7 |  | 242 |  |
| DN13288_c0_g1_i1.g25268.t1 | hypothetical protein BFJ65_g16484 | Pyridine nucleotide-disulphide oxidoreductase | 3.4 | Plastid | 105 | Oxidation-reduction |
| DN13425_c0_g1_i1.g15307.t1 | galacturan 1,4-alpha-galacturonidase | Glycoside hydrolase, family 28 | 5.7 |  | 446 | Metabolic |
| DN1361_c0_g1_i4.g3045.t1 | related to protein phosphatases | Tetratricopeptide repeat | 4.4 |  | 362 | RNA processing |
| DN13836_c0_g1_i1.g63667.t1 | hypothetical protein BFJ66_g3979 | GLEYA adhesin domain | 4.0 |  | 309 |  |
| DN1392_c0_g1_i11.g2927.t1 | Putative RING finger protein C6B12.07c | Zinc finger, RING/FYVE/PHD-type | 6.6 |  | 505 | Transcription |
| DN13944_c0_g1_i1.g24964.t1 | hypothetical protein BFJ71_g3565 |  | 4.3 |  | 151 |  |
| DN14008_c0_g1_i1.g9692.t1 | Putative phosphoketolase | Xylulose 5-phosphate/Fructose 6-phosphate phosphoketolase, N-terminal | 4.6 | Extracellular | 127 | Metabolic |
| DN1442_c0_g1_i1.g15838.t1 | Retrovirus-related Pol polyprotein from transposon TNT 1-94 | Integrase, catalytic core | 5.7 |  | 1033 | DNA integration |
| DN14440_c0_g1_i1.g6409.t1 | probable pectate lyase 1 | Pectate lyase | 3.3 | Extracellular | 327 | Metabolic |
| DN1449_c0_g1_i1.g15938.t1 | 40S ribosomal protein S9, mitochondrial | Ribosomal protein S9 | 1.0 |  | 314 | Translation |
| DN1451_c0_g1_i4.g15728.t1 | Calcium-binding mitochondrial carrier protein Aralar1 | Mitochondrial substrate/solute carrier | 3.3 |  | 695 | Transport |
| DN146_c0_g1_i21.g9321.t1 | hypothetical protein FGSG_09933 | Eukaryotic porin/Tom40 | 1.6 |  | 283 | Transport |
| DN14619_c0_g1_i1.g21092.t1 | hypothetical protein FOXG_10949 |  | 11.8 | Extracellular | 84 |  |
| DN1463_c0_g1_i1.g15494.t1 | hypothetical protein BFJ71_g8322 |  | 2.3 |  | 255 |  |
| DN14707_c0_g1_i1.g14134.t1 | hypothetical protein BFJ70_g13205 | Conserved proline-rich protein | 4.7 |  | 597 |  |
| DN1474_c0_g1_i4.g15583.t1 | putative inorganic pyrophosphatase | Inorganic pyrophosphatase | 1.8 |  | 396 | Metabolic |
| DN14756_c0_g1_i1.g14118.t1 | Pectinesterase | Pectinesterase, catalytic | 5.3 | Extracellular | 96 | Metabolic |
| DN14818_c0_g1_i1.g1803.t1 | probable GFA1-glucosamine--fructose-6-phosphate transaminase | Sugar isomerase (SIS) | 1.1 |  | 699 | Metabolic |
| DN14830_c0_g1_i1.g1783.t1 | Developmental regulator flbA | RGS domain | 4.9 |  | 464 | Signal transduction |
| DN14875_c0_g1_i1.g1799.t1 | alcohol oxidase | Glucose-methanol-choline oxidoreductase, C-terminal | 6.2 |  | 216 | Oxidation-reduction |
| DN14966_c0_g1_i1.g3492.t1 | antigenic cell wall galactomanno | Cell wall mannoprotein 1 | 7.6 | Extracellular | 172 | Fungal-type cell wall organization |
| DN1504_c0_g1_i2.g37152.t1 | hypothetical protein FOPG_10049 |  | 5.0 |  | 243 |  |
| DN15068_c0_g1_i1.g993.t1 | hypothetical protein BFJ65_g12668 |  | 9.1 |  | 73 |  |
| DN15091_c0_g1_i1.g981.t1 | hypothetical protein BFJ65_g9912 | Uncharacterised conserved protein UCP014753 | 4.2 |  | 281 |  |
| DN1539_c0_g1_i1.g18934.t1 | hypothetical protein BFJ69_g5684 | Carbon-nitrogen hydrolase | 5.2 |  | 376 | Metabolic |
| DN15496_c0_g1_i1.g46603.t1 | hypothetical protein FOXG_02597 | NodB homology domain | 4.1 | Extracellular | 255 | Metabolic |
| DN15663_c0_g1_i1.g23280.t1 | hypothetical protein BFJ72_g5189 | Major facilitator, sugar transporter-like | 6.0 |  | 193 | Transport |
| DN15812_c0_g1_i1.g21649.t1 | hypothetical protein BFJ68_g3432 | peptidase S8 family | 5.4 |  | 108 | Proteolysis |
| DN15895_c0_g1_i1.g21660.t1 | General alpha-glucoside permease | Major facilitator, sugar transporter-like | 3.2 |  | 95 | Transport |
| DN160_c0_g1_i6.g8531.t1 | hypothetical protein FPSE_08661 | Ribosomal protein S11 | 3.2 |  | 151 | Translation |
| DN16432_c0_g1_i1.g27725.t1 | hypothetical protein BFJ65_g12585 | pectinesterase | 4.2 |  | 230 | Metabolic |
| DN1663_c0_g1_i1.g1425.t1 | phenylalanyl-tRNA synthetase, beta subunit | tRNA synthetase, B5-domain | 4.2 |  | 609 | phenylalanyl-tRNA aminoacylation |
| DN16736_c0_g1_i1.g10181.t1 | hypothetical protein FOCG_10579 |  | 3.7 |  | 751 |  |
| DN16768_c0_g1_i1.g10221.t1 | Acetyl-coenzyme A synthetase | Acetate-CoA ligase | 3.3 |  | 719 | Biosynthetic |
| DN16792_c0_g1_i1.g10212.t1 | Chaperone protein hchA | Class I glutamine amidotransferase-like | 4.9 |  | 152 | Metabolic |
| DN16882_c0_g1_i1.g17133.t1 | L-aminoadipate-semialdehyde dehydrogenase | AMP-dependent synthetase/ligase | 1.2 |  | 1183 |  |
| DN16884_c0_g1_i1.g17108.t1 | related to dihydrodipicolinate synthase | DapA-like | 1.3 |  | 330 |  |
| DN1698_c0_g1_i1.g1355.t1 | hypothetical protein FOMG_15916 | Aminotransferase, class I/classII | 3.8 |  | 283 | Biosynthetic |
| DN17002_c0_g1_i1.g6513.t1 | Midasin | von Willebrand factor A-like domain superfamily | 1.9 |  | 870 |  |
| DN17219_c0_g1_i1.g5187.t1 | glucan 1,6-alpha-glucosidase | Glycoside hydrolase superfamily | 5.5 |  | 147 | Metabolic |
| DN1722_c0_g1_i3.g4697.t1 | probable MRT4-mRNA turnover 4 | Ribosomal protein L10P | 1.0 |  | 244 | Translation |
| DN17230_c0_g1_i1.g5178.t1 | hypothetical protein FOCG_10400 | FAD binding domain | 5.4 | Cytoplasm | 211 |  |
| DN17312_c0_g1_i1.g11817.t1 | D/L-glyceraldehyde reductase | NADP-dependent oxidoreductase domain | 4.2 |  | 101 | Oxidation-reduction |
| DN17313_c0_g1_i2.g11843.t1 | probable PRS5-ribose-phosphate pyrophosphokinases | Ribose-phosphate pyrophosphokinase | 2.7 |  | 442 | Biosynthetic |
| DN17673_c0_g1_i1.g17447.t1 | Transcription-associated protein 1 | Belongs to the PI3 PI4-kinase family | 3.6 |  | 289 |  |
| DN177_c0_g1_i3.g8732.t1 | hypothetical protein FPSE_09944 | Ribosomal protein L14P | 3.2 | Mitochondrion | 139 | Translation |
| DN17787_c0_g1_i1.g16343.t1 | phosphate permease 84, partial | Major facilitator, sugar transporter-like | 3.5 |  | 531 | Transport |
| DN17935_c0_g1_i1.g25307.t1 | hypothetical protein BFJ70_g10011 | Peptidase M20, dimerisation domain | 4.5 |  | 355 | Proteolysis |
| DN18222_c0_g1_i1.g25071.t1 | hypothetical protein BFJ69_g7796 | Haloacid dehalogenase-like hydrolase (HAD superfamily) | 3.8 |  | 174 |  |
| DN18228_c0_g1_i1.g25065.t1 | hypothetical protein FOC4_g10002608 |  | 4.2 |  | 99 |  |
| DN1839_c0_g1_i1.g8163.t1 | fatty acid synthase subunit alpha | Fatty acid synthase type I, helical | 2.9 |  | 1746 | Oxidation-reduction |
| DN1845_c0_g1_i1.g8290.t1 | related to hexamer-binding protein HEXBP | Zinc finger, CCHC-type | 3.3 | Cytoplasm | 223 | Transcription |
| DN19091_c0_g1_i1.g18414.t1 | hypothetical protein FOXG_16569 |  | 3.1 |  | 145 |  |
| DN19096_c0_g1_i1.g18419.t1 | hypothetical protein FOXG_03446 | Bud22 domain | 1.8 |  | 339 | Ribosome biogenesis |
| DN191_c0_g1_i1.g9020.t1 | Indoleamine 2,3-dioxygenase family protein | Indoleamine 2,3-dioxygenase-like | 5.6 |  | 361 | Catabolic |
| DN1915_c0_g1_i1.g26367.t1 | hypothetical protein BFJ68_g9415 | Protein kinase domain | 6.2 |  | 477 | Signal transduction |
| DN1927_c0_g1_i1.g26292.t1 | hypothetical protein BFJ69_g10033 |  | 4.4 |  | 353 |  |
| DN1937_c0_g1_i1.g26191.t1 | eIF-2-alpha kinase activator GCN1 | Ribosomal protein L19/L19e | 2.5 |  | 201 | Translation |
| DN19406_c0_g1_i1.g9.t1 | hypothetical protein FOC1_g10015191 |  | 5.2 |  | 225 |  |
| DN19430_c0_g1_i1.g36.t1 | hypothetical protein FOC1_g10003790 | Acetyltransferase (GNAT) domain | 3.8 |  | 179 | Biosynthetic |
| DN19437_c0_g1_i1.g62.t1 | hypothetical protein FOC1_g10015557 | Methyltransferase type 11 | 2.7 |  | 918 | Methylation |
| DN19444_c0_g1_i2.g24.t1 | hypothetical protein FOC4_g10011844 | Major facilitator, sugar transporter-like | 4.5 |  | 147 | Transport |
| DN19963_c0_g1_i1.g6961.t1 | Flavohemoprotein | Oxidoreductase FAD/NAD(P)-binding | 5.5 |  | 255 | Oxidation-reduction |
| DN20_c0_g1_i1.g10471.t1 | hypothetical protein FPSE_12018 | Ribosomal protein S21e | 3.2 | Cytoplasm | 87 | Translation |
| DN20477_c0_g1_i1.g172.t1 | Beta-glucosidase 1B | Glycoside hydrolase family 1 | 3.4 |  | 93 | Metabolic |
| DN2055_c0_g1_i1.g9790.t1 | probable CPC2 protein | WD domain, G-beta repeat | 3.3 |  | 316 | Signal transduction |
| DN2059_c0_g1_i2.g9743.t1 | hypothetical protein BFJ65_g9754 | Glycoside hydrolase 131, catalytic N-terminal | 4.2 | Extracellular | 294 | Metabolic |
| DN20779_c0_g1_i1.g25041.t1 | hypothetical protein FOTG_14057 |  | 4.3 |  | 107 |  |
| DN210_c0_g1_i7.g2437.t1 | hypothetical protein BFJ71_g6545 | Ankyrin repeat-containing domain | 3.4 |  | 251 | Metabolic |
| DN2112_c0_g1_i1.g6232.t1 | translation initiation factor 4G | MIF4G-like, type 3 | 2.2 |  | 1393 |  |
| DN21448_c0_g1_i1.g24394.t1 | hypothetical protein FOXG_09795 | Golgi-associated plant pathogenesis-related protein 1, SCP domain | 4.8 |  | 259 |  |
| DN21522_c0_g1_i1.g148.t1 | Plasma membrane fusion protein PRM1 |  | 4.3 |  | 603 |  |
| DN21523_c0_g1_i1.g109.t1 | hypothetical protein BFJ67_g12805 | Glycosyl hydrolases family 18 (GH18) active site | 3.5 |  | 863 | Metabolic |
| DN2170_c0_g1_i1.g5985.t1 | probable FBP1-fructose-1,6-bisphosphatase | Fructose-1,6-bisphosphatase class 1 | 3.2 |  | 342 | Metabolic |
| DN21863_c0_g1_i1.g14013.t1 | cAMP-dependent protein kinase type 2 | Protein kinase domain-cAMP-dependent protein kinase | 5.2 |  | 563 | Signal transduction |
| DN22050_c0_g1_i1.g12132.t1 | hypothetical protein BFJ68_g12493 | PTP type protein phosphatase | 2.4 |  | 946 | Protein dephosphorylation |
| DN22078_c0_g1_i1.g12126.t1 | hypothetical protein FOTG_09068 | RNA recognition motif domain | 3.1 |  | 554 | Cytokinesis |
| DN22148_c0_g1_i1.g23101.t1 | hypothetical protein BFJ71_g1249 | 4-coumarate coenzyme A ligase | 3.9 | Mitochondrion | 111 |  |
| DN22194_c0_g1_i1.g23102.t1 | hypothetical protein FOC4_g10010679 |  | 5.2 |  | 185 |  |
| DN22201_c0_g1_i1.g17051.t1 | hypothetical protein BFJ69_g4370 | Glycoside hydrolase, family 61 | 6.3 |  | 262 | Metabolic |
| DN22248_c0_g1_i1.g17036.t1 | hypothetical protein BFJ68_g2848 | helicase activity | 4.3 |  | 144 | Chromatin organization |
| DN2232_c0_g1_i2.g14719.t1 | hypothetical protein BFJ71_g16342, partial |  | 6.8 |  | 2088 |  |
| DN22791_c0_g1_i1.g9687.t1 | hypothetical protein BFJ70_g14817, partial |  | 4.8 |  | 110 |  |
| DN2297_c0_g1_i1.g14666.t1 | hypothetical protein FOC4_g10011765 | Cytochrome b-c1 complex subunit 10, fungi | 1.6 |  | 88 | Oxidation-reduction |
| DN23641_c0_g1_i1.g12036.t1 | hypothetical protein BFJ71_g2407 | Major facilitator, sugar transporter-like | 7.0 |  | 172 | Transport |
| DN238_c0_g1_i2.g2689.t1 | dihydropteroate synthase | Dihydropteroate synthase | 7.5 |  | 534 | Biosynthetic |
| DN240_c0_g1_i7.g53575.t1 | hypothetical protein FOTG_08741 | Zinc finger C2H2-type | 3.9 |  | 569 | Transcription |
| DN2405_c0_g1_i1.g6639.t1 | NADP-specific glutamate dehydrogenase | Glutamate/phenylalanine/leucine/valine dehydrogenase, dimerisation domain | 3.8 |  | 456 | Oxidation-reduction |
| DN245_c0_g1_i4.g2462.t1 | hypothetical protein BFJ69_g9565 | Protein of unknown function (DUF3712) | 7.2 |  | 841 |  |
| DN2482_c0_g1_i2.g6835.t1 | hypothetical protein FOCG_10489 | Peptidase M20 | 4.9 |  | 467 | Proteolysis |
| DN2554_c0_g1_i2.g16675.t1 | uncharacterized protein FFUJ_05875 |  | 4.4 | Extracellular | 190 |  |
| DN2563_c0_g1_i1.g16458.t1 | hypothetical protein FOTG_12342 | Only prolin and serin are matching in the corresponding protein | 2.6 |  | 577 |  |
| DN2633_c0_g1_i2.g21485.t1 | hypothetical protein FOC1_g10002770 |  | 3.2 | Extracellular | 203 |  |
| DN2649_c0_g1_i1.g21252.t1 | probable PAB1-mRNA polyadenylate-binding protein | RNA recognition motif domain | 1.1 |  | 750 | Cytokinesis |
| DN2701_c0_g1_i1.g5418.t1 | phosphoadenosine phosphosulfate reductase | Phosphoadenosine phosphosulphate/adenosine 5'-phosphosulphate reductase | 3.9 |  | 316 | Oxidation-reduction |
| DN2753_c0_g1_i2.g5435.t1 | Choline transport protein | Amino acid/polyamine transporter I | 3.6 |  | 390 | Transport |
| DN2785_c0_g1_i1.g5250.t1 | putative glycine dehydrogenase (decarboxylating) | Glycine cleavage system P protein | 1.4 |  | 974 | Oxidation-reduction |
| DN2844_c0_g1_i1.g24888.t1 | probable sulfate adenylyltransferase | Sulphate adenylyltransferase catalytic domain | 4.7 |  | 574 | Sulfate assimilation |
| DN2948_c0_g1_i1.g16076.t1 | putative glutamate synthase | Glutamate synthase domain | 1.5 |  | 2029 | Oxidation-reduction |
| DN3037_c0_g1_i1.g494.t1 | Putative mitochondrial carnitine O-acetyltransferase | Choline/carnitine acyltransferase domain | 4.1 |  | 931 | Proteolysis |
| DN3038_c0_g1_i1.g381.t1 | hypothetical protein FOIG_06146 | Peroxisome membrane anchor protein Pex14p, N-terminal | 5.6 |  | 352 |  |
| DN3228_c0_g1_i1.g17413.t1 | hypothetical protein FOIG_12221 | MICOS complex subunit MIC26/MIC27 | 1.3 | Mitochondrion | 232 |  |
| DN3230_c0_g1_i3.g17331.t1 | hypothetical protein BFJ72_g13236 | Sulfotransferase family | 3.7 |  | 302 |  |
| DN3285_c0_g1_i1.g17431.t1 | hypothetical protein BFJ71_g9688 |  | 4.7 | Cytoplasm | 75 |  |
| DN3298_c1_g1_i1.g17201.t1 | hypothetical protein FOC4_g10014865 | glycosyl-phosphatidyl-inositol-anchored membrane family (GPI) | 2.8 |  | 262 | Proteolysis |
| DN3347_c0_g1_i3.g5773.t1 | hypothetical protein FOC1_g10008772 | family decarboxylase | 4.5 |  | 386 |  |
| DN3379_c0_g1_i1.g10071.t1 | uncharacterized protein FOYG_12748 | Growth factor receptor cysteine-rich domain superfamily | 6.5 |  | 278 |  |
| DN3395_c0_g1_i1.g5630.t1 | hypothetical protein BFJ65_g869 | Clr5 domain | 3.3 |  | 496 |  |
| DN3411_c0_g1_i1.g22919.t1 | hypothetical protein FOXG_02808 | Major facilitator, sugar transporter-like | 4.5 |  | 557 | Transport |
| DN3427_c0_g1_i1.g22725.t1 | fatty acid synthase subunit beta, fungi type | Fatty acid synthase | 2.5 |  | 1814 | Oxidation-reduction |
| DN344_c0_g1_i1.g22362.t1 | hypothetical protein FPOA_01302 | Ribosomal protein S28e | 3.3 |  | 68 | Translation |
| DN3457_c0_g1_i1.g22824.t1 | hypothetical protein BFJ69_g3711 | DNA polymerase V/Myb-binding protein 1A | 1.6 |  | 1006 | Transcription |
| DN3782_c0_g1_i2.g11889.t1 | hypothetical protein BFJ69_g1977 | RlpA-like domain superfamily | 5.5 | Extracellular | 220 |  |
| DN3816_c0_g1_i1.g24511.t1 | hypothetical protein FOTG_04049 |  | 4.6 |  | 197 |  |
| DN3858_c0_g1_i1.g24643.t1 | Protein pyrABCN | Carbamoyl-phosphate synthase large subunit, CPSase domain | 5.9 |  | 937 | Metabolic |
| DN3953_c0_g1_i1.g1628.t1 | hypothetical protein FOCG_05131 | Aminotransferase class IV | 4.6 |  | 394 | Biosynthetic |
| DN419_c0_g1_i1.g12881.t1 | hypothetical protein BFJ71_g11557 | Ribosomal protein S17/S11 | 2.5 |  | 161 | Translation |
| DN4192_c0_g1_i2.g22640.t1 | hypothetical protein FOXG_04484 | Coenzyme A transferase family I | 4.5 |  | 494 | Catabolic |
| DN4256_c0_g1_i1.g17492.t1 | hypothetical protein FGSG_09827 |  | 3.8 |  | 93 |  |
| DN4271_c0_g1_i1.g17474.t1 | hypothetical protein BFJ65_g6824 |  | 3.5 |  | 304 |  |
| DN4308_c0_g1_i1.g11614.t1 | hypothetical protein BFJ71_g13037 | Amine oxidase | 4.3 |  | 527 | Oxidation-reduction |
| DN4424_c0_g1_i1.g19339.t1 | Succinate/fumarate mitochondrial transporter | Mitochondrial carrier protein | 5.6 |  | 323 | Transport |
| DN4454_c0_g1_i1.g19441.t1 | Aldehyde reductase 1 | NADP-dependent oxidoreductase domain | 3.6 |  | 105 | Oxidation-reduction |
| DN4526_c0_g1_i1.g25213.t1 | Purine-cytosine permease FCY21 | Purine-cytosine permease | 3.8 |  | 501 | Transport |
| DN466_c0_g1_i4.g12924.t1 | hypothetical protein FPSE_06056 | Ribosomal protein S26e | 3.2 |  | 117 | Translation |
| DN4772_c0_g1_i1.g1925.t1 | L-galactonate dehydratase | Enolase C-terminal domain-like | 3.2 |  | 450 |  |
| DN4835_c0_g1_i1.g19006.t1 | hypothetical protein BFJ65_g9718 |  | 4.5 |  | 1140 |  |
| DN485_c0_g1_i2.g13093.t1 | probable ribosomal protein L13B | Ribosomal protein L13e | 1.7 |  | 212 | Translation |
| DN4860_c0_g1_i1.g18944.t1 | hypothetical protein FOXG_06133 |  | 7.8 |  | 129 |  |
| DN4896_c0_g1_i2.g19054.t1 | hypothetical protein FOIG_10150 | Glucose receptor Git3, N-terminal-G protein-coupled | 3.1 |  | 407 | Signal transduction |
| DN5061_c1_g1_i1.g9604.t1 | uncharacterized protein FPRN_09263 |  | 1.5 |  | 505 |  |
| DN5086_c0_g1_i1.g9621.t1 | oxidoreductase | Oxidoreductase, N-terminal | 3.6 |  | 416 | Oxidation-reduction |
| DN5272_c0_g1_i1.g19327.t1 | hypothetical protein BFJ69_g6218 |  | 4.1 |  | 475 |  |
| DN5318_c0_g1_i1.g11706.t1 | Uncharacterized protein RSN1 | Calcium-dependent channel, 7TM region, putative phosphate | 4.0 |  | 892 | Transport |
| DN5366_c0_g1_i1.g10250.t1 | hypothetical protein BFJ68_g9850 | Peptidase S8/S53 domain | 3.7 |  | 562 | Proteolysis |
| DN5393_c0_g1_i1.g11683.t1 | Annexin A11 | Annexin repeat | 3.0 |  | 488 |  |
| DN546_c0_g1_i3.g18338.t1 | hypothetical protein FAVG1_02914 | Ribosomal protein L15, conserved site | 3.3 |  | 149 | Translation |
| DN5569_c0_g1_i1.g205.t1 | Lipid phosphate phosphatase 1 | Phosphatidic acid phosphatase type 2/haloperoxidase | 3.3 |  | 375 | Metabolic |
| DN5603_c0_g1_i1.g25075.t1 | hypothetical protein BFJ69_g7303 | Acetyltransferase (GNAT) family | 3.6 |  | 165 | Biosynthetic |
| DN5768_c0_g1_i1.g19843.t1 | hypothetical protein BFJ69_g6260 | Glycoside hydrolase, family 61 (AA9 CAZyme) | 5.0 |  | 248 | Metabolic |
| DN5871_c0_g1_i1.g14286.t1 | hypothetical protein BFJ70_g15072 | Galactose-binding-like domain superfamily | 4.8 |  | 215 |  |
| DN5895_c0_g1_i1.g14314.t1 | hypothetical protein BFJ68_g12847 | OPT oligopeptide transporter protein | 4.1 |  | 100 | Transport |
| DN5971_c0_g1_i1.g6390.t1 | hypothetical protein FOC4_g10007522 |  | 8.0 | Extracellular | 152 |  |
| DN6092_c0_g1_i1.g4240.t1 | hypothetical protein BFJ65_g13903 | Pectate lyase PlyH/PlyE-like | 4.5 |  | 226 | Metabolic |
| DN6129_c0_g1_i1.g26826.t1 | alcohol dehydrogenase | Aldo/keto reductase | 4.7 |  | 315 | Oxidation-reduction |
| DN6184_c0_g1_i1.g26804.t1 | hypothetical protein BFJ68_g6384 | Carbohydrate-binding, CenC-like | 3.9 |  | 267 | Metabolic |
| DN6189_c0_g1_i1.g26844.t1 | UNC93-like protein | Ion channel regulatory protein UNC-93 | 4.6 |  | 115 | Transport |
| DN622_c0_g1_i1.g15009.t1 | hypothetical protein BFJ72_g12283 | Nascent polypeptide-associated complex NAC domain | 1.7 |  | 154 | Transport |
| DN6332_c0_g1_i1.g3373.t1 | hypothetical protein FOXG_04936 | Major facilitator, sugar transporter-like | 6.6 |  | 172 | Transport |
| DN6432_c0_g1_i2.g19463.t1 | hypothetical protein BFJ65_g11029 |  | 4.4 |  | 280 |  |
| DN647_c0_g1_i4.g15255.t1 | hypothetical protein FPSE_03509 | Ribosomal protein S10 | 3.5 |  | 116 | Translation |
| DN649_c1_g1_i8.g15134.t1 | hypothetical protein FOC4_g10015188 | Protein of unknown function DUF3712 | 3.8 |  | 343 |  |
| DN6661_c0_g1_i1.g4823.t1 | endopolygalacturonase PG2 | Glycoside hydrolase, family 28 | 4.7 | Extracellular | 279 | Metabolic |
| DN6672_c0_g1_i3.g4829.t1 | Nitrogen regulatory protein areA | Nitrogen regulatory protein areA, GATA-like domain | 7.3 |  | 386 | Regulation of nitrogen utilization |
| DN67_c0_g1_i2.g10594.t1 | hypothetical protein FOMG_11171 | Ribosomal protein L24e | 1.9 |  | 472 | Translation |
| DN6701_c0_g1_i1.g883.t1 | hypothetical protein BFJ69_g263 | Haloacid dehalogenase-like (HAD superfamily) | 4.5 |  | 365 |  |
| DN673_c0_g1_i4.g15310.t1 | 2-amino-3-carboxymuconate-6-semialdehyde decarboxylase | Amidohydrolase-related | 3.7 |  | 364 | Proteolysis |
| DN6875_c0_g1_i1.g12865.t1 | L-asparaginase | L-asparaginase II | 3.7 |  | 360 | Metabolic |
| DN7_c0_g1_i1.g11254.t1 | hypothetical protein BFJ69_g10702 | Hex1, S1 domain | 1.2 |  | 292 |  |
| DN7_c1_g1_i1.g11255.t1 | hypothetical protein BFJ65_g9368 | Woronin body major protein | 2.2 |  | 198 | Translation |
| DN7002_c0_g1_i1.g26754.t1 | probable O-acetylhomoserine (thiol)-lyase | Cys/Met metabolism, pyridoxal phosphate-dependent enzyme | 3.9 | Cytoplasm | 127 | Transsulfuration |
| DN7393_c0_g1_i1.g923.t1 | hypothetical protein FOCG_13184 |  | 4.3 |  | 425 |  |
| DN745_c0_g1_i1.g20430.t1 | hypothetical protein BFJ65_g12600 | Zinc finger C2H2-type | 3.8 |  | 576 | Transcription |
| DN7499_c0_g1_i1.g14086.t1 | flavohemoglobin | Globin/Protoglobin | 6.2 |  | 160 | Transport |
| DN7762_c0_g1_i1.g21707.t1 | hypothetical protein FOTG_15499 | Uncharacterised protein family UPF0311 | 5.5 |  | 125 |  |
| DN7808_c0_g1_i2.g20895.t1 | Transcriptional regulatory protein pro-1 | Zn (2)-C6 fungal-type DNA-binding domain | 5.0 |  | 665 | Transcription |
| DN7936_c0_g1_i1.g10281.t1 | Protein alcS | Acetate transporter GPR1/FUN34/SatP family | 6.0 |  | 182 | Transport |
| DN800_c0_g1_i3.g25718.t1 | hypothetical protein FOXG_01279 |  | 8.1 |  | 380 |  |
| DN8091_c0_g1_i1.g16403.t1 | hypothetical protein BFJ70_g7983 | Major facilitator, sugar transporter-like | 5.7 |  | 246 | Transport |
| DN8096_c0_g1_i1.g16386.t1 | hypothetical protein FOXG_14666 | Major facilitator, sugar transporter-like | 4.2 |  | 413 | Transport |
| DN8265_c0_g1_i1.g16894.t1 | Sodium/potassium-transporting ATPase subunit alpha-2 | Cation-transporting P-type ATPase, C-terminal | 4.1 |  | 270 | Transport |
| DN829_c0_g1_i3.g25626.t1 | Phospholipase D1 | Phospholipase D/Transphosphatidylase | 6.0 |  | 1428 | Catabolic |
| DN831_c0_g1_i2.g25779.t1 | hypothetical protein FOXG_11163 |  | 2.2 |  | 723 |  |
| DN84_c0_g1_i1.g10587.t1 | hypothetical protein FPSE_09105 | Ribosomal protein L37ae | 3.9 |  | 92 | Translation |
| DN8431_c0_g1_i1.g263.t1 | hypothetical protein BFJ71_g15474 | Major facilitator, sugar transporter-like | 3.4 |  | 116 | Transport |
| DN8509_c0_g1_i1.g19857.t1 | Homocitrate synthase, mitochondrial | Pyruvate carboxyltransferase | 3.9 |  | 282 | Metabolic |
| DN880_c0_g1_i1.g25593.t1 | probable bifunctional D12/D15 fatty acid desaturase | Fatty acid desaturase domain | 5.8 |  | 402 | Metabolic |
| DN8846_c0_g1_i1.g21615.t1 | hypothetical protein FOC4_g10006682 | Tyrosinase copper-binding domain | 3.9 |  | 368 | Oxidation-reduction |
| DN895_c0_g1_i1.g25451.t1 | probable PRX1-mitochondrial isoform of thioredoxin peroxidase | Alkyl hydroperoxide reductase subunit C/ Thiol specific antioxidant | 4.2 |  | 227 | Oxidation-reduction |
| DN9037_c0_g1_i2.g7958.t1 | hypothetical protein BFJ68_g9844 | Jacalin-like lectin domain | 4.5 |  | 387 |  |
| DN9084_c0_g1_i1.g7960.t1 | Dicarboxylic amino acid permease | Amino acid permease/ SLC12A domain | 3.1 |  | 544 | Transport |
| DN91_c0_g1_i6.g10513.t1 | putative ribosomal protein L9.e.c14 | Ribosomal protein L6, alpha-beta domain | 1.6 |  | 193 | Translation |
| DN912_c0_g1_i1.g4102.t1 | Nucleoside diphosphate kinase | Nucleoside diphosphate kinase | 3.2 |  | 238 | Biosynthetic |
| DN9134_c0_g1_i1.g19907.t1 | hypothetical protein FOCG_05432 |  | 5.9 |  | 129 |  |
| DN9147_c0_g1_i1.g19923.t1 | hypothetical protein FOCG_08325 | Alkaline-phosphatase-like, core domain superfamily | 3.9 |  | 184 | Protein dephosphorylation |
| DN930_c1_g1_i1.g3810.t1 | Putative N-acetylglucosamine-6-phosphate deacetylase | N-acetylglucosamine-6-phosphate deacetylase | 4.5 |  | 439 | Metabolic |
| DN9331_c0_g1_i1.g1435.t1 | hypothetical protein FOC4_g10009928 |  | 5.5 |  | 187 |  |
| DN9396_c0_g1_i2.g1442.t1 | Putative rhamnogalacturonate lyase A | Rhamnogalacturonan lyase, domain II | 5.4 | Mitochondrion | 264 | Metabolic |
| DN952_c0_g1_i2.g3822.t1 | hypothetical protein FPSE_08712 | Ribosomal protein S19e | 2.2 |  | 150 | Translation |
| DN9660_c0_g1_i1.g5232.t1 | Lactose permease | Major facilitator, sugar transporter-like | 4.5 |  | 168 | Transport |
| DN967_c0_g1_i1.g3701.t1 | Maintenance of ploidy protein mob2 | MOB kinase activator family | 8.2 |  | 340 | Signal transduction |
| DN975_c0_g1_i1.g3894.t1 | Elongation factor 1-alpha | Translation elongation factor EFTu/EF1A, C-terminal | 3.4 |  | 460 | Translation |
| DN9935_c0_g1_i1.g47025.t1 | Exoglucanase 1 | Cellulose-binding domain, fungal | 4.7 |  | 252 | Metabolic |
| DN9961_c0_g1_i1.g47044.t1 | hypothetical protein FOCG_06107 |  | 9.4 |  | 301 |  |
