# Supplementary material for: Comparative transcriptomic analysis of races 1, 2, 5 and 6 of Fusarium oxysporum f.sp. pisi in a susceptible pea host identifies differential pathogenicity profiles
Source: BMC Genomics. 2021 Oct 9;22:734. doi: 10.1186/s12864-021-08033-y (PMC8502283; doi:10.1186/s12864-021-08033-y)
Supplement: Supplementary file 4 — Additional file 4: Table S2. Differentially expressed Fop genes detected in R2 at 20 dpi - column 1 with other analyses such as: predicted proteins - column 2, conserved domain - column 3, log2fold change - column 4, subcellular localisation of the effector-like proteins - column 5, protein length - column 6, and GO functional enrichment for biological processes (BP) - column 7. DEGs predicted to be effector-like are shaded yellow and they were all located on the adaptive genome. [file 12864_2021_8033_MOESM4_ESM.docx]

**Supplementary Table 2**

| NODE_100.g14760.t1 | hypothetical protein FOIG_02090 | Zinc finger C2H2 superfamily | 2.3 |  | 450 | Transcription |
| --- | --- | --- | --- | --- | --- | --- |
| NODE_102.g14860.t1 | hypothetical protein BFJ69_g8042 | Pectin lyase fold/virulence factor | 5.1 | Extracellular | 391 | Metabolic |
| NODE_102.g14871.t1 | hypothetical protein FOC4_g10008541 | Cupredoxin | 4.4 |  | 382 | Oxidation-reduction |
| NODE_103.g14941.t1 | uncharacterized protein FPRN_04559 | 60S ribosomal protein | 2.9 |  | 58 | Translation |
| NODE_103.g14948.t1 | DNA-directed RNA polymerases I and III subunit RPAC2 | DNA-directed RNA polymerase, RBP11-like dimerisation domain | 2.4 |  | 147 | Transcription |
| NODE_103.g14976.t1 | Nucleolar protein 56 | NOP5, N-terminal | 3.4 |  | 511 | Ribosome biogenesis |
| NODE_103.g14978.t1 | Cellular nucleic acid-binding protein like protein | Zinc finger, CCHC-type superfamily | 5.1 | Cytoplasm | 182 | Transcription |
| NODE_104.g14989.t1 | enoyl- | Nitronate monooxygenase | 1.9 |  | 330 | Oxidation-reduction |
| NODE_104.g14991.t1 | Pyrroline-5-carboxylate reductase | Pyrroline-5-carboxylate reductase | 1.8 |  | 232 | Oxidation-reduction |
| NODE_104.g15006.t1 | Threonine synthase | Threonine synthase, N-terminal | 1.6 |  | 539 | Biosynthetic |
| NODE_104.g15042.t1 | probable farnesyl-pyrophosphate synthetase | Polyprenyl synthetase | 2.2 |  | 347 | Biosynthetic |
| NODE_105.g4094.t1 | Dicarboxylic amino acid permease | Amino acid permease/ SLC12A domain | 2.3 |  | 577 | Transport |
| NODE_106.g15133.t1 | Myosin-2 | Myosin head, motor domain | 1.2 |  |  |  |
| NODE_106.g4105.t1 | hypothetical protein FOC4_g10011768 | Sas10/Utp3/C1D family | 2.9 |  | 362 |  |
| NODE_106.g4106.t1 | DNA-directed RNA polymerase III subunit rpc5 | Sin-like protein conserved region | 2.3 |  | 406 | Transcription |
| NODE_106.g4110.t1 | Uncharacterized protein LW93_10714 | Acyl-CoA oxidase/dehydrogenase, central domain | 2.6 |  | 446 | Oxidation-reduction |
| NODE_106.g4119.t1 | hypothetical protein FOC1_g10010203 |  | 1.7 |  | 809 |  |
| NODE_107.g15180.t1 | hypothetical protein FOTG_01958 | CFEM domain | 3.9 |  | 373 |  |
| NODE_107.g15184.t1 | hypothetical protein FOQG_09103 | Mitochondrial substrate/solute carrier | 6.3 |  | 296 | Transport |
| NODE_107.g15185.t1 | hypothetical protein FOQG_09102 | Mitochondrial protein | 2.4 |  | 291 | Metabolic |
| NODE_107.g15207.t1 | hypothetical protein FOTG_01242 | Phosphoribosyltransferase domain | 2.9 |  | 206 | Metabolic |
| NODE_107.g15223.t1 | hypothetical protein FOQG_06232 | S-adenosyl-L-methionine-dependent methyltransferase | 3.1 |  | 1463 | Methylation |
| NODE_107.g4138.t1 | Carbonic anhydrase | Carbonic anhydrase, prokaryotic-like | 3.9 | Extracellular | 289 | carbon utilization |
| NODE_107.g4147.t1 | Amino-acid permease inda1 | Amino acid/polyamine transporter I | 6.3 |  | 581 | Transport |
| NODE_107.g4151.t1 | MFS transporter, SP family, general alpha glucoside:H+ symporter | Major facilitator, sugar transporter-like | 8.0 |  | 541 | Transport |
| NODE_108.g15227.t1 | hypothetical protein BFJ69_g12427 | RNA recognition motif domain | 2.7 |  | 422 | Cytokinesis |
| NODE_108.g15243.t1 | hypothetical protein FOXG_01984 |  | 1.8 |  | 485 |  |
| NODE_108.g15244.t1 | hypothetical protein FAVG1_12529 | P-loop containing nucleoside triphosphate hydrolase | 2.8 |  | 396 | Phosphorylation |
| NODE_108.g15246.t1 | probable sulfate adenylyltransferase | Sulphate adenylyltransferase | 4.0 |  | 574 | Sulfate assimilation |
| NODE_108.g15252.t1 | hypothetical protein FAVG1_05733 | Chaperone J-domain superfamily | 1.3 |  | 1213 | Stress response |
| NODE_108.g15256.t1 | AGC/RSK/RSKP70 protein kinase | Protein kinase domain-AGC-kinase, C-terminal | 2.0 |  | 515 | Signal transduction |
| NODE_108.g15261.t1 | Phenylacetaldehyde dehydrogenase | Aldehyde dehydrogenase domain | 1.8 |  | 467 | Oxidation-reduction |
| NODE_108.g15263.t1 | probable nuclear export sequence-containing nonribosomal protein | Nmd3, N-terminal | 3.0 |  | 517 |  |
| NODE_108.g4164.t1 | hypothetical protein FOIG_08082 | Major facilitator superfamily | 1.4 |  | 566 | Transport |
| NODE_109.g15295.t1 | ribose-phosphate pyrophosphokinase | Ribose-phosphate pyrophosphokinase | 2.3 |  | 461 | Biosynthetic |
| NODE_109.g15322.t1 | hypothetical protein FOIG_02155 | Heat shock protein 70 family | 4.2 |  | 338 | Stress response |
| NODE_109.g15343.t1 | Protein sof1 | WD domain, G-beta repeat | 2.5 |  | 445 | Signal transduction |
| NODE_109.g15345.t1 | Choline transport protein | Amino acid/polyamine transporter I | 4.4 |  | 531 | Transport |
| NODE_109.g4193.t1 | Transcription elongation factor SPT6 | Helix-turn-helix DNA-binding domain of Spt6 | 1.0 |  | 1406 | Transcription |
| NODE_110.g15358.t1 | hypothetical protein FOTG_00133 | Ribosomal RNA methyltransferase FtsJ domain | 3.4 |  | 794 | Methylation |
| NODE_110.g15378.t1 | betaine-aldehyde dehydrogenase | Aldehyde dehydrogenase domain | 3.3 |  | 509 | Oxidation-reduction |
| NODE_110.g4217.t1 | Ras-like protein | Small GTPase (Ras family (G)) | 2.7 | Cell membrane | 216 | Signal Transduction |
| NODE_111.g15407.t1 | Orotidine 5'-phosphate decarboxylase | Orotidine 5'-phosphate decarboxylase domain | 1.9 |  | 366 | Biosynthetic |
| NODE_111.g15409.t1 | hypothetical protein BFJ68_g10103 | Zinc finger, CCCH-type | 2.2 |  | 457 | Transcription |
| NODE_111.g15423.t1 | Gluconolactonase | SMP-30/Gluconolactonase/LRE-like region | 7.7 |  | 903 | Regulation of catalytic activity |
| NODE_111.g15435.t1 | hypothetical protein BFJ69_g15343 | La-type HTH domain | 3.7 |  | 764 | RNA processing |
| NODE_111.g15457.t1 | Adenylosuccinate lyase | Adenylosuccinate lyase C-terminal | 3.3 |  | 283 | Biosynthetic |
| NODE_112.g15480.t1 | hypothetical protein FOPG_17151 |  | 5.1 |  | 1416 |  |
| NODE_112.g15497.t1 | probable iron inhibited ABC transporter 2 | ABC transporter-like | 3.5 |  | 618 | Transport |
| NODE_112.g4256.t1 | hypothetical protein FOPG_13741 | Galactose-binding-like domain superfamily | 3.1 |  | 233 |  |
| NODE_112.g4271.t1 | hypothetical protein FOMG_04529 | Amino acid/polyamine transporter I | 1.9 |  | 525 | Transport |
| NODE_113.g15511.t1 | General alpha-glucoside permease | Major facilitator, sugar transporter-like | 4.7 |  | 561 | Transport |
| NODE_113.g4272.t1 | eukaryotic translation initiation factor 6 | Translation initiation factor IF6 | 1.4 |  | 246 | Translation |
| NODE_114.g15596.t1 | hypothetical protein FOTG_13073 | Aminotransferase, class I/classII | 8.4 |  | 423 | Biosynthetic |
| NODE_114.g15597.t1 | hypothetical protein FOXG_20246 | Eukaryotic molybdopterin oxidoreductase | 7.2 |  | 357 | Oxidation-reduction |
| NODE_115.g15604.t1 | hypothetical protein BFJ69_g14013 | Fungal specific transcription factor domain | 2.9 |  | 268 |  |
| NODE_115.g15607.t1 | Ketol-acid reductoisomerase, mitochondrial | Ketol-acid reductoisomerase, C-terminal | 1.4 |  | 308 | Oxidation-reduction |
| NODE_115.g15637.t1 | hypothetical protein FPSE_12018 | Ribosomal protein S21e | 2.8 | Cytoplasm | 87 | Translation |
| NODE_116.g15705.t1 | hypothetical protein FOMG_16002 | Rhodanese-like domain superfamily | 4.5 | Plastid | 154 |  |
| NODE_116.g15706.t1 | hypothetical protein FOMG_16003 | NADPH-dependent FMN reductase-like | 2.8 |  | 289 | Oxidation-reduction |
| NODE_116.g4386.t1 | guanosine-diphosphatase | Nucleoside phosphatase GDA1/CD39 | 1.7 |  | 545 |  |
| NODE_116.g4401.t1 | oxidoreductase | Oxidoreductase, N-terminal | 4.5 |  | 385 | Oxidation-reduction |
| NODE_117.g15713.t1 | uncharacterized protein FPRN_12758 | RING finger domain protein | 1.4 |  | 183 |  |
| NODE_117.g15722.t1 | arginyl-tRNA synthetase | Arginyl tRNA synthetase N-terminal domain | 2.1 |  | 635 | Arginyl-tRNA aminoacylation |
| NODE_117.g15732.t1 | hypothetical protein FOTG_12292 | Cellulose-binding domain | 6.9 |  | 397 | Metabolic |
| NODE_117.g15736.t1 | hypothetical protein BFJ69_g9570 | GTP binding | 2.6 |  | 614 | Signal transduction |
| NODE_117.g15740.t1 | hypothetical protein FPSE_00983 | Plectin/S10, N-terminal | 2.5 |  | 165 | Cytoskeleton organization |
| NODE_119.g15845.t1 | 16S rRNA (-N6/-N6)-dimethyltransferase | Ribosomal RNA adenine methylase transferase, N-terminal | 3.0 |  | 343 | rRNA processing |
| NODE_119.g15850.t1 | hypothetical protein BFJ69_g466 | Small acidic protein-like domain | 2.3 |  | 345 |  |
| NODE_119.g15854.t1 | hypothetical protein FOXG_15891 | CFEM domain | 3.0 |  | 418 |  |
| NODE_119.g15858.t1 | beta-lactamase | Beta-lactamase-related | 5.5 |  | 388 |  |
| NODE_119.g4477.t1 | hypothetical protein FOC1_g10010582 | Alcohol acetyltransferase/N-acetyltransferase | 3.5 |  | 472 |  |
| NODE_119.g4484.t1 | seryl-tRNA synthetase | Serine-tRNA ligase, type1 | 2.4 |  | 477 | Seryl-tRNA aminoacylation |
| NODE_120.g15872.t1 | hypothetical protein FOC1_g10002515 | Chaperone J-domain superfamily | 3.5 |  | 444 | Stress response |
| NODE_120.g15890.t1 | hypothetical protein FOXG_08948 | FAD linked oxidase, N-terminal | 6.6 |  | 495 | Oxidation-reduction |
| NODE_120.g15917.t1 | Altered inheritance rate of mitochondria protein 38 like protein | Hypoxia induced protein, domain | 4.1 |  | 228 | Transport |
| NODE_120.g15931.t1 | hypothetical protein BFJ69_g15028 | Armadillo-like helical | 3.6 |  | 445 |  |
| NODE_121.g15961.t1 | Methylthioribose-1-phosphate isomerase | Methylthioribose-1-phosphate isomerase | 1.5 |  | 388 | Biosynthetic |
| NODE_122.g15990.t1 | hypothetical protein FOCG_01276 | Cgr1-like | 2.1 |  | 119 | rRNA processing |
| NODE_122.g16006.t1 | hypothetical protein BFJ69_g4989 | Mitochondrial substrate/solute carrier | 3.4 |  | 336 | Transport |
| NODE_122.g4547.t1 | hypothetical protein BFJ69_g263 | Haloacid dehalogenase-like (HAD superfamily) | 3.5 |  | 365 |  |
| NODE_122.g4552.t1 | hypothetical protein FOQG_11114 |  | 1.0 |  | 373 |  |
| NODE_122.g4568.t1 | beta-galactosidase | Glycoside hydrolase, family 35 | 4.1 |  | 1021 | Metabolic |
| NODE_123.g16030.t1 | phosphoribosylformylglycinamidine synthase | Phosphoribosylformylglycinamidine synthase, N-terminal | 3.3 |  | 1355 | Biosynthetic |
| NODE_123.g16035.t1 | CMGC/MAPK/P38 protein kinase | Protein kinase domain-Mitogen-activated protein (MAP) kinase p38-like | 1.7 |  | 357 | Signal Transduction |
| NODE_123.g16040.t1 | hypothetical protein FOQG_09571 |  | 1.0 |  | 899 |  |
| NODE_123.g16041.t1 | Methylenetetrahydrofolate dehydrogenase | Methylenetetrahydrofolate dehydrogenase, NAD-binding domain | 2.1 |  | 338 | Oxidation-reduction |
| NODE_124.g16071.t1 | hypothetical protein FOMG_13186 | Bestrophin/UPF0187-chloride channel | 6.8 |  | 400 | Transport |
| NODE_124.g16091.t1 | hypothetical protein BFJ71_g11557 | Ribosomal protein S17/S11 | 2.5 |  | 160 | Translation |
| NODE_124.g16096.t1 | hypothetical protein FOC4_g10004609 |  | 2.0 |  | 540 |  |
| NODE_124.g4611.t1 | hypothetical protein BFJ65_g4001 | RNA recognition motif domain | 3.7 |  | 398 | Cytokinesis |
| NODE_124.g4616.t1 | hypothetical protein FOC1_g10006735 | Cytochrome P450 | 1.4 |  | 1067 | Oxidation-reduction |
| NODE_125.g16134.t1 | Homocitrate synthase, mitochondrial | Pyruvate carboxyltransferase | 4.1 |  | 383 | Metabolic |
| NODE_125.g16152.t1 | probable GAP1-General amino acid permease | Amino acid/polyamine transporter I | 3.3 |  | 536 | Transport |
| NODE_125.g4631.t1 | hypothetical protein BFJ69_g8756 | Linker histone H1/H5, domain H15 | 1.3 |  | 224 | Nucleosome assembly |
| NODE_126.g16197.t1 | hypothetical protein FOXG_00851 | RNA recognition motif domain | 2.5 |  | 855 | Cytokinesis |
| NODE_127.g16202.t1 | probable elongation factor 2 | Translation elongation factor EFTu-like, domain 2 | 1.8 |  | 844 | Translation |
| NODE_127.g16215.t1 | hypothetical protein FOMG_01616 | Lysine methyltransferase | 2.8 |  | 353 | Methylation |
| NODE_127.g16220.t1 | hypothetical protein FOTG_04036 | WD domain, G-beta repeat | 3.3 |  | 490 | Signal transduction |
| NODE_128.g16245.t1 | hypothetical protein FOXG_12263 | Peptidase S8/S53 domain | 5.2 |  | 397 | Proteolysis |
| NODE_128.g16246.t1 | Putative pectate lyase F | Pectate lyase PlyH/PlyE-like | 7.3 | Extracellular | 240 | Metabolic |
| NODE_128.g16251.t1 | hypothetical protein FOC1_g10011313 | Nitrogen regulatory protein areA, GATA-like domain | 1.2 |  | 562 | Regulation of nitrogen utilization |
| NODE_128.g4727.t1 | hypothetical protein FOC4_g10001384 | Letm1 ribosome-binding domain | 1.4 |  | 552 |  |
| NODE_129.g16306.t1 | hypothetical protein FVEG_11508 |  | 2.2 |  | 154 |  |
| NODE_129.g16326.t1 | putative unsaturated glucuronyl hydrolase | Glycosyl Hydrolase Family 88 | 4.6 |  | 372 | Metabolic |
| NODE_129.g16331.t1 | Allergen Asp f 4 | allergen asp | 1.3 |  | 316 |  |
| NODE_129.g4731.t1 | Cellobiose dehydrogenase | Cellobiose dehydrogenase, cytochrome domain | 6.7 |  | 801 | Metabolic |
| NODE_129.g4732.t1 | hypothetical protein BFJ69_g7621 | SGNH hydrolase-type esterase domain | 6.9 | Extracellular | 257 | Metabolic |
| NODE_129.g4740.t1 | Kynureninase 1 | Kynureninase | 5.7 |  | 493 | Biosynthetic |
| NODE_129.g4741.t1 | Indoleamine 2,3-dioxygenase family protein | Indoleamine 2,3-dioxygenase | 7.7 |  | 425 | Catabolic |
| NODE_129.g4742.t1 | hypothetical protein FOC1_g10002066 | Major facilitator superfamily | 5.4 |  | 508 | Transport |
| NODE_129.g4747.t1 | murein transglycosylase | Glycoside hydrolase, family 61 | 7.8 |  | 349 | Metabolic |
| NODE_130.g16361.t1 | hypothetical protein FPSE_07350 |  | 3.8 |  | 94 |  |
| NODE_131.g16394.t1 | hypothetical protein FOC1_g10013109 |  | 3.3 |  | 421 |  |
| NODE_131.g16399.t1 | hypothetical protein FOXG_13834 | SH3-like domain superfamily | 1.6 |  | 380 | Translation |
| NODE_131.g16425.t1 | hypothetical protein FOTG_03953 | Nucleoporin Nup186/Nup192/Nup205 | 1.0 |  | 726 | Cell organisation |
| NODE_131.g4781.t1 | hypothetical protein FOQG_07675 | FAD-binding 8 | 3.8 |  | 1434 | Oxidation-reduction |
| NODE_132.g16441.t1 | probable eukaryotic release factor 1 | Peptide chain release factor eRF1/aRF1 | 1.5 |  | 1025 | Translation |
| NODE_132.g16460.t1 | 2-methylcitrate synthase, mitochondrial | Citrate synthase active site | 4.0 |  | 472 | Metabolic |
| NODE_132.g16461.t1 | isocitrate lyase | Isocitrate lyase | 1.9 |  | 598 | Metabolic |
| NODE_132.g16462.t1 | glucose 1-dehydrogenase | Enoyl- (Acyl carrier protein) reductase | 1.3 | Cytoplasm | 279 | Metabolic |
| NODE_132.g4803.t1 | hypothetical protein FAVG1_06068 | Peptide chain release factor class I | 1.6 |  | 765 | Translation |
| NODE_133.g4816.t1 | hypothetical protein FOTG_09570 |  | 4.3 |  | 584 |  |
| NODE_133.g4817.t1 | Proline-specific permease | Amino acid permease/ SLC12A domain | 4.4 |  | 536 | Transport |
| NODE_133.g4819.t1 | carboxypeptidase A4 | Peptidase M14, carboxypeptidase A | 4.7 |  | 413 | Proteolysis |
| NODE_134.g16514.t1 | Delta 8-(E)-sphingolipid desaturase | Fatty acid desaturase domain | 2.8 |  | 550 | Metabolic |
| NODE_134.g16551.t1 | related to methionine synthase II (cobalamin-independent) | Cobalamin-independent methionine synthase MetE, C-terminal/archaeal | 2.7 |  | 397 | Biosynthetic |
| NODE_134.g16552.t1 | ABC transporter CDR4 | ABC transporter-like | 4.1 |  | 1507 | Transport |
| NODE_134.g16563.t1 | Putative amidohydrolase ytcJ | Metal-dependent hydrolase, composite domain superfamily | 5.5 |  | 656 |  |
| NODE_134.g4850.t1 | succinate-semialdehyde dehydrogenase (NADP+) | Aldehyde dehydrogenase domain | 2.2 |  | 494 | Oxidation-reduction |
| NODE_134.g4862.t1 | hypothetical protein FOQG_07145 | Tetratricopeptide repeat | 1.9 |  | 1031 | RNA processing |
| NODE_135.g16568.t1 | putative nucleosome assembly protein | Nucleosome assembly protein (NAP) | 2.3 |  | 404 | Nucleosome assembly |
| NODE_135.g16574.t1 | hypothetical protein BFJ68_g11891 | Myo-inositol oxygenase | 3.3 |  | 308 | Oxidation-reduction |
| NODE_136.g16603.t1 | hypothetical protein BFJ69_g5303 | U3 small nucleolar RNA-associated SSU processome protein (Utp12) | 3.6 |  | 391 | rRNA processing |
| NODE_136.g4881.t1 | hypothetical protein FOTG_13992 | Glycoside hydrolase, family 61 | 5.8 |  | 358 | Metabolic |
| NODE_138.g16693.t1 | hypothetical protein FOC4_g10002745 | Glucose-methanol-choline oxidoreductase | 3.7 |  | 617 | Oxidation-reduction |
| NODE_138.g16694.t1 | hypothetical protein BFJ65_g13442 | family decarboxylase | 7.4 |  | 384 |  |
| NODE_138.g16695.t1 | Purine-cytosine permease FCY21 | Purine-cytosine permease | 7.0 |  | 509 | Transport |
| NODE_138.g16699.t1 | hypothetical protein FOXG_16634 | Chloroperoxidase | 6.0 | Extracellular | 231 | Proteolysis |
| NODE_138.g16700.t1 | uricase | Uricase | 3.5 | Cytoplasm | 302 | Oxidation-reduction |
| NODE_138.g4916.t1 | hypothetical protein FOXG_11627 | Brix domain | 3.9 |  | 454 | Ribosomal large subunit assembly |
| NODE_139.g16752.t1 | hypothetical protein FOQG_08021 | Domain of unknown function DUF1747 | 2.5 |  | 518 |  |
| NODE_139.g16777.t1 | hypothetical protein FOPG_10134 | Galactose-binding-like domain superfamily | 2.6 |  | 245 |  |
| NODE_140.g16781.t1 | hypothetical protein FOQG_12476 | HPP | 3.9 |  | 285 |  |
| NODE_140.g4958.t1 | hypothetical protein FOTG_10073 |  | 8.0 | Extracellular | 198 |  |
| NODE_141.g16830.t1 | hypothetical protein FOPG_16855 | Potassium transporter Trk, fungi | 4.1 |  | 658 | Transport |
| NODE_142.g16893.t1 | hypothetical protein BFJ69_g1977 | Cellulose-binding domain | 2.6 | Extracellular | 220 | Metabolic |
| NODE_143.g16928.t1 | Succinyl-CoA:3-ketoacid coenzyme A transferase 1 | Coenzyme A transferase family I | 2.9 |  | 519 | Catabolic |
| NODE_144.g16961.t1 | hypothetical protein BFJ69_g15933, partial | Galactose-binding-like domain superfamily | 8.7 |  | 413 |  |
| NODE_144.g5028.t1 | related to aminopeptidase Y precursor, vacuolar | Peptidase M28, SGAP-like | 6.7 |  | 491 | Proteolysis |
| NODE_145.g16973.t1 | Adenine phosphoribosyltransferase | Adenine phosphoribosyl transferase | 2.1 |  | 222 |  |
| NODE_145.g5051.t1 | 2-methylcitrate dehydratase | 2-methylcitrate dehydratase PrpD | 2.3 |  | 555 | Catabolic |
| NODE_145.g5056.t1 | hypothetical protein FOCG_09020 | RNA recognition motif domain | 2.6 |  | 320 | Cytokinesis |
| NODE_145.g5066.t1 | asparaginyl-tRNA synthetase | Aminoacyl-tRNA synthetase, class II (D/K/N) | 1.4 |  | 564 | Translation |
| NODE_147.g17056.t1 | hypothetical protein FOC1_g10005764 | Clr5 domain | 1.4 |  | 887 |  |
| NODE_148.g17101.t1 | hypothetical protein BFJ68_g4827 | Aminoglycoside phosphotransferase | 5.3 |  | 906 | Metabolic |
| NODE_149.g17123.t1 | related to hexamer-binding protein HEXBP | Zinc finger, CCHC-type superfamily | 3.4 | Cytoplasm | 223 | Transcription |
| NODE_149.g17145.t1 | glucan 1,6-alpha-glucosidase | Glycosyl hydrolase, family 13, catalytic domain | 9.2 |  | 594 | Metabolic |
| NODE_149.g17146.t1 | uncharacterized protein FVRRES_11925 | Importin subunit alpha | 1.4 |  | 552 | Transport |
| NODE_149.g17164.t1 | hypothetical protein FOQG_08041 | Only prolin and serin are matching in the corresponding protein | 1.8 |  | 614 |  |
| NODE_150.g17165.t1 | Inorganic phosphate transporter 1-2 | Major facilitator, sugar transporter-like | 3.6 |  | 670 | Transport |
| NODE_150.g17190.t1 | probable FBP1-fructose-1,6-bisphosphatase | Fructose-1,6-bisphosphatase class 1 | 2.1 |  | 342 | Metabolic |
| NODE_150.g17196.t1 | hypothetical protein FOMG_05784 | WD domain, G-beta repeat | 2.2 |  | 431 | Signal transduction |
| NODE_150.g5150.t1 | hypothetical protein FOPG_02419 |  | 3.0 | Extracellular | 424 |  |
| NODE_150.g5163.t1 | hypothetical protein FOXG_05893 | Major facilitator superfamily | 5.9 |  | 434 | Transport |
| NODE_151.g17205.t1 | hypothetical protein FOCG_12949 | Amino acid transporter, transmembrane domain | 1.3 |  | 459 | Transport |
| NODE_151.g5183.t1 | hypothetical protein FOXG_10350 | WD domain, G-beta repeat | 3.2 |  | 487 | Signal transduction |
| NODE_152.g5190.t1 | saccharopine dehydrogenase | Saccharopine dehydrogenase, NADP binding domain | 1.7 |  | 452 | Oxidation-reduction |
| NODE_153.g17266.t1 | hypothetical protein BFJ69_g12900 | RGS, regulator of G protein signaling domain | 1.3 |  | 147 | Signal transduction |
| NODE_153.g17267.t1 | hypothetical protein BFJ71_g14823 | RGS, regulator of G protein signaling domain | 2.9 |  | 322 | Signal transduction |
| NODE_153.g17268.t1 | hypothetical protein FOMG_10877 | Polyketide synthase, enoylreductase domain | 5.5 |  | 333 | Biosynthetic |
| NODE_153.g5209.t1 | hypothetical protein BFJ69_g2650 | Tetratricopeptide repeat | 2.6 |  | 410 | RNA processing |
| NODE_153.g5210.t1 | hypothetical protein FOC1_g10005150 | Major facilitator superfamily | 4.0 |  | 529 | Transport |
| NODE_154.g17326.t1 | cystathionine beta-lyase | Cys/Met metabolism, pyridoxal phosphate-dependent enzyme | 4.5 |  | 406 | Transsulfuration |
| NODE_154.g17337.t1 | hypothetical protein BFJ65_g10432 | Ribosomal protein L24e | 1.0 |  | 738 | Translation |
| NODE_155.g17355.t1 | Deoxyhypusine hydroxylase | Deoxyhypusine hydroxylase | 2.6 |  | 330 | Peptidyl-lysine modification |
| NODE_155.g5259.t1 | hypothetical protein FOQG_11684 | Nucleolar 27S pre-rRNA processing, Urb2/Npa2, C-terminal | 2.4 |  | 1361 | rRNA processing |
| NODE_155.g5271.t1 | hypothetical protein BFJ69_g6108 | Zinc finger C2H2 superfamily | 1.5 |  | 500 | Transcription |
| NODE_158.g17445.t1 | hypothetical protein FOIG_12077 | CFEM domain | 2.2 |  | 850 |  |
| NODE_158.g17462.t1 | Putative cytochrome b5 | Cytochrome b5-like heme/steroid binding domain | 1.4 |  | 138 | Oxidation-reduction |
| NODE_158.g5312.t1 | hypothetical protein BFJ69_g3115 | WD domain, G-beta repeat | 4.0 |  | 1042 | Signal transduction |
| NODE_159.g17473.t1 | T-complex protein 1 subunit epsilon | Chaperonin Cpn60/TCP-1 family | 1.3 |  | 546 | Stress response |
| NODE_159.g17478.t1 | hypothetical protein FOTG_16825 | FAD linked oxidase, N-terminal | 8.1 |  | 643 | Oxidation-reduction |
| NODE_160.g17529.t1 | hypothetical protein FOXG_15778 |  | 4.6 |  | 594 |  |
| NODE_161.g5351.t1 | hypothetical protein FOC4_g10003839 |  | 7.0 |  | 392 |  |
| NODE_162.g17585.t1 | hypothetical protein FOCG_02516 | WD domain, G-beta repeat | 2.1 |  | 502 | Signal transduction |
| NODE_162.g5386.t1 | Putative endoglucanase type F | Glycoside hydrolase family 10 domain | 7.1 |  | 384 | Metabolic |
| NODE_163.g5399.t1 | Elongation of fatty acids protein sre1 | ELO family | 2.1 |  | 472 |  |
| NODE_163.g5400.t1 | hypothetical protein FOTG_00705 | FYVE zinc finger | 1.1 |  | 352 | Transcription |
| NODE_164.g5410.t1 | hypothetical protein FOC4_g10006219 |  | 4.3 | Extracellular | 220 |  |
| NODE_164.g5411.t1 | hypothetical protein BFJ65_g13039 | Rpa43, N-terminal ribonucleoprotein (RNP) domain | 3.4 |  | 462 |  |
| NODE_164.g5417.t1 | hypothetical protein FAVG1_09906 |  | 2.1 | Extracellular | 81 |  |
| NODE_164.g5419.t1 | nitric oxide dioxygenase | Globin/Protoglobin | 4.8 |  | 415 | Transport |
| NODE_166.g17701.t1 | hypothetical protein FOQG_12836 |  | 8.0 |  | 939 |  |
| NODE_166.g5455.t1 | GAF protein | GAF domain | 3.1 |  | 181 | Transcription |
| NODE_166.g5457.t1 | hypothetical protein FOQG_06594 | WD domain, G-beta repeat | 2.2 |  | 1058 | Signal transduction |
| NODE_167.g5471.t1 | hypothetical protein FOC4_g10007865 |  | 1.3 |  | 574 |  |
| NODE_167.g5484.t1 | hypothetical protein FOQG_07634 | Amino acid permease/ SLC12A domain | 4.3 |  | 546 | Transport |
| NODE_168.g5490.t1 | hypothetical protein BFJ69_g1289 | Homeobox-like domain superfamily | 3.1 |  | 288 |  |
| NODE_168.g5493.t1 | hypothetical protein FPSE_08661 | Ribosomal protein S11 | 2.8 |  | 151 | Translation |
| NODE_168.g5497.t1 | 37S ribosomal protein S10, mitochondrial | Ribosomal protein S10n | 1.5 |  | 390 | Translation |
| NODE_169.g5517.t1 | Low specificity L-threonine aldolase | Aromatic amino acid beta-eliminating lyase/threonine aldolase | 2.4 |  | 353 | Metabolic |
| NODE_170.g17818.t1 | hypothetical protein FOIG_10785 | Protein kinase domain-Histidine kinase | 1.8 |  | 1194 | Signal transduction |
| NODE_170.g17830.t1 | Protein gar2 | RNA recognition motif domain | 4.4 |  | 505 | Cytokinesis |
| NODE_170.g5529.t1 | Zinc transporter ZIP9 | Zinc/iron permease | 1.6 |  | 450 | Transport |
| NODE_170.g5538.t1 | hypothetical protein FOTG_03887 | Nucleolar complex-associated protein 3 | 2.2 |  | 665 | Ribosome biogenesis |
| NODE_172.g17916.t1 | hypothetical protein BFJ69_g4370 | Glycosyl hydrolase family 61 | 5.3 |  | 357 | Metabolic |
| NODE_173.g17931.t1 | hypothetical protein FOTG_05459 |  | 4.8 |  | 487 |  |
| NODE_173.g5609.t1 | hypothetical protein FOXG_16980 | Tyrosinase copper-binding domain | 3.6 |  | 338 | Oxidation-reduction |
| NODE_174.g17958.t1 | retrotransposase | Reverse transcriptase, RNA-dependent DNA polymerase | 2.1 |  | 459 | Biosynthetic |
| NODE_176.g18010.t1 | hypothetical protein FOQG_04872 |  | 1.1 |  | 684 |  |
| NODE_177.g18036.t1 | related to quinate transport protein | Major facilitator, sugar transporter-like | 6.7 |  | 559 | Transport |
| NODE_177.g18058.t1 | catalase-peroxidase | Haem peroxidase | 1.6 |  | 761 | Proteolysis |
| NODE_179.g18095.t1 | hypothetical protein CDV36_014435 | WD domain, G-beta repeat | 6.8 |  | 1392 | Signal transduction |
| NODE_179.g18101.t1 | hypothetical protein FOXG_05017 |  | 2.6 |  | 304 |  |
| NODE_179.g18113.t1 | probable endo-1,4-beta-xylanase A precursor | Glycosyl hydrolase family 11 | 7.1 | Extracellular | 232 | Metabolic |
| NODE_180.g18139.t1 | alcohol oxidase | Glucose-methanol-choline oxidoreductase, N-terminal | 3.9 |  | 557 | Oxidation-reduction |
| NODE_181.g18156.t1 | hypothetical protein FPSE_08287 | Ribosomal protein S24e | 2.0 |  | 136 | Translation |
| NODE_181.g18165.t1 | hypothetical protein FOXG_05607 | alkaline foam protein B precursor | 1.4 | Extracellular | 154 |  |
| NODE_181.g18167.t1 | hypothetical protein FOC1_g10016072 | Glycosyl hydrolase family 63, C-terminal | 2.6 |  | 1012 | Metabolic |
| NODE_181.g5806.t1 | acid beta-fructofuranosidase precursor | Glycosyl hydrolase family 32, C-terminal | 7.8 |  | 600 | Metabolic |
| NODE_182.g18184.t1 | hypothetical protein FOC1_g10006591 | Alkaline-phosphatase-like, core domain superfamily | 5.8 |  | 678 | Protein dephosphorylation |
| NODE_184.g18242.t1 | hypothetical protein FOTG_12582 | Conserved hypothetical protein | 5.4 |  | 267 |  |
| NODE_186.g18274.t1 | Arginine permease | Amino acid permease/ SLC12A domain | 1.7 |  | 515 | Transport |
| NODE_186.g18281.t1 | Cystathionine beta-synthase | Cystathionine beta-synthase | 1.3 |  | 517 | Biosynthetic |
| NODE_186.g18296.t1 | hypothetical protein FOTG_09867 | Mitochondrial import inner membrane translocase subunit Tim44 | 1.2 |  | 553 | Transport |
| NODE_187.g18319.t1 | hypothetical protein FOQG_02431 | P-loop containing nucleoside triphosphate hydrolase | 3.2 |  | 1211 | Phosphorylation |
| NODE_187.g5953.t1 | hypothetical protein FOQG_00208 | rRNA biogenesis protein RRP36 | 1.4 |  | 313 | rRNA biogenesis |
| NODE_188.g18328.t1 | ATP synthase subunit mitochondrial | ATP synthase, F0 complex, subunit C | 1.3 |  | 146 | Transportation |
| NODE_188.g18329.t1 | hypothetical protein FOPG_06725 | HRDC domain | 2.1 |  | 496 |  |
| NODE_188.g18340.t1 | probable NADPH-dependent beta-ketoacyl reductase (rhlG) | Enoyl-(Acyl carrier protein) reductase | 1.3 |  | 287 | Metabolic |
| NODE_188.g18343.t1 | Importin subunit beta 2 | Importin-beta, N-terminal domain | 3.2 |  | 601 | Transport |
| NODE_188.g18354.t1 | hypothetical protein FOXG_09795 | SCP / Tpx-1 / Ag5 / PR-1 / Sc7 family of extracellular domains. | 3.2 |  | 88 |  |
| NODE_189.g18371.t1 | hypothetical protein BFJ69_g1493 | Lytic polysaccharide monooxygenase | 2.9 |  | 418 | Metabolic |
| NODE_189.g5984.t1 | hypothetical protein FOTG_01594 | FAD dependent oxidoreductase | 2.7 |  | 473 | Oxidation-reduction |
| NODE_189.g5988.t1 | hypothetical protein FOQG_01610 | Metal-independent alpha-mannosidase | 3.7 |  | 517 |  |
| NODE_189.g5990.t1 | probable PAB1-mRNA polyadenylate-binding protein | RNA recognition motif domain | 3.0 |  | 750 | Cytokinesis |
| NODE_190.g5997.t1 | Aldehyde reductase 1 | Aldo/keto reductase | 7.3 |  | 338 | Oxidation-reduction |
| NODE_190.g6005.t1 | hypothetical protein FOXG_14666 | Major facilitator, sugar transporter-like | 5.3 |  | 1236 | Transport |
| NODE_191.g18439.t1 | hypothetical protein FOIG_04164 | KR domain | 2.0 |  | 419 |  |
| NODE_192.g18440.t1 | hypothetical protein FOTG_13812 | Major facilitator superfamily | 5.1 |  | 513 | Transport |
| NODE_192.g18466.t1 | 40S ribosomal protein S16 | Ribosomal protein S9 | 2.0 |  | 112 | Translation |
| NODE_192.g18467.t1 | 60S ribosomal protein L32 | Ribosomal protein L32e | 1.8 |  | 130 | Translation |
| NODE_192.g6051.t1 | hypothetical protein FOCG_07260 | Conserved hypothetical protein | 8.0 |  | 368 |  |
| NODE_193.g18475.t1 | hypothetical protein FOXG_19635 | Glucose receptor Git3, N-terminal-G protein-coupled | 3.2 |  | 477 | Signal transduction |
| NODE_194.g18494.t1 | hypothetical protein FOTG_07929 | tRNA methyltransferase, Trm1 | 3.0 |  | 645 | tRNA processing |
| NODE_194.g6096.t1 | UPF0160 protein MYG1 | Metal-dependent protein hydrolase | 1.9 |  | 349 |  |
| NODE_194.g6098.t1 | Glutamate--cysteine ligase | Glutamate-cysteine ligase catalytic subunit | 1.6 |  | 716 | Biosynthetic |
| NODE_194.g6099.t1 | hypothetical protein FOMG_06736 | Zinc finger, GATA-type | 1.6 |  | 421 | Transcription |
| NODE_196.g6109.t1 | DNA-directed RNA polymerase I subunit RPA2 | DNA-directed RNA polymerase, subunit 2, hybrid-binding domain | 3.7 |  | 1232 | Transcription |
| NODE_197.g6125.t1 | hypothetical protein FOMG_14709 | Glucose receptor Git3, N-terminal-G protein-coupled | 4.7 |  | 479 | Signal transduction |
| NODE_200.g18648.t1 | hypothetical protein FPSE_10374 | Ribosomal protein L5 | 2.9 |  | 173 | Translation |
| NODE_200.g6184.t1 | hypothetical protein FOQG_09510 | CCAAT-binding factor | 2.7 |  | 549 |  |
| NODE_200.g6185.t1 | F-type H+-transporting ATPase subunit B | ATP synthase, F0 complex, subunit B/MI25 | 1.1 |  | 242 | Transportation |
| NODE_202.g18683.t1 | hypothetical protein FOQG_12048 | Bromodomain | 1.1 |  | 872 | Metabolic |
| NODE_202.g18698.t1 | hypothetical protein FPSE_06055 | Ribosomal protein S27a | 2.2 |  | 154 | Translation |
| NODE_202.g18699.t1 | hypothetical protein FPSE_06056 | Ribosomal protein S26e | 1.9 |  | 117 | Translation |
| NODE_202.g6219.t1 | hypothetical protein FOTG_06875 | P-loop containing nucleoside triphosphate hydrolase | 1.7 |  | 509 | Phosphorylation |
| NODE_202.g6234.t1 | hypothetical protein FOQG_05285 | DRG Family Regulatory Proteins, Tma46 (GTP binding) | 1.5 |  | 358 | Transcription |
| NODE_204.g18741.t1 | hypothetical protein FOXG_00622 | ATP synthase, F0 complex, subunit E, mitochondrial | 1.0 | Mitochondrion | 90 | Transportation |
| NODE_204.g6262.t1 | probable MRT4-mRNA turnover 4 | Ribosome assembly factor Mrt4 | 4.1 |  | 244 | Ribosomal large subunit assembly |
| NODE_204.g6275.t1 | hypothetical protein BFJ69_g6125 |  | 2.5 |  | 120 |  |
| NODE_206.g18781.t1 | Putative CDP-alcohol phosphatidyltransferase class-I family protein C22A12.08c | phosphatidyl synthase | 2.4 |  | 112 | Biosynthetic |
| NODE_206.g18782.t1 | Aldose reductase A | Aldo/keto reductase | 1.2 |  | 329 | Oxidation-reduction |
| NODE_206.g6300.t1 | hypothetical protein BFJ69_g8254 | Nitroreductase Frm2/Hbn1-like | 1.7 | Mitochondrion | 205 | Stress response |
| NODE_206.g6305.t1 | probable fibrillarin (NOP1) | Fibrillarin | 3.5 |  | 302 | rRNA processing |
| NODE_207.g18801.t1 | hypothetical protein BFJ71_g4374 | RNA-binding S4 domain | 1.1 |  | 528 |  |
| NODE_208.g18830.t1 | hypothetical protein FOXG_13238 | Conserved hypothetical protein | 6.2 |  | 340 |  |
| NODE_208.g6359.t1 | hypothetical protein FOQG_06658 | F-box-like domain superfamily | 1.7 |  | 306 |  |
| NODE_209.g18847.t1 | hypothetical protein FOMG_16390 | Transcriptional regulator PAI 2-type | 6.1 |  | 263 | Transcription |
| NODE_210.g18883.t1 | hypothetical protein FOTG_05938 | Helicase-associated domain | 1.9 |  | 779 | Chromatin organization |
| NODE_211.g18893.t1 | hypothetical protein FOC1_g10012848 | Zn(2)-C6 fungal-type DNA-binding domain-GAL4 | 3.1 |  | 1104 | Transcription |
| NODE_212.g6412.t1 | hypothetical protein BFJ69_g12816 | Peptidase C19, ubiquitin carboxyl-terminal hydrolase | 1.4 |  | 1662 | Proteolysis |
| NODE_212.g6413.t1 | hypothetical protein BFJ69_g12817 | 3'5'-cyclic nucleotide phosphodiesterase, catalytic domain | 1.4 |  | 876 | Signal transduction |
| NODE_212.g6416.t1 | hypothetical protein FOC1_g10013863 | Conserved hypothetical protein | 2.7 |  | 349 |  |
| NODE_212.g6423.t1 | hypothetical protein FAVG1_05598 | Ribosomal protein L1/ribosomal biogenesis protein | 2.1 | Cytoplasm | 217 | Translation |
| NODE_213.g18917.t1 | hypothetical protein FSPOR_8584 | Zinc finger C2H2 superfamily | 1.6 |  | 436 | Transcription |
| NODE_213.g6426.t1 | hypothetical protein FOC1_g10011108 | Arrestin, C-terminal | 1.2 |  | 488 |  |
| NODE_214.g18932.t1 | hypothetical protein FOC1_g10002770 |  | 1.6 | Extracellular | 203 |  |
| NODE_217.g6515.t1 | translation factor GUF1, mitochondrial | GTP-binding domain | 1.0 |  | 774 | Transcription |
| NODE_218.g19020.t1 | isopentenyl-diphosphate delta-isomerase | Isopentenyl-diphosphate delta-isomerase, type 1 | 2.1 |  | 253 |  |
| NODE_220.g19055.t1 | hypothetical protein FOQG_17375 |  | 6.9 | Extracellular | 123 |  |
| NODE_224.g19101.t1 | hypothetical protein FOXG_10013 |  | 1.2 |  | 370 |  |
| NODE_224.g6617.t1 | probable translation initiation factor eIF-4E | Translation Initiation factor eIF- 4e | 1.9 |  | 256 | Translation |
| NODE_226.g19140.t1 | related to endopeptidase K | Peptidase S8/S53 domain | 4.2 |  | 293 | Proteolysis |
| NODE_226.g6658.t1 | hypothetical protein BFJ69_g10634 | integral membrane family protein | 5.0 |  | 364 |  |
| NODE_226.g6659.t1 | hypothetical protein FOC4_g10006682 | Tyrosinase copper-binding domain | 6.7 |  | 403 | Oxidation-reduction |
| NODE_226.g6667.t1 | hypothetical protein BFJ69_g10657 |  | 6.3 |  | 446 |  |
| NODE_228.g19190.t1 | hypothetical protein BFJ65_g16851 | integral membrane protein | 3.0 |  | 321 |  |
| NODE_229.g19217.t1 | hypothetical protein FOXG_15106 | bZIP_YAP | 6.3 |  | 299 | Transcription |
| NODE_229.g6704.t1 | E3 ubiquitin-protein ligase BRE1 | BRE1 E3 ubiquitin ligase | 1.0 |  | 645 | Chromatin organization |
| NODE_229.g6710.t1 | hypothetical protein FOC1_g10013697 | U3 snoRNA associated | 2.3 |  | 275 | rRNA processing |
| NODE_229.g6711.t1 | hypothetical protein FOC1_g10013696 | von Willebrand factor, type A | 2.2 |  | 1134 |  |
| NODE_229.g6716.t1 | hypothetical protein FPSE_09105 | Ribosomal protein L37ae | 3.8 |  | 92 | Translation |
| NODE_229.g6718.t1 | hypothetical protein FOTG_00862 | Ribosomal protein L46, N-terminal | 1.3 |  | 343 | Translation |
| NODE_230.g19222.t1 | hypothetical protein FOTG_04238 | WD domain, G-beta repeat | 4.1 |  | 534 | Signal transduction |
| NODE_230.g19231.t1 | MFS transporter, FHS family, L-fucose permease | Major facilitator superfamily | 8.2 |  | 461 | Transport |
| NODE_233.g19282.t1 | hypothetical protein FOQG_10905 |  | 6.1 |  | 304 |  |
| NODE_233.g6782.t1 | hypothetical protein FOQG_04914 | NUC173 domain | 4.1 |  | 1240 | rRNA processing |
| NODE_234.g19293.t1 | hypothetical protein BFJ69_g11807 | Mitochondrial biogenesis protein AIM24 | 2.5 |  | 241 | Biogenesis protein |
| NODE_234.g19299.t1 | uncharacterized protein FMAN_13153 | LysM domain superfamily | 7.1 |  | 275 |  |
| NODE_234.g6796.t1 | Pyridoxal biosynthesis protein PDX2 | Pyridoxal 5'-phosphate synthase subunit PdxT/SNO-GATase1_PB | 2.8 | Cytoplasm | 238 | Biosynthetic |
| NODE_234.g6801.t1 | hypothetical protein BFJ69_g5447 | Alpha/Beta hydrolase fold | 2.5 |  | 297 | Metabolic |
| NODE_234.g6811.t1 | hypothetical protein FOXG_08668 | Major facilitator, sugar transporter-like | 3.1 |  | 536 | Transport |
| NODE_235.g6814.t1 | hypothetical protein FOQG_03929 | Calcium-dependent channel, 7TM region, putative phosphate | 2.8 |  | 896 | Transport |
| NODE_236.g19322.t1 | probable asparagine synthase | Asparagine synthase, glutamine-hydrolyzing | 1.2 |  | 570 | Biosynthetic |
| NODE_237.g19344.t1 | hypothetical protein FOQG_05189 |  | 1.5 |  | 354 |  |
| NODE_237.g19345.t1 | Protein SOK1 | T-complex 11 | 1.9 |  | 401 |  |
| NODE_239.g19391.t1 | Frequency clock protein | Frequency clock protein | 2.4 |  | 837 | Transcription |
| NODE_239.g6910.t1 | probable RPL34B-ribosomal protein L34.e | Ribosomal protein L34Ae | 2.7 |  | 116 | Translation |
| NODE_239.g6920.t1 | hypothetical protein FOIG_04191 | integral membrane protein | 2.0 |  | 381 |  |
| NODE_241.g19414.t1 | hypothetical protein FOXG_09275 | Dimeric alpha-beta barrel | 3.9 |  | 219 |  |
| NODE_241.g19415.t1 | Eukaryotic translation initiation factor 3 subunit H | Eukaryotic translation initiation factor 3 subunit H | 2.0 |  | 364 | Translation |
| NODE_241.g19416.t1 | hypothetical protein FPOA_07235 | Adenosylhomocysteinase-like | 2.0 |  | 449 | Metabolic |
| NODE_242.g19429.t1 | 4-coumarate--CoA ligase-like 7 | AMP-dependent synthetase/ligase | 3.8 |  | 576 |  |
| NODE_242.g6963.t1 | Hexose transporter HXT15 | Major facilitator, sugar transporter-like | 4.9 |  | 535 | Transport |
| NODE_243.g19451.t1 | hypothetical protein FOQG_16413 | Ferric reductase, NAD binding domain | 4.1 |  | 622 | Oxidation-reduction |
| NODE_243.g6981.t1 | hypothetical protein FOQG_07576 |  | 2.1 |  | 261 |  |
| NODE_246.g7031.t1 | DnaJ like subfamily A member 2 | Heat shock protein DnaJ, cysteine-rich domain | 3.2 |  | 434 | Stress response |
| NODE_247.g19509.t1 | hypothetical protein BFJ70_g8696 | Thiamine pyrophosphate enzyme, central domain | 4.7 |  | 516 |  |
| NODE_248.g19522.t1 | hypothetical protein FPSE_11676 | At2g23090-like, zinc-binding domain | 2.2 | Nucleus | 74 |  |
| NODE_248.g7057.t1 | Ankyrin repeat-containing protein YAR1 | Ankyrin repeat-containing domain | 3.6 |  | 187 | Metabolic |
| NODE_249.g19530.t1 | hypothetical protein FOMG_08164 | Sas10/Utp3/C1D | 2.9 |  | 616 |  |
| NODE_249.g19533.t1 | hypothetical protein BFJ69_g17925 | Ribonuclease H-like superfamily | 4.8 |  | 709 | RNA processing |
| NODE_249.g19535.t1 | hypothetical protein FOC4_g10013387 | Nucleotide-diphospho-sugar transferase | 1.1 |  | 398 | Biosynthetic |
| NODE_250.g19542.t1 | hypothetical protein FOQG_12469 | Glycosyl hydrolase, family 88 | 6.5 |  | 381 | Metabolic |
| NODE_250.g7073.t1 | hypothetical protein FOIG_06211 | Zn (2)-C6 fungal-type DNA-binding domain-GAL4 | 1.3 |  | 879 | Transcription |
| NODE_253.g19575.t1 | hypothetical protein FOCG_13174 | Glycoside hydrolase, family 61 | 3.3 | Extracellular | 242 | Metabolic |
| NODE_253.g7100.t1 | probable methylmalonate-semialdehyde dehydrogenase (acylating) | Aldehyde dehydrogenase domain | 1.7 |  | 570 | Oxidation-reduction |
| NODE_253.g7105.t1 | hypothetical protein FOQG_09647 | WD domain, G-beta repeat | 2.6 |  | 581 | Signal transduction |
| NODE_254.g19598.t1 | hypothetical protein BFJ70_g17546, partial | Tetratricopeptide repeat | 5.5 |  | 849 | RNA processing |
| NODE_255.g19603.t1 | hypothetical protein BFJ69_g12668 | Carbon-nitrogen hydrolase | 6.0 |  | 362 | Metabolic |
| NODE_256.g19614.t1 | hypothetical protein FOQG_04873 | Ankyrin repeat-containing domain | 4.5 |  | 629 | Metabolic |
| NODE_257.g19635.t1 | hypothetical protein FOPG_07197 | AARP2CN | 2.2 |  | 833 | Ribosome biogenesis |
| NODE_261.g19689.t1 | hypothetical protein FOXG_04951 | NADH:flavin oxidoreductase/NADH oxidase, N-terminal | 4.2 |  | 409 | Oxidation-reduction |
| NODE_262.g19697.t1 | hypothetical protein FOXG_07455 | Major facilitator superfamily | 4.4 |  | 510 | Transport |
| NODE_262.g19703.t1 | L-galactonate dehydratase | Enolase-like, C-terminal domain superfamily | 3.8 |  | 450 |  |
| NODE_263.g7286.t1 | hypothetical protein FOC1_g10014608 | Ribosomal protein L34 | 2.1 |  | 125 | Translation |
| NODE_267.g7355.t1 | hypothetical protein FOQG_12519 |  | 6.6 |  | 269 |  |
| NODE_268.g19757.t1 | Ribonucleoside-diphosphate reductase large chain | Ribonucleotide reductase large subunit, C-terminal | 1.2 |  | 713 | Oxidation-reduction |
| NODE_268.g7374.t1 | hypothetical protein FOTG_04049 |  | 3.5 |  | 197 |  |
| NODE_268.g7375.t1 | cyclopropane-fatty-acyl-phospholipid synthase | Mycolic acid cyclopropane synthase | 2.6 |  | 521 | Biosynthetic |
| NODE_269.g19771.t1 | hypothetical protein BFJ68_g17572, partial | NACHT nucleoside triphosphatase | 2.2 |  | 683 | Metabolic |
| NODE_269.g7384.t1 | sodium/potassium-transporting ATPase subunit alpha | P-type ATPase | 2.1 |  | 1099 | Transport |
| NODE_269.g7396.t1 | hypothetical protein FOQG_10533 |  | 3.6 |  | 304 |  |
| NODE_270.g19777.t1 | allantoinase | Amidohydrolase-related | 2.4 |  | 254 | Proteolysis |
| NODE_270.g19778.t1 | Putative hexaprenyl pyrophosphate synthase, mitochondrial | Polyprenyl synthetase | 2.4 |  | 455 | Biosynthetic |
| NODE_270.g7407.t1 | hypothetical protein FOC1_g10008475 | Importin-beta, N-terminal domain | 1.9 |  | 1078 | Transport |
| NODE_271.g7418.t1 | PiT family inorganic phosphate transporter | Phosphate transporter | 7.8 |  | 607 | Transport |
| NODE_272.g7433.t1 | hypothetical protein FOC4_g10009928 |  | 6.0 |  | 187 |  |
| NODE_273.g19811.t1 | Tyrosine--tRNA ligase | Aminoacyl-tRNA synthetase, class Ic | 1.2 |  | 521 | Translation |
| NODE_273.g7454.t1 | hypothetical protein FPSE_01306 | Ribosomal protein S12/S23 | 2.5 | Cytoplasm | 145 | Translation |
| NODE_274.g7468.t1 | hypothetical protein FOQG_12056 | PAN/Apple domain | 3.0 |  | 850 |  |
| NODE_274.g7470.t1 | Argininosuccinate synthase | Argininosuccinate synthase | 2.8 |  | 431 | Biosynthetic |
| NODE_274.g7472.t1 | hypothetical protein FOQG_12062 | Pectinesterase, catalytic | ### |  | 2622 | Metabolic |
| NODE_274.g7477.t1 | hypothetical protein FOQG_12067 | Haem peroxidase | 3.1 |  | 530 | Proteolysis |
| NODE_276.g19833.t1 | hypothetical protein BFJ65_g5089 | Pseudouridine synthase II, N-terminal | 3.1 |  | 249 | RNA modification |
| NODE_277.g19843.t1 | hypothetical protein FOC1_g10007236 | RNA recognition motif domain | 3.8 |  | 369 | Cytokinesis |
| NODE_277.g19848.t1 | hypothetical protein BFJ68_g12772 | Conserved hypothetical protein | 4.6 |  | 364 |  |
| NODE_279.g19872.t1 | hypothetical protein FOC4_g10009741 | Major facilitator superfamily | 1.7 |  | 602 | Transport |
| NODE_279.g7564.t1 | hypothetical protein FOTG_04522 | Major facilitator superfamily | 1.6 |  | 497 | Transport |
| NODE_280.g7593.t1 | aminodeoxychorismate synthase | ADC synthase | 2.7 |  | 830 | Biosynthetic |
| NODE_280.g7595.t1 | Protein mak16 | Mak16 protein | 2.8 |  | 312 |  |
| NODE_281.g7597.t1 | probable pectin lyase precursor | Pectate lyase | 5.5 |  | 377 | Metabolic |
| NODE_282.g7614.t1 | hypothetical protein FOC4_g10004692 | Major facilitator superfamily | 2.4 |  | 505 | Transport |
| NODE_282.g7615.t1 | hypothetical protein FVEG_11599 | Polyketide synthase, enoylreductase domain | 5.5 |  | 349 | Biosynthetic |
| NODE_284.g19913.t1 | hypothetical protein FOIG_03484 | Zn (2)-C6 fungal-type DNA-binding domain-GAL4 | 1.2 |  | 854 | Transcription |
| NODE_284.g7636.t1 | BUD22 family protein C4F10.06 | Bud22 domain | 3.8 |  | 466 | Ribosome biogenesis |
| NODE_284.g7640.t1 | hypothetical protein FOXG_03441 | bZIP_YAP | 3.1 |  | 536 | Transcription |
| NODE_284.g7647.t1 | hypothetical protein FOC1_g10006829 | RNA-binding domain superfamily | 1.7 |  | 552 |  |
| NODE_285.g7654.t1 | hypothetical protein BFJ69_g6102 | Tetratricopeptide repeat | 1.3 |  | 786 | RNA processing |
| NODE_286.g7672.t1 | hypothetical protein FOC1_g10016026 | DNA-directed RNA polymerase I, subunit RPA34.5 | 3.3 |  | 664 | Transcription |
| NODE_287.g7680.t1 | hypothetical protein FOC4_g10001177 | Short-chain dehydrogenase/reductase SDR | 3.5 |  | 283 | Oxidation-reduction |
| NODE_287.g7685.t1 | hydrophobin | Cerato-ulmin hydrophobin family | 5.6 | Extracellular | 98 |  |
| NODE_287.g7687.t1 | hypothetical protein FOCG_11701 | TauD/TfdA-like domain | 7.5 |  | 644 | Oxidation-reduction |
| NODE_288.g7705.t1 | hypothetical protein FOIG_11752 | Major facilitator, sugar transporter-like | 4.7 |  | 556 | Transport |
| NODE_289.g7708.t1 | 3-hydroxybenzoate 6-hydroxylase 1 | FAD-binding domain | 8.4 |  | 452 |  |
| NODE_289.g7710.t1 | 2,3-dihydroxybenzoate decarboxylase | Amidohydrolase-related | 8.6 |  | 336 | Proteolysis |
| NODE_289.g7713.t1 | hypothetical protein FOXG_08912 | Amino acid permease/ SLC12A domain | 9.1 |  | 540 | Transport |
| NODE_290.g19966.t1 | Sugar transporter STL1 | Major facilitator, sugar transporter-like | 3.6 |  | 540 | Transport |
| NODE_291.g19975.t1 | hypothetical protein BFJ66_g3979 | GLEYA adhesin domain | 4.6 |  | 309 |  |
| NODE_291.g7754.t1 | probable isocitrate dehydrogenase | Isopropylmalate dehydrogenase-like domain | 2.7 |  | 378 | Oxidation-reduction |
| NODE_292.g7759.t1 | hypothetical protein FOPG_10435 | PAN/Apple domain | 4.7 |  | 355 |  |
| NODE_294.g7818.t1 | hypothetical protein FOXG_06137 | Putative RNA methyltransferase | 1.8 |  | 340 | RNA methylation |
| NODE_294.g7821.t1 | uncharacterized protein FPRN_03082 |  | 6.1 |  | 129 |  |
| NODE_296.g20024.t1 | hypothetical protein FOXG_12365 |  | 1.3 |  | 494 |  |
| NODE_300.g20056.t1 | Retrovirus-related Pol polyprotein from transposon TNT 1-94 | Integrase, catalytic core | 3.4 |  | 581 | DNA integration |
| NODE_300.g7946.t1 | hypothetical protein FOXG_04143 | Polyketide synthase, enoylreductase domain | 4.5 |  | 331 | Biosynthetic |
| NODE_302.g7973.t1 | GMP synthase | GMP synthase, glutamine amidotransferase | 4.6 |  | 286 | Biosynthetic |
| NODE_302.g7983.t1 | hypothetical protein FOIG_02033 | Stress responsive alpha-beta barrel | 3.2 |  | 115 | Stress response |
| NODE_303.g20086.t1 | hypothetical protein FOQG_03978 | SNF2-related, N-terminal domain | 3.4 |  | 585 |  |
| NODE_303.g7995.t1 | glycine dehydrogenase | Glycine cleavage system P protein | 1.2 |  | 1052 | Oxidation-reduction |
| NODE_303.g7996.t1 | probable GCV3-glycine decarboxylase, subunit H | Glycine cleavage system H-protein/Simiate | 1.2 |  | 172 | Glycine decarboxylation |
| NODE_304.g20094.t1 | hypothetical protein BFJ65_g14400 | protein tyrosine phosphatase activity | 5.9 |  | 201 |  |
| NODE_305.g8012.t1 | UPF0202 protein KRE33 | Acetyltransferase (GNAT) domain | 3.3 |  | 1066 | Biosynthetic |
| NODE_306.g20105.t1 | mannosyl-oligosaccharide alpha-1,2-mannosidase | glycosyl hydrolase 47 family | 3.3 |  | 188 | Metabolic |
| NODE_306.g20106.t1 | hypothetical protein FLAG1_08396 |  | 1.2 |  | 233 |  |
| NODE_306.g8027.t1 | Iron transport multicopper oxidase FET3 | Multicopper oxidase, type 2 | 3.2 |  | 623 | Oxidation-reduction |
| NODE_311.g20145.t1 | hypothetical protein BFJ69_g5935 |  | 1.4 |  | 127 |  |
| NODE_311.g20151.t1 | hypothetical protein FOTG_11818 | Tetratricopeptide repeat | 1.9 |  | 747 | RNA processing |
| NODE_311.g8104.t1 | related to 26S proteasome subunit RPN4 | Zinc finger C2H2-type | 3.6 |  | 622 | Transcription |
| NODE_312.g8119.t1 | hypothetical protein FOQG_04467 |  | 1.3 |  | 1130 |  |
| NODE_316.g20173.t1 | S-adenosylmethionine synthase | S-adenosylmethionine synthetase, N-terminal | 2.7 |  | 375 | Biosynthetic |
| NODE_317.g8187.t1 | hypothetical protein BFJ69_g2675 | U3 small nucleolar RNA-associated SSU processome protein (Utp14 protein) | 2.9 |  | 881 | rRNA processing |
| NODE_317.g8197.t1 | hypothetical protein FOQG_01843 | Zn (2)-C6 fungal-type DNA-binding domain | 1.2 |  | 825 | Transcription |
| NODE_320.g20200.t1 | hypothetical protein FOPG_04550 | Lysine methyltransferase | 3.4 |  | 286 | Methylation |
| NODE_320.g20202.t1 | hypothetical protein BFJ69_g6249 | Telomere repeat-binding factor, dimerisation domain | 2.0 |  | 699 |  |
| NODE_320.g8235.t1 | NADH-ubiquinone oxidoreductase 19.3 kDa subunit, mitochondrial | NADH-ubiquinone oxidoreductase, 20 Kd subunit | 1.0 | Mitochondrion | 222 | Oxidation-reduction |
| NODE_321.g20210.t1 | hypothetical protein FOTG_17576 | family decarboxylase | 1.0 |  | 333 |  |
| NODE_321.g8245.t1 | Putative exoglucanase type C | Glycosyl hydrolase family 7 | 1.7 |  | 460 | Metabolic |
| NODE_321.g8251.t1 | hypothetical protein FOQG_08558 | Armadillo-type fold | 2.9 |  | 2647 |  |
| NODE_321.g8254.t1 | hypothetical protein BFJ69_g3711 | DNA polymerase V/Myb-binding protein 1A | 3.5 |  | 1008 | Transcription |
| NODE_323.g8274.t1 | glycyl-tRNA synthetase | Aminoacyl-tRNA synthetase, class II (G/ P/ S/T) | 1.3 |  | 663 | Translation |
| NODE_324.g8283.t1 | hypothetical protein FOIG_01343 | Ribosomal protein L15e | 2.8 |  | 474 | Translation |
| NODE_324.g8286.t1 | hypothetical protein FOTG_06305 | WD domain, G-beta repeat | 3.2 |  | 1077 | Signal transduction |
| NODE_326.g20240.t1 | hypothetical protein BFJ69_g856 | Domain of unknown function (DUF1708) | 3.2 |  | 468 |  |
| NODE_326.g8313.t1 | hypothetical protein BFJ70_g11906 | carbohydrate-binding module family 19 protein | 2.4 |  | 1004 | Metabolic |
| NODE_328.g8342.t1 | uncharacterized protein FFUJ_08611 | Sulfotransferase family | 4.7 |  | 303 |  |
| NODE_328.g8343.t1 | hypothetical protein BFJ65_g11198 | Mitochondrial homologous recombination protein 1 | 1.4 |  | 255 | Transcription |
| NODE_329.g8357.t1 | 40S ribosomal protein S30-B | Ribosomal protein S30 | 1.1 |  | 62 | Translation |
| NODE_330.g8363.t1 | hypothetical protein BFJ65_g7877 | Glycosyl hydrolase family 61 | 5.6 |  | 339 | Metabolic |
| NODE_333.g8417.t1 | hypothetical protein FOXG_11073 | R3H domain | 2.4 |  | 624 |  |
| NODE_335.g8449.t1 | 23S rRNA (-2'-O)-methyltransferase | Ribosomal RNA methyltransferase FtsJ domain | 3.6 |  | 375 | Methylation |
| NODE_336.g8454.t1 | hypothetical protein FOTG_09173 | Cytidine and deoxycytidylate deaminase domain | 2.4 |  | 512 |  |
| NODE_337.g20294.t1 | Fatty acid synthase subunit beta | Fatty acid synthase | 2.0 |  | 679 | Oxidation-reduction |
| NODE_337.g8466.t1 | probable EFB1-translation elongation factor eEF1beta | Translation elongation factor EF1B, beta/delta chains, conserved site | 2.3 |  | 231 | Translation |
| NODE_345.g8595.t1 | FAD-containing monooxygenase EthA | Flavin monooxygenase-like | 3.4 |  | 492 | Oxidation-reduction |
| NODE_349.g8634.t1 | Efflux pump roqT | Major facilitator superfamily | 4.9 |  | 581 | Transport |
| NODE_349.g8644.t1 | hypothetical protein FOMG_11112 | Glycosyl hydrolase family 81, N-terminal | 2.2 |  | 854 | Metabolic |
| NODE_350.g8645.t1 | 2-oxoisovalerate dehydrogenase E2 component (dihydrolipoyl transacylase) | 2-oxoacid dehydrogenase acyltransferase, catalytic domain | 3.1 |  | 488 |  |
| NODE_350.g8653.t1 | hypothetical protein FOC4_g10015188 | Protein of unknown function DUF3712 | 8.7 |  | 343 |  |
| NODE_354.g20360.t1 | hypothetical protein BFJ65_g4294 |  | 6.5 |  | 549 |  |
| NODE_357.g20373.t1 | membrane primary amine oxidase | Integrase, catalytic core | 1.9 |  | 581 | DNA integration |
| NODE_357.g8742.t1 | related to proline oxidase | Proline dehydrogenase | 2.5 |  | 482 | Oxidation-reduction |
| NODE_357.g8744.t1 | hypothetical protein FOCG_01966 |  | 1.5 |  | 734 |  |
| NODE_358.g8756.t1 | Fe-Mn family superoxide dismutase | Manganese/iron superoxide dismutase, C-terminal | 1.3 |  | 279 |  |
| NODE_358.g8757.t1 | hypothetical protein FOQG_00510 |  | 3.1 |  | 828 |  |
| NODE_361.g20392.t1 | hypothetical protein FOC1_g10008740 | Domain of unknown function DUF2431 | 1.6 |  | 281 |  |
| NODE_363.g8840.t1 | probable bifunctional D12/D15 fatty acid desaturase | Fatty acid desaturase domain | 9.1 |  | 402 | Metabolic |
| NODE_363.g8842.t1 | Putative xyloglucan-specific endo-beta-1,4-glucanase A | Glycosyl hydrolase family 12 | 1.5 |  | 328 | Metabolic |
| NODE_363.g8847.t1 | CTP synthase | CTP synthase, N-terminal | 2.5 |  | 580 | Biosynthetic |
| NODE_364.g8860.t1 | L-asparaginase | L-asparaginase II | 4.0 |  | 360 | Metabolic |
| NODE_365.g20405.t1 | hypothetical protein BFJ67_g12378 |  | 2.0 |  | 499 |  |
| NODE_366.g8894.t1 | Maintenance of ploidy protein mob2 | MOB kinase activator family | 1.1 |  | 379 | Signal transduction |
| NODE_368.g8918.t1 | hypothetical protein FOQG_10513 | Chromo/chromo shadow domain | 1.1 |  | 374 | Chromatin organisation |
| NODE_368.g8927.t1 | hypothetical protein FOQG_10523 | Tetratricopeptide repeat | 4.9 |  | 2129 | RNA processing |
| NODE_368.g8928.t1 | hypothetical protein FOC4_g10006280 |  | 4.7 |  | 703 |  |
| NODE_368.g8929.t1 | hypothetical protein FOQG_10525 |  | 4.8 |  | 1392 |  |
| NODE_372.g20427.t1 | hypothetical protein BFJ70_g2338 | Class I glutamine amidotransferase-like | 1.2 |  | 226 | Metabolic |
| NODE_374.g8999.t1 | hypothetical protein FOXG_09698 | Catechol dioxygenase, N-terminal | 2.4 |  | 300 | Oxidation-reduction |
| NODE_374.g9000.t1 | hypothetical protein BFJ69_g6212 | CoA-transferase family III | 3.0 |  | 565 | Catabolic |
| NODE_377.g9040.t1 | glycogen | Glycogen synthase | 2.1 |  | 705 | Biosynthetic |
| NODE_379.g9063.t1 | hypothetical protein FOTG_04141 | Nucleoporin, Nup133/Nup155-like, N-terminal | 1.3 |  | 1403 | Cell organisation |
| NODE_379.g9073.t1 | Bifunctional xylanase/deacetylase | Polysaccharide deacetylase | 7.7 |  | 274 | Metabolic |
| NODE_379.g9074.t1 | hypothetical protein FOPG_03948 | Zn (2)-C6 fungal-type DNA-binding domain | 1.5 |  | 591 | Transcription |
| NODE_380.g9078.t1 | hypothetical protein FOC1_g10004101 |  | 3.1 |  | 476 |  |
| NODE_380.g9080.t1 | GTP-binding protein YchF | GTP binding domain | 6.2 |  | 401 | Signal transduction |
| NODE_380.g9081.t1 | hypothetical protein BFJ69_g5585 |  | 4.3 |  | 314 |  |
| NODE_381.g9097.t1 | hypothetical protein FOTG_05490 | Velvet factor | 1.2 |  | 532 | Transcription |
| NODE_382.g9107.t1 | 2-isopropylmalate synthase | 2-isopropylmalate synthase LeuA, allosteric (dimerisation) domain | 1.7 |  | 616 | Biosynthetic |
| NODE_384.g9131.t1 | hypothetical protein BFJ69_g3127 | Transcription factor domain, fungi | 1.6 |  | 971 | Transcription |
| NODE_384.g9132.t1 | hypothetical protein FOIG_08103 | Ribosomal protein L47, mitochondrial | 1.3 |  | 255 | Translation |
| NODE_384.g9134.t1 | nucleolar GTP-binding protein | Nucleolar GTP-binding protein 1, Rossman-fold domain | 3.1 |  | 659 | Signal Transduction |
| NODE_384.g9135.t1 | orotate phosphoribosyltransferase | Phosphoribosyltransferase domain | 1.2 | Cytoplasm | 234 | Metabolic |
| NODE_386.g9158.t1 | acetoacetate-CoA ligase | Acetoacetyl-CoA synthase | 1.6 |  | 696 | Metabolic |
| NODE_388.g9174.t1 | hypothetical protein BFJ69_g6089 | GPI anchored serine-rich protein | 1.5 |  | 234 | Proteolysis |
| NODE_388.g9177.t1 | hypothetical protein FOQG_11640 | Pre-rRNA-processing protein RIX1, N-terminal | 2.3 |  | 759 | Ribosome biogenesis |
| NODE_388.g9180.t1 | MEAB protein | bZIP_YAP | 1.1 |  | 410 | Transcription |
| NODE_388.g9183.t1 | hypothetical protein BFJ69_g6142 | Stm1-like, N-terminal | 1.5 |  | 316 | Stress response |
| NODE_393.g9249.t1 | Protein BCP1 | BCP1 family | 1.5 |  | 285 |  |
| NODE_394.g9258.t1 | spermidine synthase | Spermidine/spermine synthases | 1.6 |  | 294 | Metabolic |
| NODE_394.g9261.t1 | hypothetical protein FPSE_03626 | Ribosomal protein L10e/L16 | 1.3 |  | 221 | Translation |
| NODE_395.g9269.t1 | Eukaryotic translation initiation factor 5B | GTP-binding domain | 1.5 |  | 1054 | Transcription |
| NODE_395.g9273.t1 | hypothetical protein BFJ70_g12854 | Translation elongation factor, IF5A C-terminal | 1.9 |  | 164 | Translation |
| NODE_395.g9274.t1 | hypothetical protein BFJ69_g3224 | Ribosomal protein L40e | 2.1 |  | 619 | Translation |
| NODE_401.g9353.t1 | hypothetical protein FOC4_g10011866 | Ribosomal protein L25/L23 | 1.5 |  | 202 | Translation |
| NODE_401.g9356.t1 | Eukaryotic translation initiation factor 2 subunit alpha | Translation initiation factor 2, alpha subunit | 1.9 |  | 327 | Translation |
| NODE_404.g9388.t1 | hypothetical protein FOCG_00634 | Protein of unknown function DUF4602 | 1.6 |  | 317 |  |
| NODE_405.g9406.t1 | hypothetical protein FOXG_01695 | Ribosomal biogenesis regulatory protein | 2.7 |  | 193 | Ribosomal biogenesis |
| NODE_406.g9424.t1 | hypothetical protein FPSE_00523 | Ribosomal protein L13 | 3.2 |  | 202 | Translation |
| NODE_412.g9492.t1 | Leucine--tRNA ligase, cytoplasmic | Aminoacyl-tRNA synthetase, class Ia | 1.6 |  | 1121 | Translation |
| NODE_414.g9517.t1 | uncharacterized protein FVRRES_05356 | Ribosomal protein S4e | 2.5 |  | 261 | Translation |
| NODE_415.g9525.t1 | endopolygalacturonase 1 | Glycosyl hydrolases family 28 | 2.0 |  | 360 | Metabolic |
| NODE_417.g9563.t1 | hypothetical protein BFJ71_g9688 |  | 7.0 | Cytoplasm | 75 |  |
| NODE_419.g9581.t1 | hypothetical protein FOTG_04099 | Mitochondrial ribosomal protein MRP51, fungi | 1.6 |  | 477 | Translation |
| NODE_419.g9582.t1 | hypothetical protein FAVG1_00109 | Ribosomal protein S17e | 3.4 |  | 147 | Translation |
| NODE_420.g9593.t1 | Fumarylacetoacetase | Fumarylacetoacetase | 4.7 |  | 425 | Metabolic |
| NODE_421.g9609.t1 | Putative 20S rRNA accumulation protein 4 | Programmed cell death protein 2, C-terminal | 2.9 |  | 401 |  |
| NODE_423.g9639.t1 | hypothetical protein FOQG_05736 | H/ACA ribonucleoprotein complex, subunit Gar1/Naf1 | 2.7 |  | 560 |  |
| NODE_425.g20572.t1 | hypothetical protein BFJ65_g14459 | Reverse transcriptase, RNA-dependent DNA polymerase | 2.9 |  | 295 | Biosynthetic |
| NODE_427.g9679.t1 | alcohol dehydrogenase | Aldo/keto reductase | 3.3 |  | 309 | Oxidation-reduction |
| NODE_427.g9682.t1 | Glucosamine 6-phosphate N-acetyltransferase | Acetyltransferase (GNAT) family | 1.3 | Cytoplasm | 176 | Biosynthetic |
| NODE_427.g9687.t1 | hypothetical protein FOTG_09180 | Ribosomal protein S35, mitochondrial | 1.4 |  | 321 | Translation |
| NODE_428.g9698.t1 | hypothetical protein FOC1_g10002574 |  | 1.6 |  | 386 |  |
| NODE_439.g9794.t1 | pectate lyase | Pectate lyase PlyH/PlyE-like | 7.2 |  | 367 | Metabolic |
| NODE_441.g9826.t1 | hypothetical protein BFJ68_g5846 | Peptidase M20 | 3.6 |  | 1285 | Proteolysis |
| NODE_441.g9827.t1 | probable DUF895 domain membrane protein | Major facilitator superfamily | 4.3 |  | 503 | Transport |
| NODE_442.g9835.t1 | hypothetical protein BFJ69_g3198 |  | 2.8 |  | 220 |  |
| NODE_444.g9860.t1 | CAMK/CAMKL/KIN4 protein kinase | Protein kinase domain-CAMK/CAMKL/KIN4 protein kinase | 1.2 |  | 1102 | Signal transduction |
| NODE_444.g9866.t1 | hypothetical protein FOPG_00077 | Pescadillo | 4.1 |  | 657 | Ribosome biogenesis |
| NODE_449.g9926.t1 | hypothetical protein BFJ69_g9167 | Protein of unknown function DUF2841 | 3.1 |  | 524 |  |
| NODE_451.g9943.t1 | hypothetical protein BFJ69_g7756 | Protein of unknown function DUF3405 | 1.4 |  | 757 |  |
| NODE_452.g9954.t1 | hypothetical protein BFJ71_g8244 |  | 2.3 |  | 219 |  |
| NODE_454.g9978.t1 | Malate dehydrogenase, mitochondrial | Malate dehydrogenase, type 1 | 1.1 |  | 336 | Metabolic |
| NODE_456.g9995.t1 | related to serine/threonine protein kinase | Protein kinase domain | 1.4 |  | 522 | Signal transduction |
| NODE_457.g10003.t1 | hypothetical protein FOQG_16975 | Carbohydrate-binding, CenC-like | 7.0 |  | 331 | Metabolic |
| NODE_459.g10022.t1 | hypothetical protein FOC1_g10010028 | Tetratricopeptide repeat | 2.3 |  | 851 | RNA processing |
| NODE_459.g10025.t1 | probable IgE-dependent histamine-releasing factor | Mss4/translationally controlled tumour-associated TCTP | 2.4 |  | 170 |  |
| NODE_462.g10041.t1 | alanine transaminase | Aminotransferase, class I/classII | 1.8 |  | 480 | Biosynthetic |
| NODE_462.g10047.t1 | hypothetical protein BFJ65_g10643 | PH-like domain superfamily | 1.4 |  | 490 |  |
| NODE_464.g10071.t1 | hypothetical protein FOXG_06069 | guanine nucleotide-binding (GTP) domain (Septin-type) | 1.7 |  | 383 | Signal transduction |
| NODE_465.g10085.t1 | hypothetical protein FPOA_00733 | Protein kinase domain- AGC | 3.9 |  | 1272 | Signal transduction |
| NODE_465.g20631.t1 | hypothetical protein BFJ65_g792 | WD domain, G-beta repeat | 3.5 | Cytoplasm | 199 | Signal transduction |
| NODE_468.g10114.t1 | hypothetical protein FOXG_13475 |  | 2.2 |  | 354 |  |
| NODE_468.g10115.t1 | hypothetical protein BFJ69_g6821 | Carboxylesterase, type B | 4.1 |  | 539 |  |
| NODE_470.g20640.t1 | mannitol 2-dehydrogenase | Mannitol dehydrogenase, C-terminal | 2.2 | Cytoplasm | 132 | Oxidation-reduction |
| NODE_472.g10172.t1 | hypothetical protein FOQG_04620 | Tetratricopeptide repeat | 1.4 |  | 718 | RNA processing |
| NODE_473.g10180.t1 | hypothetical protein BFJ69_g599 | 2Fe-2S ferredoxin-type iron-sulfur binding domain | 2.8 |  | 560 |  |
| NODE_473.g10186.t1 | hypothetical protein FOTG_16796 | FAD linked oxidase, N-terminal | 4.2 |  | 499 | Oxidation-reduction |
| NODE_475.g10200.t1 | hypothetical protein BFJ66_g14788, partial | Ribosomal protein L27 | 1.3 |  | 268 | Translation |
| NODE_475.g10205.t1 | hypothetical protein FOQG_04141 | N-lysine methyltransferase SETD6 | 2.7 |  | 463 |  |
| NODE_476.g10218.t1 | hypothetical protein BFJ69_g3846 | CPL domain | 3.2 |  | 683 | Methylation |
| NODE_476.g10220.t1 | hypothetical protein FOTG_05669 | Tetratricopeptide repeat | 1.9 |  | 373 | RNA processing |
| NODE_476.g10224.t1 | probable hnRNP arginine N-methyltransferase | S-adenosyl-L-methionine-dependent methyltransferase | 2.4 |  | 345 | Methylation |
| NODE_477.g10233.t1 | hypothetical protein FOPG_13928 |  | 4.1 |  | 276 |  |
| NODE_477.g10238.t1 | hypothetical protein FOQG_13866 | SKP1/BTB/POZ domain superfamily | 4.8 |  | 271 |  |
| NODE_478.g10252.t1 | hypothetical protein FOC1_g10014406 | Glycoside hydrolase, family 61 | 3.2 |  | 404 | Metabolic |
| NODE_480.g10269.t1 | hypothetical protein FPSE_00057 | Ribosomal protein S19/S15 | 3.7 |  | 152 | Translation |
| NODE_485.g10337.t1 | Pisatin demethylase | Cytochrome P450 | 5.6 |  | 510 | Oxidation-reduction |
| NODE_487.g10353.t1 | hypothetical protein FOTG_15935 |  | 2.6 |  | 196 |  |
| NODE_487.g10363.t1 | hypothetical protein FOXG_13102 |  | 5.3 |  | 467 |  |
| NODE_490.g10387.t1 | hypothetical protein BFJ69_g9090 | Amino acid transporter, transmembrane domain | 7.9 |  | 465 | Transport |
| NODE_495.g10420.t1 | hypothetical protein FOTG_15095 |  | 3.4 |  | 412 |  |
| NODE_497.g10445.t1 | hypothetical protein FOXG_07583 |  | 3.2 |  | 79 |  |
| NODE_498.g10461.t1 | hypothetical protein BFJ69_g14025 | Dual specificity protein phosphatase domain | 2.7 |  | 934 | Protein dephosphorylation |
| NODE_500.g10477.t1 | hypothetical protein BFJ69_g11453 | Calcium-dependent channel, 7TM region, putative phosphate | 1.4 |  | 1046 | Transport |
| NODE_501.g10493.t1 | hypothetical protein BFJ68_g16085 | Cys/Met metabolism, pyridoxal phosphate-dependent enzyme | 5.8 |  | 393 | Transsulfuration |
| NODE_512.g10648.t1 | hypothetical protein FOQG_15251 |  | 3.2 |  | 198 |  |
| NODE_515.g10675.t1 | arginase | Ureohydrolase | 2.6 |  | 325 |  |
| NODE_515.g10677.t1 | Mitochondrial transcription factor 1 | S-adenosyl-L-methionine-dependent methyltransferase | 1.4 |  | 594 | Methylation |
| NODE_516.g10683.t1 | Putative methionyl-tRNA synthetase, cytoplasmic | Methionyl/Leucyl tRNA synthetase | 1.7 |  | 678 | Translation |
| NODE_516.g10687.t1 | Alpha-xylosidase | Glycoside hydrolase family 31 | 2.8 |  | 781 | Metabolic |
| NODE_517.g10692.t1 | hypothetical protein FOC4_g10012645 | Queuosine salvage protein family | 2.3 |  | 365 |  |
| NODE_517.g10701.t1 | ADP, ATP carrier protein | Mitochondrial substrate/solute carrier | 2.5 |  | 312 | Transport |
| NODE_520.g10726.t1 | hypothetical protein FAVG1_01712 | IMP dehydrogenase/GMP reductase | 2.1 |  | 532 | Oxidation-reduction |
| NODE_522.g10745.t1 | related to NOP16 constituent of 66S pre-ribosomal particles | Ribosome biogenesis protein Nop16 | 2.5 |  | 215 | Ribosome biogenesis |
| NODE_524.g10773.t1 | probable acetyl-CoA carboxylase | Acetyl-CoA carboxylase, central domain | 1.1 |  | 2284 | Biosynthetic |
| NODE_525.g10783.t1 | Putative glucose transporter rco-3 | Major facilitator, sugar transporter-like | 4.9 |  | 540 | Transport |
| NODE_526.g10785.t1 | hypothetical protein FAVG1_02914 | Ribosomal L18e/L15P superfamily | 3.4 |  | 149 | Translation |
| NODE_541.g10938.t1 | Peroxisomal acyl-coenzyme A oxidase 1 | Acyl-CoA oxidase/dehydrogenase, central domain | 2.5 |  | 698 | Oxidation-reduction |
| NODE_543.g10956.t1 | hypothetical protein BFJ69_g10227 | S-adenosylmethionine decarboxylase | 1.3 |  | 494 | Biosynthetic |
| NODE_543.g10957.t1 | hypothetical protein FPSE_07318 | H/ACA ribonucleoprotein complex, subunit Nop10 | 2.1 |  | 61 | Ribosome biogenesis |
| NODE_544.g10979.t1 | hypothetical protein FOMG_14103 | Glycosyl hydrolase, family 13, catalytic domain | 5.9 |  | 576 | Metabolic |
| NODE_548.g11009.t1 | tRNA pseudouridine synthase 1 | Pseudouridine synthase I, TruA | 3.4 |  | 665 | RNA modification |
| NODE_554.g11081.t1 | Pepsin A | Aspartic peptidase A1 family | 3.8 |  | 384 | Proteolysis |
| NODE_555.g11089.t1 | hypothetical protein BFJ69_g1311 |  | 5.1 |  | 331 |  |
| NODE_559.g11137.t1 | Endo-1,4-beta-xylanase | Glycoside hydrolase family 10 domain | 7.1 |  | 356 | Metabolic |
| NODE_562.g11165.t1 | ribonuclease Z | Metallo-beta-lactamase | 2.5 |  | 836 |  |
| NODE_568.g11237.t1 | hypothetical protein FOTG_09210 | Zn (2)-C6 fungal-type DNA-binding domain superfamily | 1.4 |  | 468 | Transcription |
| NODE_575.g11310.t1 | Aquaporin-2 | Major intrinsic protein | 1.9 |  | 320 | Transport |
| NODE_579.g11338.t1 | dihydrolipoamide acetyltransferase component pyruvate dehydrogenase complex | 2-oxoacid dehydrogenase acyltransferase, catalytic domain | 2.1 |  | 457 |  |
| NODE_580.g11360.t1 | hypothetical protein BFJ69_g9020 | Ribosomal protein L24e-related | 2.5 |  | 171 | Translation |
| NODE_583.g11381.t1 | hypothetical protein FPSE_06440 | ATP synthase, F0 complex, subunit D, mitochondrial | 1.2 | Mitochondrion | 105 | Transportation |
| NODE_583.g11382.t1 | probable nascent polypeptide-associated complex alpha chain | Nascent polypeptide-associated complex NAC domain | 2.7 |  | 209 | Transport |
| NODE_584.g11394.t1 | probable beta karyopherin | Importin repeat 4 | 2.5 |  | 1096 | Transport |
| NODE_585.g11406.t1 | hypothetical protein FOXG_16569 |  | 5.6 |  | 145 |  |
| NODE_589.g11441.t1 | STE/STE20/PAKA protein kinase | Protein kinase domain | 1.5 |  | 871 | Signal transduction |
| NODE_589.g11443.t1 | hypothetical protein BFJ71_g5962 | Enoyl- (Acyl carrier protein) reductase | 3.5 |  | 362 | Metabolic |
| NODE_589.g11452.t1 | 3-ketoacyl-CoA thiolase, peroxisomal | acetyl-coenzyme A acetyltransferases (Thiolases) | 1.1 |  | 400 |  |
| NODE_592.g11467.t1 | hypothetical protein FOC1_g10005850 | Methyltransferase domain 25 | 1.2 |  | 622 | Methylation |
| NODE_604.g11596.t1 | acetylornithine aminotransferase, mitochondrial | Aminotransferase class-III | 2.7 |  | 446 | Biosynthetic |
| NODE_608.g11622.t1 | hypothetical protein FOXG_09391 | KRR1 interacting protein 1 | 2.7 |  | 657 |  |
| NODE_608.g11624.t1 | hypothetical protein BFJ69_g9745 | Zinc finger, RING/FYVE/PHD-type | 1.1 |  | 930 | Transcription |
| NODE_608.g11630.t1 | hypothetical protein FPSE_03509 | Ribosomal protein S10 | 2.9 |  | 116 | Translation |
| NODE_610.g11642.t1 | hypothetical protein FOCG_01770 | NAD(P)-binding domain superfamily | 3.7 |  | 325 |  |
| NODE_612.g11673.t1 | hypothetical protein FOPG_07758 | Alpha/Beta hydrolase fold | 3.0 |  | 375 | Metabolic |
| NODE_613.g11689.t1 | hypothetical protein FOXG_06328 |  | 7.0 |  | 258 |  |
| NODE_614.g11692.t1 | hypothetical protein BFJ69_g6260 | Glycoside hydrolase, family 61 | 4.7 |  | 327 | Metabolic |
| NODE_614.g11696.t1 | endoglucanase type B | Glycoside hydrolase, family 6, conserved site | 7.1 |  | 462 | Metabolic |
| NODE_618.g20824.t1 | hypothetical protein FLONG3_2226 | Fatty acid synthase, meander beta sheet domain | 4.1 | Mitochondrion | 111 | Oxidation-reduction |
| NODE_620.g11759.t1 | hypothetical protein FOPG_13535 | Glycoside hydrolase, family 3, N-terminal | 4.8 |  | 840 | Metabolic |
| NODE_620.g11767.t1 | hypothetical protein FOIG_14387 | Major facilitator, sugar transporter-like | ### |  | 552 | Transport |
| NODE_625.g11803.t1 | Fidgetin-like protein 1 | ATPase family associated with various cellular activities (AAA) | 3.0 |  | 488 |  |
| NODE_625.g11811.t1 | hypothetical protein BFJ72_g5189 | Major facilitator, sugar transporter-like | 6.3 |  | 516 | Transport |
| NODE_633.g11882.t1 | hypothetical protein FOC1_g10012878 | Tetratricopeptide repeat | 2.2 |  | 806 | RNA processing |
| NODE_633.g11886.t1 | related to translation initiation factor 3 (47 kDa subunit) | Eukaryotic translation initiation factor 3 subunit F | 2.8 |  | 358 | Translation |
| NODE_633.g11887.t1 | hypothetical protein FOQG_06619 | U3 small nucleolar RNA-associated SSU processome protein 25 (Utp25) | 2.9 |  | 719 | rRNA processing |
| NODE_633.g11888.t1 | hypothetical protein FOCG_04420 | Ribosomal protein L17 | 1.9 |  | 227 | Translation |
| NODE_636.g11914.t1 | hypothetical protein FOQG_00554 | Armadillo-like helical | 1.5 |  | 759 |  |
| NODE_636.g11917.t1 | hypothetical protein FOXG_01767 | Mitochondrial glycoprotein | 1.3 |  | 290 | Metabolic |
| NODE_637.g11924.t1 | probable heat shock protein 70 | Heat shock protein 70 family | 6.0 |  | 614 | Stress response |
| NODE_639.g11938.t1 | hypothetical protein FOQG_10910 | Chloramphenicol acetyltransferase-like domain superfamily | 2.7 |  | 513 |  |
| NODE_639.g11939.t1 | hypothetical protein FOMG_11532 | Oligopeptide transporter, OPT superfamily | 7.5 |  | 742 | Transport |
| NODE_641.g11959.t1 | hypothetical protein FOXG_04914 | Alpha/Beta hydrolase fold | 2.8 |  | 285 | Metabolic |
| NODE_650.g12039.t1 | hypothetical protein FOTG_00377 | Far11/STRP, N-terminal | 1.2 |  | 1023 |  |
| NODE_651.g12048.t1 | hydroxymethylglutaryl-CoA synthase | Hydroxymethylglutaryl-coenzyme A synthase, N-terminal | 1.0 |  | 456 | Biosynthetic |
| NODE_655.g12092.t1 | hypothetical protein FOPG_08978 |  | 7.7 |  | 921 |  |
| NODE_655.g12097.t1 | hypothetical protein BFJ69_g11872, partial | Ribosomal protein S19e | 1.6 |  | 139 | Translation |
| NODE_660.g12144.t1 | hypothetical protein FOTG_07898 | PAS domain | 4.5 |  | 527 |  |
| NODE_663.g12166.t1 | GTP-binding nuclear protein GSP1/Ran | Small GTPase | 1.5 |  | 216 | Signal Transduction |
| NODE_663.g12173.t1 | Uncharacterized protein Y057_14029 | RNA 3'-terminal phosphate cyclase type 2 | 2.6 |  | 404 | Ribosome biogenesis |
| NODE_663.g12177.t1 | Protein PXR1 | G-patch domain | 2.7 |  | 361 |  |
| NODE_668.g12213.t1 | CAMKK protein kinase | Protein kinase domain-CaMKK | 1.1 |  | 1245 | Signal transduction |
| NODE_673.g12269.t1 | hypothetical protein FOTG_12278 | Glucose receptor Git3, N-terminal-G protein-coupled | 3.3 |  | 389 | Signal transduction |
| NODE_683.g12357.t1 | isovaleryl-CoA dehydrogenase | Acyl-CoA dehydrogenase/oxidase C-terminal | 2.6 |  | 427 | Oxidation-reduction |
| NODE_687.g12393.t1 | hypothetical protein FOPG_09136 | Ribonuclease II/R | 2.5 |  | 1046 | RNA processing |
| NODE_687.g12394.t1 | Putative beta-glucosidase btgE | Glycoside hydrolase superfamily | 1.1 |  | 610 | Metabolic |
| NODE_691.g12421.t1 | hypothetical protein FOXG_12843 | Nop domain | 3.8 |  | 593 | Ribosome biogenesis |
| NODE_696.g12449.t1 | hypothetical protein BFJ65_g18018 | U3 small nucleolar RNA-associated SSU processome protein (Utp11) | 2.3 |  | 268 | rRNA processing |
| NODE_696.g12450.t1 | hypothetical protein FOC4_g10001474 | Conserved proline-rich protein | 5.3 |  | 597 |  |
| NODE_703.g12511.t1 | Choline dehydrogenase, mitochondrial | Glucose-methanol-choline oxidoreductase, N-terminal | 4.6 |  | 459 | Oxidation-reduction |
| NODE_706.g12543.t1 | hypothetical protein BFJ69_g14665 | NADH:cytochrome b5 reductase-like | 6.5 |  | 450 | Oxidation-reduction |
| NODE_706.g12544.t1 | Cytochrome P450 4F4 | Cytochrome P450 | 5.4 |  | 549 | Oxidation-reduction |
| NODE_707.g12552.t1 | hypothetical protein FOC1_g10008301 | U3 small nucleolar RNA-associated protein 8 | 3.8 |  | 903 | rRNA processing |
| NODE_709.g12573.t1 | hypothetical protein BFJ68_g11938 | Class II aldolase/adducin N-terminal | 6.0 |  | 298 | Metabolic |
| NODE_710.g12582.t1 | hypothetical protein BFJ69_g13017 | Brix domain | 3.7 |  | 317 | Ribosomal large subunit assembly |
| NODE_712.g12595.t1 | NADP-specific glutamate dehydrogenase | Glutamate/phenylalanine/leucine/valine dehydrogenase, C-terminal | 1.3 |  | 456 | Oxidation-reduction |
| NODE_717.g12625.t1 | hypothetical protein FOCG_16280 | NACHT domain | 6.5 |  | 527 |  |
| NODE_725.g12684.t1 | hypothetical protein FOXG_08613 | Zinc finger, GATA-type | 2.1 |  | 501 | Transcription |
| NODE_729.g12708.t1 | 4-aminobutyrate aminotransferase | Aminotransferase class-III | 2.0 |  | 459 | Biosynthetic |
| NODE_735.g12744.t1 | hypothetical protein FPSE_09444 | Ribosomal protein S3Ae | 2.1 |  | 256 | Translation |
| NODE_740.g12796.t1 | hypothetical protein FOC4_g10014215 | Solute carrier family 35 member SLC35F1/F2/F6 | 1.4 |  | 408 | Transport |
| NODE_743.g12831.t1 | Protein pyrABCN | Carbamoyl-phosphate synthase large subunit, CPSase domain | 5.7 |  | 1903 | Metabolic |
| NODE_748.g12868.t1 | hypothetical protein FOXG_01332 | Amino acid transporter, transmembrane domain | 3.4 |  | 475 | Transport |
| NODE_748.g12869.t1 | hypothetical protein FOMG_02164 | Polyketide synthase, enoylreductase domain | 3.3 |  | 348 | Biosynthetic |
| NODE_748.g12872.t1 | hypothetical protein FOMG_02161 | NADPH-dependent FMN reductase-like | 6.5 |  | 335 | Oxidation-reduction |
| NODE_750.g12885.t1 | potassium/sodium efflux P-type ATPase, fungal-type | Cation-transporting P-type ATPase, C-terminal | 1.7 |  | 1072 | Transport |
| NODE_751.g12890.t1 | hypothetical protein FOQG_14230 | Phosphoribosylaminoimidazole carboxylase, fungi/plant | 2.0 |  | 594 | Biosynthetic |
| NODE_753.g12909.t1 | Putative phosphoketolase | Xylulose 5-phosphate/Fructose 6-phosphate phosphoketolase | 1.5 |  | 819 | Metabolic |
| NODE_756.g12923.t1 | Lactose permease | Major facilitator, sugar transporter-like | 6.7 |  | 523 | Transport |
| NODE_756.g12927.t1 | hypothetical protein FOQG_15132 | Hexokinase, N-terminal | 4.5 |  | 592 | Metabolic |
| NODE_756.g12930.t1 | hypothetical protein FOXG_02597 | NodB homology domain | 7.2 | Extracellular | 255 | Metabolic |
| NODE_757.g12934.t1 | DNA topoisomerase 1 | DNA topoisomerase I, DNA binding, eukaryotic type | 1.5 |  | 930 | DNA topological change |
| NODE_758.g12938.t1 | hypothetical protein FAVG1_01471 | Aminoacyl-tRNA synthetase, class II (G/ P/ S/T) | 1.9 |  | 988 | Translation |
| NODE_758.g12942.t1 | cytochrome c peroxidase, mitochondrial | Haem peroxidase | 1.2 |  | 358 | Proteolysis |
| NODE_761.g12966.t1 | hypothetical protein FOTG_05904 | CCAAT-binding factor | 4.5 |  | 986 |  |
| NODE_761.g12968.t1 | hypothetical protein FOTG_05902 | Thioesterase domain | 2.3 | Mitochondrion | 272 |  |
| NODE_765.g12995.t1 | NAD(P)H-dependent D-xylose reductase xyl1 | Aldo/keto reductase | 1.7 |  | 324 | Oxidation-reduction |
| NODE_767.g13013.t1 | hypothetical protein FOXG_10535 | Phosphoribosylglycinamide synthetase | 3.0 |  | 797 | Biosynthetic |
| NODE_767.g13015.t1 | hypothetical protein FAVG1_00589 | Uncharacterised protein family UPF0642 | 3.1 |  | 119 |  |
| NODE_767.g13018.t1 | putative RNA-binding protein C4F6.14 | RNA recognition motif domain | 3.5 |  | 736 | Cytokinesis |
| NODE_778.g13119.t1 | hypothetical protein FPSE_09944 | Ribosomal protein L14P | 3.2 | Mitochondrion | 139 | Translation |
| NODE_780.g13140.t1 | hypothetical protein FOQG_13956 | Galactose-binding-like domain superfamily | 4.7 |  | 564 |  |
| NODE_782.g13156.t1 | hypothetical protein FOTG_11127 | Cation/H+ exchanger | 4.0 |  | 877 | Transport |
| NODE_782.g13160.t1 | Putative peptide transporter ptr2 | Major facilitator, sugar transporter-like | ### |  | 611 | Transport |
| NODE_789.g13218.t1 | hypothetical protein FPOA_01302 | Ribosomal protein S28e | 3.7 |  | 68 | Translation |
| NODE_793.g13245.t1 | hypothetical protein FOPG_12037 | Cellulose-binding domain, fungal | 7.0 |  | 357 | Metabolic |
| NODE_794.g13252.t1 | chitin synthase | Fungal chitin synthase | 1.4 |  | 902 | Biosynthetic |
| NODE_799.g13280.t1 | hypothetical protein FOQG_02582 | Protein kinse domain-Histidine kinase | 4.7 |  | 830 | Signal transduction |
| NODE_801.g13294.t1 | pectate lyase E | Pectate lyase PlyH/PlyE-like | 7.3 |  | 226 | Metabolism |
| NODE_806.g13345.t1 | hypothetical protein FOPG_02227 | Nucleolar protein 14 | 2.8 |  | 901 | Ribosome biogenesis |
| NODE_807.g13357.t1 | hypothetical protein FPSE_03202 | Ribosomal protein S14 | 2.8 | Mitochondrion | 59 | Translation |
| NODE_815.g13411.t1 | xyloglucanase | Glycoside hydrolase family 74 | 4.7 |  | 657 | Metabolic |
| NODE_818.g13433.t1 | Putative 4-hydroxy-2-oxoglutarate aldolase, mitochondrial | Dihydrodipicolinate synthetase family | 5.0 |  | 345 |  |
| NODE_818.g13434.t1 | hypothetical protein BFJ69_g6264 | Short-chain dehydrogenase/reductase SDR | 4.7 | Peroxisome | 271 | Oxidation-reduction |
| NODE_820.g13450.t1 | hypothetical protein FOC1_g10009903 | S-adenosyl-L-methionine-dependent methyltransferase | 1.3 |  | 330 | Methylation |
| NODE_827.g13519.t1 | uncharacterized protein FPRO_11845 | Zinc finger C2H2 superfamily | 1.8 |  | 685 | Transcription |
| NODE_827.g13524.t1 | hypothetical protein FOXG_04260 | Protein of unknown function DUF3455 | 3.2 |  | 264 |  |
| NODE_829.g13552.t1 | salicylate hydroxylase | FAD-binding domain | 3.3 |  | 427 |  |
| NODE_834.g13587.t1 | Protein sda1 | Uncharacterised domain NUC130/133, N-terminal | 3.8 |  | 740 |  |
| NODE_834.g13588.t1 | hypothetical protein FOXG_11113 | Mitochondrial substrate/solute carrier | 2.9 |  | 321 | Transport |
| NODE_834.g13591.t1 | L-2-aminoadipate reductase large subunit | AMP-dependent synthetase/ligase | 2.0 |  | 888 |  |
| NODE_835.g13595.t1 | mitochondrial import receptor subunit tom-40 | Eukaryotic porin/Tom40 | 1.2 |  | 356 | Transport |
| NODE_837.g13608.t1 | hypothetical protein FOPG_15295 | RmlC-like cupin domain superfamily | 4.4 |  | 189 |  |
| NODE_841.g13643.t1 | galacturan 1,4-alpha-galacturonidase | Glycoside hydrolase, family 28 | 6.3 |  | 464 | Metabolic |
| NODE_842.g13647.t1 | hypothetical protein FOMG_05050 | Heterokaryon incompatibility | 1.2 |  | 775 |  |
| NODE_844.g13663.t1 | L-lactate dehydrogenase (cytochrome) | Cytochrome b5-like heme/steroid binding domain | 1.8 |  | 502 | Oxidation-reduction |
| NODE_845.g13673.t1 | L-arabinitol 4-dehydrogenase | Alcohol dehydrogenase, N-terminal | 2.6 |  | 375 | Oxidation-reduction |
| NODE_845.g13674.t1 | Fe/S biogenesis protein NfuA | NIF system FeS cluster assembly, NifU, C-terminal | 1.4 |  | 305 | Iron-sulfur cluster assembly |
| NODE_848.g13690.t1 | delta-aminolevulinic acid dehydratase | Delta-aminolevulinic acid dehydratase | 1.8 |  | 386 | Biosynthetic |
| NODE_868.g13817.t1 | hypothetical protein FOQG_14044 | Glycoside hydrolase, family 43 | 3.3 |  | 530 | Metabolic |
| NODE_869.g13830.t1 | probable YHM1 (mitochondrial carrier) | Mitochondrial substrate/solute carrier | 1.0 |  | 303 | Transport |
| NODE_870.g13838.t1 | hypothetical protein FOQG_09589 | rRNA-processing protein Fcf1/Utp23 | 2.6 |  | 288 | rRNA processing |
| NODE_871.g13840.t1 | probable protein urg3 | Protein of unknown function DUF1688 | 2.1 |  | 529 |  |
| NODE_884.g13911.t1 | hypothetical protein FOC1_g10005228 | ZN622/Rei1/Reh1, zinc finger C2H2-type | 2.8 |  | 548 | Transcription |
| NODE_884.g13916.t1 | hypothetical protein FOC1_g10005223 | Srp40, C-terminal | 2.2 |  | 414 | Translation |
| NODE_886.g13933.t1 | related to PSA1-mannose-1-phosphate guanyltransferase | Nucleotidyl transferase domain | 1.7 |  | 447 | Biosynthetic |
| NODE_889.g13951.t1 | 54S ribosomal protein L12, mitochondrial | Ribosomal protein L7/L12, oligomerisation | 1.1 |  | 181 | Translation |
| NODE_889.g13952.t1 | hypothetical protein FOTG_00956 | ATPase, dynein-related, AAA domain | 3.0 |  | 4924 |  |
| NODE_89.g3607.t1 | hypothetical protein FOQG_06539 | U3 small nucleolar ribonucleoprotein complex, subunit Mpp10 | 3.4 |  | 709 | rRNA processing |
| NODE_896.g13995.t1 | S-(hydroxymethyl)glutathione synthase | Glutathione-dependent formaldehyde-activating enzyme | 3.8 |  | 227 | Catabolic |
| NODE_896.g14002.t1 | hypothetical protein BFJ69_g8711 |  | 2.8 |  | 154 |  |
| NODE_898.g14008.t1 | sulfite reductase (ferredoxin) | Nitrite/sulphite reductase 4Fe-4S domain | 1.4 |  | 1350 | Oxidation-reduction |
| NODE_898.g14012.t1 | hypothetical protein BFJ70_g11791 | Ribosome biogenesis protein Nop53/GLTSCR2 | 2.8 |  | 439 | Ribosome biogenesis |
| NODE_901.g14035.t1 | hypothetical protein BFJ65_g17544 | S-adenosyl-L-methionine-dependent methyltransferase | 3.7 |  | 213 | Methylation |
| NODE_901.g14041.t1 | hypothetical protein FOC1_g10003508 | integral membrane protein | 6.6 |  | 397 |  |
| NODE_906.g14082.t1 | hypothetical protein FOXG_01504 | Pyridine nucleotide-disulphide oxidoreductase | 3.2 |  | 390 | Oxidation-reduction |
| NODE_912.g14121.t1 | alpha-N-arabinofuranosidase C | Alpha-L-arabinofuranosidase, C-terminal | 5.3 |  | 507 | Metabolic |
| NODE_913.g14125.t1 | Nuclear GTP-binding protein NUG1 | Guanine nucleotide-binding protein-like 3, (GTPase)N-terminal domain | 3.5 |  | 544 | Signal transduction |
| NODE_913.g14129.t1 | Mitochondrial import inner membrane translocase subunit tim54 | Mitochondrial import inner membrane translocase subunit Tim54 | 2.4 |  | 293 | Transport |
| NODE_916.g14147.t1 | hypothetical protein BFJ69_g3312 | Transcription factor Iwr1 domain | 2.1 |  | 433 | Transcription |
| NODE_917.g14157.t1 | hypothetical protein BFJ65_g8669 | Ubiquinol-cytochrome C reductase hinge domain superfamily | 1.0 |  | 134 | Oxidation-reduction |
| NODE_918.g14159.t1 | hypothetical protein FOIG_06953 | Ribosomal protein L22/L17 | 1.0 |  | 333 | Translation |
| NODE_918.g14163.t1 | 3-oxoacyl- | Enoyl- (Acyl carrier protein) reductase | 3.5 |  | 272 | Metabolic |
| NODE_918.g14164.t1 | hypothetical protein FOTG_02726 | Enolase C-terminal domain-like | 5.6 |  | 448 |  |
| NODE_92.g3702.t1 | Plasma membrane fusion protein PRM1 | Plasma membrane fusion protein PRM1 | 2.4 |  | 732 |  |
| NODE_922.g14195.t1 | hypothetical protein FOXG_08549 | Ribosomal RNA-processing protein 8, N-terminal domain | 2.7 |  | 487 | RNA processing |
| NODE_93.g3709.t1 | hypothetical protein FOC1_g10008850 | Methyltransferase domain | 1.6 |  | 381 | Methylation |
| NODE_93.g3721.t1 | ATP-dependent RNA helicase, mitochondrial | P-loop containing nucleoside triphosphate hydrolase | 4.3 |  | 608 | Phosphorylation |
| NODE_930.g14247.t1 | hypothetical protein FOC1_g10002504 | Xanthine/uracil/vitamin C permease | 1.2 |  | 582 | Transport |
| NODE_930.g14248.t1 | uncharacterized protein FPRN_08760 | nucleoside transporter | 3.0 |  | 380 | Transport |
| NODE_935.g14284.t1 | Uncharacterized protein LW93_9471 | Cupin 1 | 3.4 |  | 465 | Metabolic |
| NODE_937.g14300.t1 | hypothetical protein FOXG_08092 | Nrap protein, domain 3 | 3.2 |  | 709 |  |
| NODE_938.g14314.t1 | hypothetical protein FPSE_02953 | Ribosomal protein S5/S7, eukaryotic/archaeal | 2.5 | Cytoplasm | 213 | Translation |
| NODE_94.g3735.t1 | hypothetical protein FOXG_08476 | Bystin | 3.5 |  | 485 | Ribosome biogenesis |
| NODE_941.g14332.t1 | hypothetical protein FOQG_01163 | ML-like domain | 1.6 |  | 835 | Transport |
| NODE_942.g14333.t1 | Oligopeptide transporter 3 | Oligopeptide transporter, OPT superfamily | 6.4 |  | 784 | Transport |
| NODE_948.g14367.t1 | hypothetical protein FOC1_g10009461 | Polyketide synthase, enoylreductase domain | 3.0 |  | 353 | Biosynthetic |
| NODE_948.g14369.t1 | hypothetical protein BFJ71_g3203 |  | 3.1 |  | 92 |  |
| NODE_957.g14427.t1 | hypothetical protein FOC1_g10002644 |  | 2.2 |  | 576 |  |
| NODE_959.g14441.t1 | Nucleoside diphosphate kinase | Nucleoside diphosphate kinase | 1.6 |  | 238 | Biosynthetic |
| NODE_961.g14457.t1 | hypothetical protein FOXG_08441 |  | 1.7 |  | 285 |  |
| NODE_962.g14461.t1 | hypothetical protein BFJ65_g3552 | Male sterility, NAD-binding | 4.5 |  | 630 |  |
| NODE_963.g14471.t1 | mitochondrial import inner membrane translocase subunit TIM10 | Tim10 | 1.4 |  | 96 | Transport |
| NODE_965.g14482.t1 | Aconitate hydratase | Aconitase/3-isopropylmalate dehydratase large subunit, alpha/beta/alpha domain | 1.9 |  | 785 | Metabolic |
| NODE_966.g14494.t1 | hypothetical protein BFJ69_g5154 | Ribosomal RNA-processing protein 14/surfeit locus protein 6, C-terminal domain | 2.4 |  | 549 | RNA processing |
| NODE_967.g14496.t1 | hypothetical protein FOC1_g10011090 | Zn (2)-C6 fungal-type DNA-binding domain | 4.1 |  | 682 | Transcription |
| NODE_968.g14502.t1 | hypothetical protein FOTG_00952 | U3 small nucleolar RNA-associated protein 10, N-terminal | 4.0 |  | 1807 | rRNA processing |
| NODE_968.g14503.t1 | Putative NADH dehydrogenase | Pyridine nucleotide-disulphide oxidoreductase | 3.1 |  | 456 | Oxidation-reduction |
| NODE_970.g14520.t1 | Glucosamine-6-phosphate isomerase 1 | Glucosamine/galactosamine-6-phosphate isomerase | 2.2 |  | 426 | Metabolic |
| NODE_970.g14522.t1 | Putative N-acetylglucosamine-6-phosphate deacetylase | N-acetylglucosamine-6-phosphate deacetylase | 3.0 |  | 444 | Metabolic |
| NODE_975.g14560.t1 | hypothetical protein FOIG_16381 | ino80 chromatin remodeling complex protein | 1.1 |  | 1018 | Cell organisation |
| NODE_977.g14566.t1 | uncharacterized protein FMAN_11213 | CFEM domain | 4.2 |  | 195 |  |
| NODE_979.g14579.t1 | Large subunit GTPase 1 | GTP binding domain | 2.1 |  | 672 | Signal transduction |
| NODE_979.g14580.t1 | hypothetical protein FOXG_01607 | Low temperature viability protein Ltv1 | 2.8 |  | 408 | Ribosomal small subunit biogenesis |
| NODE_982.g14600.t1 | hypothetical protein FOXG_10472 | RNA recognition motif domain | 3.4 |  | 551 | Cytokinesis |
| NODE_990.g14663.t1 | Delta-1-pyrroline-5-carboxylate dehydrogenase | Aldehyde dehydrogenase domain | 2.8 |  | 572 | Oxidation-reduction |
| NODE_990.g14665.t1 | hypothetical protein FOTG_05682 | Uncharacterised protein family UPF0592 | 1.4 |  | 1212 |  |
| NODE_993.g14681.t1 | Vacuolar cation/proton exchanger 2 | Sodium/calcium exchanger membrane region | 4.8 |  | 450 | Transport |
| NODE_995.g14687.t1 | hypothetical protein FOTG_16786 | Six-hairpin glycosidase-like superfamily | 3.3 |  | 490 |  |
| DN10042_c0_g1_i3.g36038.t1 | hypothetical protein BFJ68_g12847 | OPT oligopeptide transporter protein | 4.8 |  | 109 | Transport |
| DN10125_c0_g1_i1.g33827.t1 | hypothetical protein FOCG_16954 | Peptidase S8/S53 domain superfamily | 4.1 |  | 125 | Proteolysis |
| DN1014_c0_g1_i1.g11806.t1 | hypothetical protein FOTG_00142 |  | 2.3 |  | 261 |  |
| DN10333_c0_g1_i1.g13958.t1 | hypothetical protein FOXG_13912 | Brix domain | 3.0 |  | 307 | Ribosomal large subunit assembly |
| DN10377_c0_g1_i1.g13977.t1 | hypothetical protein BFJ71_g11112 | Amino acid transporter, transmembrane domain | 3.1 |  | 284 | Transport |
| DN1047_c0_g1_i7.g11967.t1 | hypothetical protein BFJ65_g12668 |  | 6.3 |  | 244 |  |
| DN1048_c0_g1_i1.g12018.t1 | beta-glucosidase | Glycosyl hydrolase family 1 | 3.1 |  | 380 | Metabolic |
| DN1062_c0_g1_i6.g11977.t1 | centromere/microtubule-binding protein cbf5 | tRNA pseudouridine synthase B family | 5.0 |  | 487 | RNA processing |
| DN1064_c0_g1_i1.g11751.t1 | cytochrome c | Cytochrome c-like domain | 2.7 |  | 106 | Oxidation-reduction |
| DN1064_c0_g1_i1.g11752.t1 | hypothetical protein FOTG_06965 | ATPase, AAA-type | 2.2 |  | 651 | Transport |
| DN10804_c0_g1_i1.g28875.t1 | Galactose oxidase | Galactose oxidase, central domain superfamily | 4.4 |  | 238 | Oxidation-reduction |
| DN11001_c0_g1_i1.g6233.t1 | hypothetical protein BFJ72_g6263 |  | 4.8 |  | 169 |  |
| DN1107_c0_g1_i3.g9464.t1 | hypothetical protein BFJ72_g11846 | Ribosomal protein L31e | 2.2 |  | 122 | Translation |
| DN11071_c0_g1_i1.g6238.t1 | uncharacterized protein FFUJ_01499 | La-type HTH domain | 3.3 | Nucleus | 291 | RNA processing |
| DN11273_c0_g1_i1.g19785.t1 | hypothetical protein FOQG_04113 | Ribosome biogenesis protein Alb1 | 2.6 |  | 170 | Ribosome biogenesis |
| DN1129_c0_g1_i1.g9689.t1 | hypothetical protein FOMG_11171 | Ribosomal protein L24e | 1.8 |  | 471 | Translation |
| DN11342_c0_g1_i1.g26194.t1 | uncharacterized protein FFUJ_01583 | 4-coumarate coenzyme A ligase | 6.4 |  | 90 |  |
| DN11366_c0_g1_i1.g811.t1 | Elongation factor 1-alpha | Translation elongation factor EF1A, eukaryotic/archaeal | 2.5 |  | 460 | Translation |
| DN1143_c0_g1_i2.g9646.t1 | hypothetical protein FPSE_10207 | Ribosomal protein L19/L19e | 1.7 |  | 261 | Translation |
| DN1143_c0_g1_i3.g9647.t1 | eIF-2-alpha kinase activator GCN1 | Ribosomal protein L19/L19e | 1.3 |  | 188 | Translation |
| DN1149_c0_g1_i2.g34275.t1 | hypothetical protein FSPOR_9594 | Ribosomal protein S15 | 2.5 |  | 158 | Translation |
| DN11510_c0_g1_i1.g25318.t1 | hypothetical protein FAVG1_12638 | Sterile alpha motif domain | 3.2 |  | 163 |  |
| DN11559_c0_g1_i1.g12144.t1 | L-rhamnonate dehydratase | Enolase C-terminal domain-like | 4.4 | Cytoplasm | 106 |  |
| DN1174_c0_g1_i2.g9504.t1 | hypothetical protein FOTG_00992 | Ubiquinol-cytochrome c chaperone/UPF0174 | 1.3 |  | 288 | Stress response |
| DN11749_c0_g1_i1.g9342.t1 | hypothetical protein BFJ69_g7223 | H/ACA ribonucleoprotein complex, subunit Gar1/Naf1 | 3.9 |  | 177 |  |
| DN1175_c0_g1_i5.g34714.t1 | probable chaperonin of the TCP1 ring complex | Chaperonin Cpn60/TCP-1 family | 1.3 |  | 538 | Stress response |
| DN1184_c0_g1_i1.g34723.t1 | hypothetical protein FOXG_04752 | Major facilitator superfamily | 6.4 |  | 486 | Transport |
| DN11842_c0_g1_i1.g670.t1 | hypothetical protein BFJ70_g7983 | Major Facilitator Superfamily | 7.4 |  | 158 | Transport |
| DN1200_c0_g1_i1.g7698.t1 | hypothetical protein FOXG_13874 | Protein of unknown function DUF3984 | 1.6 |  | 370 |  |
| DN1201_c0_g1_i3.g17355.t1 | hypothetical protein FOXG_08113 | Signal transduction response regulator, receiver domain | 1.2 |  | 520 | Signal transduction |
| DN12013_c0_g1_i1.g16543.t1 | family inorganic phosphate transporter | Phosphate transporter | 6.7 |  | 82 | Transport |
| DN12211_c0_g1_i1.g5712.t1 | putative nucleolar complex protein 14 | Nucleolar protein 14 | 4.6 |  | 158 | Ribosome biogenesis |
| DN12257_c0_g1_i1.g5714.t1 | class I alpha-mannosidase | glycosyl hydrolase 47 family | 3.7 |  | 160 | Metabolic |
| DN1260_c0_g1_i4.g7767.t1 | hypothetical protein FOXG_10681 | S-adenosyl-L-methionine-dependent methyltransferase | 1.5 |  | 364 | Methylation |
| DN1282_c0_g1_i2.g17942.t1 | Vacuolar calcium ion transporter | Sodium/calcium exchanger membrane region | 8.2 |  | 726 | Transport |
| DN12940_c0_g1_i2.g7087.t1 | 54S ribosomal protein L4, mitochondrial | Ribosomal protein L47, mitochondrial | 1.3 |  | 253 | Translation |
| DN13_c1_g1_i1.g8630.t1 | hypothetical protein BFJ65_g9368 | Woronin body major protein | 1.0 |  | 212 | Translation |
| DN1303_c0_g1_i1.g644.t1 | phosphoenolpyruvate carboxykinase | Phosphoenolpyruvate carboxykinase, ATP-utilising | 1.2 |  | 556 | Metabolic |
| DN13202_c0_g1_i1.g19713.t1 | hypothetical protein FOC1_g10010263 | Bystin | 3.4 |  | 439 | Ribosome biogenesis |
| DN13233_c0_g1_i1.g3129.t1 | ATPase | ABC-2 type transporter | 3.3 |  | 547 | Transport |
| DN1325_c0_g1_i1.g593.t1 | hypothetical protein FOC4_g10009406 | U3 small nucleolar RNA-associated SSU processome protein family (Utp8 family) | 3.2 | Cytoplasm | 134 | rRNA processing |
| DN13316_c0_g1_i1.g18283.t1 | hypothetical protein FLONG3_3225, partial | CFEM domain | 1.6 |  | 302 |  |
| DN13359_c0_g1_i2.g18263.t1 | hypothetical protein FOPG_06469 | Eukaryotic rRNA processing | 3.2 |  | 408 | rRNA processing |
| DN13444_c0_g1_i1.g36257.t1 | hypothetical protein BFJ71_g9758 | Conserved hypothetical protein | 3.1 |  | 286 |  |
| DN13464_c0_g1_i1.g8077.t1 | hypothetical protein BFJ65_g4864 | tRNA-dihydrouridine synthase | 4.8 | Cytoplasm | 169 | Oxidation-reduction |
| DN13632_c0_g1_i1.g11626.t1 | Heat shock protein SSB | Heat shock protein 70 family | 5.6 |  | 298 | Stress response |
| DN1365_c0_g1_i3.g5733.t1 | thioredoxin reductase (NADPH) | Pyridine nucleotide-disulphide oxidoreductase | 2.0 |  | 357 | Oxidation-reduction |
| DN13897_c0_g1_i1.g6245.t1 | hypothetical protein BFJ68_g8178 | NAD(P)-binding domain superfamily | 5.8 |  | 133 |  |
| DN13973_c0_g1_i1.g2812.t1 | hypothetical protein BFJ69_g2911 |  | 1.7 |  | 251 |  |
| DN13992_c0_g1_i2.g2797.t1 | putative translation initiation factor eIF-2B subunit epsilon | Nucleotide-diphospho-sugar transferases | 3.4 | Nucleus | 161 | Biosynthetic |
| DN1414_c0_g1_i2.g19591.t1 | probable dihydroxy-acid dehydratase | Dihydroxy-acid dehydratase | 4.3 |  | 575 | Biosynthetic |
| DN14219_c0_g1_i1.g8532.t1 | hypothetical protein BFJ69_g8581 |  | 3.5 |  | 195 |  |
| DN14336_c0_g1_i1.g1537.t1 | hypothetical protein BFJ70_g13205 | Conserved proline-rich protein | 4.5 |  | 312 |  |
| DN14337_c0_g1_i1.g1535.t1 | CHRromatin Organisation MOdifier | Chromo (CHRromatin Organisation MOdifier) domain | 4.8 |  | 261 | Chromatin organisation |
| DN14433_c0_g1_i1.g15060.t1 | hypothetical protein BFJ70_g13134 | Ankyrin repeat-containing domain | 4.3 |  | 115 | Metabolic |
| DN1451_c0_g1_i6.g31116.t1 | Acyl-CoA dehydrogenase family member 11 | Acyl-CoA dehydrogenase/oxidase C-terminal | 5.7 |  | 444 | Oxidation-reduction |
| DN14510_c0_g1_i1.g15750.t1 | hypothetical protein BFJ68_g16126 | Oligopeptide transporter, OPT superfamily | 5.0 |  | 106 | Transport |
| DN14668_c0_g1_i1.g4993.t1 | Pescadillo | Pescadillo | 5.3 |  | 102 | Ribosome biogenesis |
| DN1476_c0_g1_i1.g19410.t1 | hypothetical protein FOXG_04255 | Zinc finger C2H2 superfamily | 1.8 |  | 692 | Transcription |
| DN14882_c0_g1_i1.g4556.t1 | hypothetical protein FOQG_07836 | Armadillo-type fold | 5.5 |  | 530 |  |
| DN1490_c0_g1_i2.g19624.t1 | hypothetical protein BFJ72_g1037 | Zinc finger C2H2 superfamily | 3.0 |  | 429 | Transcription |
| DN1521_c0_g1_i3.g1970.t1 | hypothetical protein FPSE_02938 | Guanine nucleotide-binding (G) domain (Septin-type) | 1.2 |  | 338 | Signal transduction |
| DN1525_c0_g1_i2.g14118.t1 | Guanine nucleotide-binding protein subunit beta-like protein | WD domain, G-beta repeat | 1.6 | Extracellular | 141 | Signal transduction |
| DN15281_c0_g1_i1.g35954.t1 | hypothetical protein FOMG_15433 | NADH:flavin oxidoreductase/NADH oxidase, N-terminal | 3.2 |  | 397 | Oxidation-reduction |
| DN1546_c0_g1_i1.g14028.t1 | Dimethyladenosine transferase | Ribosomal RNA adenine methyltransferase KsgA/Erm | 3.0 |  | 298 | rRNA processing |
| DN1551_c0_g1_i1.g1320.t1 | hypothetical protein FOXG_00490 | DNA polymerase V/Myb-binding protein 1A | 3.3 |  | 669 | Transcription |
| DN15546_c0_g1_i1.g8193.t1 | hypothetical protein BFJ69_g11067 | Fatty acid desaturase domain | 2.9 |  | 269 | Metabolic |
| DN1568_c0_g1_i1.g14301.t1 | hypothetical protein BFJ71_g2440 | RNA-binding domain superfamily | 3.8 |  | 366 |  |
| DN15703_c0_g1_i1.g4581.t1 | metal-nicotianamine transporter ysl11 | Oligopeptide transporter, OPT superfamily | 5.7 |  | 87 | Transport |
| DN15760_c0_g1_i1.g4610.t1 | hypothetical protein BFJ66_g9621 |  | 2.8 |  | 108 |  |
| DN1580_c0_g1_i2.g14141.t1 | hypothetical protein BFJ69_g672 | CFEM domain | 3.0 |  | 596 |  |
| DN15833_c0_g1_i1.g17609.t1 | citrate synthase, mitochondrial | Citrate synthase superfamily | 1.9 |  | 470 | Metabolic |
| DN1586_c0_g1_i2.g14101.t1 | hypothetical protein BFJ65_g7158 | U3 small nucleolar ribonucleoprotein complex, subunit Mpp10 | 2.9 |  | 400 | rRNA processing |
| DN15882_c0_g1_i1.g17600.t1 | regulator of G protein signaling-like protein | RGS, regulator of G protein signaling domain | 4.4 |  | 85 | Signal transduction |
| DN15931_c0_g1_i1.g3170.t1 | related to RGD2-GTPase activating protein | GTPase-activating protein domain (Rho) | 1.6 |  | 548 | Signal transduction |
| DN15990_c0_g1_i1.g3193.t1 | RNA cytidine acetyltransferase | Acetyltransferase (GNAT) domain | 4.5 | Nucleus | 162 | Biosynthetic |
| DN16050_c0_g1_i1.g28671.t1 | hypothetical protein BFJ71_g7880 |  | 3.7 | Extracellular | 129 |  |
| DN161_c0_g1_i1.g18857.t1 | Endonuclease III like protein | HhH-GPD domain | 3.8 |  | 395 |  |
| DN1610_c0_g1_i3.g26909.t1 | hypothetical protein FOQG_12898 | NmrA-like domain | 2.8 |  | 329 |  |
| DN16102_c0_g1_i1.g11473.t1 | hypothetical protein BFJ71_g1880 | Lsm14-like, N-terminal | 1.4 |  | 556 |  |
| DN16496_c0_g1_i1.g8055.t1 | hypothetical protein BFJ65_g13903 | Pectate lyase PlyH/PlyE-like | 4.9 |  | 232 | Metabolism |
| DN16540_c0_g1_i1.g5755.t1 | 25S rRNA (adenine-N (1))-methyltransferase | Ribosomal RNA processing protein 8 | 2.5 |  | 365 | RNA processing |
| DN16652_c0_g1_i1.g16050.t1 | tRNA-dihydrouridine synthase 1 | tRNA-dihydrouridine synthase | 4.7 |  | 120 | Oxidation-reduction |
| DN1667_c0_g1_i2.g17108.t1 | D-arabinono-1,4-lactone oxidase | D-arabinono-1,4-lactone oxidase | 1.5 |  | 514 | Oxidation-reduction |
| DN16825_c0_g1_i1.g12586.t1 | hypothetical protein BFJ71_g5446 |  | 6.9 |  | 110 |  |
| DN16875_c0_g1_i1.g7575.t1 | hypothetical protein BFJ70_g3725 |  | 4.5 |  | 116 |  |
| DN1698_c0_g1_i1.g26715.t1 | hypothetical protein FOPG_06852 | bZIP_YAP | 3.0 |  | 513 | Transcription |
| DN17043_c0_g1_i1.g3664.t1 | hypothetical protein BFJ71_g3363 | Gar1/Naf1 RNA binding region | 1.9 |  | 222 |  |
| DN17242_c0_g1_i1.g16873.t1 | probable L-alanine-DL-glutamate epimerase and related enzymes of enolase superfamily | Enolase C-terminal domain-like | 3.4 |  | 188 |  |
| DN17342_c0_g1_i1.g9299.t1 | hypothetical protein FPSE_05918 | Carbamoyl-phosphate synthase large subunit, CPSase domain | 4.5 | Cytoplasm | 145 | Metabolic |
| DN1737_c0_g1_i2.g13214.t1 | Diphthine methyl ester synthase | Tetrapyrrole methylase, subdomain 2 | 3.7 |  | 130 |  |
| DN17440_c0_g1_i1.g35662.t1 | hypothetical protein BFJ69_g6218 |  | 4.7 |  | 382 |  |
| DN17463_c0_g1_i1.g295.t1 | hypothetical protein BFJ69_g14666 | Cytochrome P450 | 4.7 |  | 106 | Oxidation-reduction |
| DN1754_c0_g1_i6.g13115.t1 | hypothetical protein FOQG_03847 | WD domain, G-beta repeat | 4.3 |  | 238 | Signal transduction |
| DN1766_c0_g1_i2.g23659.t1 | Carbamoyl-phosphate synthase arginine-specific large chain | Carbamoyl-phosphate synthetase large subunit-like, ATP-binding domain | 1.1 |  | 1175 | Metabolic |
| DN18234_c0_g1_i1.g339.t1 | related to POX1-acyl-CoA oxidase | Acyl-CoA dehydrogenase-like, C-terminal | 3.2 | Peroxisome | 213 | Oxidation-reduction |
| DN18276_c0_g1_i2.g319.t1 | hypothetical protein BFJ70_g8234 | von Willebrand factor, type A | 2.1 |  | 808 |  |
| DN18345_c0_g1_i1.g13375.t1 | hypothetical protein BFJ69_g9743 | KRR1 interacting protein 1 | 2.4 |  | 382 |  |
| DN18415_c0_g1_i1.g13438.t1 | Multisite-specific tRNA:(cytosine-C(5))-methyltransferase | 16S rRNA methyltransferase | 4.8 | Mitochondrion | 85 | Methylation |
| DN1865_c0_g1_i1.g10054.t1 | phosphoribosylaminoimidazole carboxylase | ATP-grasp fold, ATP-dependent carboxylate-amine ligase-type | 1.7 |  | 408 | Biosynthetic |
| DN18803_c0_g1_i1.g1519.t1 | hypothetical protein BFJ70_g935 | Major facilitator, sugar transporter-like | 5.4 |  | 98 | Transport |
| DN1885_c0_g1_i1.g10284.t1 | hypothetical protein BFJ69_g7080 | Membrane insertase YidC/ALB3/OXA1/COX18 | 1.1 |  | 444 |  |
| DN1900_c0_g2_i1.g7284.t1 | hypothetical protein FOC4_g10009109 | Haemerythrin-like | 2.7 |  | 188 |  |
| DN1928_c0_g1_i4.g31798.t1 | hypothetical protein FOC4_g10012971 | K Homology domain, type 1 superfamily | 4.7 |  | 478 |  |
| DN1935_c0_g1_i4.g7163.t1 | Fatty acid synthase subunit alpha | 4'-phosphopantetheinyl transferase domain | 2.4 |  | 1123 | Biosynthetic |
| DN19401_c0_g1_i1.g3030.t1 | hypothetical protein FOQG_02920 | Peptidase M20, dimerisation domain | 3.3 |  | 285 | Proteolysis |
| DN1942_c0_g1_i3.g7259.t1 | hypothetical protein BFJ68_g10243 | DNA polymerase V/Myb-binding protein 1A | 3.3 |  | 648 | Transcription |
| DN19472_c0_g1_i1.g3031.t1 | hypothetical protein FOQG_12024 | AARP2CN | 3.8 |  | 145 | Ribosome biogenesis |
| DN19537_c0_g1_i2.g618.t1 | Long chain acyl-CoA synthetase 7, peroxisomal | AMP-dependent synthetase/ligase | 2.2 |  | 623 |  |
| DN1972_c0_g1_i2.g7266.t1 | hypothetical protein BFJ66_g6321 | ATPase assembly factor ATP10 | 2.1 |  | 263 | Transport |
| DN19908_c0_g1_i1.g17503.t1 | phospholipase D | Phospholipase D/Transphosphatidylase | 3.1 |  | 444 | Catabolic |
| DN20017_c0_g1_i1.g2698.t1 | hypothetical protein FOMG_08000 | NUC153 domain | 4.4 |  | 119 | rRNA processing |
| DN20343_c0_g1_i1.g13963.t1 | Calcium-transporting ATPase 2 | P-type ATPase, cytoplasmic domain N | 4.0 |  | 233 | Transport |
| DN20674_c0_g1_i1.g35479.t1 | 1-aminocyclopropane-1-carboxylate synthase-like protein 1 | Aminotransferase, class I/classII | 7.1 |  | 131 | Biosynthetic |
| DN20848_c0_g1_i1.g28679.t1 | PelD, partial | Pectate lyase PlyH/PlyE-like | 5.3 | Extracellular | 112 | Metabolic |
| DN21093_c0_g1_i1.g18317.t1 | Elongation factor G | Translation elongation factor EFG/EF2 | 2.3 |  | 711 | Translation |
| DN211_c0_g1_i7.g10767.t1 | hypothetical protein FOQG_02455 | biological adhesion | 4.1 |  | 493 |  |
| DN21251_c0_g1_i1.g4547.t1 | hypothetical protein BFJ72_g144 | Major facilitator, sugar transporter-like | 4.4 |  | 146 | Transport |
| DN213_c0_g2_i1.g10793.t1 | aspartate aminotransferase, mitochondrial | Aminotransferase, class I/classII | 2.6 |  | 424 | Biosynthetic |
| DN2142_c0_g1_i1.g6837.t1 | hypothetical protein FOXG_01596 | rRNA-processing protein Fcf1/Utp23 | 1.8 |  | 198 | rRNA processing |
| DN21437_c0_g1_i1.g5389.t1 | hypothetical protein BFJ68_g16108 | carbohydrate-binding module family 19 protein | 2.2 |  | 819 | Metabolic |
| DN21472_c0_g1_i1.g5318.t1 | tyrosyl-tRNA synthetase | Tyrosine-tRNA ligase | 1.2 |  | 615 |  |
| DN21576_c0_g1_i1.g10819.t1 | Flavohemoprotein | Ferredoxin-NADP reductase (FNR), nucleotide-binding domain | 4.5 |  | 254 |  |
| DN21599_c0_g1_i1.g3187.t1 | 25S rRNA (cytosine-C (5))-methyltransferase nop2 | 16S rRNA methyltransferase | 4.9 |  | 163 | Methylation |
| DN21672_c0_g1_i1.g507.t1 | hypothetical protein BFJ65_g15717 | Amine oxidase | 6.3 |  | 126 | Oxidation-reduction |
| DN2180_c0_g1_i1.g6965.t1 | hypothetical protein FOXG_07705 | protein serine/threonine kinase activity | 3.1 |  | 265 |  |
| DN2246_c0_g1_i1.g10672.t1 | Putative pre-mRNA-splicing factor ATP-dependent RNA helicase prp43 | Helicase-associated domain | 3.7 |  | 574 | Chromatin organization |
| DN2256_c0_g1_i5.g10617.t1 | Adenylosuccinate synthetase | Adenylosuccinate synthetase | 1.1 |  | 417 | Biosynthetic |
| DN22580_c0_g1_i1.g17523.t1 | Putative quinate permease | major facilitator superfamily | 5.8 |  | 81 | Transport |
| DN22984_c0_g1_i1.g4604.t1 | hypothetical protein BFJ69_g15711 | Putative FMN-binding domain | 3.9 |  | 91 |  |
| DN2333_c0_g1_i1.g3837.t1 | glucose-6-phosphate/phosphate translocator | Sugar phosphate transporter domain | 1.9 |  | 369 | Transport |
| DN2340_c0_g1_i1.g3912.t1 | probable GFA1-glucosamine--fructose-6-phosphate transaminase | Sugar isomerase (SIS) | 1.0 |  | 699 | Metabolic |
| DN23519_c0_g1_i1.g3771.t1 | hypothetical protein BFJ69_g3962 | Methyltransferase domain 25 | 1.1 |  | 437 | Methylation |
| DN23603_c0_g1_i1.g19739.t1 | U3 small nucleolar RNA-associated protein 10 | BP28CT (NUC211) domain | 4.3 |  | 187 |  |
| DN23785_c0_g1_i1.g11082.t1 | Maltose permease MAL31 | Major facilitator, sugar transporter-like | 6.1 |  | 117 | Transport |
| DN23911_c0_g1_i1.g17974.t1 | uncharacterized protein FPRN_09263 |  | 1.0 |  | 505 |  |
| DN24_c0_g2_i1.g8975.t1 | hypothetical protein FOXG_09777 | RNA recognition motif domain | 2.2 |  | 112 | Cytokinesis |
| DN24041_c0_g1_i1.g3426.t1 | Pre-rRNA-processing protein esf1 | NUC153 domain | 4.2 |  | 182 | rRNA processing |
| DN2410_c0_g1_i1.g17770.t1 | hypothetical protein BFJ68_g9002 | R3H domain | 2.2 |  | 340 |  |
| DN2412_c0_g1_i1.g17734.t1 | DNA-directed RNA polymerase, mitochondrial | DNA-directed RNA polymerase, phage-type | 1.6 |  | 715 | Transcription |
| DN24377_c0_g1_i1.g17952.t1 | 25S rRNA (cytosine (2870)-C (5))-methyltransferase | RNA (C5-cytosine) methyltransferase, NOP2 | 3.4 |  | 463 | Methylation |
| DN2487_c0_g1_i1.g17732.t1 | hypothetical protein FOXG_01669 | Mitochondrial carrier protein | 4.0 |  | 695 | Transport |
| DN24903_c0_g1_i1.g14496.t1 | hypothetical protein BFJ72_g3096 | Type III restriction enzyme, res subunit | 3.5 |  | 107 |  |
| DN2504_c0_g1_i5.g4758.t1 | fumarate hydratase, mitochondrial | Fumarate hydratase, class II | 1.5 |  | 529 | Metabolic |
| DN25042_c0_g1_i1.g12970.t1 | flavohemoglobin | Globin/Protoglobin | 5.8 |  | 159 | Transport |
| DN2516_c0_g1_i1.g4899.t1 | ATP-dependent RNA helicase MAK5 | P-loop containing nucleoside triphosphate hydrolase | 1.4 |  | 565 | Phosphorylation |
| DN2534_c0_g1_i2.g4711.t1 | Periodic tryptophan protein 2 like protein | WD domain, G-beta repeat | 3.0 |  | 321 | Signal transduction |
| DN2536_c0_g1_i1.g4864.t1 | hypothetical protein FPSE_12273, partial | H/ACA ribonucleoprotein complex, subunit Gar1/Naf1 | 3.1 |  | 210 |  |
| DN25373_c0_g1_i1.g18002.t1 | Para-nitrobenzyl esterase | Carboxylesterase, type B | 5.3 | Extracellular | 120 |  |
| DN2560_c0_g1_i7.g19981.t1 | hypothetical protein BFJ65_g129 |  | 5.4 |  | 380 |  |
| DN2597_c0_g1_i3.g19860.t1 | carnitine O-acetyltransferase | Choline/Carnitine o-acyltransferase, domain 2 | 1.1 |  | 610 | Proteolysis |
| DN2728_c0_g1_i1.g14680.t1 | hypothetical protein BFJ72_g5893 | Zinc finger C2H2 superfamily | 1.3 |  | 493 | Transcription |
| DN2784_c0_g1_i2.g16586.t1 | hypothetical protein FOTG_01069 | Arf3-interacting protein 1, N-terminal domain | 2.1 |  | 845 |  |
| DN2847_c0_g1_i1.g13851.t1 | RNA 3'-terminal phosphate cyclase | RNA 3'-terminal phosphate cyclase domain | 6.1 |  | 400 | Ribosome biogenesis |
| DN2865_c0_g1_i2.g33427.t1 | hypothetical protein FOTG_13797 | ZN598/Hel2, RING finger | 6.4 |  | 763 | Transcription |
| DN290_c0_g1_i9.g9764.t1 | hypothetical protein FOPG_11896 | Heat shock protein DnaJ, cysteine-rich domain | 5.2 |  | 396 | Stress response |
| DN2907_c0_g1_i1.g19265.t1 | hypothetical protein FOXG_10023 | Sodium/calcium exchanger membrane region | 3.1 |  | 530 | Transport |
| DN293_c0_g1_i4.g5401.t1 | hypothetical protein FOQG_10642 | Acetyltransferase (GNAT) domain | 5.8 |  | 622 | Biosynthetic |
| DN2939_c0_g1_i1.g3356.t1 | hypothetical protein FOXG_18156 | Glucose-repressible protein Grg1 | 6.1 |  | 69 |  |
| DN3072_c0_g1_i2.g12282.t1 | hypothetical protein BFJ69_g6143 | Pre-rRNA-processing protein RIX1, N-terminal | 2.2 |  | 648 | Ribosome biogenesis |
| DN308_c0_g1_i3.g15242.t1 | hypothetical protein FOMG_01413 | Zn (2)-C6 fungal-type DNA-binding domain-GAL4 | 7.0 |  | 726 | Transcription |
| DN314_c0_g1_i5.g12836.t1 | AdoMet-dependent rRNA methyltransferase SPB1 | Ribosomal RNA methyltransferase, Spb1, C-terminal | 3.3 |  | 564 | Methylation |
| DN3191_c0_g1_i1.g26.t1 | hypothetical protein BFJ72_g13593 | U3 small nucleolar RNA-associated SSU processome protein (Utp14 protein) | 2.6 |  | 437 | rRNA processing |
| DN3286_c0_g1_i3.g34114.t1 | hypothetical protein BFJ71_g15301 | Tetratricopeptide repeat | 2.9 |  | 1167 | RNA processing |
| DN3316_c0_g1_i1.g19583.t1 | Putative methylcrotonoyl-CoA carboxylase beta chain, mitochondrial | Acetyl-CoA carboxylase | 2.4 |  | 542 | Biosynthetic |
| DN3335_c0_g1_i1.g16728.t1 | hypothetical protein FOXG_01546 | Chaperone J-domain superfamily | 1.4 |  | 408 | Stress response |
| DN3342_c0_g1_i1.g16670.t1 | threonyl-tRNA synthetase | Threonine-tRNA ligase, class IIa | 2.3 |  | 745 | Threonyl-tRNA aminoacylation |
| DN3395_c0_g1_i2.g16822.t1 | hypothetical protein FOCG_01077 | Homeobox domain | 1.8 |  | 529 |  |
| DN3403_c0_g1_i1.g10902.t1 | hypothetical protein BFJ69_g5063 | Transcription factor domain, fungi | 5.0 |  | 351 | Transcription |
| DN3435_c0_g1_i2.g24733.t1 | hypothetical protein BFJ65_g11918 | G protein-coupled receptor GPR1, C-terminal | 4.2 |  | 561 | Signal transduction |
| DN345_c0_g1_i2.g14821.t1 | Zinc finger protein rsv2 | Zinc finger C2H2 superfamily | 3.8 |  | 602 | Transcription |
| DN3496_c0_g1_i1.g10965.t1 | UDP-glucose 6-dehydrogenase | UDP-glucose/GDP-mannose dehydrogenase, N-terminal | 2.1 |  | 605 | Oxidation-reduction |
| DN3508_c0_g1_i1.g15987.t1 | chromatin modification-like protein VID21 | Helicase/SANT-associated domain | 3.0 |  | 582 | Chromatin organization |
| DN352_c0_g1_i1.g12466.t1 | probable RPS16B-ribosomal protein S16.e | Ribosomal protein S9 | 2.6 | Cytoplasm | 142 | Translation |
| DN3542_c0_g1_i1.g16020.t1 | 30S ribosomal protein S2 | Ribosomal protein S2 | 1.5 |  | 521 | Translation |
| DN38_c1_g1_i2.g8917.t1 | hypothetical protein BFJ69_g14063 | extracellular protein | 1.1 |  | 431 |  |
| DN3808_c0_g1_i2.g24978.t1 | Mps one binder kinase activator-like 4 | MOB kinase activator family | 5.0 |  | 497 | Signal transduction |
| DN3846_c0_g1_i1.g4083.t1 | probable FPR3-prolyl cis-trans isomerase | FKBP-type peptidyl-prolyl cis-trans isomerase domain | 4.0 |  | 463 |  |
| DN3890_c0_g1_i1.g4142.t1 | Metacaspase-1B | Caspase domain | 1.5 |  | 414 |  |
| DN3924_c0_g1_i1.g2663.t1 | hypothetical protein FOXG_08098 | Ribosomal RNA-processing protein 14/surfeit locus protein 6, C-terminal domain | 2.3 |  | 522 | RNA processing |
| DN4013_c0_g1_i2.g7411.t1 | hypothetical protein FOQG_05630 | tRNA methyltransferase, Trm1 | 2.7 |  | 307 | tRNA processing |
| DN4022_c0_g1_i1.g7427.t1 | hypothetical protein FOMG_14220 | Growth factor receptor cysteine-rich domain superfamily | 3.2 | Extracellular | 174 |  |
| DN4071_c0_g1_i1.g7482.t1 | hypothetical protein BFJ69_g2367 |  | 1.4 |  | 392 |  |
| DN4098_c0_g1_i1.g7426.t1 | hypothetical protein FOXG_01850 | P-loop containing nucleoside triphosphate hydrolase | 2.0 |  | 578 | Phosphorylation |
| DN4150_c0_g1_i1.g18165.t1 | hypothetical protein BFJ69_g14907 | Pentacotripeptide-repeat region of PRORP | 3.8 | Mitochondrion | 151 |  |
| DN4217_c0_g1_i1.g14839.t1 | Aldehyde dehydrogenase | Aldehyde dehydrogenase domain | 5.8 |  | 493 | Oxidation-reduction |
| DN4220_c0_g1_i1.g13973.t1 | hypothetical protein FOXG_02155 | BP28, C-terminal domain | 3.7 |  | 856 |  |
| DN4280_c0_g1_i1.g14148.t1 | Succinate/fumarate mitochondrial transporter | Mitochondrial substrate/solute carrier | 5.2 | Peroxisome | 174 | Transport |
| DN4292_c0_g1_i1.g14869.t1 | hypothetical protein BFJ69_g9668 | Pentatricopeptide repeat | 1.8 |  | 374 |  |
| DN4295_c0_g1_i1.g14014.t1 | hypothetical protein BFJ69_g8557 | Conserved proline-rich protein | 5.2 |  | 388 |  |
| DN4382_c0_g1_i1.g17488.t1 | Repressible high-affinity phosphate permease | Major facilitator, sugar transporter-like | 4.3 |  | 144 | Transport |
| DN4405_c0_g1_i2.g14432.t1 | hypothetical protein FOQG_00936 | Pseudouridine synthase, TruD, catalytic domain | 2.1 |  | 742 | RNA modification |
| DN443_c0_g1_i3.g12271.t1 | translation initiation factor 2 subunit 2 | Translation initiation factor IF2/IF5 | 2.0 |  | 316 | Translation |
| DN4471_c0_g1_i1.g4856.t1 | hypothetical protein BFJ69_g3040 | Fungal chitosanase | 3.8 |  | 172 |  |
| DN4492_c0_g1_i1.g14411.t1 | hypothetical protein FOC4_g10001156 | chromatin remodelling complex protein | 1.0 |  | 358 | Chromatin organisation |
| DN4513_c0_g1_i1.g11546.t1 | hypothetical protein FOQG_02770 | CP2 transcription factor | 1.0 |  | 788 | Transcription |
| DN4524_c0_g1_i1.g11568.t1 | hypothetical protein FOTG_07367 |  | 1.8 |  | 750 |  |
| DN4632_c0_g1_i1.g25873.t1 | hypothetical protein FOIG_00995 | Armadillo-type fold | 2.2 |  | 809 |  |
| DN4643_c0_g1_i2.g17305.t1 | hypothetical protein BFJ69_g1022 | DNA-directed RNA polymerase I, subunit RPA34.5 | 2.7 |  | 322 | Transcription |
| DN4656_c0_g1_i1.g17314.t1 | hypothetical protein FOC1_g10010105 | Tetratricopeptide repeat | 2.6 |  | 874 | RNA processing |
| DN4658_c0_g1_i2.g25791.t1 | hypothetical protein BFJ65_g7352 |  | 6.2 | Cytoplasm | 151 |  |
| DN4679_c0_g1_i1.g25796.t1 | ISWI chromatin-remodelling complex ATPase ISW2 | SNF2-related, N-terminal domain | 3.1 |  | 420 |  |
| DN4734_c0_g1_i1.g19129.t1 | hypothetical protein FOPG_08517 | Coenzyme A transferase family I | 2.5 |  | 451 | Catabolic |
| DN4787_c0_g1_i1.g3089.t1 | hypothetical protein BFJ72_g553 | Fatty acid desaturase domain | 8.9 |  | 376 | Metabolic |
| DN4871_c0_g1_i2.g26102.t1 | hypothetical protein FOMG_10229 | AARP2CN (NUC121) domain | 4.5 |  | 166 | Ribosome biogenesis |
| DN4875_c0_g1_i2.g26106.t1 | related to levodione reductase | NAD(P)-binding domain superfamily | 3.4 |  | 251 |  |
| DN4917_c0_g1_i1.g24869.t1 | uncharacterized protein FFUJ_01666 | Homeobox-like domain superfamily | 2.0 |  | 467 |  |
| DN4984_c0_g1_i1.g15159.t1 | hypothetical protein FOXG_12803 | Heat shock factor (HSF)-type, DNA-binding | 4.0 |  | 414 | Transcription |
| DN5064_c0_g1_i1.g12052.t1 | hypothetical protein FOC4_g10008504 | Mitochondrial ribosomal protein MRP51, fungi | 1.4 |  | 364 | Translation |
| DN5073_c0_g1_i1.g12019.t1 | chromodomain-helicase-DNA-binding protein 1 | Domain of unknown function DUF4208 | 1.9 |  | 607 |  |
| DN5107_c0_g1_i2.g15345.t1 | hypothetical protein FOC4_g10012609 | Pyridine nucleotide-disulphide oxidoreductase | 3.5 |  | 515 | Oxidation-reduction |
| DN518_c0_g1_i1.g1907.t1 | hypothetical protein FGSG_05999 | Ribosomal protein L27e | 3.0 |  | 135 | Translation |
| DN5184_c0_g1_i1.g4682.t1 | methyltransferase | tRNA (C5-cytosine) methyltransferase, NCL1 | 3.0 |  | 852 | tRNA methylation |
| DN526_c0_g1_i4.g2244.t1 | rRNA methyltransferase 1, mitochondrial | tRNA/rRNA methyltransferase, SpoU type | 2.2 |  | 206 | RNA processing |
| DN5291_c0_g1_i1.g15033.t1 | probable zuotin | Chaperone DnaJ-domain superfamily | 3.4 |  | 430 | Stress response |
| DN5293_c0_g1_i1.g15048.t1 | hypothetical protein FOC1_g10001023 |  | 2.8 |  | 195 |  |
| DN53_c0_g1_i3.g8821.t1 | hypothetical protein BFJ69_g2667 | Zn (2)-C6 fungal-type DNA-binding domain-GAL4 | 1.2 |  | 779 | Transcription |
| DN53_c0_g1_i4.g8819.t1 | L-aminoadipate-semialdehyde dehydrogenase | Amino acid adenylation domain | 1.9 |  | 1185 | Biosynthetic |
| DN5316_c0_g1_i1.g8321.t1 | tRNA (adenine (58)-N (1))-methyltransferase non-catalytic subunit trm6 | tRNA (adenine (58)-N (1))-methyltransferase non-catalytic subunit TRM6 | 2.7 |  | 283 | tRNA methylation |
| DN5325_c0_g1_i1.g3308.t1 | hypothetical protein FOMG_06847 | Pentatricopeptide repeat | 2.0 |  | 530 |  |
| DN5332_c0_g1_i1.g3239.t1 | Uncharacterized protein LW93_8144 | Major facilitator, sugar transporter-like | 4.2 |  | 260 | Transport |
| DN5400_c0_g1_i1.g24456.t1 | hypothetical protein BFJ69_g8623 |  | 3.4 |  | 255 |  |
| DN5425_c0_g1_i1.g24486.t1 | hypothetical protein FOTG_07624 | RNA (C5-cytosine) methyltransferase, NOP2 | 3.4 |  | 515 | Methylation |
| DN5496_c0_g1_i2.g3026.t1 | Bifunctional cytochrome P450/NADPH--P450 reductase | Cytochrome P450 | 3.4 |  | 320 | Oxidation-reduction |
| DN5699_c0_g1_i1.g2869.t1 | Putative WD repeat-containing protein C17D11.16 | WD domain, G-beta repeat | 4.7 |  | 115 | Signal transduction |
| DN5712_c0_g1_i1.g14562.t1 | Midasin |  | 3.7 |  | 196 |  |
| DN5713_c0_g1_i1.g14580.t1 | related to nuclear WD protein PRL1 | Smr domain | 2.5 |  | 233 |  |
| DN5768_c0_g1_i1.g33186.t1 | Protein transport protein SEC7 | Sec7 domain | 5.0 |  | 1658 | Signal transduction |
| DN5860_c0_g1_i1.g18735.t1 | hypothetical protein BFJ69_g6500 | Acyl-CoA dehydrogenase/oxidase C-terminal | 2.6 |  | 424 | Oxidation-reduction |
| DN5880_c0_g1_i1.g18771.t1 | Elongator complex protein 1 | Elongator complex protein 1 | 2.8 |  | 548 | tRNA wobble uridine modification |
| DN590_c0_g1_i2.g2064.t1 | succinyl-CoA ligase | ATP-grasp fold, succinyl-CoA synthetase-type | 1.1 |  | 447 | Metabolic |
| DN5969_c0_g1_i2.g1842.t1 | hypothetical protein BFJ70_g8898 | NADP-dependent leukotriene b4 12-hydroxydehydrogenase | 4.8 | Mitochondrion | 114 | Oxidation-reduction |
| DN5993_c0_g1_i3.g1839.t1 | Uncharacterized protein LW93_1282 | MmgE/PrpD | 2.2 |  | 464 |  |
| DN607_c0_g1_i33.g6144.t1 | hypothetical protein FGSG_09933 | Porin, eukaryotic type | 1.0 |  | 283 | Transport |
| DN611_c0_g1_i16.g8522.t1 | hypothetical protein FOIG_04816 | Ribosomal protein L4/L1e | 1.0 |  | 320 | Translation |
| DN6143_c0_g1_i1.g19846.t1 | hypothetical protein FOIG_07700 |  | 2.6 |  | 206 |  |
| DN6173_c0_g1_i1.g19841.t1 | hypothetical protein FOMG_13266 | Tyrosinase copper-binding domain | 3.5 |  | 266 | Oxidation-reduction |
| DN6183_c0_g1_i1.g16926.t1 | hypothetical protein FOTG_00827 | WD domain, G-beta repeat | 2.6 |  | 824 | Signal transduction |
| DN6223_c0_g1_i1.g13529.t1 | hypothetical protein BFJ69_g8667 | Ankyrin repeat-containing domain superfamily | 3.5 |  | 142 | Metabolic |
| DN6354_c0_g1_i2.g2757.t1 | related to arsenate reductase (Arc2) | Rhodanese-like domain superfamily | 4.6 |  | 59 |  |
| DN6364_c0_g1_i1.g2788.t1 | hypothetical protein BFJ72_g9221 | NAP-like superfamily | 6.7 |  | 104 |  |
| DN6368_c0_g1_i1.g2795.t1 | hypothetical protein FOC1_g10002935 | FAD dependent oxidoreductase | 3.3 |  | 495 | Oxidation-reduction |
| DN6468_c0_g1_i1.g13420.t1 | hypothetical protein BFJ69_g7034 | Carbohydrate esterase 2, N-terminal | 3.1 |  | 319 |  |
| DN653_c0_g1_i1.g8157.t1 | hypothetical protein FPSE_01587 | CFEM domain | 2.9 |  | 287 |  |
| DN6659_c0_g1_i1.g14921.t1 | hypothetical protein FOQG_01266 | Mitochondrial inner-membrane-bound regulator | 3.1 |  | 435 |  |
| DN6777_c0_g1_i2.g17217.t1 | Annexin A11 | Annexin repeat | 3.7 |  | 423 |  |
| DN6994_c0_g1_i1.g2739.t1 | hypothetical protein FOMG_10134 | RNA polymerase Rpb7-like, N-terminal domain superfamily | 3.2 |  | 338 | Transcription |
| DN6996_c0_g1_i1.g33658.t1 | hypothetical protein FOQG_00710 | Peptidase C19, ubiquitin carboxyl-terminal hydrolase | 2.5 |  | 1259 | Proteolysis |
| DN7065_c0_g1_i6.g2427.t1 | Amidophosphoribosyltransferase | Glutamine amidotransferase type 2 domain | 4.5 | Cytoplasm | 224 | Metabolic |
| DN7104_c0_g1_i1.g13265.t1 | Protein kri1 | KRI1-like family | 4.6 |  | 144 |  |
| DN7159_c0_g1_i1.g2644.t1 | hypothetical protein FOPG_10692 | G protein-coupled receptor GPR1, C-terminal | 3.1 |  | 264 | Signal transduction |
| DN7200_c0_g1_i2.g15837.t1 | hypothetical protein BFJ72_g6692 | GLEYA adhesin domain | 3.1 | Extracellular | 178 |  |
| DN727_c0_g1_i2.g15605.t1 | hypothetical protein FPSE_08712 | Ribosomal protein S19e | 1.9 |  | 150 | Translation |
| DN737_c0_g1_i2.g4128.t1 | hypothetical protein FOXG_00544 | Tetratricopeptide repeat | 6.1 |  | 338 | RNA processing |
| DN7394_c0_g1_i1.g13992.t1 | UTP-ammonia ligase, partial | CTP synthase, N-terminal | 4.5 | Cytoplasm | 133 | Biosynthetic |
| DN7571_c0_g1_i2.g9423.t1 | hypothetical protein FOC1_g10004629 | CFEM domain | 3.7 |  | 457 |  |
| DN7768_c0_g1_i1.g13686.t1 | hypothetical protein FOIG_08866 | RNA recognition motif domain | 3.4 |  | 549 | Cytokinesis |
| DN779_c0_g1_i1.g15875.t1 | hypothetical protein FOTG_02856 | WD domain, G-beta repeat | 2.6 |  | 348 | Signal transduction |
| DN780_c0_g1_i2.g15871.t1 | hypothetical protein FOQG_06352 | PAS domain | 2.9 |  | 658 |  |
| DN782_c0_g1_i1.g15540.t1 | hypothetical protein BFJ72_g8406 | RNA-binding domain superfamily | 2.7 |  | 314 |  |
| DN7823_c0_g1_i1.g11133.t1 | rRNA biogenesis protein rrp5 | S1 domain | 5.3 |  | 346 |  |
| DN7905_c0_g1_i2.g13930.t1 | DNA-directed RNA polymerase I subunit rpa49 | RNA polymerase I associated factor, A49-like | 3.0 |  | 388 | Transcription |
| DN793_c0_g1_i1.g15501.t1 | Serine hydroxymethyltransferase, cytosolic | Serine hydroxymethyltransferase | 1.7 |  | 468 | Tetrahydrofolate interconversion |
| DN7994_c0_g1_i1.g13912.t1 | Low-affinity potassium transport protein | potassium transport family | 2.6 |  | 442 | Transport |
| DN802_c0_g1_i6.g22965.t1 | probable phosphoprotein phosphatase (serine/threonine specific protein phosphatase) | Tetratricopeptide repeat | 2.3 |  | 472 | RNA processing |
| DN8060_c0_g1_i1.g18584.t1 | Phosphate-repressible phosphate permease | Phosphate transporter | 7.2 |  | 195 | Transport |
| DN8090_c0_g1_i1.g12385.t1 | hypothetical protein FOC1_g10010125 | PAS domain | 4.8 |  | 343 |  |
| DN8101_c0_g1_i1.g5042.t1 | hypothetical protein BFJ71_g6046 | Dihydrouridine synthase (Dus) | 4.2 |  | 121 | tRNA processing |
| DN8294_c0_g1_i1.g18469.t1 | hypothetical protein FOC4_g10014708 | Six-hairpin glycosidase-like superfamily | 2.4 |  | 580 |  |
| DN8341_c0_g1_i1.g36342.t1 | Putative enoyl-CoA hydratase, mitochondrial | Enoyl-CoA hydratase/isomerase | 3.2 |  | 119 | Catabolic |
| DN837_c0_g1_i6.g16144.t1 | hypothetical protein FOC1_g10006776 | Ribosomal protein L50, mitochondria | 2.3 |  | 380 | Translation |
| DN8391_c0_g1_i1.g9171.t1 | hypothetical protein FOQG_03246 | P-loop containing nucleoside triphosphate hydrolase | 1.9 |  | 459 | Phosphorylation |
| DN8425_c0_g1_i1.g8126.t1 | hypothetical protein FOC4_g10010006 | Major intrinsic protein | 3.3 |  | 175 | Transport |
| DN8507_c0_g1_i1.g242.t1 | hypothetical protein BFJ69_g1986 | NADH:flavin oxidoreductase/NADH oxidase, N-terminal | 3.6 |  | 330 | Oxidation-reduction |
| DN8608_c0_g1_i1.g15362.t1 | hypothetical protein BFJ68_g9508 | Aspartic peptidase A1 family | 3.4 |  | 242 | Proteolysis |
| DN8652_c0_g1_i1.g31305.t1 | hypothetical protein FOC4_g10003626 | Acyl-CoA N-acyltransferase | 5.3 | Mitochondrion | 140 | Biosynthetic |
| DN868_c0_g1_i1.g16277.t1 | hypothetical protein FOXG_08701 | WD domain, G-beta repeat | 1.1 |  | 484 | Signal transduction |
| DN8942_c0_g1_i1.g420.t1 | hypothetical protein FPSE_12049 | Heat shock protein 70 family | 6.8 |  | 79 | Stress response |
| DN897_c0_g1_i2.g16201.t1 | hypothetical protein BFJ72_g12283 | Nascent polypeptide-associated complex NAC domain | 2.1 |  | 154 | Transport |
| DN9027_c0_g1_i2.g2682.t1 | hypothetical protein BFJ69_g150 | Glycosyl hydrolase family 32, C-terminal | 6.5 |  | 149 | Metabolic |
| DN9228_c0_g1_i1.g16302.t1 | Polyamine oxidase | Amine oxidase | 6.5 |  | 180 | Oxidation-reduction |
| DN9229_c0_g1_i3.g16322.t1 | hypothetical protein FOCG_16049 | Flavoprotein-like superfamily | 6.3 |  | 265 |  |
| DN940_c0_g1_i1.g32092.t1 | Phospholipase D1 | Phospholipase D/Transphosphatidylase | 8.6 |  | 1475 | Catabolic |
| DN9622_c0_g1_i1.g10530.t1 | putative isoamyl alcohol oxidase | Berberine/berberine-like | 4.0 |  | 295 | Oxidation-reduction |
| DN977_c0_g1_i2.g6321.t1 | hypothetical protein BFJ65_g6222 | bZIP_YAP | 3.1 |  | 392 | Transcription |
| DN9778_c0_g1_i1.g9267.t1 | hypothetical protein FOQG_06889 | RNA 2-O ribose methyltransferase, substrate binding | 4.7 |  | 492 | Methylation |
| DN9788_c0_g1_i1.g9247.t1 | hypothetical protein BFJ69_g101 | NADH:flavin oxidoreductase/NADH oxidase, N-terminal | 3.3 |  | 394 | Oxidation-reduction |
| DN992_c0_g1_i1.g6531.t1 | hypothetical protein FOPG_19920, partial |  | 3.1 |  | 257 |  |
| DN992_c0_g1_i1.g6532.t1 | hypothetical protein FOC4_g10013861 | Zn (2)-C6 fungal-type DNA-binding domain-GAL4 | 3.0 |  | 986 | Transcription |
| DN996_c0_g2_i3.g6496.t1 | tyrosine 3-monooxygenase/tryptophan 5-monooxygenase activation protein | 14-3-3 domain | 1.5 |  | 275 |  |
| DN9987_c0_g1_i1.g18357.t1 | 6-hydroxy-D-nicotine oxidase | FAD linked oxidase, N-terminal | 5.4 | Extracellular | 219 | Oxidation-reduction |
