# Supplementary material for: Comparative transcriptomic analysis of races 1, 2, 5 and 6 of Fusarium oxysporum f.sp. pisi in a susceptible pea host identifies differential pathogenicity profiles
Source: BMC Genomics. 2021 Oct 9;22:734. doi: 10.1186/s12864-021-08033-y (PMC8502283; doi:10.1186/s12864-021-08033-y)
Supplement: Supplementary file 5 — Additional file 5: Table S3. Differentially expressed Fop genes detected in R5 at 20 dpi - column 1 with other analyses such as: predicted proteins - column 2, conserved domain - column 3, log2fold change - column 4, subcellular localisation of the effector-like proteins - column 5, protein length - column 6, and GO functional enrichment for biological processes (BP) - column 7. DEGs predicted to be effector-like are shaded yellow and they were all located on the adaptive genome. [file 12864_2021_8033_MOESM5_ESM.docx]

**Supplementary Table 3**

| NODE_1.g87.t1 | hypothetical protein FOTG_14254 |  | 5.3 |  | 337 |  |
| --- | --- | --- | --- | --- | --- | --- |
| NODE_10.g1401.t1 | hypothetical protein BFJ69_g6347 | Carboxylesterase, type B | 4.5 |  | 525 |  |
| NODE_10.g1403.t1 | hypothetical protein FOC4_g10009543 |  | 4.2 |  | 282 |  |
| NODE_10.g1421.t1 | Developmental regulator flbA | RGS domain, Regulator of G protein | 6.4 |  | 479 | Signal transduction |
| NODE_10.g1465.t1 | hypothetical protein FOC1_g10002677 |  | 5.5 |  | 364 |  |
| NODE_100.g7628.t1 | F-type H+-transporting ATPase subunit B | ATP synthase, F0 complex, subunit B/MI25 | 1.3 |  | 242 | Transport |
| NODE_100.g7629.t1 | hypothetical protein FOC1_g10015989 | CCAAT-binding factor | 1.6 |  | 549 |  |
| NODE_101.g7666.t1 | murein transglycosylase | Glycoside hydrolase family 17 | 1.4 |  | 403 | Metabolic |
| NODE_101.g7674.t1 | Protein pyrABCN | Carbamoyl-phosphate synthetase large subunit-like, ATP-binding domain | 6.0 |  | 2240 | Metabolic |
| NODE_101.g7689.t1 | hypothetical protein BFJ69_g4989 | Mitochondrial substrate/solute carrier | 2.2 |  | 336 | Transport |
| NODE_101.g7696.t1 | hypothetical protein FOXG_06328 |  | 7.8 |  | 258 |  |
| NODE_101.g7697.t1 | hypothetical protein FOTG_03461 |  | 4.2 |  | 260 |  |
| NODE_102.g7713.t1 | probable ribosomal protein L13B | Ribosomal protein L13e | 1.5 |  | 212 | Translation |
| NODE_102.g7716.t1 | Phospho-2-dehydro-3-deoxyheptonate aldolase, tyrosine-inhibited | DAHP synthetase I/KDSA | 2.0 |  | 364 | Biosynthetic |
| NODE_102.g7726.t1 | Choline transport protein | Amino acid/polyamine transporter I | 4.0 |  | 531 | Transport |
| NODE_102.g7730.t1 | serine hydroxymethyltransferase, mitochondrial | Serine hydroxymethyltransferase-like domain | 1.8 |  | 502 | Biosynthetic |
| NODE_102.g7732.t1 | glycine dehydrogenase | Glycine cleavage system P protein | 2.4 |  | 1052 | Oxidation-reduction |
| NODE_104.g15115.t1 | hypothetical protein FOXG_09988 | Glycoside hydrolase family 23 | 1.5 |  | 511 | Metabolic |
| NODE_104.g7812.t1 | 60S ribosomal protein L30 | Ribosomal protein L7Ae/L30e/S12e/Gadd45 | 1.4 |  | 110 | Translation |
| NODE_104.g7815.t1 | hypothetical protein FOTG_03160 |  | 1.6 |  | 938 |  |
| NODE_104.g7816.t1 | spermidine synthase | Spermidine synthase, tetramerisation domain | 2.4 |  | 294 | Metabolic |
| NODE_104.g7829.t1 | Ribosomal RNA-processing protein 7 | Ribosomal RNA-processing protein 7 | 1.6 |  | 313 | rRNA processing |
| NODE_104.g7834.t1 | eukaryotic translation initiation factor 3 subunit I | WD domain, G-beta repeat | 6.1 |  | 366 | Signal transduction |
| NODE_105.g7867.t1 | hypothetical protein FOXG_10949 |  | 7.7 | Extracellular | 109 |  |
| NODE_106.g15139.t1 | hypothetical protein BFJ69_g9930 | Major facilitator, sugar transporter-like | 2.2 |  | 557 | Transport |
| NODE_106.g7906.t1 | hypothetical protein FOTG_02910 | U3 small nucleolar ribonucleoprotein complex, subunit Mpp10 | 1.6 |  | 709 | rRNA processing |
| NODE_106.g7927.t1 | hypothetical protein FPSE_03899 | Ribosomal protein L7Ae/L30e/S12e/Gadd45 | 3.4 | Cytoplasm | 124 | Translation |
| NODE_108.g7999.t1 | hypothetical protein FPSE_00523 | Ribosomal protein L13 | 1.6 |  | 202 | Translation |
| NODE_108.g8006.t1 | hypothetical protein BFJ69_g3924 | DSBA-like thioredoxin domain | 1.5 |  | 222 |  |
| NODE_11.g1509.t1 | hypothetical protein FOXG_08912 | Amino acid permease/ SLC12A domain | 3.8 |  | 540 | Transport |
| NODE_11.g1534.t1 | pectate lyase E | pectate lyase | 7.1 |  | 594 | Metabolic |
| NODE_11.g1535.t1 | MFS transporter, SP family, general alpha glucoside:H+ symporter | Major facilitator, sugar transporter-like | 7.1 |  | 548 | Transport |
| NODE_110.g8072.t1 | probable TIM23 translocase | Tim17/Tim22/Tim23/Pmp24 family | 1.5 | Mitochondrion | 239 | Transport |
| NODE_110.g8080.t1 | hypothetical protein FOTG_04036 | WD domain, G-beta repeat | 2.7 |  | 490 | Signal transduction |
| NODE_110.g8091.t1 | hypothetical protein FOTG_04049 |  | 3.9 |  | 197 |  |
| NODE_110.g8110.t1 | translation initiation factor 3 subunit H | Eukaryotic translation initiation factor 3 subunit H | 1.1 |  | 364 | Translation |
| NODE_111.g8118.t1 | Protein SOK1 | T-complex 11 | 2.3 |  | 638 |  |
| NODE_111.g8129.t1 | probable NADH dehydrogenase (ubiquinone) 78K chain precursor | Molybdopterin oxidoreductase | 1.0 |  | 742 | Oxidation-reduction |
| NODE_111.g8134.t1 | hypothetical protein FOTG_07624 | SAM-dependent methyltransferase RsmB/NOP2-type | 2.7 |  | 679 | Methylation |
| NODE_112.g8159.t1 | hypothetical protein BFJ69_g10033 |  | 1.0 |  | 356 |  |
| NODE_112.g8162.t1 | pre-mRNA-splicing factor ATP-dependent RNA helicase prp43 | P-loop containing nucleoside triphosphate hydrolase | 4.2 |  | 767 | Phosphorylation |
| NODE_112.g8174.t1 | aspartate aminotransferase, mitochondrial | Aminotransferase, class I/classII | 2.7 |  | 424 | Biosynthetic |
| NODE_112.g8177.t1 | probable tryptophan synthase | Tryptophan synthase, alpha chain | 1.6 |  | 717 | Metabolic |
| NODE_112.g8183.t1 | probable adenylate kinase | Adenylate kinase/UMP-CMP kinase | 2.2 |  | 256 | Metabolic |
| NODE_115.g8315.t1 | Cellular nucleic acid-binding protein like protein | Zinc finger, CCHC-type | 2.6 | Cytoplasm | 182 | Transcription |
| NODE_116.g8324.t1 | Putative tRNA pseudouridine synthase 4 | Pseudouridine synthase II, N-terminal | 1.9 |  | 490 | RNA modification |
| NODE_116.g8353.t1 | Large subunit GTPase 1 | GTP binding domain | 1.8 |  | 672 | Signal transduction |
| NODE_116.g8356.t1 | malate dehydrogenase (oxaloacetate-decarboxylating) (NADP+) | Malic enzyme, NAD-binding | 1.2 |  | 613 |  |
| NODE_117.g8366.t1 | hypothetical protein FOTG_02410 | Ribonuclease H-like superfamily | 2.4 |  | 810 | RNA processing |
| NODE_119.g8451.t1 | hypothetical protein FOQG_12476 |  | 4.0 |  | 285 |  |
| NODE_119.g8464.t1 | enoyl-CoA hydratase | Enoyl-CoA hydratase/isomerase | 8.7 |  | 281 | Catabolic |
| NODE_121.g8518.t1 | hypothetical protein FOTG_05526 |  | 1.6 |  | 605 |  |
| NODE_121.g8529.t1 | citrate synthase, mitochondrial | Citrate synthase | 2.2 |  | 470 | Metabolic |
| NODE_121.g8531.t1 | plasma membrane ATPase | Cation-transporting P-type ATPase, N-terminal | 2.1 |  | 923 | Transport |
| NODE_122.g8541.t1 | hypothetical protein FOTG_08127 | Pectinesterase, catalytic | 8.7 |  | 2621 | metabolic |
| NODE_122.g8573.t1 | hypothetical protein FOTG_10371 | Protein kinase domain-Histidine kinase | 1.7 |  | 1228 | Signal transduction |
| NODE_123.g8580.t1 | methylenetetrahydrofolate dehydrogenase (NAD+) | Tetrahydrofolate dehydrogenase/cyclohydrolase, catalytic domain | 2.0 |  | 338 | Oxidation-reduction |
| NODE_123.g8584.t1 | S-adenosylmethionine synthase | S-adenosylmethionine synthetase, C-terminal | 1.8 |  | 403 | Biosynthetic |
| NODE_123.g8597.t1 | Putative protein phosphatase 2C 80 | PPM-type phosphatase domain superfamily | 1.4 |  | 325 |  |
| NODE_123.g8598.t1 | hypothetical protein FOTG_06087 | rRNA-processing protein Fcf1/Utp23 | 2.0 |  | 287 | RNA processing |
| NODE_123.g8610.t1 | putative WD repeat-containing protein C17D11.16 | WD domain, G-beta repeat | 3.4 |  | 559 | Signal transduction |
| NODE_124.g8657.t1 | probable succinyl-CoA 3-ketoacid-coenzyme A transferase, mitochondrial precursor | Coenzyme A transferase family I | 3.2 |  | 1087 | Catabolic |
| NODE_124.g8659.t1 | hypothetical protein FOQG_09102 | AhpD-like | 3.1 |  | 291 | Stress response |
| NODE_125.g8685.t1 | hypothetical protein FOTG_15691 | Zn (2)-C6 fungal-type DNA-binding domain superfamily | 2.8 |  | 434 | Transcription |
| NODE_126.g8713.t1 | ATP synthase subunit beta, mitochondrial | ATPase, F1/V1/A1 complex, alpha/beta subunit, nucleotide-binding domain | 1.3 |  | 514 | Transport |
| NODE_126.g8714.t1 | 1,4-alpha-glucan-branching enzyme | Glycoside hydrolase, family 13, N-terminal | 2.9 |  | 707 | Metabolic |
| NODE_127.g8738.t1 | hypothetical protein FOC4_g10014240 |  | 1.8 |  | 330 |  |
| NODE_127.g8753.t1 | hypothetical protein FOTG_04573 | Nucleolar complex protein 2 | 2.6 |  | 777 | ribosome biogenesis |
| NODE_127.g8757.t1 | threonine ammonia-lyase, biosynthetic | Threonine dehydratase, ACT-like domain | 1.1 |  | 584 | Biosynthetic |
| NODE_127.g8760.t1 | homocitrate synthase, mitochondrial | Pyruvate carboxyltransferase | 1.5 |  | 420 | Metabolic |
| NODE_127.g8767.t1 | Aquaporin-2 | Major intrinsic protein | 4.6 |  | 320 | Transport |
| NODE_127.g8770.t1 | homocitrate synthase | Pyruvate carboxyltransferase | 5.7 |  | 383 | Metabolic |
| NODE_128.g8779.t1 | aconitate hydratase, mitochondrial | Aconitase/3-isopropylmalate dehydratase large subunit, alpha/beta/alpha domain | 2.2 |  | 809 | Metabolic |
| NODE_128.g8805.t1 | hypothetical protein FOTG_03075 | tRNA/rRNA methyltransferase, SpoU type | 2.0 |  | 692 | RNA processing |
| NODE_129.g8818.t1 | Protein sof1 | WD domain, G-beta repeat | 1.5 |  | 445 | Signal transduction |
| NODE_129.g8828.t1 | hypothetical protein FOXG_11256 | Telomere repeat-binding factor, dimerisation domain | 1.9 |  | 919 |  |
| NODE_129.g8829.t1 | hypothetical protein FOTG_05467 |  | 3.5 |  | 221 |  |
| NODE_129.g8830.t1 | hypothetical protein FOC1_g10013109 |  | 3.9 |  | 421 |  |
| NODE_129.g8840.t1 | zinc finger protein | Zinc finger, ZPR1-type | 1.3 |  | 483 | Transcription |
| NODE_130.g8855.t1 | hypothetical protein FOTG_03996 | Sas10 C-terminal domain | 2.7 |  | 616 |  |
| NODE_130.g8858.t1 | DNA-directed RNA polymerase I subunit rpa49 | RNA polymerase I associated factor, A49-like | 3.0 |  | 439 | Transcription |
| NODE_130.g8873.t1 | Guanine nucleotide binding protein (G-protein), alpha subunit | Guanine nucleotide binding protein (G-protein), alpha subunit | 1.2 |  | 353 | Signal transduction |
| NODE_132.g8921.t1 | 50S ribosomal protein L14e | Ribosomal protein L14e | 1.3 |  | 147 | Translation |
| NODE_133.g8971.t1 | tRNA (Met) cytidine acetyltransferase | Acetyltransferase (GNAT) domain | 3.4 |  | 1066 | Biosynthetic |
| NODE_133.g8981.t1 | Adenine phosphoribosyltransferase | Phosphoribosyltransferase domain | 1.6 |  | 222 | Metabolic |
| NODE_135.g9012.t1 | hypothetical protein FOTG_00564 | Enoyl-CoA hydratase/isomerase, HIBYL-CoA-H type | 1.2 |  | 504 | Catabolic |
| NODE_135.g9030.t1 | hypothetical protein FOTG_00584 | Mitochondrial protein Pet127 | 1.5 |  | 1059 | metabolic |
| NODE_136.g9051.t1 | hypothetical protein BFJ69_g9258 | Ribosomal protein S11 superfamily | 1.5 |  | 237 | Translation |
| NODE_136.g9052.t1 | hypothetical protein FOTG_09867 | Tim44-like domain | 2.6 |  | 553 | Transport |
| NODE_136.g9060.t1 | ATP-dependent RNA helicase DBP9 | P-loop containing nucleoside triphosphate hydrolase | 1.6 |  | 614 | Phosphorylation |
| NODE_137.g9083.t1 | hypothetical protein FOXG_01669 | Mitochondrial substrate/solute carrier | 5.5 |  | 695 | Transport |
| NODE_137.g9093.t1 | Eukaryotic translation initiation factor 3 subunit M | Proteasome component (PCI) domain | 2.0 |  | 451 |  |
| NODE_138.g15453.t1 | hypothetical protein FOC1_g10013863 |  | 2.9 |  | 307 |  |
| NODE_139.g9160.t1 | hypothetical protein BFJ65_g13442 |  | 5.9 |  | 384 |  |
| NODE_139.g9161.t1 | Purine-cytosine permease FCY21 | Purine-cytosine permease | 4.8 |  | 509 | Transport |
| NODE_140.g9218.t1 | hypothetical protein FOCG_07260 |  | 7.9 |  | 368 |  |
| NODE_141.g9234.t1 | hypothetical protein FOC1_g10006917 | NmrA-like domain | 7.7 |  | 308 |  |
| NODE_141.g9244.t1 | probable iron inhibited ABC transporter 2 | ABC transporter-like | 3.6 |  | 1110 | Transport |
| NODE_144.g9337.t1 | mitochondrial inner membrane magnesium transporter mrs2 | Magnesium transporter MRS2-like | 1.6 |  | 485 | Transport |
| NODE_144.g9339.t1 | hypothetical protein FOTG_01491 | Cyclin PHO80-like | 1.1 |  | 691 | Signal transduction |
| NODE_145.g9366.t1 | Eukaryotic translation initiation factor 5B | GTP-binding domain | 1.4 |  | 985 | Signal transduction |
| NODE_145.g9367.t1 | heat shock 70kDa protein 4 | Heat shock protein 70 family | 2.7 |  | 784 | Stress response |
| NODE_145.g9371.t1 | Vacuolar cation/proton exchanger 2 | Sodium/calcium exchanger membrane region | 3.8 |  | 450 | Transport |
| NODE_145.g9375.t1 | hypothetical protein FOC1_g10006701 | PAS fold 3 | 1.8 |  | 658 |  |
| NODE_145.g9397.t1 | SWI/SNF and RSC complexes subunit ssr2 | Homeobox-like domain superfamily | 1.3 |  | 672 |  |
| NODE_145.g9398.t1 | carnitine O-acetyltransferase | Choline/carnitine acyltransferase domain | 2.3 |  | 610 | Proteolysis |
| NODE_146.g9411.t1 | hypothetical protein BFJ69_g877 | Aminoacyl-tRNA synthetase, class II (D/K/N) | 1.4 |  | 580 | tRNA aminoacylation |
| NODE_146.g9415.t1 | thioredoxin reductase (NADPH) | Thioredoxin reductase | 1.1 |  | 330 | Oxidation-reduction |
| NODE_146.g9423.t1 | hypothetical protein FAVG1_01712 | IMP dehydrogenase/GMP reductase | 2.0 |  | 532 | Oxidation-reduction |
| NODE_146.g9429.t1 | hypothetical protein FOTG_06316 | Protein kinase domain-Histidine kinase/HSP90-like ATPase | 2.4 |  | 1508 | Signal transduction |
| NODE_147.g15522.t1 | hypothetical protein FOIG_07053 |  | 1.6 | Nucleus | 149 |  |
| NODE_147.g9435.t1 | 60S ribosomal protein L4-B | Ribosomal protein L4/L1e | 1.5 |  | 1360 | Translation |
| NODE_147.g9437.t1 | probable translation elongation factor EF-Tu precursor, mitochondrial | GTP-binding domain | 2.0 |  | 445 | Signal transduction |
| NODE_147.g9450.t1 | uncharacterized protein FFUJ_02673 |  | 4.5 |  | 641 |  |
| NODE_148.g9475.t1 | hypothetical protein BFJ65_g10643 | PH-like domain superfamily | 3.1 |  | 490 |  |
| NODE_148.g9476.t1 | hypothetical protein FOTG_01616 | Arrestin-like, N-terminal | 1.2 |  | 599 |  |
| NODE_148.g9484.t1 | Ribosome biogenesis protein enp2 like protein | WD domain, G-beta repeat | 3.1 |  | 651 | Signal transduction |
| NODE_149.g9496.t1 | translation initiation factor 4G | MIF4G-like, type 3 | 3.8 |  | 1504 |  |
| NODE_149.g9498.t1 | hypothetical protein FOQG_03650 | Pentatricopeptide repeat | 2.0 |  | 704 |  |
| NODE_149.g9506.t1 | solute carrier family 25, member 46 | Mitochondrial substrate/solute carrier | 1.3 |  | 310 | Transport |
| NODE_150.g9526.t1 | hypothetical protein FOTG_09300 | Ketopantoate reductase, C-terminal domain | 1.3 |  | 465 | Oxidation-reduction |
| NODE_150.g9538.t1 | hypothetical protein FOCG_09020 | RNA recognition motif domain | 1.4 |  | 320 | Cytokinesis |
| NODE_151.g9561.t1 | hypothetical protein FOXG_11073 | R3H domain | 2.5 |  | 624 |  |
| NODE_151.g9572.t1 | hypothetical protein BFJ69_g5697 | Protein kinase domain | 1.9 |  | 1245 | Signal transduction |
| NODE_153.g9601.t1 | Nucleolar protein 56 | Nop domain | 3.1 |  | 511 | Ribosome biogenesis |
| NODE_153.g9611.t1 | homoaconitase, mitochondrial | Aconitase/3-isopropylmalate dehydratase large subunit, alpha/beta/alpha domain | 1.1 |  | 777 | Metabolic |
| NODE_154.g9633.t1 | hypothetical protein FOTG_02891 | Fork head domain | 2.3 |  | 1313 | Transcription |
| NODE_154.g9640.t1 | probable 60S ribosomal protein L5 | Ribosomal protein L5 eukaryotic/L18 archaeal | 1.5 |  | 302 | Translation |
| NODE_154.g9641.t1 | hypothetical protein FOIG_07115 | Leucine-rich repeat domain superfamily | 1.9 |  | 1186 |  |
| NODE_154.g9648.t1 | hypothetical protein FOC1_g10012848 | Zn (2)-C6 fungal-type DNA-binding domain | 4.0 |  | 1149 | Transcription |
| NODE_155.g9677.t1 | hypothetical protein FOTG_18404 | ATP-grasp fold, succinyl-CoA synthetase-type | 6.7 |  | 418 | Metabolic |
| NODE_156.g9695.t1 | hypothetical protein FOTG_00827 | WD domain, G-beta repeat | 2.0 |  | 908 | Signal transduction |
| NODE_156.g9697.t1 | Eukaryotic translation initiation factor 4E type 2 | Translation Initiation factor eIF- 4e | 1.5 |  | 341 | Translation |
| NODE_156.g9698.t1 | 60S ribosomal subunit assembly/export protein loc-1 |  | 1.6 |  | 195 |  |
| NODE_156.g9700.t1 | CTD kinase subunit alpha | Protein kinase domain | 1.3 |  | 994 | Signal transduction |
| NODE_157.g9706.t1 | eukaryotic translation initiation factor 3 subunit D | Eukaryotic translation initiation factor 3 subunit D | 1.9 |  | 576 | Translation |
| NODE_157.g9711.t1 | dynamin GTPase | Dynamin superfamily | 1.8 |  | 838 |  |
| NODE_157.g9720.t1 | hypothetical protein FOTG_05902 |  | 2.2 |  | 272 |  |
| NODE_157.g9722.t1 | hypothetical protein FOTG_05904 | CCAAT-binding factor | 2.7 |  | 987 |  |
| NODE_159.g15623.t1 | probable GAP1-General amino acid permease | Amino acid permease/ SLC12A domain | 1.4 |  | 496 | Transport |
| NODE_159.g9787.t1 | hypothetical protein FOTG_04522 | Major facilitator superfamily | 1.8 |  | 497 | Transport |
| NODE_160.g9788.t1 | hypothetical protein FOQG_03292 | Zinc finger C2H2-type | 1.9 |  | 692 | Transcription |
| NODE_160.g9789.t1 | hypothetical protein FOTG_04369 | Kelch-type beta propeller | 1.0 |  | 757 |  |
| NODE_160.g9796.t1 | Fe/S biogenesis protein NfuA | NIF system FeS cluster assembly, NifU, C-terminal | 1.5 |  | 305 | Iron-sulfur cluster assembly |
| NODE_160.g9798.t1 | hypothetical protein FOTG_04359 |  | 5.7 |  | 433 |  |
| NODE_160.g9804.t1 | probable YHM1 (mitochondrial carrier) | Mitochondrial substrate/solute carrier | 1.5 |  | 303 | Transport |
| NODE_160.g9805.t1 | DNA-directed RNA polymerase III subunit rpc-3 | RNA polymerase III Rpc82, C -terminal | 2.1 |  | 633 | Transcription |
| NODE_161.g9821.t1 | succinate-semialdehyde dehydrogenase (NADP+) | Aldehyde dehydrogenase domain | 2.7 |  | 494 | Oxidation-reduction |
| NODE_161.g9824.t1 | hypothetical protein FOTG_06625 | Zn (2)-C6 fungal-type DNA-binding domain | 7.3 |  | 784 | Transcription |
| NODE_161.g9825.t1 | hypothetical protein FOTG_06621 | WD domain, G-beta repeat | 4.5 |  | 433 | Signal transduction |
| NODE_161.g9832.t1 | alpha-L-arabinofuranosidase | Glycoside hydrolase, family 43 | 1.6 |  | 1457 | Metabolic |
| NODE_161.g9834.t1 | General amino acid permease AGP2 | Amino acid permease/ SLC12A domain | 3.2 |  | 565 | Transport |
| NODE_161.g9835.t1 | probable translation elongation factor eEF-3 | ABC transporter-like | 4.7 |  | 1055 | Transport |
| NODE_163.g9881.t1 | probable heat shock protein 70 | Heat shock protein 70 family | 5.6 |  | 614 | Stress response |
| NODE_163.g9887.t1 | Laccase | Multicopper oxidase, type 2 | 4.3 |  | 91 | Oxidation-reduction |
| NODE_164.g15661.t1 | tRNA modification GTPase mnmE | GTP-binding protein TrmE, N-terminal | 1.7 |  | 569 | Signal transduction |
| NODE_164.g9927.t1 | hypothetical protein BFJ69_g14497 | Glycoside hydrolase family 18, catalytic domain | 6.4 |  | 432 | Metabolic |
| NODE_167.g9991.t1 | succinate dehydrogenase | Fumarate reductase/succinate dehydrogenase flavoprotein-like, C-terminal | 1.1 |  | 649 | Oxidation-reduction |
| NODE_168.g10007.t1 | aspartate kinase | Aspartate/glutamate/uridylate kinase | 1.5 |  | 511 | Biosynthetic |
| NODE_168.g10013.t1 | hypothetical protein BFJ69_g466 | Small acidic protein-like domain | 1.4 |  | 345 |  |
| NODE_168.g10027.t1 | 40S ribosomal protein S9, mitochondrial | Ribosomal protein S9 | 1.3 |  | 314 | Translation |
| NODE_168.g10028.t1 | Elongation factor 1-gamma 1 | Elongation factor 1B gamma, C-terminal | 1.1 |  | 427 | Translation |
| NODE_169.g10058.t1 | Putative metal ion transporter C27B12.12c | CorA-like Mg2+ transporter protein | 1.7 |  | 623 | Transport |
| NODE_169.g15717.t1 | hypothetical protein BFJ67_g12378 |  | 3.2 |  | 532 |  |
| NODE_170.g10064.t1 | Cytoplasmic 60S subunit biogenesis factor REI1 | ZN622/Rei1/Reh1, zinc finger C2H2-type | 1.7 |  | 548 | Transcription |
| NODE_170.g10069.t1 | hypothetical protein BFJ65_g9062 | Srp40, C-terminal | 1.8 |  | 417 |  |
| NODE_170.g10077.t1 | probable TIM17-mitochondrial inner membrane import translocase subunit | Tim17/Tim22/Tim23/Pmp24 family | 1.8 |  | 152 | Transport |
| NODE_174.g10205.t1 | U3 small nucleolar RNA-associated protein 4 | WD domain, G-beta repeat | 2.3 |  | 879 | Signal transduction |
| NODE_177.g10257.t1 | probable isocitrate dehydrogenase (NAD) | Isopropylmalate dehydrogenase-like domain | 1.2 |  | 381 | Oxidation-reduction |
| NODE_178.g10278.t1 | hypothetical protein FOTG_00133 | Ribosomal RNA methyltransferase FtsJ domain | 3.2 |  | 794 | Methylation |
| NODE_178.g10284.t1 | ATPase | P-type ATPase, subfamily V | 1.4 |  | 1337 | Transport |
| NODE_178.g10285.t1 | putative nucleosome assembly protein | Nucleosome assembly protein (NAP) | 2.5 |  | 404 | Nucleosome assembly |
| NODE_178.g10297.t1 | hypothetical protein FPSE_09444 | Ribosomal protein S3Ae | 1.1 |  | 256 | Translation |
| NODE_179.g10312.t1 | Ribosome production factor 1 | Brix domain | 1.5 |  | 415 | Ribosomal large subunit assembly |
| NODE_179.g10313.t1 | hypothetical protein FOTG_03887 | CCAAT-binding factor | 1.6 |  | 665 |  |
| NODE_179.g10323.t1 | F-box and WD-40 domain-containing protein CDC4 | WD domain, G-beta repeat | 1.4 |  | 621 | Signal transduction |
| NODE_181.g10343.t1 | hypothetical protein FOPG_06469 | Eukaryotic rRNA processing | 2.4 |  | 415 | rRNA processing |
| NODE_181.g10353.t1 | histone-lysine N-methyltransferase, H3 lysine-4 specific | Histone-lysine N-methyltransferase, H3 lysine-4 specific | 1.8 |  | 1258 | Histone lysine methylation |
| NODE_184.g10441.t1 | hypothetical protein FOC1_g10002574 |  | 2.1 |  | 386 |  |
| NODE_185.g10471.t1 | related to high mobility group-like protein NHP2 | Ribosomal protein L7Ae/L30e/S12e/Gadd45 | 1.2 |  | 352 | Translation |
| NODE_185.g10484.t1 | preli msf1 | PRELI/MSF1 domain | 1.9 |  | 184 |  |
| NODE_186.g10496.t1 | hypothetical protein FOTG_04178 | PAS domain | 2.1 |  | 663 |  |
| NODE_189.g10571.t1 | hypothetical protein FOC4_g10007865 |  | 1.9 |  | 574 |  |
| NODE_19.g2375.t1 | hypothetical protein FOTG_18944 | Peptidase M12A | 5.3 |  | 521 | Proteolysis |
| NODE_19.g2376.t1 | hypothetical protein FOTG_07720 |  | 4.2 | Extracellular | 90 |  |
| NODE_192.g10648.t1 | hypothetical protein FOC1_g10005850 | Methyltransferase domain 25 | 1.5 |  | 622 | Methylation |
| NODE_193.g10682.t1 | hypothetical protein FOQG_01907 |  | 1.3 |  | 421 |  |
| NODE_194.g10701.t1 | hypothetical protein FOXG_12803 | Heat shock factor (HSF)-type, DNA-binding | 4.0 |  | 586 | Transcription |
| NODE_194.g10709.t1 | tRNA (uracil(54)-C(5))-methyltransferase | (Uracil-5)-methyltransferase family | 1.6 |  | 542 | RNA processing |
| NODE_195.g10736.t1 | hypothetical protein FOPG_08978 |  | 3.5 |  | 921 |  |
| NODE_195.g10746.t1 | hypothetical protein FOTG_09068 | RNA recognition motif domain | 4.4 |  | 551 | Cytokinesis |
| NODE_196.g10752.t1 | ATP-dependent RNA helicase DRS1 | P-loop containing nucleoside triphosphate hydrolase | 2.5 |  | 797 | Phosphorylation |
| NODE_196.g10754.t1 | 2-isopropylmalate synthase | 2-isopropylmalate synthase LeuA, allosteric (dimerisation) domain | 1.9 |  | 616 | Biosynthetic |
| NODE_196.g10760.t1 | ribonuclease Z | Metallo-beta-lactamase | 3.1 |  | 836 |  |
| NODE_197.g10775.t1 | Peroxisomal adenine nucleotide transporter 1 | Mitochondrial substrate/solute carrier | 1.7 |  | 340 | Transport |
| NODE_197.g10785.t1 | protein TIF31 | Tetratricopeptide repeat | 3.2 |  | 1261 | RNA processing |
| NODE_197.g10798.t1 | hypothetical protein FOIG_07922 | guanine-nucleotide exchange factors catalytic domain (Ras) | 1.7 |  | 1209 | Signal transduction |
| NODE_199.g10823.t1 | hypothetical protein FOQG_00538 | Armadillo-type fold | 1.4 |  | 1210 |  |
| NODE_199.g10826.t1 | 40S ribosomal protein S13 |  | 1.0 |  | 151 |  |
| NODE_199.g10830.t1 | probable ring-finger protein Ariadne-1 | Zinc finger, RING/FYVE/PHD-type | 1.7 |  | 545 | Transcription |
| NODE_199.g10836.t1 | hypothetical protein FOTG_00538 | Armadillo-type fold | 1.9 |  | 759 |  |
| NODE_20.g2502.t1 | hypothetical protein FAVG1_02914 | Ribosomal protein L15 | 1.3 |  | 149 | Translation |
| NODE_20.g2538.t1 | hypothetical protein FOIG_06903 |  | 8.7 |  | 362 |  |
| NODE_201.g10891.t1 | hypothetical protein BFJ71_g13037 | Amine oxidase | 6.6 |  | 625 | Oxidation-reduction |
| NODE_204.g10955.t1 | Nucleolar GTP-binding protein 2 | Nucleolar GTP-binding protein 2 | 1.1 |  | 620 | Signal Transduction |
| NODE_204.g10960.t1 | elongation factor G, mitochondrial | GTP-binding domain | 3.2 |  | 825 | Signal transduction |
| NODE_204.g10964.t1 | kynurenine 3-monooxygenase | FAD-binding domain | 1.6 |  | 503 |  |
| NODE_205.g10985.t1 | Na (+)/H (+) antiporter | Cation/H+ exchanger | 1.2 |  | 1205 | Transport |
| NODE_206.g11003.t1 | MAP kinase kinase kinase wis4 | Protein kinase domain-Mitogen-activated protein (MAP) kinase kinase kinase Ssk2/Ssk22 | 1.8 |  | 1314 | Signal transduction |
| NODE_206.g11013.t1 | nucleolar protein 12 | RNA recognition motif 1 | 3.2 |  | 560 | Cytokinesis |
| NODE_207.g11029.t1 | phosphoenolpyruvate carboxykinase | Phosphoenolpyruvate carboxykinase, ATP-utilising | 3.9 |  | 589 | Metabolic |
| NODE_207.g11030.t1 | Eukaryotic peptide chain release factor GTP-binding subunit | Translation elongation factor EFTu/EF1A, C-terminal | 1.3 |  | 704 | Translation |
| NODE_209.g11092.t1 | Ribosome biogenesis protein ERB1 | WD domain, G-beta repeat | 3.6 |  | 763 | Signal transduction |
| NODE_21.g2558.t1 | succinate-semialdehyde dehydrogenase | Aldehyde dehydrogenase, C-terminal | 1.3 |  | 490 | Oxidation-reduction |
| NODE_21.g2559.t1 | 4-aminobutyrate aminotransferase | Aminotransferase class-III | 1.7 |  | 459 | Biosynthetic |
| NODE_21.g2597.t1 | 40S ribosomal protein S12 | Ribosomal protein S12e | 1.1 |  | 146 | Translation |
| NODE_21.g2598.t1 | 40S ribosomal protein S22 | Ribosomal protein S8 | 1.3 |  | 130 | Translation |
| NODE_21.g2629.t1 | hypothetical protein BFJ66_g8830 | Pyridine nucleotide-disulphide oxidoreductase | 3.5 |  | 456 | Oxidation-reduction |
| NODE_21.g2630.t1 | hypothetical protein FOTG_00952 | HEAT repeat | 4.0 |  | 1807 |  |
| NODE_21.g2634.t1 | hypothetical protein FOTG_00956 | ATPase, AAA type | 3.8 |  | 4923 | Transport |
| NODE_21.g2637.t1 | putative pyrroline-5-carboxylate reductase | Pyrroline-5-carboxylate reductase | 3.7 |  | 343 | Oxidation-reduction |
| NODE_210.g11102.t1 | Malate synthase, glyoxysomal | Malate synthase | 4.1 |  | 542 | metabolic |
| NODE_213.g11172.t1 | probable ribosomal protein L7a.e.B, cytosolic | Ribosomal protein L7Ae/L30e/S12e/Gadd45 | 1.6 |  | 261 | Translation |
| NODE_215.g11204.t1 | hypothetical protein FOTG_01094 | Pre-rRNA-processing protein RIX1, N-terminal | 1.7 |  | 759 | Ribosome biogenesis |
| NODE_215.g11207.t1 | MEAB protein | bZIP | 1.8 |  | 410 | Transcription |
| NODE_218.g11285.t1 | alpha-N-arabinofuranosidase | Glycoside hydrolase, family 54 | 2.2 |  | 726 | Metabolic |
| NODE_219.g11318.t1 | PiT family inorganic phosphate transporter | Phosphate transporter | 6.6 |  | 607 | Transport |
| NODE_22.g2642.t1 | hypothetical protein FOTG_02190 | Clr5 domain | 2.2 |  | 887 |  |
| NODE_22.g2644.t1 | hypothetical protein BFJ71_g9758 | S-adenosyl-L-methionine-dependent methyltransferase | 3.1 |  | 1458 | Methylation |
| NODE_22.g2670.t1 | hypothetical protein BFJ69_g8254 | Nitroreductase | 3.3 | Mitochondrion | 205 | Stress response |
| NODE_22.g2674.t1 | probable fibrillarin (NOP1) | Fibrillarin | 3.0 |  | 319 | rRNA processing |
| NODE_22.g2675.t1 | 60S ribosomal protein L17 | Ribosomal protein L22/L17 | 1.4 |  | 185 | Translation |
| NODE_22.g2676.t1 | hypothetical protein FOTG_10041 | F-box domain | 1.5 |  | 853 |  |
| NODE_22.g2703.t1 | hypothetical protein BFJ71_g9776 |  | 1.2 |  | 475 |  |
| NODE_220.g11327.t1 | probable IMP4 protein | Brix domain | 2.6 |  | 298 | Ribosomal large subunit assembly |
| NODE_220.g11328.t1 | probable HUPF1 protein | RNA helicase UPF1, UPF2-interacting domain | 1.6 |  | 1083 | Catabolic |
| NODE_220.g11335.t1 | aarF domain-containing kinase | UbiB domain | 1.8 |  | 440 |  |
| NODE_221.g11341.t1 | E3 ubiquitin-protein ligase BRE1 | BRE1 E3 ubiquitin ligase | 1.5 |  | 645 | Chromatin organization |
| NODE_221.g11347.t1 | hypothetical protein FOC1_g10013697 | U3 snoRNA associated | 1.6 |  | 275 | rRNA processing |
| NODE_221.g11348.t1 | hypothetical protein FOC1_g10013696 | von Willebrand factor, type A | 3.0 |  | 1134 |  |
| NODE_221.g11353.t1 | hypothetical protein FPSE_09105 | Ribosomal protein L37ae | 1.3 |  | 92 | Translation |
| NODE_222.g11363.t1 | hypothetical protein BFJ68_g14319 | Major facilitator superfamily | 2.3 |  | 622 | Transport |
| NODE_222.g11364.t1 | Putative mitochondrial carnitine O-acetyltransferase | Choline/carnitine acyltransferase domain | 4.7 |  | 859 | Proteolysis |
| NODE_222.g11366.t1 | DNA-directed RNA polymerase III subunit RPC2 | RNA polymerase Rpb2, domain 3 | 1.5 |  | 1157 | Transcription |
| NODE_222.g11368.t1 | hypothetical protein FOIG_01343 | Ribosomal protein L15e | 1.4 |  | 474 | Translation |
| NODE_225.g11440.t1 | hypothetical protein FOC1_g10003552 | CoA-transferase family III | 4.0 |  | 576 | Catabolic |
| NODE_225.g11441.t1 | 3-hydroxybutyryl-CoA dehydrogenase | 3-hydroxyacyl-CoA dehydrogenase | 6.4 |  | 221 | Oxidation-reduction |
| NODE_226.g11460.t1 | Calcium-transporting ATPase 2 | Cation-transporting P-type ATPase, C-terminal | 4.7 |  | 1284 | Transport |
| NODE_227.g11464.t1 | hypothetical protein FOPG_07197 | Ribosome biogenesis protein BMS1/TSR1, C-terminal | 1.7 |  | 833 | Ribosome biogenesis |
| NODE_227.g11484.t1 | hypothetical protein BFJ69_g5286 | Bromodomain | 1.9 |  | 909 | Metabolic |
| NODE_228.g11496.t1 | U3 small nucleolar RNA-associated protein 17 | WD domain, G-beta repeat | 3.1 |  | 962 | Signal transduction |
| NODE_228.g11505.t1 | hypothetical protein FOXG_00851 | RNA recognition motif domain | 3.1 |  | 855 | Cytokinesis |
| NODE_229.g11523.t1 | hypothetical protein FOXG_11627 | Brix domain | 2.2 |  | 454 | Ribosomal large subunit assembly |
| NODE_229.g11524.t1 | ATP-dependent rRNA helicase RRP3 | P-loop containing nucleoside triphosphate hydrolase | 1.8 |  | 485 | Phosphorylation |
| NODE_23.g2718.t1 | chaperone hchA | glutamine amidotransferase-like | 7.4 |  | 295 | Metabolic |
| NODE_23.g2719.t1 | hypothetical protein FOTG_08258 | Alcohol dehydrogenase, zinc-type, conserved site | 4.4 |  | 365 | Oxidation-reduction |
| NODE_23.g2782.t1 | phosphoribosylaminoimidazole carboxylase | Phosphoribosylaminoimidazole carboxylase, fungi/plant | 2.2 |  | 594 | Biosynthetic |
| NODE_231.g11549.t1 | hypothetical protein BFJ69_g14551 |  | 6.9 |  | 646 |  |
| NODE_232.g11576.t1 | hypothetical protein FOQG_05794 | Tetratricopeptide repeat | 1.2 |  | 1239 | RNA processing |
| NODE_233.g11578.t1 | chromatin modification-like protein VID21 | Myb-like DNA-binding domain | 1.5 |  | 1612 |  |
| NODE_233.g11589.t1 | hypothetical protein FOXG_09391 | KRR1 interacting protein 1 | 1.3 |  | 657 |  |
| NODE_234.g11604.t1 | hypothetical protein FOTG_08614 | Armadillo-type fold | 3.9 |  | 2644 |  |
| NODE_234.g11608.t1 | hypothetical protein FOTG_08608 | DNA polymerase V/Myb-binding protein 1A | 2.0 |  | 1011 | Transcription |
| NODE_235.g11626.t1 | hypothetical protein FAVG1_05598 | Ribosomal protein L1 | 1.1 | Cytoplasm | 217 | Translation |
| NODE_235.g11634.t1 | 60S ribosomal protein L2 | Ribosomal protein L2 | 1.1 |  | 214 | Translation |
| NODE_237.g11648.t1 | Pumilio domain-containing protein C56F2.08c | RNA recognition motif domain | 1.1 |  | 1163 | Cytokinesis |
| NODE_237.g11658.t1 | NADH-cytochrome b5 reductase 1, partial | Flavoprotein pyridine nucleotide cytochrome reductase-like | 1.2 |  | 314 | Oxidation-reduction |
| NODE_238.g11661.t1 | probable beta karyopherin | Importin repeat 4 | 2.6 |  | 1096 | Transport |
| NODE_238.g11673.t1 | Aconitate hydratase | Aconitase/3-isopropylmalate dehydratase large subunit, alpha/beta/alpha domain | 2.7 |  | 785 | Metabolic |
| NODE_24.g2831.t1 | hypothetical protein BFJ72_g5189 | Major facilitator, sugar transporter-like | 4.8 |  | 516 | Transport |
| NODE_24.g2893.t1 | allantoicase | Allantoicase repeat | 1.8 |  | 360 | Catabolic |
| NODE_24.g2898.t1 | hypothetical protein FOC4_g10012609 | Amine oxidase | 1.7 |  | 519 | Oxidation-reduction |
| NODE_24.g2900.t1 | hypothetical protein FGSG_05999 | Ribosomal protein L27e | 1.1 |  | 135 | Translation |
| NODE_242.g11738.t1 | hypothetical protein BFJ68_g329 |  | 3.3 |  | 667 |  |
| NODE_243.g11745.t1 | hypothetical protein FVEG_11508 |  | 4.7 |  | 154 |  |
| NODE_246.g11809.t1 | hypothetical protein FOC1_g10002515 | Chaperone J-domain superfamily | 2.9 |  | 444 | Stress response |
| NODE_247.g11828.t1 | hypothetical protein BFJ72_g11734 | GDP/GTP exchange factor Sec2, N-terminal | 1.2 |  | 673 |  |
| NODE_249.g11851.t1 | hypothetical protein BFJ69_g6466 |  | 3.5 |  | 389 |  |
| NODE_25.g2955.t1 | hypothetical protein BFJ65_g12668 |  | 3.8 |  | 285 |  |
| NODE_250.g11872.t1 | hypothetical protein FOTG_03416 | Arrestin, C-terminal | 1.6 |  | 488 |  |
| NODE_250.g11888.t1 | hypothetical protein FOC1_g10011090 | Zn (2)-C6 fungal-type DNA-binding domain-GAL4 | 2.5 |  | 682 | Transcription |
| NODE_251.g11895.t1 | ribosomal protein L28e | Ribosomal L28e/Mak16 | 1.5 | Nucleus | 159 | Translation |
| NODE_251.g11898.t1 | hypothetical protein FOXG_10535 | Phosphoribosylglycinamide synthetase, ATP-grasp (A) domain | 3.0 |  | 797 | Biosynthetic |
| NODE_251.g11900.t1 | hypothetical protein FAVG1_00589 | Uncharacterised protein family UPF0642 | 2.0 |  | 119 |  |
| NODE_251.g11903.t1 | putative RNA-binding protein C4F6.14 | RNA recognition motif domain | 2.5 |  | 736 | Cytokinesis |
| NODE_254.g11956.t1 | hypothetical protein FOTG_09153 | Pentatricopeptide repeat | 2.2 |  | 851 |  |
| NODE_254.g11958.t1 | Imitation switch two complex protein 1 | WSTF/Acf1/Cbp146 | 1.4 |  | 1005 | Chromatin assembly and remodelling |
| NODE_256.g11993.t1 | mitochondrial import receptor subunit tom-40 | Eukaryotic porin/Tom40 | 1.7 |  | 356 | Transport |
| NODE_257.g11995.t1 | MFS transporter, FHS family, L-fucose permease | Major facilitator superfamily | 6.9 |  | 461 | Transport |
| NODE_257.g11996.t1 | hypothetical protein FOTG_16893 | BTB/POZ domain | 6.0 |  | 243 |  |
| NODE_26.g3041.t1 | hypothetical protein BFJ69_g13977 | Major facilitator superfamily | 2.5 |  | 505 | Transport |
| NODE_26.g3063.t1 | hypothetical protein FOIG_06037 | Tetratricopeptide repeat | 4.5 |  | 719 | RNA processing |
| NODE_26.g3065.t1 | hypothetical protein FOCG_02355 | Zinc finger C2H2-type | 1.3 |  | 276 | Transcription |
| NODE_260.g12031.t1 | hypothetical protein FOTG_15997 |  | 3.2 |  | 360 |  |
| NODE_261.g12055.t1 | Periodic tryptophan protein 2 | WD domain, G-beta repeat | 2.2 |  | 894 | Signal transduction |
| NODE_262.g12065.t1 | hypothetical protein FPSE_00057 | Ribosomal protein S19/S15 | 1.0 |  | 152 | Translation |
| NODE_263.g12085.t1 | hypothetical protein FOC4_g10012645 | Queuosine salvage protein family | 2.3 |  | 365 |  |
| NODE_263.g12097.t1 | hypothetical protein FPSE_02953 | Ribosomal protein S7 | 1.1 | Cytoplasm | 213 | Translation |
| NODE_266.g12143.t1 | Adenylosuccinate lyase | Adenylosuccinate lyase C-terminal | 3.3 |  | 485 | Biosynthetic |
| NODE_268.g12184.t1 | hypothetical protein FOTG_02856 | U3 small nucleolar RNA-associated protein 15 | 2.8 |  | 528 | rRNA processing |
| NODE_268.g12193.t1 | SWR1-complex protein 4 | DAMP1, SANT/Myb-like domain | 2.0 |  | 626 |  |
| NODE_269.g12203.t1 | Sugar transporter STL1 | Major facilitator, sugar transporter-like | 3.8 |  | 540 | Transport |
| NODE_269.g12211.t1 | hypothetical protein FOMG_04343 | Lysine methyltransferase | 4.1 |  | 575 | Methylation |
| NODE_27.g3105.t1 | hypothetical protein BFJ69_g4704 | NmrA-like domain | 5.4 |  | 299 |  |
| NODE_27.g3136.t1 | related to quinate transport protein | Major facilitator, sugar transporter-like | 3.8 |  | 559 | Transport |
| NODE_27.g3140.t1 | hypothetical protein FOTG_12357 |  | 5.1 |  | 961 |  |
| NODE_27.g3149.t1 | hypothetical protein FOTG_12342 |  | 2.9 |  | 614 |  |
| NODE_27.g3156.t1 | hypothetical protein FOXG_04914 | Protein of unknown function DUF829, TMEM53 | 2.3 |  | 285 |  |
| NODE_27.g3163.t1 | hypothetical protein FOQG_08021 | Domain of unknown function DUF1747 | 1.8 |  | 518 |  |
| NODE_27.g3166.t1 | hypothetical protein BFJ69_g14025 | Dual specificity protein phosphatase domain | 2.9 |  | 1059 | Protein dephosphorylation |
| NODE_271.g12225.t1 | delta-aminolevulinic acid dehydratase | Delta-aminolevulinic acid dehydratase | 2.6 |  | 386 | Biosynthetic |
| NODE_271.g12230.t1 | hypothetical protein FOXG_05440 | U3 small nucleolar RNA-associated SSU processome protein 25 (Utp25) | 2.0 |  | 719 | rRNA processing |
| NODE_271.g12231.t1 | related to translation initiation factor 3 (47 kDa subunit) | Eukaryotic translation initiation factor 3 subunit F | 1.4 |  | 358 | Translation |
| NODE_271.g12235.t1 | hypothetical protein FOC1_g10012878 | Tetratricopeptide repeat | 2.3 |  | 806 | RNA processing |
| NODE_28.g3176.t1 | mitochondrial presequence protease | Peptidase M16C associated | 1.6 |  | 1004 | Proteolysis |
| NODE_28.g3177.t1 | ornithine decarboxylase | Orn/DAP/Arg decarboxylase 2 | 1.2 |  | 448 |  |
| NODE_28.g3178.t1 | DNA repair protein RAD16 | Zinc finger, C3HC4 RING-type | 1.9 |  | 1073 | Transcription |
| NODE_28.g3233.t1 | preprotein translocase subunit YidC | Membrane insertase YidC/ALB3/OXA1/COX18 | 1.8 |  | 484 |  |
| NODE_28.g3238.t1 | ATP-dependent RNA helicase FAL1 | P-loop containing nucleoside triphosphate hydrolase | 1.3 |  | 402 | Phosphorylation |
| NODE_280.g12348.t1 | hypothetical protein FOTG_11444 | Glucose-methanol-choline oxidoreductase, C-terminal | 3.8 |  | 546 | Oxidation-reduction |
| NODE_281.g12369.t1 | hypothetical protein FOTG_09173 | Cytidine and deoxycytidylate deaminase domain | 2.7 |  | 513 |  |
| NODE_282.g12398.t1 | hypothetical protein FOTG_06378 | NACHT nucleoside triphosphatase | 4.8 |  | 1434 | Metabolic |
| NODE_283.g12402.t1 | hypothetical protein FOTG_13860 |  | 4.5 |  | 260 |  |
| NODE_285.g12429.t1 | hypothetical protein FOIG_02155 | Heat shock protein 70 family | 2.6 |  | 563 | Stress response |
| NODE_286.g12444.t1 | hypothetical protein FOXG_01504 | Pyridine nucleotide-disulphide oxidoreductase | 3.7 |  | 390 | Oxidation-reduction |
| NODE_29.g3267.t1 | hypothetical protein FOXG_08113 | Heat shock factor (HSF)-type, DNA-binding | 1.0 |  | 586 | Transcription |
| NODE_29.g3269.t1 | hypothetical protein FOQG_02770 | transcription factor-CP2 | 1.9 |  | 788 | Transcription |
| NODE_29.g3273.t1 | Eukaryotic translation initiation factor 2A | Translation initiation factor 2A | 2.1 |  | 674 | Translation |
| NODE_29.g3287.t1 | hypothetical protein FOTG_07507 | Nrap protein, domain 5 | 3.0 |  | 1105 |  |
| NODE_29.g3313.t1 | hypothetical protein BFJ69_g5147 | Programmed cell death protein 2, C-terminal | 1.6 |  | 401 |  |
| NODE_29.g3326.t1 | hypothetical protein BFJ69_g5136 | Mitochondrial carrier protein | 1.0 |  | 300 | Transport |
| NODE_296.g12581.t1 | Eukaryotic translation initiation factor 3 subunit L | Translation initiation factor 3 complex subunit L | 1.6 |  | 476 | Translation |
| NODE_296.g12589.t1 | Mitochondrial-processing peptidase subunit alpha | Peptidase M16, C-terminal | 1.7 |  | 524 | Proteolysis |
| NODE_296.g12591.t1 | hypothetical protein FOTG_09083 | S1 domain | 4.1 |  | 1783 |  |
| NODE_30.g3330.t1 | ATP-dependent RNA helicase DBP4 | P-loop containing nucleoside triphosphate hydrolase | 2.2 |  | 795 | Phosphorylation |
| NODE_30.g3331.t1 | Pre-rRNA-processing protein esf1 | NUC153 | 2.0 |  | 691 | rRNA processing |
| NODE_30.g3350.t1 | hypothetical protein FOTG_04141 | Nucleoporin, Nup133/Nup155-like, N-terminal | 1.3 |  | 1403 | Cell organisation |
| NODE_30.g3369.t1 | Putative protein RSN1 | Calcium-dependent channel, 7TM region, putative phosphate | 1.8 |  | 892 | Transport |
| NODE_30.g3378.t1 | Spartin | Senescence-associated protein | 1.0 |  | 504 |  |
| NODE_30.g3384.t1 | hypothetical protein FOTG_04099 | Ribosomal protein MRP51, fungi | 1.7 |  | 477 | Translation |
| NODE_30.g3393.t1 | hypothetical protein FOTG_04086 | Cyclin PHO80-like | 1.3 |  | 482 | Signal transduction |
| NODE_30.g3396.t1 | DNA-directed RNA polymerase I subunit RPA2 | DNA-directed RNA polymerase, subunit 2, hybrid-binding domain | 3.2 |  | 1232 | Transcription |
| NODE_300.g12648.t1 | hypothetical protein FPOA_00733 | Protein kinase domain | 4.0 |  | 1272 | Signal transduction |
| NODE_302.g12672.t1 | hypothetical protein FOXG_08441 |  | 1.5 |  | 285 |  |
| NODE_302.g12674.t1 | uncharacterized protein FFUJ_04877 | Mitochondrial protein | 1.6 | Mitochondrion | 282 | Metabolic |
| NODE_303.g12691.t1 | Altered inheritance rate of mitochondria protein 38 like protein | Hypoxia induced protein, domain | 3.1 |  | 228 |  |
| NODE_304.g12706.t1 | uncharacterized protein FMAN_11213 | CFEM domain | 1.0 |  | 195 |  |
| NODE_306.g12735.t1 | hypothetical protein BFJ69_g14983 |  | 1.4 |  | 991 |  |
| NODE_306.g12739.t1 | endo-xylogalacturonan hydrolase | Glycoside hydrolase, family 28 | 2.0 |  | 484 | Metabolic |
| NODE_306.g12741.t1 | 60S ribosomal protein L3 | Ribosomal protein L3 | 2.1 |  | 391 | Translation |
| NODE_307.g12745.t1 | hypothetical protein FOTG_16235 | Major facilitator, sugar transporter-like | 4.4 |  | 515 | Transport |
| NODE_31.g3421.t1 | hypothetical protein FOC1_g10005942 | Protein of unknown function DUF2434 | 1.4 |  | 581 |  |
| NODE_31.g3447.t1 | Putative CDP-alcohol phosphatidyltransferase class-I family protein C22A12.08c | Haloacid dehalogenase hydrolase like (HAD superfamily) | 1.4 |  | 494 |  |
| NODE_31.g3448.t1 | Aldose reductase A | Aldo/keto reductase | 1.2 |  | 329 | Oxidation-reduction |
| NODE_31.g3462.t1 | pectate lyase E | pectate lyase | 2.2 |  | 580 | Metabolic |
| NODE_31.g3469.t1 | omega-6 fatty acid desaturase (delta-12 desaturase) | Fatty acid desaturase domain | 5.0 |  | 402 | Metabolic |
| NODE_311.g12797.t1 | L-2-aminoadipate reductase large subunit | AMP-dependent synthetase/ligase | 1.6 |  | 1180 |  |
| NODE_311.g12800.t1 | hypothetical protein FOXG_11113 | Mitochondrial substrate/solute carrier | 4.0 |  | 321 | Transport |
| NODE_311.g12801.t1 | Protein sda1 | SDA1 domain | 2.4 |  | 740 |  |
| NODE_311.g12807.t1 | tRNA (adenine-N (1)-)-methyltransferase non-catalytic subunit trm6 | tRNA (adenine (58)-N (1))-methyltransferase non-catalytic subunit TRM6 | 2.7 |  | 360 | Methylation |
| NODE_312.g12819.t1 | hypothetical protein FOTG_05758 | P-loop containing nucleoside triphosphate hydrolase | 1.4 |  | 2209 | Phosphorylation |
| NODE_315.g12847.t1 | hypothetical protein FOMG_10877 | Polyketide synthase, enoylreductase domain | 4.0 |  | 333 | Biosynthetic |
| NODE_315.g12850.t1 | D-arabinose 1-dehydrogenase | NADP-dependent oxidoreductase domain | 1.6 |  | 388 | Oxidation-reduction |
| NODE_32.g3514.t1 | probable iron inhibited ABC transporter 2 | ABC transporter-like | 3.2 |  | 618 | Transport |
| NODE_32.g3528.t1 | hypothetical protein FOTG_02131 | Chaperone J-domain superfamily | 1.3 |  | 573 | Stress response |
| NODE_320.g12917.t1 | hypothetical protein FOTG_05488 | Ankyrin repeats (many copies) | 1.7 |  | 1386 | Metabolic |
| NODE_323.g12939.t1 | hypothetical protein FOTG_07588 |  | 1.9 |  | 614 |  |
| NODE_326.g12963.t1 | phosphoribosylformylglycinamidine synthase | Phosphoribosylformylglycinamidine synthase, N-terminal | 2.8 |  | 1355 | Biosynthetic |
| NODE_326.g12967.t1 | hypothetical protein FOXG_06133 |  | 9.0 |  | 129 |  |
| NODE_326.g12968.t1 | Lipoyl synthase, mitochondrial | Lipoyl synthase | 1.1 |  | 411 | Biosynthetic |
| NODE_327.g12972.t1 | Argininosuccinate synthase | Argininosuccinate synthase | 1.9 |  | 431 | Biosynthetic |
| NODE_327.g12981.t1 | hypothetical protein FOTG_08114 | U3 small nucleolar RNA-associated SSU processome protein 12 (Utp12) | 1.8 |  | 391 | rRNA processing |
| NODE_328.g12990.t1 | hypothetical protein FOPG_05207 | Alpha/beta hydrolase fold 1 | 1.3 |  | 475 | Metabolic |
| NODE_329.g13003.t1 | Mitochondrial import inner membrane translocase subunit tim54 | Tim54-like domain | 1.5 |  | 459 | Transport |
| NODE_33.g3580.t1 | Monocarboxylate transporter 8 | Major facilitator superfamily | 4.6 |  | 450 | Transport |
| NODE_33.g3598.t1 | UDP-glucose 6-dehydrogenase | UDP-glucose/GDP-mannose dehydrogenase, N-terminal | 2.4 |  | 605 | Oxidation-reduction |
| NODE_33.g3603.t1 | amidase | Amidase signature domain | 4.6 |  | 448 | Biosynthetic |
| NODE_33.g3607.t1 | hypothetical protein FOTG_06685 | Sodium:neurotransmitter symporter | 5.0 |  | 684 | Transport |
| NODE_33.g3610.t1 | 3-ketoacyl-CoA thiolase B, peroxisomal | acetyl-coenzyme A acetyltransferases (Thiolases) | 1.7 |  | 416 |  |
| NODE_33.g3629.t1 | ribose-phosphate pyrophosphokinase | Ribose-phosphate pyrophosphokinase | 2.8 |  | 503 | Biosynthetic |
| NODE_330.g13011.t1 | DNA-directed RNA polymerase I subunit RPA1 | RNA polymerase, alpha subunit | 2.7 |  | 1676 | Transcription |
| NODE_331.g13024.t1 | hypothetical protein FOC1_g10012738 | Thioredoxin-like fold | 1.8 |  | 211 |  |
| NODE_332.g13026.t1 | 40s ribosomal protein s3 | Ribosomal protein S3, C-terminal | 1.2 |  | 288 | Translation |
| NODE_334.g13053.t1 | chromodomain-helicase-DNA-binding protein 1 | SNF2-related, N-terminal domain | 2.7 |  | 1630 |  |
| NODE_337.g13086.t1 | hypothetical protein FOTG_13204 | Major facilitator, sugar transporter-like | 3.7 |  | 682 | Transport |
| NODE_34.g3665.t1 | Transcription elongation factor SPT6 | Helix-turn-helix DNA-binding domain of Spt6 | 1.1 |  | 1406 | Transcription |
| NODE_34.g3672.t1 | Isocitrate lyase | Isocitrate lyase | 7.6 |  | 546 | Metabolic |
| NODE_34.g3684.t1 | hypothetical protein FOTG_02727 | Transcription factor domain, fungi | 2.6 |  | 807 | Transcription |
| NODE_34.g3687.t1 | Putative CDP-alcohol phosphatidyltransferase class-I family protein C22A12.08c | Haloacid dehalogenase hydrolase like (HAD superfamily) | 1.8 |  | 425 |  |
| NODE_34.g3690.t1 | hypothetical protein FOIG_06953 | Ribosomal protein L22/L17 | 1.3 |  | 333 | Translation |
| NODE_34.g3697.t1 | hypothetical protein XA68_10480 | WD domain, G-beta repeat | 1.5 |  | 670 | Signal transduction |
| NODE_34.g3701.t1 | probable ribosomal protein L18, cytosolic | Ribosomal protein L18e | 1.1 |  | 184 | Translation |
| NODE_345.g13188.t1 | DNA topoisomerase 1 | DNA topoisomerase I, DNA binding, eukaryotic type | 2.6 |  | 930 | DNA topological change |
| NODE_348.g13213.t1 | 40S ribosomal protein S8 | Ribosomal protein S8e | 1.2 |  | 206 | Translation |
| NODE_349.g13218.t1 | hypothetical protein FOPG_05882 | La-type HTH domain | 4.0 |  | 775 | RNA processing |
| NODE_35.g3765.t1 | hypothetical protein FOQG_10790 | Haem peroxidase superfamily | 4.1 |  | 1426 | Proteolysis |
| NODE_35.g3800.t1 | hypothetical protein FOTG_13406 | Xaa-Pro dipeptidyl-peptidase, C-terminal | 1.2 |  | 593 | Proteolysis |
| NODE_353.g13260.t1 | hypothetical protein FOTG_00552 |  | 1.4 |  | 651 |  |
| NODE_36.g3836.t1 | hypothetical protein FPOA_01302 | Ribosomal protein S28e | 1.1 |  | 68 | Translation |
| NODE_36.g3845.t1 | hypothetical protein FOC4_g10002990 | Nitrogen regulatory protein areA, GATA-like domain | 1.9 |  | 584 | Regulation of nitrogen utilization |
| NODE_36.g3846.t1 | hypothetical protein FOTG_05810 | Ribonuclease II/R | 3.3 |  | 1046 | RNA processing |
| NODE_36.g3847.t1 | Putative beta-glucosidase btgE | Glycoside hydrolase superfamily | 1.1 |  | 610 | Metabolic |
| NODE_36.g3853.t1 | cytochrome c peroxidase, mitochondrial | Haem peroxidase | 4.1 |  | 358 | Proteolysis |
| NODE_361.g13332.t1 | hypothetical protein FOTG_07077 | Ribosomal RNA adenine methyltransferase KsgA/Erm | 2.2 |  | 594 | Methylation |
| NODE_364.g13356.t1 | hypothetical protein FOTG_01519 | Mediator complex, subunit Med12 | 1.4 |  | 1519 | Transcription |
| NODE_364.g13359.t1 | nitrogen regulatory protein areA | Zinc finger, GATA-type | 1.1 |  | 965 | Transcription |
| NODE_367.g13381.t1 | hypothetical protein FOPG_17154 | P-loop containing nucleoside triphosphate hydrolase | 6.5 |  | 1068 | Phosphorylation |
| NODE_368.g13386.t1 | hypothetical protein FOTG_15366 | F-box-like domain superfamily | 3.7 |  | 423 |  |
| NODE_369.g13392.t1 | hypothetical protein FOTG_09210 | Zn (2)-C6 fungal-type DNA-binding domain | 2.4 |  | 468 | Transcription |
| NODE_37.g3918.t1 | L-lactate dehydrogenase (cytochrome) | FMN-dependent alpha-hydroxy acid dehydrogenase, active site | 2.4 |  | 502 | Oxidation-reduction |
| NODE_37.g3925.t1 | Fatty acid synthase subunit beta | Fatty acid synthase | 3.7 |  | 2104 | Oxidation-reduction |
| NODE_37.g3926.t1 | Fatty acid synthase subunit alpha | Fatty acid synthase subunit alpha, acyl carrier domain | 3.5 |  | 1855 | Oxidation-reduction |
| NODE_379.g13482.t1 | related to NOP16 constituent of 66S pre-ribosomal particles | Ribosome biogenesis protein Nop16 | 1.4 |  | 215 | Ribosome biogenesis |
| NODE_38.g3983.t1 | ATP-dependent RNA helicase, mitochondrial | P-loop containing nucleoside triphosphate hydrolase | 2.6 |  | 611 | Phosphorylation |
| NODE_380.g13497.t1 | hypothetical protein FOCG_01770 | NmrA-like domain | 4.4 |  | 325 |  |
| NODE_382.g13514.t1 | hypothetical protein FOTG_01594 | FAD dependent oxidoreductase | 3.5 |  | 473 | Oxidation-reduction |
| NODE_382.g13515.t1 | TATA-box-binding protein | TATA-box binding protein | 1.1 | Nucleus | 254 | Transcription |
| NODE_383.g13525.t1 | related to 24-dehydrocholesterol reductase precursor | FAD linked oxidase, N-terminal | 1.4 |  | 541 | Oxidation-reduction |
| NODE_383.g13526.t1 | hydroxymethylglutaryl-CoA synthase | Hydroxymethylglutaryl-coenzyme A synthase C-terminal domain | 1.4 |  | 456 | Biosynthetic |
| NODE_385.g13542.t1 | DNA-directed RNA polymerase, mitochondrial | DNA-directed RNA polymerase, phage-type | 1.9 |  | 1420 | Transcription |
| NODE_385.g13544.t1 | hypothetical protein FOTG_04169 | SNF2-related, N-terminal domain | 2.9 |  | 871 |  |
| NODE_386.g13551.t1 | hypothetical protein FOTG_01842 | Apoptosis-antagonizing transcription factor, C-terminal | 2.1 |  | 543 | Transcription |
| NODE_386.g13556.t1 | BUD22 family protein C4F10.06 | Bud22 domain | 3.3 |  | 466 | Ribosome biogenesis |
| NODE_39.g4018.t1 | hypothetical protein FOC1_g10008301 | U3 small nucleolar RNA-associated protein 8 | 3.5 |  | 903 | rRNA processing |
| NODE_39.g4038.t1 | hypothetical protein FOC4_g10009379 | Glycosyl transferase, family 15 | 1.2 |  | 508 | Lipid glycosylation |
| NODE_39.g4041.t1 | hypothetical protein FOTG_09805 | Nitronate monooxygenase | 2.6 |  | 364 | Oxidation-reduction |
| NODE_39.g4054.t1 | hypothetical protein FOXG_10350 | WD domain, G-beta repeat | 2.0 |  | 487 | Signal transduction |
| NODE_39.g4083.t1 | hypothetical protein FOTG_09758 | Major facilitator superfamily | 1.4 |  | 577 | Transport |
| NODE_392.g13595.t1 | ABC transporter CDR4 | ABC transporter-like | 1.2 |  | 1507 | Transport |
| NODE_393.g13598.t1 | hypothetical protein FOTG_05294 | PTP type protein phosphatase | 1.5 |  | 946 | Protein dephosphorylation |
| NODE_394.g13608.t1 | hypothetical protein FOTG_06965 | ATPase, AAA-type, core | 2.5 |  | 758 | Transport |
| NODE_394.g13609.t1 | cytochrome c | Cytochrome c-like domain | 2.7 |  | 106 | Oxidation-reduction |
| NODE_40.g4107.t1 | aliphatic nitrilase | Carbon-nitrogen hydrolase | 7.3 |  | 362 | Metabolic |
| NODE_40.g4127.t1 | hypothetical protein BFJ69_g12662 |  | 3.9 |  | 288 |  |
| NODE_40.g4169.t1 | hypothetical protein FOTG_01242 | Phosphoribosyltransferase domain | 2.2 |  | 206 | Metabolic |
| NODE_406.g13703.t1 | hypothetical protein FOPG_02227 | Nucleolar protein 14 | 3.0 |  | 901 | Ribosome biogenesis |
| NODE_409.g13718.t1 | hypothetical protein FOMG_06736 | Zinc finger, GATA-type | 2.3 |  | 421 | Transcription |
| NODE_41.g4183.t1 | amidophosphoribosyltransferase | Amidophosphoribosyltransferase | 3.1 |  | 551 | Biosynthetic |
| NODE_41.g4194.t1 | hypothetical protein FOC1_g10010203 |  | 3.8 |  | 809 |  |
| NODE_41.g4208.t1 | hypothetical protein FOC4_g10011768 | Sas10/Utp3/C1D | 1.5 |  | 362 |  |
| NODE_41.g4238.t1 | hypothetical protein FOC1_g10010154 | Major facilitator superfamily | 2.2 |  | 614 | Transport |
| NODE_42.g4337.t1 | hypothetical protein BFJ69_g12033 | FAD-binding domain | 5.3 |  | 508 |  |
| NODE_420.g13790.t1 | hypothetical protein BFJ69_g12816 | Peptidase C19, ubiquitin carboxyl-terminal hydrolase | 2.2 |  | 1669 | Proteolysis |
| NODE_422.g13801.t1 | beta-galactosidase | Glycoside hydrolase, family 35 | 2.0 |  | 777 | Metabolic |
| NODE_426.g13818.t1 | hypothetical protein FPSE_09944 | Ribosomal protein L14P | 1.9 | Mitochondrion | 139 | Translation |
| NODE_428.g13827.t1 | Alternative oxidase, mitochondrial | Alternative oxidase | 3.3 |  | 353 | Oxidation-reduction |
| NODE_43.g4375.t1 | hypothetical protein FOTG_10559 | Chloride channel, voltage gated | 3.2 |  | 742 | Transport |
| NODE_43.g4382.t1 | Gluconolactonase | SMP-30/Gluconolactonase/LRE-like region | 6.5 |  | 385 | Regulation of catalytic activity |
| NODE_43.g4383.t1 | hypothetical protein BFJ69_g8391 |  | 6.9 |  | 363 |  |
| NODE_44.g4431.t1 | DNA-binding protein creA | Zinc finger C2H2-type | 1.6 |  | 420 | Transcription |
| NODE_44.g4442.t1 | hypothetical protein FOTG_07898 |  | 3.0 |  | 527 |  |
| NODE_44.g4445.t1 | hypothetical protein FOTG_07896 | Armadillo-like helical | 2.1 |  | 654 |  |
| NODE_44.g4455.t1 | Aspartate aminotransferase, cytoplasmic | Aminotransferases, class I | 2.2 |  | 414 | Biosynthetic |
| NODE_44.g4457.t1 | probable nuclear export sequence-containing nonribosomal protein | Nmd3, N-terminal | 1.9 |  | 517 |  |
| NODE_44.g4459.t1 | Phenylacetaldehyde dehydrogenase | Aldehyde dehydrogenase, C-terminal | 2.1 |  | 467 | Oxidation-reduction |
| NODE_44.g4462.t1 | hypothetical protein BFJ71_g15301 | Tetratricopeptide repeat | 3.8 |  | 1167 | RNA processing |
| NODE_44.g4463.t1 | ATP-dependent RNA helicase DBP7 | P-loop containing nucleoside triphosphate hydrolase | 1.9 |  | 739 | Phosphorylation |
| NODE_44.g4467.t1 | methyltransferase | tRNA (C5-cytosine) methyltransferase, NCL1 | 2.6 |  | 852 | tRNA methylation |
| NODE_44.g4482.t1 | molecular chaperone DnaJ | Heat shock protein DnaJ, cysteine-rich domain | 1.6 |  | 526 | Stress response |
| NODE_44.g4484.t1 | ATP-dependent RNA helicase DBP10 | P-loop containing nucleoside triphosphate hydrolase | 2.6 |  | 897 | Phosphorylation |
| NODE_44.g4491.t1 | Deoxyhypusine hydroxylase | Armadillo-like helical | 1.9 |  | 330 |  |
| NODE_440.g13902.t1 | mannitol 2-dehydrogenase | Mannitol dehydrogenase, N-terminal | 2.7 |  | 503 | Oxidation-reduction |
| NODE_440.g13908.t1 | hypothetical protein FOTG_12750 | FAD linked oxidase, N-terminal | 4.3 |  | 654 | Oxidation-reduction |
| NODE_45.g4501.t1 | dihydrolipoamide acetyltransferase component pyruvate dehydrogenase complex | Peripheral subunit-binding domain | 2.3 |  | 457 |  |
| NODE_45.g4539.t1 | hypothetical protein FOIG_04164 |  | 1.7 |  | 419 |  |
| NODE_45.g4540.t1 | ATP-dependent RNA helicase DBP2 | P-loop containing nucleoside triphosphate hydrolase | 3.0 |  | 563 | Phosphorylation |
| NODE_45.g4542.t1 | Putative NADH-ubiquinone oxidoreductase C3A11.07, mitochondrial | Pyridine nucleotide-disulphide oxidoreductase | 4.9 |  | 577 | Oxidation-reduction |
| NODE_45.g4551.t1 | DnaJ like subfamily A member 2 | Chaperone DnaJ | 3.4 |  | 434 | Stress response |
| NODE_45.g4555.t1 | NAD-specific glutamate dehydrogenase | Glutamate/phenylalanine/leucine/valine dehydrogenase, C-terminal | 2.4 |  | 1068 | Oxidation-reduction |
| NODE_45.g4558.t1 | mitofusin | P-loop containing nucleoside triphosphate hydrolase | 1.3 |  | 906 | Phosphorylation |
| NODE_46.g4605.t1 | hypothetical protein BFJ69_g2650 | Tetratricopeptide repeat | 1.6 |  | 410 | RNA processing |
| NODE_46.g4613.t1 | Ribosome biogenesis protein bms1 | AARP2CN | 3.1 |  | 1144 | Ribosome biogenesis |
| NODE_47.g4644.t1 | hypothetical protein FOXG_05017 |  | 4.2 |  | 304 |  |
| NODE_47.g4679.t1 | Isocitrate dehydrogenase | Isocitrate/isopropylmalate dehydrogenase, conserved site | 2.7 |  | 462 | Oxidation-reduction |
| NODE_47.g4692.t1 | hypothetical protein FOTG_03263 | Short-chain dehydrogenase/reductase, conserved site | 4.9 |  | 278 | Oxidation-reduction |
| NODE_471.g14082.t1 | ATP-dependent RNA helicase suv3, mitochondrial | P-loop containing nucleoside triphosphate hydrolase | 1.7 |  | 763 | Phosphorylation |
| NODE_473.g14091.t1 | Nuclear GTP-binding protein NUG1 | Guanine nucleotide-binding protein-like 3 (GTP binding) | 2.8 |  | 544 | Signal transduction |
| NODE_48.g4721.t1 | hypothetical protein FOPG_03563 |  | 2.4 |  | 668 |  |
| NODE_48.g4739.t1 | predicted protein | Translation initiation factor SUI1 | 1.4 | Cytoplasm | 127 | Translation |
| NODE_48.g4744.t1 | hypothetical protein FOTG_09283 | ML-like domain | 1.9 |  | 934 | Transport |
| NODE_48.g4755.t1 | probable sulfate adenylyltransferase | Adenylyl-sulfate kinase | 3.7 |  | 574 | Sulfate assimilation |
| NODE_487.g14164.t1 | hypothetical protein FOC4_g10013939 | F-box-like domain superfamily | 2.6 |  | 306 |  |
| NODE_489.g14174.t1 | related to 26S proteasome subunit RPN4 | Zinc finger C2H2-type | 4.5 |  | 622 | Transcription |
| NODE_49.g4771.t1 | CAMK/CAMKL/KIN4 protein kinase | Protein kinase domain | 1.2 |  | 1102 | Signal transduction |
| NODE_49.g4777.t1 | hypothetical protein FOPG_00077 | Pescadillo | 2.9 |  | 657 | Ribosome biogenesis |
| NODE_49.g4781.t1 | isoleucyl-tRNA synthetase | Isoleucine-tRNA ligase | 1.4 |  | 936 | Isoleucyl-tRNA aminoacylation |
| NODE_49.g4793.t1 | 40S ribosomal protein S11-B | Ribosomal protein S17/S11 | 1.4 |  | 167 | Translation |
| NODE_49.g4810.t1 | hypothetical protein FOXG_00982 | Hypoxia induced protein, domain | 2.3 |  | 231 |  |
| NODE_5.g793.t1 | glycerol kinase | Carbohydrate kinase, FGGY, N-terminal | 3.9 |  | 516 | Metabolic |
| NODE_5.g807.t1 | hypothetical protein FOXG_10013 |  | 1.4 |  | 370 |  |
| NODE_50.g4830.t1 | Bifunctional purine biosynthesis protein ADE17 | Phosphoribosyl aminoimidazole carboxamide formyltransferase | 2.2 |  | 595 | Biosynthetic |
| NODE_502.g14240.t1 | probable holocytochrome-c synthase | Cytochrome c/c1 haem-lyase | 1.4 |  | 318 | Oxidation-reduction |
| NODE_51.g4879.t1 | related to hexamer-binding protein HEXBP | Zinc finger, CCHC-type | 4.3 | Cytoplasm | 223 | Transcription |
| NODE_519.g14306.t1 | ribose-phosphate pyrophosphokinase | Ribose-phosphate pyrophosphokinase | 3.8 |  | 461 | Biosynthetic |
| NODE_52.g4941.t1 | hypothetical protein FOTG_01372 | Protein kinase domain histidine kinase | 1.2 |  | 1372 | Signal transduction |
| NODE_52.g4963.t1 | hypothetical protein FOTG_01343 | U3 small nucleolar RNA-associated SSU processome protein 14 (Utp14) | 1.9 |  | 887 | rRNA processing |
| NODE_53.g4990.t1 | succinyl-CoA ligase | ATP-citrate lyase/succinyl-CoA ligase | 1.5 |  | 447 | Metabolic |
| NODE_53.g4992.t1 | Iron-sulfur assembly protein 1 | FeS cluster insertion protein | 1.5 |  | 228 | protein maturation |
| NODE_53.g4999.t1 | hypothetical protein FOTG_06982 | Alpha/beta hydrolase fold 1 | 3.7 |  | 327 | Metabolic |
| NODE_53.g5000.t1 | Alanine--glyoxylate aminotransferase 1 | Aminotransferase class V domain | 1.1 |  | 460 | Biosynthetic |
| NODE_53.g5003.t1 | ATP-dependent rRNA helicase SPB4 | P-loop containing nucleoside triphosphate hydrolase | 1.8 |  | 635 | Phosphorylation |
| NODE_53.g5010.t1 | hypothetical protein BFJ69_g2310 | guanine-nucleotide exchange factors (Ras-GTPases) | 2.7 |  | 1809 | Signal transduction |
| NODE_53.g5011.t1 | 40S ribosomal protein S0 | Ribosomal protein S2 | 2.5 |  | 293 | Translation |
| NODE_53.g5014.t1 | hypothetical protein FOXG_12843 | Nop domain | 3.4 |  | 593 | Ribosome biogenesis |
| NODE_53.g5025.t1 | hypothetical protein FVEG_11599 | Polyketide synthase, enoylreductase domain | 3.0 |  | 349 | Biosynthetic |
| NODE_53.g5040.t1 | allantoinase | Amidohydrolase-related | 3.2 |  | 527 | Proteolysis |
| NODE_53.g5041.t1 | Putative hexaprenyl pyrophosphate synthase, mitochondrial | Polyprenyl synthetase | 1.4 |  | 455 | Biosynthetic |
| NODE_537.g14359.t1 | aminodeoxychorismate synthase | Glutamine amidotransferase | 2.8 |  | 830 | Metabolic |
| NODE_537.g14361.t1 | Protein mak16 | Ribosomal L28e/Mak16 | 1.2 |  | 312 | Ribosome biogenesis |
| NODE_54.g5047.t1 | hypothetical protein FOTG_01914 |  | 1.2 |  | 1156 |  |
| NODE_54.g5062.t1 | hypothetical protein FOTG_01934 | Protein Sls1 | 3.0 |  | 1269 | Translation |
| NODE_54.g5075.t1 | hypothetical protein BFJ69_g599 | 2Fe-2S ferredoxin-type iron-sulfur binding domain | 2.8 |  | 560 |  |
| NODE_549.g14398.t1 | sulfite reductase (NADPH) flavoprotein alpha-component | Sulfite reductase [NADPH] flavoprotein alpha-component-like, FAD-binding | 3.7 |  | 1065 | Oxidation-reduction |
| NODE_55.g5156.t1 | hypothetical protein FOTG_13023 | Glycosyltransferase family 1 | 6.4 |  | 803 | Lipid glycosylation |
| NODE_551.g14401.t1 | hypothetical protein BFJ71_g14823 | RGS domain, regulator of G protein signaling | 3.6 |  | 322 | Signal transduction |
| NODE_556.g14415.t1 | hypothetical protein FOXG_08701 | WD domain, G-beta repeat | 2.6 |  | 431 | Signal transduction |
| NODE_560.g14425.t1 | hypothetical protein FOTG_02943 | Ran binding domain | 1.3 |  | 1201 | Transport |
| NODE_562.g14432.t1 | acetolactate synthase I/II/III large subunit | Thiamine pyrophosphate enzyme, central domain | 1.7 |  | 684 |  |
| NODE_57.g5252.t1 | hypothetical protein FOTG_06876 | Homeobox-like domain superfamily | 2.7 |  | 983 |  |
| NODE_57.g5253.t1 | hypothetical protein FOTG_06875 | P-loop containing nucleoside triphosphate hydrolase | 2.7 |  | 566 | Phosphorylation |
| NODE_57.g5262.t1 | Ubiquitin-conjugating enzyme | Ubiquitin-conjugating enzyme E2 | 1.0 | Cytoplasm | 147 |  |
| NODE_57.g5292.t1 | S-(hydroxymethyl)glutathione synthase | Glutathione-dependent formaldehyde-activating enzyme | 6.6 |  | 227 | Catabolic |
| NODE_58.g5368.t1 | Putative importin subunit beta 4 | Importin-beta, N-terminal domain | 2.8 |  | 1098 | Transport |
| NODE_59.g5398.t1 | hypothetical protein FOXG_13238 |  | 7.5 |  | 340 |  |
| NODE_59.g5421.t1 | MFS transporter, SP family, general alpha glucoside:H+ symporter | Major facilitator, sugar transporter-like | 7.1 |  | 537 | Transport |
| NODE_6.g820.t1 | hypothetical protein FOTG_09553 | FAD dependent oxidoreductase | 4.6 |  | 477 | Oxidation-reductiion |
| NODE_6.g824.t1 | hypothetical protein FOTG_09559 | RTA-like protein | 3.9 |  | 288 |  |
| NODE_6.g832.t1 | Proline-specific permease | Amino acid/polyamine transporter I | 3.4 |  | 536 | Transport |
| NODE_6.g951.t1 | hypothetical protein BFJ69_g6821 | Alpha/beta hydrolase fold 1 | 2.8 |  | 539 | Metabolic |
| NODE_60.g5450.t1 | hypothetical protein FOXG_13834 | SH3-like domain superfamily | 1.2 |  | 380 | Translation |
| NODE_60.g5459.t1 | hypothetical protein FOC1_g10007236 | RNA recognition motif domain | 2.3 |  | 369 | Cytokinesis |
| NODE_60.g5481.t1 | putative transporter C36.03c | Major facilitator superfamily | 1.1 |  | 633 | Transport |
| NODE_60.g5487.t1 | hypothetical protein FOXG_13874 | Protein of unknown function DUF3984 | 1.4 |  | 370 |  |
| NODE_60.g5488.t1 | elongator complex protein 1 | Elongator complex protein 1 | 2.6 |  | 1297 | tRNA wobble uridine modification |
| NODE_60.g5499.t1 | ATP-dependent RNA helicase HAS1 | P-loop containing nucleoside triphosphate hydrolase | 2.5 |  | 587 | Phosphorylation |
| NODE_606.g14544.t1 | pre-mRNA-splicing factor spp42 | Pre-mRNA-processing-splicing factor 8, U6-snRNA-binding | 1.2 |  | 2323 | Ribosome biogenesis |
| NODE_61.g5514.t1 | hypothetical protein BFJ70_g11808 | Protein kinase domain | 2.9 |  | 422 | Signal transduction |
| NODE_61.g5516.t1 | 4,5-DOPA dioxygenase extradiol-like protein | Extradiol aromatic ring-opening dioxygenase, DODA-type | 1.9 |  | 287 | Oxidation-reduction |
| NODE_61.g5520.t1 | Sulfite reductase | Nitrite/sulphite reductase 4Fe-4S domain | 2.7 |  | 1537 | Oxidation-reduction |
| NODE_61.g5524.t1 | hypothetical protein BFJ70_g11791 | Ribosome biogenesis protein Nop53/GLTSCR2 | 1.6 |  | 439 | Ribosome biogenesis |
| NODE_61.g5542.t1 | Malate dehydrogenase, mitochondrial | Lactate dehydrogenase/glycoside hydrolase, family 4, C-terminal | 1.9 |  | 336 | Oxidation-reduction |
| NODE_61.g5558.t1 | hypothetical protein FOTG_08988 | Transcription factor domain, fungi | 1.2 |  | 720 | Transcription |
| NODE_610.g14549.t1 | hypothetical protein FOTG_11411 |  | 5.2 |  | 875 |  |
| NODE_618.g14560.t1 | Ras-like protein | GTPase (Ras type) | 2.0 | Cell membrane | 216 | Signal Transduction |
| NODE_62.g5572.t1 | putative UTP--glucose-1-phosphate uridylyltransferase | UTP--glucose-1-phosphate uridylyltransferase | 2.1 |  | 513 | Metabolic |
| NODE_62.g5607.t1 | hypothetical protein FOQG_09647 | WD domain, G-beta repeat | 2.0 |  | 581 | Signal transduction |
| NODE_62.g5622.t1 | AGC/RSK/RSKP70 protein kinase | Protein kinase domain AGC-kinase, C-terminal | 2.6 |  | 515 | Signal transduction |
| NODE_63.g5636.t1 | related to serine/threonine protein kinase | Protein kinase domain | 1.4 |  | 522 | Signal transduction |
| NODE_63.g5649.t1 | hypothetical protein FSPOR_8584 | Zinc finger C2H2-type | 2.1 |  | 436 | Transcription |
| NODE_63.g5661.t1 | Cutinase | Cutinase | 2.0 |  | 426 |  |
| NODE_63.g5663.t1 | Putative N-acetylglucosamine-6-phosphate deacetylase | N-acetylglucosamine-6-phosphate deacetylase | 2.8 |  | 444 | Metabolic |
| NODE_634.g14583.t1 | related to Eukaryotic translation initiation factor 3 subunit J | Eukaryotic translation initiation factor 3 subunit J | 3.9 |  | 276 | Translation |
| NODE_634.g14585.t1 | Pre-rRNA-processing protein PNO1 | K Homology domain, type 1 superfamily | 2.2 |  | 256 |  |
| NODE_64.g5701.t1 | hypothetical protein FOC4_g10001384 | Letm1 ribosome-binding domain | 1.5 |  | 552 |  |
| NODE_64.g5731.t1 | hypothetical protein BFJ66_g5886 | Zn (2)-C6 fungal-type DNA-binding domain-GAL4 | 3.3 |  | 825 | Transcription |
| NODE_649.g14609.t1 | Betaine aldehyde dehydrogenase | Aldehyde dehydrogenase domain | 2.1 |  | 427 | Oxidation-reduction |
| NODE_649.g14610.t1 | choline dehydrogenase | Glucose-methanol-choline oxidoreductase, N-terminal | 1.6 |  | 543 | Oxidation-reduction |
| NODE_65.g5772.t1 | hypothetical protein BFJ69_g1977 | Expansin, cellulose-binding-like domain superfamily | 5.6 | Extracellular | 220 | Metabolic |
| NODE_65.g5803.t1 | hypothetical protein FPOA_05897 | RNA polymerase archaeal subunit P/eukaryotic subunit RPABC4 | 1.1 | Cytoplasm | 72 | Transcription |
| NODE_66.g5818.t1 | probable GMP synthase | GMP synthase, C-terminal | 3.5 |  | 544 | Biosynthetic |
| NODE_66.g5822.t1 | CK1/CK1/CK1-D protein kinase | Protein kinase domain | 2.8 |  | 371 | Signal transduction |
| NODE_66.g5838.t1 | uncharacterized protein FVRRES_05356 | Ribosomal protein S4e, N-terminal | 1.3 |  | 261 | Translation |
| NODE_66.g5840.t1 | fumarate hydratase, mitochondrial | Fumarate hydratase, class II | 2.3 |  | 529 | Metabolic |
| NODE_69.g5976.t1 | probable translation initiation factor eIF-2 gamma chain | Translation initiation factor 2, gamma subunit, C-terminal | 1.5 |  | 509 | Translation |
| NODE_69.g5978.t1 | hypothetical protein FAVG1_12529 | P-loop containing nucleoside triphosphate hydrolase | 2.8 |  | 396 | Phosphorylation |
| NODE_69.g5990.t1 | 60S acidic ribosomal protein P0 | 60S ribosomal protein L10P | 1.1 |  | 313 | Translation |
| NODE_69.g5995.t1 | glycogen | Glycogen synthase | 2.8 |  | 705 | Biosynthetic |
| NODE_69.g5996.t1 | ribosome biogenesis protein ytm-1 | WD domain, G-beta repeat | 1.7 |  | 475 | Signal transduction |
| NODE_69.g6013.t1 | ATP-dependent RNA helicase DED1 | P-loop containing nucleoside triphosphate hydrolase | 4.4 |  | 670 | Phosphorylation |
| NODE_69.g6015.t1 | Ribosomal RNA-processing protein 9 | WD domain, G-beta repeat | 2.3 |  | 582 | Signal transduction |
| NODE_694.g14678.t1 | propionyl-CoA synthetase | AMP-dependent synthetase/ligase | 5.1 |  | 700 |  |
| NODE_70.g6030.t1 | beta-fructofuranosidase | Glycosyl hydrolase family 32, N-terminal | 6.2 |  | 546 | Metabolic |
| NODE_70.g6068.t1 | Peroxisomal primary amine oxidase | Copper amine oxidase, catalytic domain | 7.0 |  | 702 | Oxidation-reduction |
| NODE_70.g6070.t1 | Aldehyde dehydrogenase | Aldehyde dehydrogenase domain | 4.0 |  | 501 | Oxidation-reduction |
| NODE_70.g6076.t1 | hypothetical protein BFJ69_g1208 | Acyl-CoA N-acyltransferase | 6.3 |  | 286 | Biosynthetic |
| NODE_70.g6081.t1 | Peroxisomal acyl-coenzyme A oxidase 1 | Acyl-CoA oxidase/dehydrogenase, central domain | 1.9 |  | 698 | Oxidation-reduction |
| NODE_71.g6100.t1 | hypothetical protein FOXG_04143 | Polyketide synthase, enoylreductase domain | 3.2 |  | 331 | Biosynthetic |
| NODE_71.g6107.t1 | aspartyl-tRNA synthetase | Aminoacyl-tRNA synthetase, class II (D/K/N) | 1.7 |  | 967 | tRNA aminoacylation |
| NODE_71.g6114.t1 | 54S ribosomal protein L3, mitochondrial | Ribonuclease III, endonuclease domain superfamily | 1.6 |  | 383 | RNA processing |
| NODE_71.g6116.t1 | hypothetical protein BFJ65_g4001 | La-type HTH domain | 3.1 |  | 398 | RNA processing |
| NODE_71.g6128.t1 | hypothetical protein BFJ70_g12854 | Translation elongation factor, IF5A C-terminal | 1.0 |  | 164 | Translation |
| NODE_72.g6147.t1 | hypothetical protein BFJ69_g11453 | Calcium-dependent channel, 7TM region, putative phosphate | 1.3 |  | 1046 | Transport |
| NODE_72.g6181.t1 | hypothetical protein FOPG_02419 |  | 2.6 | Extracellular | 424 |  |
| NODE_72.g6184.t1 | Putative tartrate dehydrogenase/decarboxylase ttuC | Isocitrate/isopropylmalate dehydrogenase | 1.9 |  | 369 | Oxidation-reduction |
| NODE_73.g6200.t1 | Putative helicase C6F12.16c | P-loop containing nucleoside triphosphate hydrolase | 2.9 |  | 1094 | Phosphorylation |
| NODE_73.g6223.t1 | related to WSS1 Protein involved in sister chromatid separation and segregation | WLM domain | 2.1 |  | 348 |  |
| NODE_74.g6266.t1 | Threonine synthase | Threonine synthase, N-terminal | 1.7 |  | 539 | Biosynthetic |
| NODE_74.g6303.t1 | probable isocitrate dehydrogenase | Isocitrate/isopropylmalate dehydrogenase | 2.6 |  | 378 | Oxidation-reduction |
| NODE_74.g6307.t1 | polyadenylate-binding protein | RNA recognition motif domain | 1.1 |  | 416 | Cytokinesis |
| NODE_75.g6314.t1 | Ribosomal RNA assembly protein KRR1 | Krr1, KH1 domain | 1.6 |  | 312 |  |
| NODE_75.g6322.t1 | hypothetical protein BFJ69_g11508 | Armadillo-type fold | 1.8 |  | 812 |  |
| NODE_75.g6330.t1 | hypothetical protein FOC1_g10010277 | Mitochondrial substrate/solute carrier | 2.2 |  | 402 | Transport |
| NODE_75.g6336.t1 | hypothetical protein FOXG_08482 | RGS domain, Regulator of G protein signaling | 3.1 |  | 722 | Signal transduction |
| NODE_75.g6341.t1 | hypothetical protein FOXG_08476 | Bystin | 1.7 |  | 485 | Ribosome biogenesis |
| NODE_75.g6355.t1 | hypothetical protein FOQG_05736 | H/ACA ribonucleoprotein complex, subunit Gar1/Naf1 | 2.1 |  | 560 | Ribosome biogenesis |
| NODE_76.g6385.t1 | hypothetical protein BFJ71_g5717 | Transcription factor domain, fungi | 4.0 |  | 490 | Transcription |
| NODE_76.g6409.t1 | hypothetical protein FOC1_g10006143 |  | 3.5 |  | 210 |  |
| NODE_77.g6452.t1 | hypothetical protein FOMG_09609 | Patatin-like phospholipase domain | 4.6 |  | 567 | Metabolic |
| NODE_77.g6485.t1 | hypothetical protein FOTG_10772 | Heterokaryon incompatibility | 4.6 |  | 810 |  |
| NODE_78.g6500.t1 | hypothetical protein BFJ69_g6102 | Chaperone J-domain superfamily | 2.6 |  | 786 | Stress response |
| NODE_78.g6527.t1 | probable MRT4-mRNA turnover 4 | Ribosomal protein L10P | 2.6 |  | 244 | Translation |
| NODE_78.g6540.t1 | hypothetical protein BFJ69_g6125 |  | 2.4 |  | 120 |  |
| NODE_78.g6545.t1 | 50S ribosomal protein L24e | Ribosomal protein L24e | 2.3 |  | 220 | Translation |
| NODE_79.g6573.t1 | Eukaryotic translation initiation factor 3 subunit B | Translation initiation factor, beta propellor-like domain | 1.5 |  | 747 | Translation |
| NODE_8.g1187.t1 | hypothetical protein BFJ70_g669 |  | 4.0 |  | 240 |  |
| NODE_80.g6607.t1 | hypothetical protein BFJ66_g7752 | Growth factor receptor cysteine-rich domain superfamily | 5.9 | Extracellular | 168 |  |
| NODE_81.g6667.t1 | translation initiation factor RLI1 | ABC transporter-like | 2.1 |  | 607 | Transport |
| NODE_81.g6668.t1 | hypothetical protein FOTG_00453 | FF domain | 1.1 |  | 784 |  |
| NODE_81.g6696.t1 | Pyruvate carboxylase | Carbamoyl-phosphate synthetase large subunit-like, ATP-binding domain | 2.3 |  | 1197 | Metabolic |
| NODE_82.g6716.t1 | probable formate dehydrogenase | D-isomer specific 2-hydroxyacid dehydrogenase, NAD-binding domain | 1.8 |  | 365 | Phosphorylation |
| NODE_82.g6723.t1 | probable Tim22, subunit of the Tim22-complex | Tim17/Tim22/Tim23/Pmp24 family | 1.2 |  | 177 | Transport |
| NODE_82.g6731.t1 | Ribosomal RNA-processing protein 15 | Ribosomal RNA-processing protein 15 | 1.1 |  | 300 | rRNA processing |
| NODE_82.g6742.t1 | hypothetical protein FOQG_02431 | P-loop containing nucleoside triphosphate hydrolase | 2.1 |  | 1211 | Phosphorylation |
| NODE_82.g6756.t1 | hypothetical protein FOXG_07797 | Glycoside hydrolase, family 61 | 4.0 |  | 404 | Metabolic |
| NODE_83.g6768.t1 | Protein gar2 | RNA recognition motif domain | 3.0 |  | 501 | Cytokinesis |
| NODE_83.g6787.t1 | 50s ribosomal l6e | 60S ribosomal protein L6E | 1.7 |  | 200 | Translation |
| NODE_83.g6791.t1 | Mitochondrial ATPase complex subunit ATP10 | ATPase assembly factor ATP10 | 1.9 |  | 345 | Transport |
| NODE_83.g6793.t1 | 60S ribosomal protein L35 | Ribosomal protein L29/L35 | 1.4 |  | 124 | Translation |
| NODE_83.g6798.t1 | hypothetical protein FOC4_g10015188 | Protein of unknown function DUF3712 | 5.2 |  | 343 |  |
| NODE_83.g6804.t1 | Amino-acid transporter arg-13 | Mitochondrial substrate/solute carrier | 1.4 |  | 331 | Transport |
| NODE_83.g6812.t1 | hypothetical protein BFJ65_g1762 |  | 3.4 |  | 406 |  |
| NODE_84.g6859.t1 | hypothetical protein BFJ69_g21 | Major facilitator superfamily | 5.4 |  | 519 | Transport |
| NODE_84.g6860.t1 | hypothetical protein FOTG_14057 |  | 7.1 |  | 107 |  |
| NODE_85.g6901.t1 | hypothetical protein FOC4_g10003170 |  | 6.3 |  | 225 |  |
| NODE_85.g6923.t1 | hypothetical protein BFJ69_g9090 | Amino acid transporter, transmembrane domain | 1.9 |  | 465 | Transport |
| NODE_86.g6951.t1 | probable FBP1-fructose-1,6-bisphosphatase | Fructose-1-6-bisphosphatase class I, N-terminal | 3.9 |  | 342 | Metabolic |
| NODE_86.g6952.t1 | hypothetical protein FAVG1_03363 | Peroxisomal biogenesis factor 11 | 1.3 |  | 235 | Peroxisome fission |
| NODE_86.g6968.t1 | hypothetical protein FOTG_09455 |  | 1.6 |  | 852 |  |
| NODE_86.g6969.t1 | probable general amino acid permease | Amino acid permease/ SLC12A domain | 4.6 |  | 558 | Transport |
| NODE_86.g6970.t1 | hypothetical protein FOXG_05940 | Flavin prenyltransferase-like | 1.4 |  | 589 | Oxidation-reduction |
| NODE_86.g6971.t1 | ribose-phosphate pyrophosphokinase 3 | Ribose-phosphate pyrophosphokinase | 2.7 |  | 323 | Biosynthetic |
| NODE_86.g6987.t1 | Transcriptional regulatory protein SIN3 | Paired amphipathic helix superfamily | 2.0 |  | 1522 | Transcription |
| NODE_87.g6989.t1 | hypothetical protein FOTG_06214 | DNA-directed RNA polymerase I, subunit RPA34.5 | 3.3 |  | 664 | Transcription |
| NODE_87.g6997.t1 | acetyl-coenzyme A synthetase | AMP-dependent synthetase/ligase | 4.1 |  | 721 |  |
| NODE_87.g7018.t1 | hypothetical protein FOXG_00760 | Mitochondrial substrate/solute carrier | 1.8 |  | 346 | Transport |
| NODE_87.g7021.t1 | molecular chaperone GrpE | GrpE nucleotide exchange factor | 1.1 |  | 244 | Stress response |
| NODE_89.g7109.t1 | hypothetical protein FOTG_13072 | Oxidoreductase, molybdopterin-binding domain | 4.5 |  | 357 | Oxidation-reduction |
| NODE_89.g7110.t1 | hypothetical protein FOTG_13073 | Aminotransferase, class I/class II | 6.1 |  | 423 | Biosynthetic |
| NODE_9.g1253.t1 | RIO kinase 1 | Protein kinase-like domain-RIO kinase | 3.8 |  | 544 | Signal transduction |
| NODE_9.g1257.t1 | Arginine permease | Amino acid permease, conserved site | 3.1 |  | 515 | Transport |
| NODE_9.g1263.t1 | hypothetical protein FOXG_02452 | Peptidase C2, calpain, catalytic domain | 3.1 |  | 966 | Proteolysis |
| NODE_9.g1319.t1 | Succinate/fumarate mitochondrial transporter | Mitochondrial carrier protein | 6.7 |  | 346 | Transport |
| NODE_9.g1325.t1 | arginyl-tRNA synthetase | Arginyl tRNA synthetase N-terminal domain | 1.4 |  | 635 | Arginyl-tRNA aminoacylation |
| NODE_9.g1334.t1 | probable acetyl-CoA carboxylase | Acetyl-CoA carboxylase, central domain | 2.7 |  | 2284 | Biosynthetic |
| NODE_9.g1341.t1 | tRNA pseudouridine synthase 1 | Pseudouridine synthase I, TruA, alpha/beta domain | 2.6 |  | 604 | RNA modification |
| NODE_9.g1342.t1 | 40S ribosomal protein S7 | Ribosomal protein S7e | 1.9 |  | 203 | Translation |
| NODE_93.g7318.t1 | Putative RING finger protein C6B12.07c | SPX domain | 1.2 |  | 512 |  |
| NODE_93.g7326.t1 | MRS4-like protein | Mitochondrial substrate/solute carrier | 1.9 |  | 260 | Transport |
| NODE_93.g7332.t1 | hypothetical protein FOTG_07438 | MIF4G-like, type 3 | 1.6 |  | 871 |  |
| NODE_93.g7360.t1 | hypothetical protein FPSE_12273, partial | H/ACA ribonucleoprotein complex, subunit Gar1/Naf1 | 3.1 |  | 210 | Ribosome biogenesis |
| NODE_94.g7381.t1 | hypothetical protein BFJ65_g18018 | U3 small nucleolar RNA-associated SSU processome protein (Utp11) | 1.2 |  | 268 | rRNA processing |
| NODE_94.g7386.t1 | hypothetical protein BFJ69_g8554 |  | 1.7 |  | 157 |  |
| NODE_94.g7393.t1 | hypothetical protein FOMG_11532 | Oligopeptide transporter, OPT superfamily | 4.5 |  | 801 | Transport |
| NODE_94.g7409.t1 | kynureninase 2 | Kynureninase | 1.5 |  | 493 | Biosynthetic |
| NODE_95.g7414.t1 | hypothetical protein FPSE_08661 | Ribosomal protein S11 | 1.6 |  | 151 | Translation |
| NODE_95.g7415.t1 | 60S ribosomal protein L7 | Ribosomal protein L30 | 1.2 |  | 248 | Translation |
| NODE_95.g7417.t1 | Eukaryotic translation initiation factor 3 subunit C | Eukaryotic translation initiation factor 3 subunit C, N-terminal domain | 1.3 |  | 857 | Translation |
| NODE_95.g7439.t1 | Bifunctional protein RIB2 | Pseudouridine synthase, RsuA/RluA | 1.8 |  | 514 | RNA modification |
| NODE_95.g7440.t1 | hypothetical protein FOPG_06852 | bZIP_YAP | 3.6 |  | 513 | Transcription |
| NODE_95.g7451.t1 | ATP-dependent RNA helicase DBP3 | P-loop containing nucleoside triphosphate hydrolase | 3.5 |  | 586 | Phosphorylation |
| NODE_97.g7493.t1 | hypothetical protein BFJ65_g4243 | CFEM domain | 2.3 |  | 314 |  |
| NODE_97.g7508.t1 | Orotidine 5'-phosphate decarboxylase | Orotidine 5'-phosphate decarboxylase domain | 2.0 |  | 366 | Biosynthetic |
| NODE_97.g7510.t1 | hypothetical protein BFJ68_g10103 | Zinc finger, CCCH-type | 2.6 |  | 457 | Transcription |
| NODE_97.g7522.t1 | guanosine-diphosphatase | Nucleoside phosphatase GDA1/CD39 | 1.8 |  | 545 |  |
| NODE_97.g7532.t1 | probable ubiquitin-conjugating enzyme CDC34 | Ubiquitin-conjugating enzyme E2 | 1.3 |  | 224 |  |
| NODE_98.g7549.t1 | hypothetical protein FOTG_00705 | FYVE zinc finger | 1.9 |  | 352 | Transcription |
| NODE_98.g7556.t1 | related to SRP40 Suppressor of mutant AC40 of RNA polymerase I and III | Immunoglobulin-like fold | 1.1 |  | 318 |  |
| NODE_98.g7565.t1 | hypothetical protein FOTG_03037 | bZIP | 2.3 |  | 526 | Transcription |
| NODE_98.g7580.t1 | hypothetical protein BFJ69_g12427 | RNA recognition motif domain | 1.4 |  | 422 | Cytokinesis |
| NODE_99.g7594.t1 | ribosome-interacting gtpase 1 | GTP binding domain | 1.9 |  | 367 | Signal transduction |
| NODE_99.g7598.t1 | Eukaryotic translation initiation factor 3 subunit A | Proteasome component (PCI) domain | 1.2 |  | 1056 |  |
| NODE_99.g7616.t1 | tRNA-dihydrouridine synthase 1 | tRNA-dihydrouridine synthase | 2.7 |  | 508 | Oxidation-reduction |
| DN10_c0_g1_i1.g4247.t1 | Phospho-2-dehydro-3-deoxyheptonate aldolase, tyrosine-inhibited | DAHP synthetase I/KDSA | 2.1 |  | 283 | Biosynthetic |
| DN10023_c0_g1_i1.g15676.t1 | hypothetical protein FOTG_07507 | U3 small nucleolar RNA-associated protein 22 | 2.2 |  | 567 | rRNA processing |
| DN10030_c0_g1_i1.g15705.t1 | probable heat shock protein 70 | Heat shock protein 70 family | 5.3 |  | 485 | Stress response |
| DN1004_c0_g1_i5.g38759.t1 | hypothetical protein FOTG_02891 | Fork head domain | 2.2 |  | 1313 | Transcription |
| DN10041_c0_g1_i1.g15700.t1 | Peroxisomal acyl-coenzyme A oxidase 1 | Acyl-CoA oxidase, C-terminal | 1.8 |  | 704 | Fatty acid beta-oxidation |
| DN1006_c0_g1_i1.g13636.t1 | hypothetical protein BFJ70_g8234 | von Willebrand factor, type A | 2.6 |  | 808 |  |
| DN10068_c0_g1_i1.g15685.t1 | hypothetical protein FOXG_10350 | WD domain, G-beta repeat | 1.9 |  | 487 | Signal transduction |
| DN10070_c0_g1_i1.g15683.t1 | hypothetical protein FOC4_g10012609 | Amine oxidase | 1.6 |  | 519 | Oxidation-reduction |
| DN1012_c0_g1_i4.g39599.t1 | ribonuclease Z | Metallo-beta-lactamase | 3.0 |  | 836 |  |
| DN10140_c1_g1_i1.g13232.t1 | Eukaryotic translation initiation factor 3 subunit M | Proteasome component (PCI) domain | 1.9 |  | 451 |  |
| DN10164_c0_g1_i1.g13220.t1 | Nuclear GTP-binding protein NUG1 | Guanine nucleotide-binding protein-like 3 (GTP) | 2.7 |  | 541 | Signal transduction |
| DN10165_c0_g1_i1.g13244.t1 | hypothetical protein FOTG_05294 | PTP type protein phosphatase | 1.4 |  | 946 | Protein dephosphorylation |
| DN10193_c0_g1_i1.g13276.t1 | hypothetical protein FOMG_06847 | Tetratricopeptide repeat | 2.1 |  | 659 | RNA processing |
| DN1020_c0_g1_i2.g13647.t1 | probable iron inhibited ABC transporter 2 | ABC transporter-like | 3.1 |  | 618 | Transport |
| DN10249_c0_g1_i1.g12851.t1 | hypothetical protein BFJ69_g14497 | Glycoside hydrolase superfamily | 1.6 |  | 432 | Metabolic |
| DN10254_c0_g1_i1.g12841.t1 | ATP-dependent RNA helicase, mitochondrial | P-loop containing nucleoside triphosphate hydrolase | 2.4 |  | 575 | Phosphorylation |
| DN10292_c0_g1_i1.g12862.t1 | hypothetical protein BFJ72_g8252 |  | 4.8 | Cytoplasm | 261 |  |
| DN10319_c0_g1_i1.g17247.t1 | hypothetical protein FOC4_g10009379 | Glycosyl transferase, family 15 | 1.1 |  | 508 | Protein glycosylation |
| DN10351_c0_g1_i1.g17239.t1 | putative pyrroline-5-carboxylate reductase | Pyrroline-5-carboxylate reductase, dimerisation domain | 3.6 |  | 343 | Oxidation-reduction |
| DN1037_c0_g1_i15.g40088.t1 | hypothetical protein BFJ71_g9758 | S-adenosyl-L-methionine-dependent methyltransferase | 3.0 |  | 1458 | Methylation |
| DN1045_c0_g1_i1.g13326.t1 | Serine/threonine-protein kinase oca2 | Protein kinase domain | 2.0 |  | 765 | Signal transduction |
| DN1049_c0_g1_i2.g13661.t1 | probable sulfate adenylyltransferase | Sulphate adenylyltransferase catalytic domain | 3.6 |  | 574 | Sulfate assimilation |
| DN1053_c0_g1_i4.g13649.t1 | hypothetical protein FPSE_00523 | Ribosomal protein L13 | 1.7 |  | 202 | Translation |
| DN10553_c0_g1_i1.g10224.t1 | hypothetical protein FOC1_g10005709 | FAD/NAD(P)-binding domain superfamily | 4.3 | Mitochondrion | 199 |  |
| DN1058_c0_g1_i1.g40123.t1 | hypothetical protein BFJ71_g8322 |  | 4.4 |  | 253 |  |
| DN1059_c0_g1_i2.g13371.t1 | Peroxisomal adenine nucleotide transporter 1 | Mitochondrial substrate/solute carrier | 1.5 |  | 340 | Transport |
| DN106_c0_g1_i1.g1562.t1 | hypothetical protein FAVG1_01712 | IMP dehydrogenase/GMP reductase | 1.9 |  | 532 | Oxidation-reduction |
| DN10611_c0_g1_i1.g12473.t1 | 4,5-DOPA dioxygenase extradiol-like protein | Extradiol ring-cleavage dioxygenase, class III enzyme, subunit B | 1.8 |  | 287 | Metabolic |
| DN1062_c0_g1_i5.g39219.t1 | hypothetical protein BFJ65_g11565 | Glycosyl transferase CAP10 domain | 4.8 |  | 633 | Protein glycosylation |
| DN10680_c0_g1_i1.g12474.t1 | hypothetical protein FOTG_03996 | Sas10 C-terminal domain | 2.6 |  | 616 |  |
| DN1069_c0_g1_i1.g39362.t1 | hypothetical protein BFJ69_g6821 | Carboxylesterase, type B | 2.7 |  | 539 |  |
| DN1076_c0_g1_i2.g13421.t1 | Alternative oxidase, mitochondrial | Alternative oxidase | 3.2 |  | 353 | Oxidation-reduction |
| DN1077_c0_g1_i4.g13592.t1 | preprotein translocase subunit YidC | Membrane insertase YidC/ALB3/OXA1/COX18 | 1.7 |  | 484 |  |
| DN10799_c0_g1_i1.g12502.t1 | hypothetical protein BFJ65_g10914 | SNF2-related, N-terminal domain | 4.2 |  | 164 |  |
| DN1081_c0_g1_i2.g13439.t1 | hypothetical protein FOTG_14309 | Protein kinase domain-Histidine kinase/HSP90-like ATPase | 1.7 |  | 966 | Signal transduction |
| DN1086_c0_g1_i1.g13375.t1 | nucleolar GTP-binding protein | Nucleolar GTP-binding protein 1 | 3.3 |  | 659 | Signal Transduction |
| DN10864_c0_g1_i1.g14247.t1 | hypothetical protein BFJ69_g13977 | Major facilitator superfamily | 2.4 |  | 505 | Transport |
| DN10917_c0_g1_i1.g49587.t1 | hypothetical protein FOTG_00827 | U3 small nucleolar RNA-associated SSU processome protein (Utp13 protein) | 1.9 |  | 885 | rRNA processing |
| DN10992_c0_g1_i1.g14818.t1 | rRNA biogenesis protein rrp5 | S1 domain | 5.6 |  | 102 |  |
| DN1101_c0_g1_i1.g17854.t1 | solute carrier family 25, member 46 | Mitochondrial substrate/solute carrier | 1.3 |  | 310 | Transport |
| DN11046_c0_g1_i1.g11561.t1 | probable general amino acid permease | Amino acid permease/ SLC12A domain | 4.4 |  | 374 | Transport |
| DN11070_c0_g1_i1.g11576.t1 | Calcium-transporting ATPase 2 | Cation-transporting P-type ATPase, C-terminal | 5.5 |  | 784 | Transport |
| DN11073_c0_g1_i1.g11554.t1 | hypothetical protein FOTG_09570 |  | 6.6 |  | 394 |  |
| DN1111_c0_g1_i1.g17654.t1 | carnitine O-acetyltransferase | Choline/carnitine acyltransferase domain | 2.2 |  | 610 | Proteolysis |
| DN11110_c0_g1_i1.g49980.t1 | hypothetical protein BFJ69_g14497 | Glycoside hydrolase family 18, catalytic domain | 1.6 |  | 432 | Metabolic |
| DN11149_c0_g1_i1.g49970.t1 | hypothetical protein FOTG_03658 | Ribosomal protein Rsm22-like | 1.4 |  | 742 | Translation |
| DN1118_c0_g1_i5.g17603.t1 | hypothetical protein BFJ69_g557 | Methyltransferase domain 25 | 1.1 |  | 395 | Methylation |
| DN11280_c0_g1_i1.g9714.t1 | Plasma membrane fusion protein PRM1 |  | 3.5 |  | 153 |  |
| DN1130_c0_g1_i5.g43828.t1 | hypothetical protein FPSE_09944 | Ribosomal protein L14P | 1.8 | Mitochondrion | 139 | Translation |
| DN11384_c0_g1_i1.g11519.t1 | DNA-directed RNA polymerase III subunit RPC2 | RNA polymerase Rpb2, domain 7 | 1.3 |  | 1030 | Transcription |
| DN11394_c0_g1_i1.g11540.t1 | mitofusin | Dynamin superfamily | 1.2 |  | 906 |  |
| DN11396_c0_g1_i1.g11515.t1 | ATP-dependent RNA helicase DED1 | P-loop containing nucleoside triphosphate hydrolase | 4.3 |  | 670 | Phosphorylation |
| DN11398_c0_g1_i1.g11535.t1 | hypothetical protein BFJ69_g7791 | Transcription factor domain, fungi | 3.8 | Nucleus | 167 | Transcription |
| DN1140_c0_g1_i1.g17577.t1 | 40S ribosomal protein S9, mitochondrial | Ribosomal protein S9 | 1.2 |  | 314 | Translation |
| DN11417_c0_g1_i2.g3786.t1 | molecular chaperone DnaJ | Chaperone DnaJ, C-terminal | 1.5 |  | 522 | Stress response |
| DN1142_c0_g1_i4.g43698.t1 | related to nuclear WD protein PRL1 | Small MutS-related domain | 3.9 |  | 234 | Metabolic |
| DN11441_c0_g1_i1.g3802.t1 | hypothetical protein BFJ72_g11734 | GDP/GTP exchange factor Sec2, N-terminal | 1.1 |  | 673 |  |
| DN11444_c0_g1_i1.g3761.t1 | hypothetical protein FOTG_09283 | ML-like domain | 1.8 |  | 934 | Transport |
| DN11460_c0_g1_i1.g3756.t1 | probable acetyl-CoA carboxylase | Carbamoyl-phosphate synthetase large subunit-like, ATP-binding domain | 2.6 |  | 2284 | Metabolic |
| DN11474_c0_g1_i1.g3790.t1 | hypothetical protein FOTG_05810 | Ribonuclease II/R | 3.0 |  | 687 | RNA processing |
| DN11474_c0_g1_i1.g3791.t1 | hypothetical protein FOC4_g10002990 | Nitrogen regulatory protein areA, GATA-like domain | 1.8 |  | 584 | Regulation of nitrogen utilization |
| DN11480_c0_g1_i1.g3770.t1 | hypothetical protein BFJ69_g9930 | Major facilitator, sugar transporter-like | 3.3 |  | 557 | Transport |
| DN11506_c0_g1_i1.g203.t1 | hypothetical protein FOC1_g10009461 | Polyketide synthase, enoylreductase domain | 2.4 |  | 353 | Biosynthetic |
| DN11535_c0_g1_i1.g187.t1 | hypothetical protein FOTG_03887 | Nucleolar complex-associated protein 3, N-terminal | 1.4 |  | 629 | Ribosome biogenesis |
| DN11562_c0_g1_i1.g202.t1 | hypothetical protein FOIG_07053 |  | 4.4 | Nucleus | 149 |  |
| DN11577_c0_g1_i1.g184.t1 | E3 ubiquitin-protein ligase BRE1 | Zinc finger, RING-type | 1.1 |  | 702 | Transcription |
| DN11578_c0_g1_i1.g216.t1 | Dimethyladenosine transferase | Ribosomal RNA adenine methyltransferase KsgA/Erm | 2.1 |  | 298 | Methylation |
| DN11613_c0_g1_i1.g11378.t1 | Protein pyrABCN | Pre-ATP-grasp domain superfamily | 7.3 |  | 98 |  |
| DN11647_c0_g1_i1.g11377.t1 | Arginine permease | Amino acid permease/ SLC12A domain | 3.0 |  | 515 | Transport |
| DN11654_c0_g1_i1.g11401.t1 | Putative protein RSN1 | Calcium-dependent channel, 7TM region, putative phosphate | 1.7 |  | 853 | Transport |
| DN11662_c0_g1_i1.g11410.t1 | hypothetical protein BFJ68_g10103 | Zinc finger, CCCH-type | 2.5 |  | 476 | Transcription |
| DN11666_c0_g1_i1.g11367.t1 | citrate synthase, mitochondrial | Citrate synthase | 2.1 |  | 470 | Metabolic |
| DN11699_c0_g1_i1.g11393.t1 | hypothetical protein BFJ65_g18018 | U3 small nucleolar RNA-associated SSU processome protein 11 (Utp11) | 1.1 |  | 264 | rRNA processing |
| DN11770_c0_g1_i1.g7091.t1 | sulfite reductase (NADPH) flavoprotein alpha-component | Sulfite reductase [NADPH] flavoprotein alpha-component-like, FAD-binding | 3.6 |  | 1065 | Oxidation-reduction |
| DN11799_c0_g1_i1.g7097.t1 | aconitate hydratase, mitochondrial | Aconitase A/isopropylmalate dehydratase small subunit, swivel domain | 2.1 |  | 809 | Metabolic |
| DN11801_c0_g1_i1.g4935.t1 | hypothetical protein FOPG_02227 | Nucleolar protein 14 | 2.9 |  | 885 | Ribosome biogenesis |
| DN11837_c0_g1_i1.g4953.t1 | tRNA-dihydrouridine synthase 1 | tRNA-dihydrouridine synthase | 2.3 |  | 366 | Oxidation-reduction |
| DN11908_c0_g1_i1.g16742.t1 | hypothetical protein FOXG_01850 | P-loop containing nucleoside triphosphate hydrolase | 1.6 |  | 563 | Phosphorylation |
| DN11922_c0_g1_i1.g16747.t1 | Aldose reductase A | NADP-dependent oxidoreductase domain | 1.2 |  | 329 | Oxidation-reduction |
| DN1195_c0_g1_i1.g17770.t1 | hypothetical protein FOXG_07797 | Glycoside hydrolase, family 61 | 3.9 |  | 404 | Metabolic |
| DN11954_c0_g1_i1.g16715.t1 | UDP-glucose 6-dehydrogenase | UDP-glucose/GDP-mannose dehydrogenase, N-terminal | 2.3 |  | 605 | Oxidation-reduction |
| DN12007_c0_g1_i1.g11786.t1 | related to E. coli tetracycline resistance protein TCR1 |  | 4.8 |  | 133 |  |
| DN12069_c0_g1_i1.g11799.t1 | hypothetical protein FOTG_09758 | Major facilitator superfamily | 1.4 |  | 632 | Transport |
| DN1207_c0_g1_i1.g7472.t1 | hypothetical protein BFJ69_g4989 | Mitochondrial substrate/solute carrier | 2.1 |  | 336 | Transport |
| DN12141_c0_g1_i1.g13216.t1 | serine hydroxymethyltransferase, mitochondrial | Serine hydroxymethyltransferase-like domain | 1.6 |  | 495 | Biosynthetic |
| DN1215_c0_g1_i3.g7359.t1 | hypothetical protein FOC1_g10006701 | PAS fold 3 | 4.5 |  | 658 |  |
| DN12172_c0_g1_i1.g13194.t1 | Protein pyrABCN | Carbamoyl-phosphate synthetase large subunit-like, ATP-binding domain | 6.9 |  | 307 | Metabolic |
| DN12187_c0_g1_i1.g13208.t1 | hypothetical protein FOXG_01504 | Pyridine nucleotide-disulphide oxidoreductase | 3.8 |  | 367 | Oxidation-reduction |
| DN12288_c0_g1_i1.g5220.t1 | Putative glucose transporter rco-3 | Major facilitator, sugar transporter-like | 2.5 |  | 271 | Transport |
| DN12305_c0_g1_i1.g14896.t1 | hypothetical protein FOTG_04208 | Ribosomal protein L1 | 2.0 |  | 377 | Translation |
| DN1240_c0_g1_i4.g7162.t1 | ribose-phosphate pyrophosphokinase | Ribose-phosphate pyrophosphokinase | 2.7 |  | 503 | Biosynthetic |
| DN125_c0_g1_i2.g1529.t1 | uncharacterized protein FVRRES_05356 | Ribosomal protein S4e, central region | 1.2 |  | 263 | Translation |
| DN12593_c0_g1_i1.g17215.t1 | Developmental regulator flbA | Winged helix DNA-binding domain superfamily | 7.7 | Mitochondrion | 116 |  |
| DN12644_c0_g1_i1.g1311.t1 | elongator complex protein 1 | Elongator complex protein 1 | 3.6 |  | 266 | Uridine modification |
| DN12668_c0_g1_i1.g4246.t1 | 3-hydroxybutyryl-CoA dehydrogenase | 3-hydroxyacyl-CoA dehydrogenase, NAD binding | 6.3 |  | 223 | Oxidation-reduction |
| DN1270_c0_g1_i3.g7397.t1 | hypothetical protein FPSE_09944 | Ribosomal protein L14P | 1.8 |  | 139 | Translation |
| DN1290_c0_g1_i1.g7298.t1 | probable fibrillarin (NOP1) | Fibrillarin | 2.9 |  | 319 | rRNA processing |
| DN13_c0_g1_i1.g4410.t1 | Malate dehydrogenase, mitochondrial | Lactate/malate dehydrogenase, C-terminal | 1.9 |  | 336 | Oxidation-reduction |
| DN1300_c0_g1_i5.g14158.t1 | U3 small nucleolar RNA-associated protein 17 | WD domain, G-beta repeat | 2.2 |  | 463 | Signal transduction |
| DN1317_c0_g1_i6.g14505.t1 | hypothetical protein FOTG_04522 | Major facilitator superfamily | 1.3 |  | 497 | Transport |
| DN1326_c0_g1_i1.g14456.t1 | ATP-dependent RNA helicase DBP3 | P-loop containing nucleoside triphosphate hydrolase | 2.7 |  | 292 | Phosphorylation |
| DN1346_c0_g1_i1.g14407.t1 | allantoicase | Allantoicase domain | 1.7 |  | 360 | Catabolic |
| DN1348_c0_g1_i6.g45609.t1 | hypothetical protein FOTG_05894 | Nuclear pore protein 84/107 | 1.5 |  | 905 | Transcription |
| DN13515_c0_g1_i1.g5402.t1 | hypothetical protein FOC1_g10003552 | CoA-transferase family III | 3.9 |  | 576 | Catabolic |
| DN13523_c0_g1_i1.g5427.t1 | hypothetical protein FOTG_01519 | Mediator complex, subunit Med12 | 1.3 |  | 1519 | Transcription |
| DN13637_c0_g1_i1.g35173.t1 | hypothetical protein BFJ69_g21 | Major facilitator superfamily | 5.2 |  | 488 | Transport |
| DN13673_c0_g1_i1.g35218.t1 | Purine-cytosine permease FCY21 | Purine-cytosine permease | 3.6 |  | 307 | Transport |
| DN13679_c0_g1_i1.g311.t1 | hypothetical protein FOQG_02770 | CP2 transcription factor | 1.8 |  | 788 | Transcription |
| DN137_c0_g1_i2.g10073.t1 | hypothetical protein FOTG_00133 | Ribosomal RNA methyltransferase FtsJ domain | 4.8 |  | 835 | Methylation |
| DN1370_c0_g1_i2.g14575.t1 | hypothetical protein FOMG_02164 | Polyketide synthase, enoylreductase domain | 3.7 |  | 348 | Biosynthetic |
| DN1370_c1_g1_i2.g45526.t1 | hypothetical protein FOIG_07053 |  | 4.4 | Nucleus | 149 |  |
| DN13702_c0_g1_i1.g4207.t1 | hypothetical protein BFJ69_g6347 | Carboxylesterase, type B | 4.4 |  | 525 |  |
| DN13709_c0_g1_i1.g465.t1 | hypothetical protein FOXG_05017 |  | 4.1 |  | 304 |  |
| DN1371_c0_g1_i1.g14490.t1 | Fatty acid synthase subunit alpha | Fatty acid synthase type I, helical | 3.4 |  | 1855 | Oxidation-reduction |
| DN13716_c0_g1_i1.g460.t1 | Hybrid signal transduction protein dokA | GAF-like domain superfamily | 4.3 |  | 381 |  |
| DN13718_c0_g1_i1.g4208.t1 | hypothetical protein BFJ69_g4704 | NmrA-like domain | 5.3 |  | 299 |  |
| DN13775_c0_g1_i1.g395.t1 | Putative N-acetylglucosamine-6-phosphate deacetylase | N-acetylglucosamine-6-phosphate deacetylase | 2.7 |  | 444 | Metabolic |
| DN13791_c0_g1_i1.g419.t1 | allantoinase | Amidohydrolase-related | 3.1 |  | 527 | Proteolysis |
| DN1382_c0_g1_i16.g45825.t1 | hypothetical protein FOC1_g10006917 | NAD(P)H-binding | 7.6 |  | 308 |  |
| DN13826_c0_g1_i1.g9523.t1 | hypothetical protein FOXG_08441 |  | 1.4 |  | 285 |  |
| DN1383_c0_g1_i3.g14452.t1 | U3 small nucleolar RNA-associated protein 10 | U3 small nucleolar RNA-associated protein 10, N-terminal | 5.1 |  | 754 | rRNA processing |
| DN13832_c0_g1_i1.g9530.t1 | hypothetical protein FOPG_00077 | Pescadillo | 2.8 |  | 659 | Ribosome biogenesis |
| DN13854_c0_g1_i1.g9580.t1 | putative amino-acid permease | Amino acid permease/ SLC12A domain | 2.8 |  | 281 | Transport |
| DN13894_c0_g1_i1.g9576.t1 | hypothetical protein FOTG_07624 | SAM-dependent methyltransferase RsmB/NOP2-type | 2.6 |  | 679 | Methylation |
| DN13907_c0_g1_i1.g4271.t1 | chaperone hchA | Chaperone protein hchA | 7.1 |  | 243 | Stress response |
| DN13911_c0_g1_i1.g4262.t1 | hypothetical protein FOTG_07720 |  | 4.1 | Extracellular | 90 |  |
| DN13980_c0_g1_i1.g9484.t1 | Elongator complex protein 1 | Elongator complex protein 1 | 4.1 |  | 197 | Uridine modification |
| DN14012_c0_g1_i1.g7831.t1 | related to serine/threonine protein kinase | Protein kinase domain | 1.3 |  | 522 | Signal transduction |
| DN14032_c0_g1_i1.g7815.t1 | hypothetical protein FOMG_08604 | ATPase, AAA-type, core | 2.3 |  | 682 | Transport |
| DN1407_c0_g1_i1.g585.t1 | probable nuclear export sequence-containing nonribosomal protein | Nmd3, N-terminal | 1.8 |  | 517 |  |
| DN1411_c0_g1_i2.g17415.t1 | hypothetical protein FOTG_02910 | U3 small nucleolar ribonucleoprotein complex, subunit Mpp10 | 2.1 |  | 709 | rRNA processing |
| DN1423_c1_g1_i1.g597.t1 | ATP-dependent RNA helicase DBP9 | P-loop containing nucleoside triphosphate hydrolase | 1.5 |  | 613 | Phosphorylation |
| DN14231_c0_g1_i1.g2804.t1 | Monocarboxylate transporter 8 | Major facilitator, sugar transporter-like | 4.6 |  | 268 | Transport |
| DN14249_c0_g1_i1.g3572.t1 | Orotidine 5'-phosphate decarboxylase | Orotidine 5'-phosphate decarboxylase domain | 2.0 |  | 366 | Biosynthetic |
| DN14261_c0_g1_i1.g3596.t1 | probable PAB1-mRNA polyadenylate-binding protein | Hyperplastic disc protein | 2.4 |  | 750 |  |
| DN1429_c0_g1_i3.g17486.t1 | probable FPR3-prolyl cis-trans isomerase | FKBP-type peptidyl-prolyl cis-trans isomerase domain | 4.0 |  | 463 | Protein peptidyl-prolyl isomerization |
| DN1431_c0_g1_i2.g17390.t1 | enoyl reductase | 3-oxo-5-alpha-steroid 4-dehydrogenase, C-terminal | 4.6 |  | 295 | Metabolic |
| DN1432_c0_g1_i1.g17322.t1 | hypothetical protein BFJ69_g8180 | zinc finger | 6.8 |  | 668 | Transcription |
| DN14327_c0_g1_i1.g9751.t1 | methylenetetrahydrofolate dehydrogenase (NAD+) | Tetrahydrofolate dehydrogenase/cyclohydrolase, catalytic domain | 1.9 |  | 338 | Oxidation-reduction |
| DN14344_c0_g1_i1.g9728.t1 | hypothetical protein FVEG_11508 |  | 4.7 |  | 154 |  |
| DN1438_c0_g1_i2.g868.t1 | serine hydroxymethyltransferase, cytosolic | Serine hydroxymethyltransferase-like domain | 1.9 |  | 484 | Tetrahydrofolate interconversion |
| DN1443_c0_g1_i2.g805.t1 | phosphoribosylaminoimidazole carboxylase | Phosphoribosylaminoimidazole carboxylase, C-terminal domain | 2.0 |  | 586 | Biosynthetic |
| DN14503_c0_g1_i1.g14131.t1 | DNA-directed RNA polymerase I subunit RPA2 | DNA-directed RNA polymerase, subunit 2, hybrid-binding domain | 2.3 |  | 391 | Transcription |
| DN1451_c0_g1_i3.g647.t1 | heat shock 70kDa protein 4 | Heat shock protein 70 family | 2.6 |  | 706 | Stress response |
| DN1451_c1_g1_i1.g652.t1 | Eukaryotic translation initiation factor 5B | GTP-binding domain | 1.3 |  | 704 | Transcription |
| DN1452_c0_g1_i2.g771.t1 | Phospholipase D1 | Phospholipase D/Transphosphatidylase | 7.2 |  | 1548 | Signal transduction |
| DN1455_c0_g1_i3.g758.t1 | hypothetical protein FOTG_06615 | GYF domain | 1.5 |  | 1457 |  |
| DN1468_c0_g1_i6.g700.t1 | hypothetical protein FOXG_08613 | Zinc finger, GATA-type | 2.9 |  | 429 | Transcription |
| DN1470_c0_g1_i1.g579.t1 | MRS4-like protein | Mitochondrial substrate/solute carrier | 2.1 |  | 310 | Transport |
| DN14739_c0_g1_i1.g44771.t1 | Peroxisomal primary amine oxidase | Copper amine oxidase, catalytic domain | 6.3 | Cytoplasm | 275 | Oxidation-reduction |
| DN1474_c0_g1_i1.g627.t1 | Importin subunit beta 2 | HEAT-like repeat | 1.5 |  | 943 |  |
| DN1478_c0_g1_i2.g634.t1 | Centromere/microtubule-binding protein cbf5 | Pseudouridine synthase II, N-terminal | 3.1 |  | 487 | RNA modification |
| DN1479_c0_g1_i7.g889.t1 | NADH dehydrogenase | Pyridine nucleotide-disulphide oxidoreductase | 3.5 |  | 485 | Oxidation-reduction |
| DN1481_c0_g1_i1.g801.t1 | hypothetical protein FOXG_11073 | R3H domain | 2.4 |  | 624 |  |
| DN1487_c0_g1_i2.g828.t1 | hypothetical protein FOTG_05488 | Ankyrin repeats (many copies) | 1.5 |  | 1155 | Metabolic |
| DN1520_c0_g1_i1.g3364.t1 | ADP, ATP carrier protein | Mitochondrial substrate/solute carrier | 3.3 |  | 311 | Transport |
| DN1529_c0_g1_i4.g3388.t1 | amidophosphoribosyltransferase | Amidophosphoribosyltransferase | 3.0 |  | 551 | Biosynthetic |
| DN1530_c0_g1_i2.g3424.t1 | hypothetical protein FAVG1_12529 | P-loop containing nucleoside triphosphate hydrolase | 2.7 |  | 396 | Phosphorylation |
| DN15420_c0_g1_i1.g17502.t1 | Protein pyrABCN | Carbamoyl-phosphate synthetase, large subunit oligomerisation domain | 7.4 |  | 249 | Metabolic |
| DN155_c0_g1_i1.g1611.t1 | spermidine synthase | Spermidine synthase, tetramerisation domain | 1.5 |  | 293 | Metabolic |
| DN1556_c0_g1_i2.g3491.t1 | F-box and WD-40 domain-containing protein CDC4 | WD domain, G-beta repeat | 1.4 |  | 674 | Signal transduction |
| DN1559_c0_g1_i2.g3498.t1 | ATPase | P-type ATPase, subfamily V | 1.3 |  | 1293 | Transport |
| DN15676_c0_g1_i1.g14940.t1 | hypothetical protein FOXG_04914 | Protein of unknown function DUF829, TMEM53 | 2.2 |  | 284 |  |
| DN1568_c0_g1_i2.g3495.t1 | 60S ribosomal subunit assembly/export protein loc-1 |  | 1.5 |  | 195 |  |
| DN1581_c0_g1_i2.g3568.t1 | hypothetical protein FOTG_05682 | Uncharacterised protein family UPF0592 | 1.6 |  | 1216 |  |
| DN15850_c0_g1_i1.g16792.t1 | hypothetical protein FOC1_g10005942 | Protein of unknown function DUF2434 | 1.3 |  | 581 |  |
| DN15891_c0_g1_i1.g16758.t1 | hypothetical protein BFJ69_g13017 | Brix domain | 1.9 |  | 317 | Ribosomal large subunit assembly |
| DN15916_c0_g1_i1.g6884.t1 | hypothetical protein FOTG_01051 | Nucleolar 27S | 1.9 |  | 611 | Ribosome biogenesis |
| DN15931_c0_g1_i1.g6868.t1 | Putative CDP-alcohol phosphatidyltransferase class-I family protein C22A12.08c | Haloacid dehalogenase-like hydrolase | 1.3 |  | 494 |  |
| DN15940_c0_g1_i1.g6898.t1 | hypothetical protein BFJ69_g2823 |  | 4.0 |  | 381 |  |
| DN16042_c0_g1_i1.g6455.t1 | hypothetical protein FOXG_09391 | KRR1 interacting protein 1 | 1.2 |  | 647 |  |
| DN16055_c0_g1_i1.g6463.t1 | ATP-dependent RNA helicase DBP7 | P-loop containing nucleoside triphosphate hydrolase | 1.6 |  | 519 | Phosphorylation |
| DN1606_c0_g1_i3.g7933.t1 | Serine/threonine-protein kinase MST20 | Protein kinase domain | 2.9 |  | 851 | Signal transduction |
| DN1610_c0_g1_i1.g8158.t1 | Sulfite reductase | Nitrite/sulphite reductase 4Fe-4S domain | 2.6 |  | 1537 | Oxidation-reduction |
| DN16100_c0_g1_i1.g5334.t1 | hypothetical protein FOC1_g10005850 | Methyltransferase domain 25 | 1.4 |  | 544 | Methylation |
| DN16121_c0_g1_i1.g5348.t1 | Malate synthase, glyoxysomal | Malate synthase | 4.0 |  | 542 | Metabolic |
| DN1613_c0_g1_i1.g7345.t1 | hypothetical protein BFJ69_g4919 | Zn (2)-C6 fungal-type DNA-binding domain | 5.8 |  | 726 | Transcription |
| DN16179_c0_g1_i1.g5326.t1 | Protein VTS1 |  | 1.3 |  | 323 |  |
| DN16202_c0_g1_i1.g2567.t1 | related to quinate transport protein | Major facilitator, sugar transporter-like | 3.6 |  | 559 | Transport |
| DN16211_c0_g1_i1.g14969.t1 | hypothetical protein FOXG_08476 | Bystin | 1.6 |  | 479 | Ribosome biogenesis |
| DN1623_c0_g1_i1.g8015.t1 | hypothetical protein FOTG_07898 | Zn (2)-C6 fungal-type DNA-binding domain | 3.1 |  | 591 | Transcription |
| DN16251_c0_g1_i1.g14991.t1 | Bifunctional purine biosynthesis protein ADE17 | purine biosynthesis protein PurH-like | 2.1 |  | 595 | Biosynthetic |
| DN16261_c0_g1_i1.g14957.t1 | hypothetical protein FOTG_06087 | U3 small nucleolar RNA-associated SSU processome protein (Utp23) | 1.9 |  | 287 | rRNA processing |
| DN16273_c0_g1_i2.g2523.t1 | Sugar transporter STL1 | Major facilitator, sugar transporter-like | 3.8 |  | 546 | Transport |
| DN16275_c0_g1_i1.g14954.t1 | probable ubiquitin-conjugating enzyme CDC34 | Ubiquitin-conjugating enzyme E2 | 1.2 |  | 224 |  |
| DN16277_c0_g1_i1.g14975.t1 | glycine dehydrogenase | Glycine cleavage system P protein | 2.3 |  | 1052 | Oxidation-reduction |
| DN16286_c0_g1_i1.g14965.t1 | hypothetical protein FOCG_02355 | Zinc finger C2H2 superfamily | 1.3 |  | 276 | Transcription |
| DN16295_c0_g1_i1.g14955.t1 | Bifunctional protein RIB2 | Pseudouridine synthase, RsuA/RluA | 1.7 |  | 514 | RNA modification |
| DN16314_c0_g1_i1.g15320.t1 | hypothetical protein FOXG_12843 | NOP5, N-terminal | 3.3 |  | 593 | Ribosome biogenesis |
| DN16335_c0_g1_i1.g15298.t1 | Threonine synthase | Threonine synthase, N-terminal | 1.5 |  | 517 | Biosynthetic |
| DN16336_c0_g1_i1.g17179.t1 | hypothetical protein FOXG_13238 |  | 6.2 |  | 193 |  |
| DN1635_c0_g1_i1.g8002.t1 | Eukaryotic translation initiation factor 3 subunit B | Translation initiation factor, beta propellor-like domain | 1.4 |  | 747 | Translation |
| DN16383_c0_g1_i1.g17142.t1 | hypothetical protein FOXG_05893 | Major facilitator superfamily | 6.8 |  | 434 | Transport |
| DN16389_c0_g1_i1.g15321.t1 | betaine-aldehyde dehydrogenase | Aldehyde dehydrogenase domain | 1.5 |  | 509 | Oxidation-reduction |
| DN16407_c0_g1_i1.g5259.t1 | Aspartate aminotransferase, cytoplasmic | Aminotransferase, class I/classII | 2.1 |  | 414 | Biosynthetic |
| DN16443_c0_g1_i1.g3850.t1 | hypothetical protein BFJ69_g8254 | Nitroreductase | 3.2 | Mitochondrion | 205 | Stress response |
| DN16479_c0_g1_i1.g5253.t1 | hypothetical protein FOXG_11627 | Brix domain | 2.0 |  | 422 | Ribosomal large subunit assembly |
| DN16522_c0_g1_i2.g14898.t1 | hypothetical protein BFJ69_g3297 | Alcohol dehydrogenase, N-terminal | 3.1 |  | 321 | Oxidation-reduction |
| DN16593_c0_g1_i1.g14900.t1 | hypothetical protein FOC4_g10001384 | Letm1 ribosome-binding domain | 1.4 |  | 552 |  |
| DN16638_c0_g1_i1.g13031.t1 | Succinate/fumarate mitochondrial transporter | Mitochondrial substrate/solute carrier | 5.8 | Mitochondrion | 164 | Transport |
| DN1675_c0_g1_i2.g8139.t1 | Amino-acid transporter arg-13 | Mitochondrial substrate/solute carrier | 1.3 |  | 331 | Transport |
| DN1677_c0_g1_i8.g7099.t1 | hypothetical protein FOTG_00952 | U3 small nucleolar RNA-associated protein 10, N-terminal | 5.5 |  | 1807 | rRNA processing |
| DN16788_c0_g1_i1.g11863.t1 | rRNA biogenesis protein rrp5 |  | 6.1 |  | 96 |  |
| DN16801_c0_g1_i1.g3736.t1 | hypothetical protein FOTG_13072 | Oxidoreductase, molybdopterin-binding domain | 4.2 |  | 299 | Oxidation-reduction |
| DN16823_c0_g1_i1.g11258.t1 | Mitochondrial protein cyt-4 |  | 3.6 | Peroxisome | 212 |  |
| DN16880_c0_g1_i1.g11269.t1 | hypothetical protein FOTG_08114 | U3 small nucleolar RNA-associated SSU processome protein 11 (Utp12) | 1.7 |  | 391 | rRNA processing |
| DN16883_c0_g1_i1.g3717.t1 | aliphatic nitrilase | Carbon-nitrogen hydrolase | 7.2 |  | 362 | Metabolic |
| DN1691_c0_g1_i2.g8004.t1 | hypothetical protein FOXG_01669 | Mitochondrial substrate/solute carrier | 5.5 |  | 695 | Transport |
| DN16930_c0_g1_i1.g7068.t1 | Dol-P-Man:Man(5)GlcNAc(2)-PP-Dol alpha-1,3-mannosyltransferase | AhpD-like | 3.0 |  | 270 | Stress response |
| DN1707_c0_g1_i1.g48038.t1 | aminodeoxychorismate synthase | Glutamine amidotransferase | 2.7 |  | 830 | Metabolic |
| DN17093_c0_g1_i1.g21692.t1 | hypothetical protein BFJ69_g6466 | integral membrane protein | 3.0 |  | 243 |  |
| DN17095_c0_g1_i1.g21666.t1 | hypothetical protein FOTG_09559 | RTA-like protein | 3.8 |  | 288 |  |
| DN1718_c0_g1_i3.g11929.t1 | hypothetical protein FOMG_10877 | Polyketide synthase, enoylreductase domain | 3.9 |  | 333 | Biosynthetic |
| DN1724_c0_g1_i2.g11956.t1 | probable zuotin | Chaperone DnaJ-domain superfamily | 2.7 |  | 435 | Stress response |
| DN17291_c0_g1_i1.g35136.t1 | hypothetical protein FOXG_10949 |  | 7.7 | Extracellular | 109 |  |
| DN1735_c0_g1_i2.g12029.t1 | hypothetical protein FOC1_g10002574 |  | 8.4 |  | 360 |  |
| DN17444_c0_g1_i1.g49156.t1 | hypothetical protein BFJ72_g1866 |  | 9.0 | Extracellular | 139 |  |
| DN17536_c0_g1_i1.g5589.t1 | hypothetical protein FOPG_02419 |  | 8.1 | Extracellular | 122 |  |
| DN1757_c0_g1_i1.g48161.t1 | hypothetical protein FOXG_04143 | Polyketide synthase, enoylreductase domain | 3.1 |  | 331 | Biosynthetic |
| DN17619_c0_g1_i1.g35493.t1 | hypothetical protein BFJ65_g12585 |  | 6.4 |  | 237 |  |
| DN17641_c0_g1_i1.g5560.t1 | putative amino-acid permease | Amino acid permease/ SLC12A domain | 5.5 |  | 101 | Transport |
| DN17699_c0_g1_i1.g35513.t1 | hypothetical protein BFJ65_g6361 | Alcohol dehydrogenase, C-terminal | 4.0 | Cytoplasm | 212 | Oxidation-reduction |
| DN1790_c0_g1_i3.g11938.t1 | eukaryotic translation initiation factor 3 subunit I | WD domain, G-beta repeat | 1.6 |  | 337 | Signal transduction |
| DN1807_c0_g1_i1.g16541.t1 | Translation initiation factor IF-2, mitochondrial | GTP-binding domain | 7.9 |  | 906 | Transcription |
| DN18082_c0_g1_i1.g18682.t1 | hypothetical protein FOXG_12803 | Heat shock factor (HSF)-type, DNA-binding | 3.9 |  | 586 | Transcription |
| DN18087_c0_g1_i1.g18692.t1 | related to SRP40 Suppressor of mutant AC40 of RNA polymerase I and III | GTP-binding protein | 1.0 |  | 318 | Signal transduction |
| DN181_c1_g1_i1.g8130.t1 | Ribosome production factor 1 | Brix domain | 1.3 |  | 415 | Ribosomal large subunit assembly |
| DN18109_c0_g1_i1.g17912.t1 | Elongation factor G | Elongation Factor G, domain II (GTP binding) | 3.1 |  | 786 | Translation |
| DN1813_c0_g1_i2.g16464.t1 | related to translation initiation factor 3 (47 kDa subunit) | Eukaryotic translation initiation factor 3 subunit F | 1.3 |  | 358 | Translation |
| DN18184_c0_g1_i1.g17867.t1 | hydroxymethylglutaryl-CoA synthase | Hydroxymethylglutaryl-coenzyme A synthase C-terminal domain | 1.3 |  | 456 | Biosynthetic |
| DN18203_c0_g1_i1.g11665.t1 | hypothetical protein FOTG_06876 | Myb-like DNA-binding domain | 1.4 |  | 1062 |  |
| DN18238_c0_g1_i1.g11687.t1 | RNA cytidine acetyltransferase | tRNA (Met) cytidine acetyltransferase TmcA, N-terminal | 5.5 |  | 217 | tRNA methylation |
| DN18264_c0_g1_i1.g11644.t1 | hypothetical protein FOC1_g10011313 | Nitrogen regulatory protein areA, GATA-like domain | 1.8 |  | 562 | Regulation of nitrogen utilization |
| DN18268_c0_g1_i1.g11691.t1 | NAD-specific glutamate dehydrogenase | Glutamate/phenylalanine/leucine/valine dehydrogenase, C-terminal | 2.3 |  | 1068 | Oxidation-reduction |
| DN18273_c0_g1_i1.g11701.t1 | Eukaryotic translation initiation factor 3 subunit A | Proteasome component (PCI) domain | 1.1 |  | 1056 |  |
| DN1830_c0_g1_i2.g18570.t1 | hypothetical protein FOPG_07197 | Ribosome biogenesis protein BMS1/TSR1, C-terminal | 1.7 |  | 816 | Ribosome biogenesis |
| DN18308_c0_g1_i1.g17298.t1 | Calcium-transporting ATPase 2 | P-type ATPase, cytoplasmic domain N | 5.4 | Mitochondrion | 110 | Transport |
| DN18310_c0_g1_i1.g17341.t1 | ATP-dependent RNA helicase DBP4 | P-loop containing nucleoside triphosphate hydrolase | 2.1 |  | 782 | Phosphorylation |
| DN1832_c0_g1_i1.g16523.t1 | hypothetical protein FOTG_06214 | DNA-directed RNA polymerase I, subunit RPA34.5 | 3.2 |  | 653 | Transcription |
| DN1835_c0_g1_i1.g16636.t1 | omega-6 fatty acid desaturase (delta-12 desaturase) | Fatty acid desaturase domain | 5.0 |  | 379 | Metabolic |
| DN18411_c0_g1_i1.g10312.t1 | hypothetical protein BFJ71_g5962 | Enoyl- (Acyl carrier protein) reductase | 1.5 |  | 362 | Metabolic |
| DN1843_c0_g1_i1.g18387.t1 | hypothetical protein FOTG_01934 | Protein Sls1 | 2.9 |  | 1245 | Translation |
| DN18462_c0_g1_i1.g10355.t1 | ATP-dependent RNA helicase cyt-19, mitochondrial | P-loop containing nucleoside triphosphate hydrolase | 2.1 |  | 679 | Phosphorylation |
| DN1848_c0_g1_i1.g16329.t1 | hypothetical protein FOTG_08608 | DNA polymerase V/Myb-binding protein 1A | 1.7 |  | 627 | Transcription |
| DN18485_c0_g1_i1.g10335.t1 | ABC transporter CDR4 | ABC-2 type transporter | 1.1 |  | 1507 | Transport |
| DN18491_c0_g1_i1.g10313.t1 | hypothetical protein FOC1_g10010154 | Major facilitator superfamily | 2.1 |  | 614 | Transport |
| DN18573_c0_g1_i1.g2548.t1 | Protein SOK1 | T-complex 11 | 2.2 |  | 638 |  |
| DN18576_c0_g1_i1.g2550.t1 | hypothetical protein FOTG_07588 | P-loop containing nucleoside triphosphate hydrolase | 1.8 |  | 614 | Phosphorylation |
| DN18678_c0_g1_i1.g7763.t1 | Glucose/galactose transporter | Major Facilitator Superfamily of transporters (MFS_FucP_like) | 6.7 |  | 94 | Transport |
| DN18716_c0_g1_i1.g5740.t1 | nitric oxide dioxygenase | Oxidoreductase FAD/NAD(P)-binding | 3.7 |  | 255 | Oxidation-reduction |
| DN18744_c0_g1_i1.g5752.t1 | hypothetical protein BFJ71_g7699 | Transcription factor domain, fungi | 4.0 |  | 302 | Transcription |
| DN1877_c0_g1_i1.g16413.t1 | probable FBP1-fructose-1,6-bisphosphatase | Fructose-1-6-bisphosphatase class I, N-terminal | 3.5 |  | 274 | Metabolic |
| DN18789_c0_g1_i2.g5724.t1 | hypothetical protein BFJ69_g8554 |  | 1.6 |  | 157 |  |
| DN188_c0_g1_i2.g1462.t1 | Nucleolar protein 56 | NOP5, N-terminal | 3.0 |  | 511 | Ribosome biogenesis |
| DN1891_c0_g1_i2.g16527.t1 | 60S ribosomal protein L4-B | Ribosomal protein L4/L1e | 1.6 |  | 352 | Translation |
| DN19023_c0_g1_i1.g50956.t1 | hypothetical protein FOQG_09102 | Mitochondrial protein | 2.9 |  | 291 | Metabolic |
| DN19030_c0_g1_i1.g13767.t1 | hypothetical protein BFJ65_g1762 |  | 3.3 |  | 406 |  |
| DN19110_c0_g1_i1.g21604.t1 | hypothetical protein FOTG_08988 | Zn (2)-C6 fungal-type DNA-binding domain-GAL4 | 1.2 |  | 731 | Transcription |
| DN19112_c0_g1_i1.g3965.t1 | hypothetical protein FOC4_g10003626 |  | 5.7 | Mitochondrion | 135 |  |
| DN1919_c0_g1_i1.g11242.t1 | Phosphoribosylformylglycinamidine synthase | Glutamine amidotransferase domain | 1.7 |  | 509 | Metabolic |
| DN19198_c0_g1_i1.g21594.t1 | Maltose permease MAL31 | Major facilitator, sugar transporter-like | 5.7 |  | 157 | Transport |
| DN193_c0_g1_i1.g1489.t1 | Zinc finger protein rsv2 | Zinc finger C2H2-type | 5.2 |  | 602 | Transcription |
| DN193_c0_g1_i2.g1490.t1 | related to 26S proteasome subunit RPN4 | Zinc finger C2H2-type | 2.8 |  | 622 | Transcription |
| DN1930_c0_g1_i1.g11194.t1 | probable HUPF1 protein | P-loop containing nucleoside triphosphate hydrolase | 1.5 |  | 1083 | Phosphorylation |
| DN1931_c0_g1_i1.g11154.t1 | probable hnRNP arginine N-methyltransferase | Ribosomal protein L11 | 2.7 |  | 345 | Translation |
| DN1934_c0_g1_i1.g11244.t1 | hypothetical protein FOPG_06469 | Eukaryotic rRNA processing | 2.3 |  | 407 | rRNA processing |
| DN19408_c0_g1_i1.g2515.t1 | hypothetical protein BFJ69_g3924 | DSBA-like thioredoxin domain | 1.4 |  | 222 |  |
| DN19495_c0_g1_i1.g10394.t1 | Putative enoyl-CoA hydratase, mitochondrial | Enoyl-CoA hydratase/isomerase | 7.5 |  | 119 | Catabolic |
| DN19556_c0_g1_i1.g49438.t1 | hypothetical protein FOTG_12750 | FAD linked oxidase, N-terminal | 4.1 |  | 490 | Oxidation-reduction |
| DN19557_c0_g1_i1.g49473.t1 | Invertase 2 | Glycosyl hydrolase family 32, N-terminal | 6.0 |  | 433 | Metabolic |
| DN19610_c0_g1_i1.g14856.t1 | 1-aminocyclopropane-1-carboxylate synthase-like protein 1 | Pyridoxal phosphate-dependent transferase | 5.1 |  | 135 |  |
| DN1964_c0_g1_i2.g11001.t1 | translation initiation factor 4G | MIF4G-like, type 3 | 3.8 |  | 1393 |  |
| DN1965_c0_g1_i2.g11066.t1 | DNA-directed RNA polymerase, mitochondrial | DNA-directed RNA polymerase, N-terminal | 1.6 |  | 1232 | Transcription |
| DN197_c0_g1_i2.g8746.t1 | hypothetical protein FOTG_02327 | Phosphatidate cytidylyltransferase | 6.4 |  | 865 |  |
| DN19745_c0_g1_i1.g6441.t1 | hypothetical protein BFJ70_g13134 | Ankyrin repeat-containing domain | 4.8 | Extracellular | 143 | Metabolic |
| DN19839_c0_g1_i1.g18982.t1 | U3 small nucleolar RNA-associated protein 20 | Down-regulated-in-metastasis protein | 5.3 |  | 222 |  |
| DN199_c0_g1_i3.g1876.t1 | hypothetical protein FOTG_06621 | WD domain, G-beta repeat | 3.9 |  | 255 | Signal transduction |
| DN1995_c0_g1_i3.g41605.t1 | Threonine synthase | Threonine synthase, N-terminal | 1.6 |  | 539 | Biosynthetic |
| DN2002_c0_g1_i1.g20025.t1 | hypothetical protein FOXG_08912 | Amino acid permease/ SLC12A domain | 3.7 |  | 540 | Transport |
| DN20085_c0_g1_i1.g12287.t1 | hypothetical protein FOPG_10676 | Major facilitator, sugar transporter-like | 3.3 |  | 383 | Transport |
| DN20182_c0_g1_i1.g18675.t1 | Alcohol oxidase 1 | Glucose-methanol-choline oxidoreductase, C-terminal | 5.5 |  | 79 | Oxidation-reduction |
| DN20369_c0_g1_i1.g16667.t1 | hypothetical protein FOQG_01907 |  | 1.2 |  | 421 |  |
| DN20371_c0_g1_i1.g16668.t1 | probable holocytochrome-c synthase | Cytochrome c/c1 haem-lyase | 1.3 |  | 318 | Oxidation-reduction |
| DN2039_c0_g1_i2.g17098.t1 | Mitochondrial import inner membrane translocase subunit tim54 | Tim54 | 1.4 |  | 459 | Transport |
| DN20417_c0_g1_i1.g3153.t1 | hypothetical protein FOTG_04178 | PAS domain | 2.0 |  | 663 |  |
| DN20448_c0_g1_i1.g3136.t1 | Ribosome biogenesis protein NOC1 |  | 4.2 |  | 156 |  |
| DN20463_c0_g1_i1.g20363.t1 | hypothetical protein BFJ69_g5419 |  | 3.1 |  | 273 |  |
| DN20495_c0_g1_i2.g3098.t1 | ribosome biogenesis protein ERB1 | WD domain, G-beta repeat | 3.5 |  | 753 | Signal transduction |
| DN205_c0_g1_i2.g19274.t1 | hypothetical protein FOTG_02943 | Ran binding domain | 1.3 |  | 1201 | Transport |
| DN20535_c0_g1_i1.g1080.t1 | hypothetical protein FOTG_00538 | Armadillo-type fold | 1.8 |  | 759 |  |
| DN20547_c0_g1_i1.g1037.t1 | Putative tartrate dehydrogenase/decarboxylase ttuC | Isopropylmalate dehydrogenase-like domain | 1.8 |  | 369 | Oxidation-reduction |
| DN20554_c0_g1_i1.g1052.t1 | probable S-adenosylmethionine decarboxylase (spe-2) | S-adenosylmethionine decarboxylase | 1.7 |  | 494 | Biosynthetic |
| DN206_c0_g1_i3.g19739.t1 | delta-aminolevulinic acid dehydratase | Delta-aminolevulinic acid dehydratase | 2.5 |  | 386 | Biosynthetic |
| DN20630_c0_g1_i1.g9653.t1 | Aconitate hydratase | Aconitase A/isopropylmalate dehydratase small subunit, swivel domain | 2.6 |  | 788 | Metabolic |
| DN20635_c0_g1_i2.g9618.t1 | hypothetical protein BFJ69_g15185 | Anthranilate synthase component I, N-terminal | 3.6 |  | 129 | Biosynthetic |
| DN20640_c0_g1_i1.g16772.t1 | hypothetical protein BFJ68_g12772 | Conserved hypothetical protein | 4.0 |  | 163 |  |
| DN20694_c0_g1_i1.g9659.t1 | hypothetical protein BFJ65_g12668 |  | 5.0 |  | 90 |  |
| DN20706_c0_g1_i1.g14640.t1 | hypothetical protein BFJ69_g101 | NADH:flavin oxidoreductase/NADH oxidase, N-terminal | 5.7 |  | 323 | Oxidation-reduction |
| DN2071_c0_g1_i2.g20113.t1 | Ca2+:H+ antiporter | Sodium/calcium exchanger membrane region | 8.1 |  | 455 | Transport |
| DN20730_c0_g1_i2.g14667.t1 | 40s ribosomal protein s3 | Ribosomal protein S3, C-terminal | 1.5 |  | 288 | Translation |
| DN2074_c0_g1_i1.g16922.t1 | probable CPC2 protein | WD domain, G-beta repeat | 1.6 |  | 316 | Signal transduction |
| DN2078_c0_g1_i1.g17006.t1 | acetyl-coenzyme A synthetase | AMP-dependent synthetase/ligase | 4.0 |  | 721 |  |
| DN20781_c0_g1_i1.g14644.t1 | Calcium-transporting ATPase 2 | Haloacid dehalogenase-like hydrolase | 5.6 | Cytoplasm | 166 |  |
| DN208_c0_g1_i1.g19204.t1 | hypothetical protein FOQG_00379 |  | 2.1 |  | 621 |  |
| DN20813_c0_g1_i1.g15595.t1 | hypothetical protein FOTG_02856 | U3 small nucleolar RNA-associated protein 15, C-terminal | 2.7 |  | 532 | rRNA processing |
| DN20881_c0_g1_i1.g15619.t1 | hypothetical protein FOTG_04573 | Nucleolar complex protein 2 | 2.5 |  | 777 | Ribosome biogenesis |
| DN2089_c0_g1_i1.g17109.t1 | hypothetical protein BFJ69_g9225 | F-box domain | 3.8 |  | 562 |  |
| DN2090_c0_g1_i1.g16870.t1 | probable GMP synthase | GMP synthase, C-terminal | 3.4 |  | 544 | Biosynthetic |
| DN2097_c0_g1_i11.g19443.t1 | hypothetical protein FOIG_07922 | Guanine nucleotide exchange factor (Ras-like) | 1.9 |  | 1209 |  |
| DN2098_c0_g1_i5.g19938.t1 | hypothetical protein BFJ69_g15007 | Leucine-rich repeat domain superfamily | 1.8 |  | 1168 |  |
| DN21_c0_g2_i1.g4571.t1 | RNA cytidine acetyltransferase | Acetyltransferase (GNAT) domain | 2.3 |  | 351 | Biosynthetic |
| DN21010_c0_g1_i1.g96.t1 | hypothetical protein FOXG_13912 | Brix domain | 1.5 |  | 307 | Ribosomal large subunit assembly |
| DN2102_c0_g1_i1.g26708.t1 | hypothetical protein FOPG_05882 | La-type HTH domain | 3.9 |  | 775 |  |
| DN21024_c0_g1_i1.g91.t1 | ABC transporter G family member 14 | ABC transporter-like | 4.4 |  | 332 | Transport |
| DN2103_c0_g1_i1.g6211.t1 | hypothetical protein BFJ69_g14025 | Zinc finger C2H2 superfamily | 2.9 |  | 620 | Transcription |
| DN2109_c0_g1_i1.g26399.t1 | hypothetical protein FOC1_g10007236 | RNA recognition motif domain | 2.2 |  | 369 | Cytokinesis |
| DN21092_c0_g1_i1.g104.t1 | hypothetical protein FOXG_11113 | Mitochondrial substrate/solute carrier | 3.9 |  | 321 | Transport |
| DN21396_c0_g1_i1.g18792.t1 | ribosome biogenesis protein n | Ribosomal protein S8e | 2.1 |  | 264 | Translation |
| DN214_c0_g1_i2.g19754.t1 | eukaryotic translation initiation factor 3 subunit D | Eukaryotic translation initiation factor 3 subunit D | 1.8 |  | 576 | Translation |
| DN21408_c0_g1_i1.g381.t1 | Invertase 2 | Glycosyl hydrolase family 32, C-terminal | 4.7 |  | 121 | Metabolic |
| DN21429_c0_g1_i1.g387.t1 | 3-ketoacyl-CoA thiolase B, peroxisomal | acetyl-coenzyme A acetyltransferases (Thiolases) | 1.6 |  | 416 |  |
| DN21461_c0_g1_i1.g393.t1 | isocitrate lyase | Isocitrate lyase | 9.2 |  | 131 | Metabolic |
| DN21682_c0_g1_i1.g10236.t1 | Homocitrate synthase, mitochondrial | Pyruvate carboxyltransferase | 4.1 |  | 119 | Metabolic |
| DN2170_c0_g1_i1.g6287.t1 | ribosomal protein L28e | Ribosomal L28e/Mak16 | 1.4 | Nucleus | 159 | Translation |
| DN2172_c0_g1_i2.g26788.t1 | putative potassium transporter TRK-1 | Cation transporter | 1.7 |  | 973 | Transport |
| DN2176_c0_g1_i1.g6333.t1 | ribosome biogenesis protein ytm-1 | WD domain, G-beta repeat | 1.6 |  | 475 | Signal transduction |
| DN21931_c0_g1_i1.g4920.t1 | hypothetical protein BFJ72_g7039 | Annexin repeat | 5.3 |  | 185 |  |
| DN2196_c0_g1_i2.g6379.t1 | hypothetical protein BFJ69_g6102 | Tetratricopeptide repeat | 3.3 |  | 592 | RNA processing |
| DN2197_c0_g1_i2.g26455.t1 | hypothetical protein FOMG_10310 |  | 4.9 |  | 191 |  |
| DN2199_c0_g1_i1.g6339.t1 | hypothetical protein FOC1_g10012848 | Zn (2)-C6 fungal-type DNA-binding domain | 3.9 |  | 1149 | Transcription |
| DN22_c0_g1_i2.g4242.t1 | hypothetical protein BFJ65_g4001 | La-type HTH domain | 3.0 |  | 398 | RNA processing |
| DN2226_c0_g1_i2.g26126.t1 | MEAB protein | bZIP_YAP | 1.7 |  | 410 | Transcription |
| DN2230_c0_g1_i2.g25695.t1 | Developmental regulator flbA | RGS domain, Regulator of G protein signaling domain | 6.3 |  | 479 | Signal transduction |
| DN2237_c0_g1_i1.g12794.t1 | DnaJ like subfamily A member 2 | Chaperone DnaJ-domain superfamily | 3.3 |  | 434 | Stress response |
| DN2247_c0_g1_i2.g12650.t1 | amino-acid permease inda1 | Amino acid permease/ SLC12A domain | 1.4 |  | 559 | Transport |
| DN22555_c0_g1_i1.g12507.t1 | hypothetical protein FPSE_05918 | Methylglyoxal synthase-like domain | 5.7 | Extracellular | 80 |  |
| DN2261_c0_g1_i3.g12641.t1 | hypothetical protein FOTG_03461 |  | 4.1 |  | 260 |  |
| DN2270_c0_g1_i3.g12584.t1 | hypothetical protein FOTG_05904 | CCAAT-binding factor | 2.5 |  | 859 |  |
| DN228_c0_g1_i52.g1659.t1 | hypothetical protein FOTG_01343 | U3 small nucleolar RNA-associated protein 14 (Utp14 protein) | 3.7 |  | 671 | rRNA processing |
| DN2300_c0_g1_i1.g12323.t1 | Putative helicase C6F12.16c | P-loop containing nucleoside triphosphate hydrolase | 2.4 |  | 647 | Phosphorylation |
| DN2304_c0_g1_i3.g40859.t1 | Putative hexaprenyl pyrophosphate synthase, mitochondrial | Polyprenyl synthetase | 1.2 |  | 455 | Biosynthetic |
| DN231_c0_g1_i10.g2281.t1 | NIK-1 nonidentical kinase-1 | Protein kinase domain-histidine kinase, dimerisation/phosphoacceptor domain | 4.8 |  | 1272 | Signal transduction |
| DN2310_c0_g1_i3.g12198.t1 | hypothetical protein FOXG_00760 | Mitochondrial substrate/solute carrier | 1.7 |  | 346 | Transport |
| DN2325_c0_g1_i1.g12356.t1 | mitochondrial inner membrane magnesium transporter mrs2 | Magnesium transporter MRS2-like | 1.5 |  | 485 | Transport |
| DN2330_c2_g1_i1.g12195.t1 | Calcium-transporting ATPase 2 | Cation-transporting P-type ATPase, C-terminal | 4.1 |  | 112 | Transport |
| DN2335_c0_g1_i1.g12148.t1 | hypothetical protein FOC1_g10013863 |  | 2.7 |  | 349 |  |
| DN2336_c0_g1_i1.g12174.t1 | hypothetical protein FOXG_00851 | RNA recognition motif domain | 3.0 |  | 854 | Cytokinesis |
| DN234_c0_g1_i1.g19346.t1 | hypothetical protein FOMG_08719 | JAB1/MPN/MOV34 metalloenzyme domain | 7.0 |  | 463 |  |
| DN2359_c0_g1_i1.g12340.t1 | probable formate dehydrogenase | D-isomer specific 2-hydroxyacid dehydrogenase, NAD-binding domain | 1.7 |  | 365 | Oxidation-reduction |
| DN2365_c0_g1_i5.g40631.t1 | histone-lysine N-methyltransferase, H3 lysine-4 specific | Histone lysine methyltransferase SET associated | 1.7 |  | 1258 | Transcription |
| DN2368_c0_g1_i1.g12353.t1 | Adenine phosphoribosyltransferase | Phosphoribosyltransferase domain | 1.5 |  | 222 | Metabolic |
| DN2371_c0_g1_i2.g12248.t1 | Putative pre-mRNA-splicing factor ATP-dependent RNA helicase prp43 | P-loop containing nucleoside triphosphate hydrolase | 3.8 |  | 555 | Phosphorylation |
| DN2372_c0_g1_i1.g12369.t1 | Putative metal ion transporter C27B12.12c | CorA-like Mg2+ transporter protein | 1.6 |  | 623 | Transport |
| DN2435_c0_g1_i1.g13977.t1 | hypothetical protein FOTG_09867 | Tim44-like domain | 2.5 |  | 553 | Transport |
| DN2436_c0_g1_i1.g10994.t1 | hypothetical protein FOTG_09283 | ML-like domain | 1.8 |  | 934 | Transport |
| DN2439_c1_g1_i1.g11014.t1 | hypothetical protein FOC4_g10009109 | Haemerythrin-like | 2.2 |  | 188 |  |
| DN2440_c0_g1_i3.g11200.t1 | probable tryptophan synthase | Tryptophan synthase, alpha chain | 1.4 |  | 717 | Metabolic |
| DN2449_c0_g1_i1.g14033.t1 | hypothetical protein FOIG_06037 | Tetratricopeptide repeat | 4.4 |  | 658 | RNA processing |
| DN2451_c0_g1_i1.g13825.t1 | Putative WD repeat-containing protein C17D11.16 | WD domain, G-beta repeat | 3.4 |  | 529 | Signal transduction |
| DN2459_c0_g1_i1.g13851.t1 | Putative tRNA pseudouridine synthase 4 | Pseudouridine synthase II, N-terminal | 1.8 |  | 490 | RNA modification |
| DN2481_c0_g1_i1.g11058.t1 | aspartyl-tRNA synthetase | Aminoacyl-tRNA synthetase, class II (D/K/N) | 1.6 |  | 967 | tRNA aminoacylation |
| DN2490_c0_g1_i10.g10947.t1 | hypothetical protein FOTG_06446 | Cation transporter | 6.6 |  | 694 | Transport |
| DN2498_c0_g1_i1.g13874.t1 | homoaconitase, mitochondrial | Aconitase/3-isopropylmalate dehydratase large subunit, alpha/beta/alpha domain | 1.0 |  | 772 | Metabolic |
| DN2500_c0_g1_i4.g2892.t1 | RNA 3'-terminal phosphate cyclase | RNA 3'-terminal phosphate cyclase domain | 3.6 |  | 405 | Ribosome biogenesis |
| DN2545_c0_g1_i2.g4650.t1 | Malate dehydrogenase, mitochondrial | Lactate/malate dehydrogenase, N-terminal | 2.9 | Mitochondrion | 162 | Oxidation-reduction |
| DN261_c0_g1_i3.g19314.t1 | arginyl-tRNA synthetase | Arginyl-tRNA synthetase, catalytic core domain | 1.3 |  | 635 | Arginyl-tRNA aminoacylation |
| DN2618_c0_g1_i1.g6065.t1 | hypothetical protein FOTG_00705 | FYVE zinc finger | 1.7 |  | 287 | Transcription |
| DN2637_c0_g1_i1.g5960.t1 | Lipoyl synthase, mitochondrial | Lipoyl synthase, N-terminal | 1.0 |  | 411 | Biosynthetic |
| DN2645_c0_g1_i1.g3572.t1 | hypothetical protein FOTG_10398 | rna polymerase I subunit | 1.2 |  | 426 | Transcription |
| DN2645_c0_g1_i1.g5989.t1 | mitochondrial import receptor subunit tom-40 | Eukaryotic porin/Tom40 | 1.6 |  | 356 | Transport |
| DN2646_c0_g1_i1.g3008.t1 | hypothetical protein FOC1_g10013696 | von Willebrand factor, type A | 2.8 |  | 1134 |  |
| DN2658_c0_g1_i2.g6048.t1 | hypothetical protein FPSE_02953 | Ribosomal protein S7 domain | 1.1 | Cytoplasm | 217 | Translation |
| DN2675_c0_g1_i1.g6109.t1 | hypothetical protein FOTG_09210 | Zn (2)-C6 fungal-type DNA-binding domain | 2.3 |  | 468 | Transcription |
| DN2677_c0_g1_i1.g6022.t1 | hypothetical protein FOXG_10535 | Phosphoribosylglycinamide synthetase, ATP-grasp (A) domain | 2.9 |  | 797 | Biosynthetic |
| DN2688_c0_g1_i1.g5996.t1 | hypothetical protein BFJ69_g5697 | Protein kinase domain | 1.8 |  | 1245 | Signal transduction |
| DN27_c0_g1_i2.g4659.t1 | 40S ribosomal protein S7 | Ribosomal protein S7e | 1.8 |  | 203 | Translation |
| DN2721_c0_g1_i2.g15133.t1 | 1,4-alpha-glucan-branching enzyme | Glycoside hydrolase, family 13, N-terminal | 2.8 |  | 707 | Metabolic |
| DN2751_c0_g1_i2.g15055.t1 | uncharacterized protein FFUJ_04429 | Zinc finger, FYVE/PHD-type | 2.3 |  | 347 | Transcription |
| DN2758_c0_g1_i3.g48994.t1 | hypothetical protein FOXG_05440 | U3 small nucleolar RNA-associated SSU processome protein (Utp25 protein) | 1.9 |  | 719 | rRNA processing |
| DN2760_c0_g1_i1.g15178.t1 | hypothetical protein FOTG_04238 |  | 4.4 |  | 367 |  |
| DN2762_c0_g1_i2.g15210.t1 | probable TIM17-mitochondrial inner membrane import translocase subunit | Tim17/Tim22/Tim23/Pmp24 family | 1.7 |  | 152 | Transport |
| DN277_c0_g1_i1.g19706.t1 | hypothetical protein FOTG_04859 | Transcription factor domain, fungi | 2.1 |  | 507 | Transcription |
| DN277_c0_g1_i1.g19707.t1 | hypothetical protein BFJ72_g1037 | Zinc finger C2H2 superfamily | 4.6 |  | 429 | Transcription |
| DN2770_c0_g1_i1.g15105.t1 | fumarate hydratase, mitochondrial | Fumarase C, C-terminal | 2.2 |  | 529 | Metabolic |
| DN2774_c0_g1_i1.g15106.t1 | hypothetical protein FOC4_g10013939 | F-box-like domain superfamily | 2.5 |  | 306 | Transcription |
| DN279_c0_g1_i7.g19708.t1 | 60S ribosomal protein L35 | Ribosomal protein L29/L35 | 1.3 |  | 124 | Translation |
| DN279_c0_g1_i7.g19710.t1 | Mitochondrial ATPase complex subunit ATP10 | ATPase assembly factor ATP10 | 1.8 |  | 345 | Transport |
| DN2793_c0_g1_i2.g15202.t1 | Isocitrate lyase | Isocitrate lyase | 8.6 |  | 414 | Metabolic |
| DN2844_c0_g1_i1.g15482.t1 | hypothetical protein BFJ69_g2310 | guanine-nucleotide exchange factors catalytic domain (Ras) | 2.2 |  | 1238 | Signal transduction |
| DN2849_c0_g1_i1.g15535.t1 | Adenylosuccinate lyase | Adenylosuccinate lyase C-terminal | 3.2 |  | 481 | Biosynthetic |
| DN2855_c0_g1_i2.g15483.t1 | probable translation elongation factor eEF-3 | ABC transporter-like | 4.6 |  | 1055 | Transport |
| DN2871_c0_g1_i2.g15416.t1 | hypothetical protein FOPG_03657 | Glycosyltransferase family 1, N-terminal domain | 8.2 |  | 882 | Lipid glycosylation |
| DN2874_c0_g1_i1.g15520.t1 | tRNA pseudouridine synthase 1 | Pseudouridine synthase I, TruA, alpha/beta domain | 2.6 |  | 604 | RNA modification |
| DN288_c0_g1_i1.g19479.t1 | hypothetical protein FOPG_06852 | Protein of unknown function DUF3425 | 3.1 |  | 513 |  |
| DN288_c0_g1_i4.g19482.t1 | hypothetical protein BFJ65_g6222 | Protein of unknown function DUF3425 | 5.2 |  | 409 |  |
| DN2885_c0_g1_i1.g15478.t1 | Putative MFS-type transporter C16A3.17c | Major facilitator superfamily | 2.0 |  | 474 | Transport |
| DN2889_c0_g1_i1.g15494.t1 | BUD22 family protein C4F10.06 | Bud22 domain | 3.2 |  | 446 | Ribosome biogenesis |
| DN2890_c0_g1_i1.g38273.t1 | hypothetical protein BFJ72_g2866 | Zinc finger C2H2 superfamily | 1.8 |  | 616 | Transcription |
| DN2922_c0_g1_i1.g18489.t1 | hypothetical protein FOXG_10013 |  | 1.3 |  | 363 |  |
| DN2943_c0_g1_i2.g18422.t1 | hypothetical protein FOTG_04086 | Cyclin PHO80-like | 1.2 |  | 482 | Signal transduction |
| DN2947_c0_g1_i2.g18540.t1 | serine/threonine protein kinase | Protein kinase domain | 6.1 |  | 1198 | Signal transduction |
| DN2959_c0_g1_i2.g11861.t1 | hypothetical protein FAVG1_09906 |  | 2.7 | Extracellular | 81 |  |
| DN2974_c0_g1_i2.g18543.t1 | 3-isopropylmalate dehydratase | Aconitase/3-isopropylmalate dehydratase large subunit, alpha/beta/alpha domain | 1.9 |  | 777 | Metabolic |
| DN2982_c0_g1_i2.g11935.t1 | hypothetical protein FOTG_06235 | Homeobox-like domain superfamily | 6.3 |  | 1394 |  |
| DN3009_c0_g1_i2.g5701.t1 | hypothetical protein FOIG_08154 | Component of IIS longevity pathway SMK-1 | 1.8 |  | 880 | Metabolic |
| DN3022_c0_g1_i1.g1148.t1 | hypothetical protein FOC1_g10010277 | Mitochondrial substrate/solute carrier | 2.1 |  | 402 | Transport |
| DN3026_c0_g1_i3.g1193.t1 | Activator of stress proteins 1 | Zn (2)-C6 fungal-type DNA-binding domain | 2.6 |  | 960 | Transcription |
| DN3045_c0_g1_i3.g1207.t1 | Putative mitochondrial carnitine O-acetyltransferase | Choline/carnitine acyltransferase domain | 4.7 |  | 859 | Proteolysis |
| DN3050_c0_g1_i2.g1227.t1 | probable isocitrate dehydrogenase | Isopropylmalate dehydrogenase-like domain | 2.5 |  | 378 | Oxidation-reduction |
| DN3057_c0_g1_i1.g5711.t1 | Succinate/fumarate mitochondrial transporter | Mitochondrial substrate/solute carrier | 6.6 |  | 323 | Transport |
| DN3063_c0_g1_i1.g5952.t1 | Cystathionine beta-synthase | Pyridoxal-phosphate dependent enzyme | 1.8 |  | 517 |  |
| DN3086_c0_g1_i1.g1208.t1 | hypothetical protein FOXG_08482 | RGS domain, regulator of G protein | 3.0 |  | 722 | Signal transduction |
| DN3088_c0_g1_i1.g1260.t1 | hypothetical protein FOTG_09300 | Ketopantoate reductase, C-terminal domain | 1.2 |  | 465 | Oxidation-reduction |
| DN3097_c0_g1_i2.g5680.t1 | hypothetical protein FOTG_04049 |  | 3.8 |  | 197 |  |
| DN3139_c0_g1_i1.g6702.t1 | cytochrome c | Cytochrome c-like domain | 2.6 |  | 106 | Oxidation-reduction |
| DN3159_c0_g1_i1.g6794.t1 | AdoMet-dependent rRNA methyltransferase SPB1 | Ribosomal RNA methyltransferase FtsJ domain | 5.5 | Nucleus | 260 | Methylation |
| DN3169_c0_g1_i2.g6723.t1 | hypothetical protein FOTG_02190 | Clr5 domain | 2.1 |  | 887 |  |
| DN3184_c0_g1_i4.g6669.t1 | Required for respiratory growth protein 9, mitochondrial | Neugrin/Rrg9 | 1.3 |  | 344 |  |
| DN319_c0_g1_i19.g15736.t1 | 50s ribosomal l6e | 60S ribosomal protein L6E | 1.6 |  | 200 | Translation |
| DN32_c0_g1_i1.g4521.t1 | hypothetical protein FOTG_12342 |  | 3.2 |  | 602 |  |
| DN321_c0_g1_i2.g16317.t1 | putative ribosomal protein L9.e.c14 | Ribosomal protein L6, alpha-beta domain | 1.2 |  | 193 | Translation |
| DN3210_c0_g1_i1.g5401.t1 | related to hexamer-binding protein HEXBP | Zinc finger, CCHC-type | 4.2 | Cytoplasm | 223 | Transcription |
| DN3219_c0_g1_i1.g50367.t1 | hypothetical protein FOXG_07797 | Glycoside hydrolase, family 61 | 3.9 |  | 404 | Metabolic |
| DN322_c0_g1_i1.g16262.t1 | U3 small nucleolar RNA-associated protein 6 | U3 small nucleolar RNA-associated protein 6 | 1.6 |  | 392 | rRNA processing |
| DN3260_c0_g1_i1.g5531.t1 | Protein mak16 | Ribosomal L28e/Mak16 | 1.1 |  | 312 | Translation |
| DN3268_c0_g1_i1.g5472.t1 | Putative NADH-ubiquinone oxidoreductase C3A11.07, mitochondrial | Pyridine nucleotide-disulphide oxidoreductase | 4.8 |  | 577 | Oxidation-reduction |
| DN3271_c0_g1_i1.g50347.t1 | hypothetical protein FOTG_06965 | ATPase, AAA-type, core | 2.4 |  | 758 | Transport |
| DN3302_c0_g1_i1.g8338.t1 | hypothetical protein FOIG_08082 | Major facilitator superfamily | 1.5 |  | 566 | Transport |
| DN3314_c0_g1_i1.g43014.t1 | hypothetical protein FOXG_00305 | Chaperone DnaJ-domain superfamily | 6.9 |  | 222 | Stress response |
| DN3319_c0_g1_i1.g8268.t1 | aarF domain-containing kinase | UbiB domain | 2.1 |  | 573 |  |
| DN3329_c0_g1_i2.g8249.t1 | hypothetical protein BFJ65_g4243 | CFEM domain | 2.2 |  | 314 |  |
| DN3342_c0_g1_i3.g8262.t1 | Multisite-specific tRNA:(cytosine-C(5))-methyltransferase |  | 5.4 | Nucleus | 149 |  |
| DN3364_c0_g1_i1.g8319.t1 | probable 60S ribosomal protein L5 | Ribosomal protein L5 eukaryotic/L18 archaeal | 1.4 |  | 302 | Translation |
| DN3387_c0_g1_i1.g8311.t1 | hypothetical protein FOTG_03037 | Transcription factor Aft1, HRR domain | 2.2 |  | 526 | Transcription |
| DN3396_c0_g1_i1.g8241.t1 | hypothetical protein FOXG_11256 | Telomere repeat-binding factor, dimerisation domain | 1.7 |  | 846 |  |
| DN3418_c0_g1_i1.g18801.t1 | U3 small nucleolar RNA-associated protein 10 | BP28, C-terminal domain | 3.5 |  | 917 |  |
| DN3419_c0_g1_i1.g18922.t1 | hypothetical protein FOMG_14709 | Glucose receptor Git3, N-terminal-G protein-coupled | 4.0 |  | 407 | Signal transduction |
| DN3428_c0_g1_i2.g6349.t1 | Protein PXR1 | G-patch domain | 1.4 |  | 361 |  |
| DN3429_c0_g1_i1.g18874.t1 | Glutamate--cysteine ligase | Glutamate-cysteine ligase catalytic subunit | 3.0 |  | 716 | Biosynthetic |
| DN3453_c0_g1_i1.g18923.t1 | Fatty acid synthase subunit beta | Fatty acid synthase subunit beta, N-terminal domain | 3.6 |  | 2104 | Metabolic |
| DN3486_c0_g1_i2.g18939.t1 | Pre-rRNA-processing protein esf1 | NUC153 | 1.8 |  | 642 | rRNA processing |
| DN3493_c0_g1_i1.g18832.t1 | hypothetical protein FOPG_05207 | Alpha/beta hydrolase fold 1 | 1.2 |  | 475 | Metabolic |
| DN3505_c0_g1_i1.g4091.t1 | hypothetical protein FOC4_g10015188 | Protein of unknown function DUF3712 | 5.1 |  | 344 |  |
| DN3591_c0_g1_i1.g4156.t1 | DNA-directed RNA polymerase III subunit rpc-3 | RNA polymerase III Rpc82, C -terminal | 2.0 |  | 633 | Transcription |
| DN3594_c0_g1_i3.g4052.t1 | hypothetical protein BFJ69_g11453 | Calcium-dependent channel, 7TM region, putative phosphate | 1.2 |  | 1046 | Transport |
| DN3600_c0_g1_i1.g49894.t1 | chromatin modification-like protein VID21 | Helicase/SANT-associated domain | 1.5 |  | 1600 | Chromatin organization |
| DN3601_c0_g1_i1.g5052.t1 | probable TIM23 translocase | Tim17/Tim22/Tim23/Pmp24 family | 1.4 | Mitochondrion | 239 | Transport |
| DN3602_c0_g1_i1.g49679.t1 | elongation factor G, mitochondrial | GTP-binding domain | 3.1 |  | 825 | Transcription |
| DN3623_c0_g1_i1.g5084.t1 | hypothetical protein FOC4_g10011984 | Peptidase S1, PA clan | 1.8 |  | 1012 | Proteolysis |
| DN3625_c0_g1_i2.g49718.t1 | Choline transport protein | Amino acid/polyamine transporter I | 3.9 |  | 510 | Transport |
| DN3627_c0_g1_i2.g49834.t1 | hypothetical protein FOIG_00702 | Exonuclease, RNase T/DNA polymerase III | 2.4 |  | 698 | rRNA processing |
| DN3635_c0_g1_i2.g5108.t1 | hypothetical protein FOTG_12210 | Major facilitator superfamily | 2.0 |  | 442 | Transport |
| DN3657_c0_g1_i1.g5124.t1 | hypothetical protein FOTG_02410 | HRDC domain | 2.0 |  | 524 |  |
| DN368_c0_g1_i11.g32940.t1 | hypothetical protein FOIG_04164 | Zinc finger C2H2-type | 2.3 |  | 394 | Transcription |
| DN3701_c0_g1_i2.g14649.t1 | hypothetical protein FOTG_06875 | P-loop containing nucleoside triphosphate hydrolase | 2.6 |  | 566 | Phosphorylation |
| DN3723_c0_g1_i1.g2804.t1 | DNA-directed RNA polymerase I subunit rpa49 | RNA polymerase I associated factor, A49-like | 2.9 |  | 436 | Transcription |
| DN3735_c1_g1_i1.g2760.t1 | Elongator complex protein 2 | WD domain, G-beta repeat | 4.1 |  | 177 | Signal transduction |
| DN3740_c0_g1_i1.g2716.t1 | hypothetical protein FOQG_00538 | Armadillo-type fold | 1.3 |  | 1210 |  |
| DN3746_c0_g1_i1.g2866.t1 | ATP-dependent RNA helicase suv3, mitochondrial | P-loop containing nucleoside triphosphate hydrolase | 1.6 |  | 685 | Phosphorylation |
| DN3758_c0_g1_i1.g2754.t1 | hypothetical protein FOXG_01332 | Amino acid transporter, transmembrane domain | 4.5 |  | 469 | Transport |
| DN3769_c0_g1_i1.g2724.t1 | hypothetical protein FOQG_09647 | WD domain, G-beta repeat | 1.9 |  | 581 | Signal transduction |
| DN3784_c0_g1_i2.g14552.t1 | CTD kinase subunit alpha | Protein kinase domain | 1.2 |  | 994 | Signal transduction |
| DN389_c0_g1_i2.g16092.t1 | succinyl-CoA ligase | ATP-grasp fold, succinyl-CoA synthetase-type | 1.4 |  | 447 | Metabolic |
| DN39_c0_g1_i3.g4284.t1 | 60S ribosomal protein L2 | Ribosomal protein L2 | 1.1 |  | 254 | Translation |
| DN391_c0_g1_i2.g16003.t1 | AdoMet-dependent rRNA methyltransferase SPB1 | Ribosomal RNA methyltransferase, Spb1, C-terminal | 2.9 |  | 510 | Methylation |
| DN3918_c0_g1_i1.g3196.t1 | Protein SDA1 | SDA1 domain | 1.2 |  | 279 |  |
| DN3941_c0_g1_i6.g35308.t1 | glycerol kinase | Carbohydrate kinase, FGGY, N-terminal | 3.8 |  | 516 | Metabolic |
| DN399_c0_g1_i16.g33912.t1 | hypothetical protein FOTG_00564 | Enoyl-CoA hydratase/isomerase, HIBYL-CoA-H type | 1.1 |  | 504 | Catabolic |
| DN3995_c0_g1_i1.g3219.t1 | probable ATP-dependent RNA helicase | P-loop containing nucleoside triphosphate hydrolase | 2.3 |  | 668 | Phosphorylation |
| DN4017_c0_g1_i1.g14733.t1 | hypothetical protein BFJ69_g14983 |  | 1.3 |  | 982 |  |
| DN4032_c0_g1_i1.g14738.t1 | hypothetical protein BFJ71_g15301 | Tetratricopeptide repeat | 3.7 |  | 1167 | RNA processing |
| DN4056_c0_g1_i1.g14752.t1 | DNA-directed RNA polymerase I subunit RPA2 | DNA-directed RNA polymerase I subunit RPA2, domain 4 | 5.6 |  | 834 | Transcription |
| DN406_c0_g1_i3.g1967.t1 | hypothetical protein FOTG_04099 | Mitochondrial ribosomal protein MRP51, fungi | 1.6 |  | 477 | Translation |
| DN4069_c0_g1_i2.g5241.t1 | DNA repair protein RAD16 | SNF2-related, N-terminal domain | 1.8 |  | 1073 |  |
| DN4076_c0_g1_i1.g14756.t1 | ATP-dependent RNA helicase DBP10 | P-loop containing nucleoside triphosphate hydrolase | 2.4 |  | 867 | Phosphorylation |
| DN4087_c0_g1_i2.g14798.t1 | ATP-dependent RNA helicase MAK5 | P-loop containing nucleoside triphosphate hydrolase | 1.9 |  | 695 | Phosphorylation |
| DN4089_c0_g1_i1.g14749.t1 | elongator complex protein 1 | Elongator complex protein 1 | 1.9 |  | 466 | Uridine modification |
| DN412_c0_g1_i1.g2463.t1 | lysyl-tRNA synthetase, class II | Aminoacyl-tRNA synthetase, class II (D/K/N) | 1.2 |  | 632 | tRNA aminoacylation |
| DN4140_c0_g1_i2.g10856.t1 | hypothetical protein FOTG_07896 | Armadillo-like helical | 2.0 |  | 654 |  |
| DN4142_c0_g1_i2.g12963.t1 | hypothetical protein FOQG_03650 | Pentatricopeptide repeat | 1.9 |  | 705 |  |
| DN4159_c0_g1_i2.g12988.t1 | Ribosomal RNA-processing protein 9 | WD domain, G-beta repeat | 2.1 |  | 546 | Signal transduction |
| DN416_c0_g1_i2.g2511.t1 | hypothetical protein FOXG_00982 | Hypoxia induced protein, domain | 2.2 |  | 231 |  |
| DN4162_c0_g1_i1.g10842.t1 | hypothetical protein FPOA_05897 | RNA polymerase archaeal subunit P/eukaryotic subunit RPABC4 | 1.2 | Mitochondrion | 119 | Transcription |
| DN418_c0_g1_i3.g2198.t1 | hypothetical protein FOTG_09083 | S1 domain | 3.0 |  | 578 |  |
| DN420_c0_g1_i2.g2404.t1 | ATP synthase subunit alpha, mitochondrial | ATPase, F1/V1/A1 complex, alpha/beta subunit, N-terminal domain | 1.2 |  | 552 | Transport |
| DN4232_c0_g1_i1.g13110.t1 | hypothetical protein FOC1_g10015989 | CCAAT-binding factor | 1.5 |  | 452 |  |
| DN4235_c0_g1_i2.g13109.t1 | Annexin A11 | Annexin repeat | 3.7 |  | 273 |  |
| DN4240_c0_g1_i3.g17063.t1 | hypothetical protein FOTG_04324 | P-loop containing nucleoside triphosphate hydrolase | 2.1 |  | 893 | Phosphorylation |
| DN4265_c0_g1_i1.g13176.t1 | hypothetical protein FOCG_02516 | WD domain, G-beta repeat | 1.4 |  | 502 | Signal transduction |
| DN4322_c0_g1_i1.g5921.t1 | General amino acid permease AGP2 | Amino acid permease/ SLC12A domain | 1.5 |  | 301 | Transport |
| DN44_c0_g1_i3.g4329.t1 | plasma membrane ATPase | Cation-transporting P-type ATPase, N-terminal | 2.0 |  | 923 | Transport |
| DN446_c0_g1_i1.g2040.t1 | Eukaryotic peptide chain release factor GTP-binding subunit | Translation elongation factor EFTu/EF1A, C-terminal | 3.0 |  | 703 | Translation |
| DN447_c0_g1_i8.g29247.t1 | Periodic tryptophan protein 2 | WD domain, G-beta repeat | 2.1 |  | 894 | Signal transduction |
| DN4487_c0_g1_i1.g3975.t1 | hypothetical protein FOTG_02727 | Transcription factor domain, fungi | 2.4 |  | 807 | Transcription |
| DN4540_c0_g1_i2.g3948.t1 | tRNA (uracil (54)-C (5))-methyltransferase | (Uracil-5)-methyltransferase family | 1.5 |  | 542 | RNA processing |
| DN4549_c0_g1_i1.g3897.t1 | hypothetical protein FOMG_13855 | Zn (2)-C6 fungal-type DNA-binding domain | 2.3 |  | 689 | Transcription |
| DN4562_c0_g1_i1.g3902.t1 | Phosphoribosylformylglycinamidine synthase | Phosphoribosylformylglycinamidine synthase, N-terminal | 5.4 |  | 310 | Biosynthetic |
| DN4575_c0_g1_i1.g3859.t1 | hypothetical protein BFJ66_g5886 | Transcription factor domain, fungi | 3.1 |  | 740 | Transcription |
| DN4592_c0_g1_i1.g12247.t1 | MFS transporter, FHS family, L-fucose permease | Major facilitator superfamily | 6.7 |  | 394 | Transport |
| DN4605_c0_g1_i1.g9384.t1 | L-aminoadipate-semialdehyde dehydrogenase | AMP-dependent synthetase/ligase | 1.5 |  | 1185 |  |
| DN4607_c0_g1_i2.g9369.t1 | hypothetical protein FOC4_g10003170 |  | 6.2 |  | 228 |  |
| DN461_c0_g1_i5.g2294.t1 | hypothetical protein FAVG1_03363 | Peroxisomal biogenesis factor 11 | 1.2 |  | 235 | Peroxisome fission |
| DN4640_c0_g1_i1.g9380.t1 | hypothetical protein FOXG_02452 | Peptidase C2, calpain, catalytic domain | 3.0 |  | 966 | Proteolysis |
| DN465_c1_g1_i1.g2543.t1 | ATP-dependent RNA helicase HAS1 | P-loop containing nucleoside triphosphate hydrolase | 2.4 |  | 587 | Phosphorylation |
| DN467_c0_g1_i2.g2111.t1 | hypothetical protein FPSE_08661 | Ribosomal protein S11 | 1.5 |  | 151 | Translation |
| DN4671_c0_g1_i1.g9398.t1 | Na(+)/H (+) antiporter | Cation/H+ exchanger | 2.2 |  | 945 | Transport |
| DN4672_c0_g1_i4.g9388.t1 | hypothetical protein FOC1_g10005461 | Major intrinsic protein | 5.7 |  | 331 | Transport |
| DN4675_c0_g1_i2.g2647.t1 | hypothetical protein BFJ68_g329 |  | 5.1 |  | 628 |  |
| DN4723_c0_g1_i1.g19045.t1 | hypothetical protein FOTG_06316 | Protein kinase domain-Histidine kinase/HSP90-like ATPase | 2.3 |  | 1508 | Signal transduction |
| DN4742_c0_g1_i2.g19060.t1 | L-lactate dehydrogenase (cytochrome) | FMN-dependent dehydrogenase | 2.3 |  | 502 |  |
| DN475_c0_g1_i3.g2036.t1 | hypothetical protein FOTG_05902 | Thioesterase domain | 2.1 |  | 272 |  |
| DN477_c0_g1_i1.g2520.t1 | Elongation factor 1-gamma 1 | Glutathione S-transferase | 1.1 |  | 416 | Metabolic |
| DN4796_c0_g1_i1.g19116.t1 | Protein pyrABCN | Carbamoyl-phosphate synthase small chain, CPSase domain | 5.1 |  | 753 | Metabolic |
| DN4811_c0_g1_i1.g50753.t1 | hypothetical protein FOC1_g10006143 |  | 3.3 |  | 271 |  |
| DN485_c0_g1_i4.g27928.t1 | F-type H+-transporting ATPase subunit B | ATP synthase, F0 complex, subunit B/MI25 | 1.1 |  | 242 | Transport |
| DN4875_c0_g1_i1.g918.t1 | rRNA biogenesis protein rrp5 |  | 5.6 |  | 116 |  |
| DN4885_c0_g1_i1.g50842.t1 | MAP kinase kinase kinase wis4 |  | 6.7 |  | 294 |  |
| DN489_c0_g1_i15.g27995.t1 | hypothetical protein FOTG_08907 | Cleavage/polyadenylation specificity factor | 5.3 |  | 1426 | Metabolic |
| DN4890_c0_g1_i2.g932.t1 | hypothetical protein FAVG1_02914 | Ribosomal protein L18e/L15P | 1.2 |  | 149 | Translation |
| DN4917_c0_g1_i2.g6265.t1 | hypothetical protein FOTG_03263 | Short-chain dehydrogenase/reductase SDR | 4.8 |  | 272 | Oxidation-reduction |
| DN4953_c0_g1_i1.g17449.t1 | hypothetical protein FOTG_07367 |  | 2.6 |  | 750 |  |
| DN4961_c0_g1_i1.g17369.t1 | kynureninase 2 | Pyridoxal phosphate-dependent transferase, major domain | 1.4 |  | 493 |  |
| DN4972_c0_g1_i1.g17492.t1 | Vacuolar cation/proton exchanger 2 | Sodium/calcium exchanger membrane region | 3.2 |  | 366 | Transport |
| DN4994_c0_g1_i1.g17477.t1 | hypothetical protein FOQG_08021 | Histone chaperone Rttp106-like | 1.7 |  | 518 | Transcription |
| DN506_c0_g1_i1.g10934.t1 | protein TIF31 | Tetratricopeptide repeat | 3.2 |  | 1261 | RNA processing |
| DN5081_c0_g1_i1.g7665.t1 | succinate-semialdehyde dehydrogenase (NADP+) | Aldehyde dehydrogenase domain | 2.6 |  | 482 | Oxidation-reduction |
| DN5088_c0_g1_i1.g7690.t1 | hypothetical protein FOTG_08614 | Armadillo-type fold | 2.3 |  | 534 |  |
| DN5098_c0_g1_i2.g7627.t1 | glycogen | Glycogen synthase | 2.7 |  | 705 | Biosynthetic |
| DN5142_c0_g1_i1.g5606.t1 | flavohemoglobin | Globin | 5.7 |  | 164 | Transport |
| DN5156_c0_g1_i1.g27223.t1 | hypothetical protein BFJ65_g13442 |  | 5.4 |  | 193 |  |
| DN5251_c0_g1_i1.g48.t1 | Midasin |  | 4.2 |  | 405 |  |
| DN5271_c0_g1_i2.g15.t1 | hypothetical protein BFJ65_g15717 | Amine oxidase | 5.3 |  | 199 | Oxidation-reduction |
| DN532_c0_g1_i8.g10581.t1 | hypothetical protein FOTG_01594 | FAD dependent oxidoreductase | 3.4 |  | 473 | Oxidation-reduction |
| DN532_c0_g1_i8.g10582.t1 | TATA-box-binding protein | TATA-box binding protein | 1.1 | Nucleus | 254 | Transcription |
| DN5499_c0_g1_i1.g50849.t1 | hypothetical protein FOTG_16895 | Short-chain dehydrogenase/reductase SDR | 4.7 |  | 286 | Oxidation-reduction |
| DN5515_c0_g1_i1.g4908.t1 | probable CYB2-lactate dehydrogenase cytochrome b2 | FMN-dependent dehydrogenase | 4.4 |  | 246 | Oxidation-reduction |
| DN555_c0_g1_i3.g10780.t1 | 60S ribosomal protein L7 | Ribosomal protein L30 | 1.1 |  | 248 | Translation |
| DN5566_c0_g1_i1.g4888.t1 | hypothetical protein FOTG_06625 | Zn (2)-C6 fungal-type DNA-binding domain | 7.5 |  | 544 | Transcription |
| DN5596_c0_g1_i2.g20323.t1 | hypothetical protein FOPG_06971 | Alcohol dehydrogenase, N-terminal | 3.8 |  | 360 | Oxidation-reduction |
| DN564_c0_g1_i2.g10916.t1 | probable 40s ribosomal protein S6.e, cytosolic | Ribosomal protein S6e | 2.4 |  | 239 | Translation |
| DN5674_c0_g1_i1.g38434.t1 | Ankyrin repeat domain-containing protein 50 | Ankyrin repeat-containing domain | 4.4 |  | 1043 | Metabolic |
| DN57_c0_g1_i33.g25519.t1 | stress-induced-phosphoprotein 1 | Tetratricopeptide repeat | 6.7 |  | 528 | RNA processing |
| DN572_c0_g1_i1.g10913.t1 | probable isocitrate dehydrogenase (NAD) | Isopropylmalate dehydrogenase-like domain | 1.2 |  | 381 | Oxidation-reduction |
| DN576_c0_g1_i1.g10642.t1 | DNA-directed RNA polymerase I subunit RPA1 | RNA polymerase, alpha subunit | 2.6 |  | 1666 | Transcription |
| DN5767_c0_g1_i1.g17145.t1 | hypothetical protein BFJ69_g14578 |  | 4.0 |  | 95 |  |
| DN582_c0_g1_i13.g29701.t1 | hypothetical protein FOTG_05526 |  | 2.1 |  | 631 |  |
| DN5860_c0_g1_i1.g47731.t1 | hypothetical protein FOC1_g10003033 | RGS domain superfamily | 3.2 |  | 571 | Signal transduction |
| DN5878_c0_g1_i1.g11732.t1 | Phosphoribosylformylglycinamidine synthase | PurM-like, N-terminal domain superfamily | 4.4 |  | 284 |  |
| DN5958_c0_g1_i1.g51036.t1 | hypothetical protein FOTG_09553 | FAD dependent oxidoreductase | 4.5 |  | 477 | Oxidation-reduction |
| DN596_c0_g1_i1.g10601.t1 | Ribosomal RNA assembly protein KRR1 | Krr1, KH1 domain | 1.5 |  | 320 |  |
| DN5981_c0_g1_i2.g51070.t1 | hypothetical protein BFJ71_g13037 | Amine oxidase | 6.2 |  | 470 | Oxidation-reduction |
| DN603_c0_g1_i2.g8809.t1 | CTP synthase | CTP synthase, N-terminal | 2.1 |  | 580 | Biosynthetic |
| DN6055_c0_g1_i2.g37782.t1 | hypothetical protein FOCG_01770 | NmrA-like domain | 4.3 |  | 312 |  |
| DN6061_c0_g1_i2.g37751.t1 | hypothetical protein FOTG_13023 |  | 5.1 |  | 300 |  |
| DN616_c0_g1_i1.g8806.t1 | preli msf1 | PRELI/MSF1 domain | 1.8 |  | 184 |  |
| DN6161_c0_g1_i1.g15573.t1 | Putative WD repeat-containing protein C3D6.12 | U3 small nucleolar RNA-associated SSU processome protein (Utp12) | 2.4 |  | 947 | rRNA processing |
| DN620_c0_g1_i2.g8988.t1 | GTPase | GTP binding domain | 1.2 |  | 394 | Signal transduction |
| DN6219_c0_g1_i1.g6630.t1 | hypothetical protein BFJ70_g11808 | Protein kinase domain | 2.8 |  | 422 | Signal transduction |
| DN632_c0_g1_i3.g8849.t1 | hypothetical protein FSPOR_18 | Nuclear pore protein 84/107 | 2.5 |  | 1148 | Transcription |
| DN632_c1_g1_i3.g8855.t1 | hypothetical protein FOIG_06207 | Paired amphipathic helix | 3.5 |  | 1448 | Transcription |
| DN636_c0_g1_i1.g9091.t1 | 40S ribosomal protein S8 | Ribosomal protein S8e | 1.1 |  | 206 | Translation |
| DN649_c0_g1_i1.g8789.t1 | guanosine-diphosphatase | Nucleoside phosphatase GDA1/CD39 | 1.4 |  | 545 |  |
| DN649_c0_g1_i2.g47419.t1 | hypothetical protein BFJ67_g298 | Tetratricopeptide repeat | 3.0 |  | 1176 | RNA processing |
| DN649_c0_g1_i2.g8791.t1 | guanosine-diphosphatase | Nucleoside phosphatase GDA1/CD39 | 2.2 |  | 550 |  |
| DN6498_c0_g1_i1.g14058.t1 | hypothetical protein FOTG_03075 | tRNA/rRNA methyltransferase, SpoU type | 1.9 |  | 692 | RNA processing |
| DN6516_c0_g1_i1.g43360.t1 | 16S rRNA (-N6/-N6)-dimethyltransferase | Ribosomal RNA adenine methyltransferase KsgA/Erm | 2.0 |  | 343 | Methylation |
| DN6526_c0_g1_i1.g2673.t1 | S-(hydroxymethyl)glutathione synthase | Glutathione-dependent formaldehyde-activating enzyme/centromere protein V | 6.5 |  | 227 | Catabolic |
| DN6638_c0_g1_i1.g124.t1 | hypothetical protein FOMG_14220 | Growth factor receptor cysteine-rich domain superfamily | 5.0 | Extracellular | 87 |  |
| DN6706_c0_g1_i2.g3636.t1 | Protein sda1 | SDA1 domain | 4.2 |  | 485 |  |
| DN671_c0_g1_i3.g9093.t1 | hypothetical protein BFJ69_g466 | Small acidic protein-like domain | 1.3 |  | 345 |  |
| DN6780_c0_g1_i1.g3667.t1 | Putative helicase C6F12.16c | P-loop containing nucleoside triphosphate hydrolase | 5.0 |  | 427 | Phosphorylation |
| DN6782_c0_g1_i2.g3642.t1 | hypothetical protein FOQG_12476 | HPP | 3.9 |  | 285 |  |
| DN690_c0_g1_i1.g8871.t1 | hypothetical protein FOC4_g10007865 |  | 1.8 |  | 574 |  |
| DN691_c0_g1_i17.g47051.t1 | hypothetical protein BFJ69_g9258 | Ribosomal protein S11 | 1.4 |  | 237 | Translation |
| DN6918_c0_g1_i3.g11459.t1 | eukaryotic peptide chain release factor subunit 1 | eRF1 domain 1/Pelota-like | 2.9 |  | 435 |  |
| DN698_c0_g1_i1.g9232.t1 | hypothetical protein FOCG_09020 | RNA recognition motif domain | 1.3 |  | 320 | Cytokinesis |
| DN704_c0_g1_i3.g18325.t1 | ATP-dependent RNA helicase DBP2 | P-loop containing nucleoside triphosphate hydrolase | 4.5 |  | 334 | Phosphorylation |
| DN7042_c0_g1_i1.g288.t1 | tRNA (Met) cytidine acetyltransferase | Acetyltransferase (GNAT) domain | 4.8 |  | 427 | Biosynthetic |
| DN705_c0_g1_i3.g17958.t1 | putative UTP--glucose-1-phosphate uridylyltransferase | UTP--glucose-1-phosphate uridylyltransferase | 3.4 |  | 521 | Metabolic |
| DN7097_c0_g1_i3.g267.t1 | hypothetical protein FOTG_05352 | JmjC domain | 8.6 |  | 1063 |  |
| DN71_c0_g1_i3.g4434.t1 | multiprotein-bridging factor 1 | Multiprotein bridging factor 1 | 1.3 |  | 152 |  |
| DN7297_c0_g1_i1.g18270.t1 | hypothetical protein BFJ69_g11886 |  | 3.0 |  | 390 |  |
| DN73_c0_g1_i2.g4429.t1 | Deoxyhypusine hydroxylase | Armadillo-type fold | 1.8 |  | 330 |  |
| DN734_c0_g1_i3.g35751.t1 | phosphoenolpyruvate carboxykinase | Phosphoenolpyruvate carboxykinase, ATP-utilising | 5.7 |  | 589 | Metabolic |
| DN7362_c0_g1_i1.g15020.t1 | hypothetical protein FOIG_03544 | P-loop containing nucleoside triphosphate hydrolase | 2.0 |  | 777 | Phosphorylation |
| DN738_c0_g1_i1.g18084.t1 | hypothetical protein BFJ69_g5721 | Phosphoglycerate mutase family | 4.0 |  | 609 |  |
| DN742_c0_g1_i2.g18229.t1 | Phenylacetaldehyde dehydrogenase | Aldehyde dehydrogenase domain | 2.0 |  | 467 | Oxidation-reduction |
| DN756_c0_g1_i2.g18093.t1 | probable beta karyopherin | Importin repeat 4 | 2.5 |  | 1096 | Transport |
| DN7590_c0_g1_i1.g4782.t1 | Protein pyrABCN | Carbamoyl-phosphate synthase small subunit, N-terminal domain | 7.5 |  | 691 | Metabolic |
| DN76_c0_g1_i3.g4271.t1 | ATP-dependent RNA helicase DBP2 | P-loop containing nucleoside triphosphate hydrolase | 2.4 |  | 281 | Phosphorylation |
| DN7604_c0_g1_i3.g18706.t1 | hypothetical protein FOTG_10447 |  | 4.4 |  | 199 |  |
| DN765_c0_g1_i3.g35669.t1 | hypothetical protein FOC1_g10002515 | Chaperone J-domain superfamily | 2.8 |  | 444 | Stress response |
| DN7653_c0_g1_i1.g18714.t1 | Heat shock protein SSB | Heat shock protein 70 family | 7.5 |  | 178 | Stress response |
| DN7819_c0_g1_i1.g13803.t1 | hypothetical protein FLONG3_6238 | P-loop containing nucleoside triphosphate hydrolase | 5.1 |  | 116 | Phosphorylation |
| DN7970_c0_g1_i2.g13011.t1 | hypothetical protein BFJ69_g1977 | Expansin, cellulose-binding-like domain superfamily | 5.6 | Extracellular | 220 | Metabolic |
| DN826_c0_g2_i1.g13434.t1 | hypothetical protein BFJ67_g11621 | RNA recognition motif domain | 1.8 |  | 210 | Cytokinesis |
| DN8733_c0_g1_i1.g43213.t1 | hypothetical protein FOTG_05381 |  | 3.6 |  | 440 |  |
| DN886_c0_g1_i9.g13928.t1 | related to LisH motif-containing protein | LIS1 homology motif | 6.5 |  | 247 |  |
| DN8984_c0_g1_i1.g8015.t1 | hypothetical protein BFJ69_g12662 | Fe-ADH-like | 3.2 |  | 254 |  |
| DN9158_c0_g1_i1.g43342.t1 | hypothetical protein FOTG_16235 | Major facilitator, sugar transporter-like | 4.3 |  | 515 | Transport |
| DN917_c0_g1_i5.g15888.t1 | hypothetical protein FOC1_g10012878 | Pentatricopeptide repeat domain | 2.2 |  | 806 |  |
| DN938_c0_g1_i1.g14801.t1 | CK1/CK1/CK1-D protein kinase | Protein kinase domain | 7.2 |  | 371 | Signal transduction |
| DN938_c0_g1_i6.g14802.t1 | hypothetical protein FPOA_04401 | Protein kinase domain | 3.3 |  | 373 | Signal transduction |
