# Supplementary material for: Comparative transcriptomic analysis of races 1, 2, 5 and 6 of Fusarium oxysporum f.sp. pisi in a susceptible pea host identifies differential pathogenicity profiles
Source: BMC Genomics. 2021 Oct 9;22:734. doi: 10.1186/s12864-021-08033-y (PMC8502283; doi:10.1186/s12864-021-08033-y)
Supplement: Supplementary file 6 — Additional file 6: Table S4. Differentially expressed Fop genes detected in R6 at 20 dpi - column 1 with other analyses such as: predicted proteins - column 2, conserved domain - column 3, log2fold change - column 4, subcellular localisation of the effector-like proteins - column 5, protein length - column 6, and GO functional enrichment for biological processes (BP) - column 7. DEGs predicted to be effector-like are shaded yellow and they were all located on the adaptive genome. [file 12864_2021_8033_MOESM6_ESM.docx]

**Supplementary Table 4**

| NODE_101.g12398.t1 | 2,3-dihydroxybenzoate decarboxylase | Amidohydrolase-related | 5.9 |  | 336 | Proteolysis |
| --- | --- | --- | --- | --- | --- | --- |
| NODE_101.g12446.t1 | hypothetical protein FOTG_10844 | Zn (II)2Cys6 (or C6 zinc) binuclear cluster DNA-binding domainGAL4-like | 4.5 |  | 535 | Transcription |
| NODE_102.g12456.t1 | enoyl-CoA hydratase | Enoyl-CoA hydratase/isomerase | 5.2 |  | 281 | Catabolic |
| NODE_103.g12541.t1 | hypothetical protein FOXG_05893 | Major facilitator superfamily | 3.9 |  | 434 | Transport |
| NODE_105.g12630.t1 | cytochrome P450 55A1 | Cytochrome P450 | 5.1 |  | 434 | Oxidation-reduction |
| NODE_107.g3025.t1 | hypothetical protein FOTG_08859 |  | 3.3 |  | 304 |  |
| NODE_109.g12836.t1 | hypothetical protein FOTG_18404 | ATP-citrate lyase/succinyl-CoA ligase | 3.2 |  | 418 | Metabolic |
| NODE_110.g12864.t1 | hypothetical protein FOTG_11411 |  | 3.0 |  | 875 |  |
| NODE_111.g12904.t1 | hypothetical protein BFJ65_g13304 | Major intrinsic protein | 5.8 |  | 251 | Transport |
| NODE_118.g3257.t1 | PiT family inorganic phosphate transporter | Phosphate transporter | 5.3 |  | 607 | Transport |
| NODE_122.g13445.t1 | hypothetical protein FOC1_g10005957 | Carbohydrate-binding domain, family 9 | 4.3 | Extracellular | 221 | Metabolic |
| NODE_130.g3477.t1 | Polyol transporter 5 | Major facilitator, sugar transporter-like | 6.7 |  | 618 | Transport |
| NODE_133.g3552.t1 | related to quinate transport protein | Major facilitator, sugar transporter-like | 7.2 |  | 559 | Transport |
| NODE_134.g13924.t1 | Protein alcS | Acetate transporter GPR1/FUN34/SatP family | 4.4 |  | 289 | Transport |
| NODE_134.g3560.t1 | Hexose transporter HXT15 | Major facilitator, sugar transporter-like | 4.2 |  | 535 | Transport |
| NODE_139.g14125.t1 | hypothetical protein FOTG_14797 |  | 3.4 |  | 361 |  |
| NODE_139.g3662.t1 | arabinogalactan endo-1,4-beta-galactosidase | Glycosyl hydrolase family 53 | 3.6 |  | 348 | Metabolic |
| NODE_143.g3737.t1 | hypothetical protein FOTG_13015 | NACHT nucleoside triphosphatase | 3.8 |  | 1109 | Metabolic |
| NODE_149.g3846.t1 | hypothetical protein FOTG_14494 |  | 7.0 |  | 544 |  |
| NODE_149.g3847.t1 | hypothetical protein FOTG_14493 |  | 4.0 |  | 868 |  |
| NODE_15.g677.t1 | hypothetical protein FOPG_02419 |  | 10.9 | Extracellular | 424 |  |
| NODE_154.g3940.t1 | Oxalate decarboxylase oxdC | Cupin 1 | 5.3 |  | 465 | Metabolic |
| NODE_166.g4157.t1 | hypothetical protein FOTG_14959 |  | 6.7 |  | 240 |  |
| NODE_166.g4160.t1 | Galactose oxidase | galactose oxidase | 7.3 |  | 679 | Oxidation-reduction |
| NODE_172.g15142.t1 | hypothetical protein BFJ67_g1536 |  | 9.0 |  | 433 |  |
| NODE_172.g4260.t1 | hypothetical protein FOMG_09609 | Patatin-like phospholipase domain | 4.3 |  | 567 | Metabolic |
| NODE_173.g15165.t1 | hypothetical protein FOXG_06713 | NTF2-like domain superfamily | 5.9 | Mitochondrion | 90 | Transport |
| NODE_174.g15209.t1 | alcohol oxidase | Glucose-methanol-choline oxidoreductase, N-terminal | 6.1 |  | 559 | Oxidation-reduction |
| NODE_180.g15339.t1 | hypothetical protein FOPG_08978 |  | 4.0 |  | 921 |  |
| NODE_183.g4460.t1 | Carbonic anhydrase | Alpha carbonic anhydrase domain superfamily | 4.7 | Extracellular | 289 | Carbon utilization |
| NODE_185.g15460.t1 | hypothetical protein BFJ69_g14497 | Glycoside hydrolase family 18, catalytic domain | 5.9 |  | 432 | Metabolic |
| NODE_187.g4536.t1 | hypothetical protein FOQG_12546 | Gamma-glutamyl cyclotransferase-like | 4.2 |  | 479 |  |
| NODE_19.g772.t1 | Proline-specific permease | Amino acid/polyamine transporter I | 4.5 |  | 536 | Transport |
| NODE_190.g4595.t1 | prostaglandin-endoperoxide synthase 1 | Haem peroxidase superfamily | 2.5 |  | 1101 | Proteolysis |
| NODE_191.g15592.t1 | Beta-fructofuranosidase, insoluble isoenzyme 3 | Glycoside hydrolase, family 32 | 5.5 |  | 528 | Metabolic |
| NODE_200.g15748.t1 | hypothetical protein FOMG_14220 | Growth factor receptor cysteine-rich domain superfamily | 6.0 | Extracellular | 123 |  |
| NODE_214.g4970.t1 | hypothetical protein BFJ69_g1977 | cellulose-binding-like domain superfamily-Expansin | 4.8 | Extracellular | 220 | Metabolic |
| NODE_221.g16034.t1 | hypothetical protein FOC4_g10003170 |  | 6.1 |  | 225 |  |
| NODE_224.g5124.t1 | murein transglycosylase | Glycosyl hydrolase family 61 | 3.8 |  | 349 | Metabolic |
| NODE_237.g5320.t1 | hypothetical protein FOTG_01219 | Lamin tail domain | 6.7 |  | 349 |  |
| NODE_239.g16199.t1 | Polygalacturonase | Glycoside hydrolase, family 28 | 3.6 |  | 350 | Metabolic |
| NODE_24.g963.t1 | hypothetical protein FOCG_16099 | Major facilitator, sugar transporter-like | 6.0 |  | 499 | Transport |
| NODE_247.g5471.t1 | hypothetical protein BFJ69_g3239 | Protein kinase domain-Alpha kinase | 4.0 |  | 822 | Signal transduction |
| NODE_252.g5546.t1 | endo-1,3(4)-beta-glucanase | Glycoside hydrolase family 16 | 4.3 |  | 286 | Metabolic |
| NODE_259.g5657.t1 | hypothetical protein FOC1_g10006917 | NmrA-like domain | 5.2 |  | 308 |  |
| NODE_264.g5730.t1 | hypothetical protein FOC4_g10003870 |  | 4.6 |  | 299 |  |
| NODE_264.g5734.t1 | serine/threonine protein kinase | Protein kinase domain | 7.6 |  | 548 | Signal transduction |
| NODE_267.g5777.t1 | Endoglucanase 3 | Glycoside hydrolase, family 5 | 4.1 |  | 382 | Metabolic |
| NODE_278.g16416.t1 | hypothetical protein FOC1_g10013863 | Conserved hypothetical protein | 3.8 |  | 300 |  |
| NODE_283.g16433.t1 | hypothetical protein BFJ69_g4369 | Growth factor receptor cysteine-rich domain superfamily | 6.7 | Extracellular | 379 |  |
| NODE_285.g6031.t1 | Isocitrate lyase | Isocitrate lyase | 4.3 |  | 546 | Metabolic |
| NODE_295.g6161.t1 | hypothetical protein FOTG_11204 |  | 5.0 | Extracellular | 168 |  |
| NODE_306.g16513.t1 | hypothetical protein FOTG_16073 |  | 4.7 |  | 123 |  |
| NODE_308.g6325.t1 | hypothetical protein FOTG_03521 |  | 3.5 | Extracellular | 171 |  |
| NODE_326.g6569.t1 | hypothetical protein FOXG_06328 |  | 7.4 |  | 258 |  |
| NODE_329.g6591.t1 | hypothetical protein FOTG_17181 |  | 6.3 | Extracellular | 128 |  |
| NODE_329.g6599.t1 | General alpha-glucoside permease | Major facilitator, sugar transporter-like | 4.2 |  | 561 | Transport |
| NODE_34.g1268.t1 | hypothetical protein FOTG_14582 | Rossmann-like alpha/beta/alpha sandwich fold | 4.3 |  | 288 |  |
| NODE_345.g6810.t1 | hypothetical protein FOTG_12938 | Ankyrin repeat | 6.2 |  | 502 | Metabolic |
| NODE_345.g6811.t1 | hypothetical protein BFJ65_g1631 |  | 4.5 |  | 299 |  |
| NODE_378.g7204.t1 | hypothetical protein BFJ69_g14850 | Major facilitator, sugar transporter-like | 3.4 |  | 507 | Transport |
| NODE_387.g7304.t1 | hypothetical protein FOCG_11444 | Cation/H+ exchanger | 3.6 |  | 658 | Transport |
| NODE_39.g1402.t1 | hypothetical protein FOTG_10453 | Tetratricopeptide repeat | 4.8 |  | 2148 | RNA processing |
| NODE_4.g191.t1 | hypothetical protein FOC4_g10005707 | Amine oxidase | 5.5 |  | 640 | Oxidation-reduction |
| NODE_4.g194.t1 | hypothetical protein FOXG_13238 | Conserved hypothetical protein | 4.5 |  | 340 |  |
| NODE_416.g7626.t1 | hypothetical protein FOXG_13102 |  | 5.4 |  | 467 |  |
| NODE_42.g1469.t1 | hypothetical protein FOXG_10949 |  | 8.5 | Extracellular | 174 |  |
| NODE_42.g1470.t1 | hypothetical protein FOC4_g10007522 |  | 7.1 | Extracellular | 118 |  |
| NODE_432.g7810.t1 | MFS transporter, SP family, general alpha glucoside:H+ symporter | Major facilitator, sugar transporter-like | 4.4 |  | 548 | Transport |
| NODE_433.g7822.t1 | hypothetical protein BFJ65_g10346 |  | 3.6 |  | 109 |  |
| NODE_441.g7901.t1 | hypothetical protein BFJ65_g12668 | Proline rich extensin signature | 7.0 |  | 245 |  |
| NODE_452.g8030.t1 | probable CYB2-lactate dehydrogenase cytochrome b2 | Alpha-hydroxy acid dehydrogenase, FMN-dependent | 6.0 |  | 383 | Oxidation-reduction |
| NODE_5.g241.t1 | Pisatin demethylase | Cytochrome P450 | 3.4 |  | 544 | Oxidation-reduction |
| NODE_538.g8878.t1 | Sugar transporter STL1 | Sugar transporter, conserved site | 3.7 |  | 540 | Transport |
| NODE_538.g8886.t1 | hypothetical protein FOMG_04343 | methyltransferase | 3.6 |  | 581 | Methylation |
| NODE_542.g8919.t1 | hypothetical protein BFJ71_g13037 | Amine oxidase | 5.6 |  | 625 | Oxidation-reduction |
| NODE_552.g9021.t1 | S-(hydroxymethyl)glutathione synthase | Glutathione-dependent formaldehyde-activating enzyme | 5.8 |  | 227 | Catabolic |
| NODE_555.g9053.t1 | MFS transporter, FHS family, L-fucose permease | Major facilitator superfamily | 6.7 |  | 461 | Transport |
| NODE_560.g9103.t1 | Putative peptide transporter ptr2 | Proton-dependent oligopeptide transporter family | 4.8 |  | 611 | Transport |
| NODE_57.g1857.t1 | hypothetical protein BFJ69_g7592 | Fasciclin domain | 3.8 |  | 731 |  |
| NODE_6.g307.t1 | Choline transport protein | Amino acid/polyamine transporter I | 4.9 |  | 531 | Transport |
| NODE_61.g1953.t1 | hypothetical protein FOC1_g10003496 | Lactonase, 7-bladed beta propeller | 4.2 |  | 401 |  |
| NODE_63.g2016.t1 | choline dehydrogenase | Glucose-methanol-choline oxidoreductase, N-terminal | 4.8 |  | 640 | Oxidation-reduction |
| NODE_637.g9791.t1 | hypothetical protein FOTG_03461 |  | 4.8 |  | 260 |  |
| NODE_676.g10109.t1 | General amino acid permease AGP2 | Amino acid/polyamine transporter I | 5.9 |  | 485 | Transport |
| NODE_677.g10118.t1 | hypothetical protein FOTG_16328 | Major facilitator, sugar transporter-like | 4.8 |  | 557 | Transport |
| NODE_719.g10448.t1 | hypothetical protein FOTG_04359 |  | 4.5 |  | 433 |  |
| NODE_730.g10532.t1 | Aldehyde dehydrogenase | Aldehyde dehydrogenase, C-terminal | 3.9 |  | 495 | Oxidation-reduction |
| NODE_758.g10751.t1 | hypothetical protein FOC1_g10004355 | Glycosyl hydrolases family 16 | 4.7 |  | 811 | Metabolic |
| NODE_766.g10820.t1 | probable translation elongation factor eEF-3 | ABC transporter-like | 3.2 |  | 1055 | Transport |
| NODE_771.g10864.t1 | probable formate dehydrogenase | D-isomer specific 2-hydroxyacid dehydrogenase, catalytic domain | 3.2 |  | 365 | Oxidation-reduction |
| NODE_791.g11026.t1 | ADP, ATP carrier protein | Mitochondrial carrier protein | 1.7 |  | 312 | Transport |
| NODE_89.g2623.t1 | hypothetical protein FOTG_05467 |  | 3.5 |  | 221 |  |
| NODE_895.g11723.t1 | hypothetical protein FOC4_g10015188 | Protein of unknown function DUF3712 | 5.9 |  | 343 |  |
| NODE_919.g11894.t1 | hypothetical protein FOXG_01504 | Pyridine nucleotide-disulphide oxidoreductase | 4.4 |  | 390 | Oxidation-reduction |
| NODE_954.g12100.t1 | hypothetical protein FOPG_17154 | Tetratricopeptide repeat | 6.9 |  | 1068 | RNA processing |
| NODE_97.g2804.t1 | hypothetical protein FOTG_15008 | Pectinesterase, catalytic | 4.5 |  | 329 | Metabolic |
| NODE_98.g2817.t1 | nitric oxide dioxygenase | Oxidoreductase FAD/NAD(P)-binding | 3.9 |  | 415 | Oxidation-reduction |
| DN10613_c0_g1_i1.g23.t1 | Pectinesterase | Pectinesterase, catalytic | 4.2 |  | 243 | Metabolic |
| DN11944_c0_g1_i2.g13465.t1 | hypothetical protein FOXG_08912 | Amino acid/polyamine transporter I | 5.2 |  | 540 | Transport |
| DN12959_c0_g1_i1.g13104.t1 | hypothetical protein BFJ68_g6975 | Major facilitator, sugar transporter-like | 4.6 |  | 141 | Transport |
| DN14371_c0_g1_i1.g15943.t1 | hypothetical protein FAVG1_09499 | Cell wall mannoprotein 1 | 4.8 | Cytoplasm | 111 | Fungal-type cell wall organization |
| DN16839_c0_g1_i1.g1411.t1 | Heat shock protein SSB | Heat shock protein 70 family | 5.3 |  | 177 | Stress response |
| DN17376_c0_g1_i1.g15772.t1 | hypothetical protein FOXG_06133 |  | 4.7 |  | 129 |  |
| DN17490_c0_g1_i1.g18966.t1 | Ca2+-transporting ATPase | P-type ATPase, cytoplasmic domain N | 3.6 |  | 191 | Transport |
| DN1784_c0_g1_i1.g19985.t1 | hypothetical protein FOTG_07898 | Zn (2)-C6 fungal-type DNA-binding domain-GAL4 | 4.7 |  | 591 | Transcription |
| DN17917_c0_g1_i1.g19797.t1 | hypothetical protein BFJ71_g6736 | Amine oxidase | 4.1 |  | 200 | Oxidation-reduction |
| DN18847_c0_g1_i1.g10493.t1 | hypothetical protein FOXG_22929 |  | 4.3 | Extracellular | 118 |  |
| DN3245_c0_g1_i6.g16352.t1 | hypothetical protein FOC1_g10005461 | Major intrinsic protein | 5.7 |  | 331 | Transport |
| DN3822_c0_g1_i1.g17164.t1 | hypothetical protein FOTG_14648 |  | 7.5 | Extracellular | 151 |  |
| DN3942_c0_g1_i1.g18153.t1 | Putative quinate permease | Major facilitator, sugar transporter-like | 6.2 |  | 185 | Transport |
| DN5455_c0_g1_i1.g6913.t1 | hypothetical protein BFJ65_g15373 | FAD/NAD(P)-binding domain superfamily | 2.4 |  | 161 | Oxidation-reduction |
| DN623_c0_g1_i1.g16491.t1 | hypothetical protein FOMG_19844 | NTF2-like domain superfamily | 6.0 |  | 155 | Transport |
| DN6853_c0_g1_i1.g18528.t1 | Elongation factor 1-alpha | GTP-binding elongation factor | 1.7 |  | 460 | Signal transduction |
| DN7045_c0_g1_i1.g14084.t1 | Psi-producing oxygenase C | Animal haem peroxidase | 3.7 |  | 208 | Proteolysis |
| DN7206_c0_g1_i1.g5166.t1 | hypothetical protein FOXG_00487 | Armadillo-type fold | 4.6 |  | 1281 |  |
| DN7520_c0_g1_i1.g3441.t1 | hypothetical protein FOQG_15399 | Fasciclin domain | 3.7 |  | 217 |  |
| DN753_c0_g1_i3.g2245.t1 | hypothetical protein FOTG_08845 | Major facilitator superfamily | 6.2 |  | 500 | Transport |
| DN9457_c0_g1_i1.g15949.t1 | hypothetical protein BFJ69_g3238 | Ankyrin repeat-containing domain superfamily | 3.3 |  | 249 | Metabolic |
