# Supplementary material for: Comparative transcriptomic analysis of races 1, 2, 5 and 6 of Fusarium oxysporum f.sp. pisi in a susceptible pea host identifies differential pathogenicity profiles
Source: BMC Genomics. 2021 Oct 9;22:734. doi: 10.1186/s12864-021-08033-y (PMC8502283; doi:10.1186/s12864-021-08033-y)
Supplement: Supplementary file 7 — Additional file 7: Table S5. Differentially expressed genes in R1 that mapped to the virulence-associated genes on the PHI-base database. [file 12864_2021_8033_MOESM7_ESM.docx]

**Supplementary Table 5**

| **Unigene** | **PHI No.** | **Sequence homology to** | **Phytopathogens** | **Gene knockout result** | **Predicted protein** | **Protein domain** |
| --- | --- | --- | --- | --- | --- | --- |
| DN22201_c0_g1_i1.g17051.t1 | PHI:3216 | MoCDIP4 | *Magnaporthe_oryzae* | effector | hypothetical protein BFJ69_g4370 | Glycoside hydrolase, family 61 |
| DN5768_c0_g1_i1.g19843.t1 | PHI:3216 | MoCDIP4 | *Magnaporthe_oryzae* | effector | hypothetical protein BFJ69_g6260 | Glycoside hydrolase, family 61 (AA9 CAZyme) |
| NODE_107.g3183.t1 | PHI:3216 | MoCDIP4 | *Magnaporthe_oryzae* | effector | Putative endo-beta-1,4-glucanase D | Cellulose-binding domain, fungal |
| NODE_302.g6444.t1 | PHI:2216 | PemG1 | *Magnaporthe_oryzae* | effector | hypothetical protein BFJ69_g6142 | Stm1-like, N-terminal |
| DN17673_c0_g1_i1.g17447.t1 | PHI:1227 | FGSG_06089 | *Fusarium_graminearum* | lethal | Transcription-associated protein 1 | Belongs to the PI3 PI4-kinase family |
| NODE_103.g12525.t1 | PHI:1569 | GzOB009 | *Fusarium_graminearum* | lethal | 50S ribosomal protein L2 | Ribosomal Proteins L2, RNA binding domain |
| NODE_218.g15716.t1 | PHI:1226 | FGSG_05845 | *Fusarium_graminearum* | lethal | Serine/threonine-protein kinase psk1 | Protein kinase domain |
| NODE_384.g7504.t1 | PHI:1546 | GzMyb010 | *Fusarium_graminearum* | lethal | chromatin modification-like protein VID21 | Homeobox-like domain superfamily |
| DN15496_c0_g1_i1.g46603.t1 | PHI:4639 | Cbp1 | *Magnaporthe_oryzae* | loss_of_pathogenicity | hypothetical protein FOXG_02597 | NodB homology domain |
| DN2055_c0_g1_i1.g9790.t1 | PHI:4475 | MoTup1 | *Magnaporthe_oryzae* | loss_of_pathogenicity | probable CPC2 protein | WD domain, G-beta repeat |
| DN21863_c0_g1_i1.g14013.t1 | PHI:341 | CPK1 | *Colletotrichum_lagenaria* | loss_of_pathogenicity | cAMP-dependent protein kinase type 2 | Protein kinase domain-cAMP-dependent protein kinase |
| DN3038_c0_g1_i1.g381.t1 | PHI:9101 | Mopex14_(MGG_01028) | *Magnaporthe_oryzae* | loss_of_pathogenicity | hypothetical protein FOIG_06146 | Peroxisome membrane anchor protein Pex14p, N-terminal |
| DN3953_c0_g1_i1.g1628.t1 | PHI:157 | TOXF | *Bipolaris_zeicola* | loss_of_pathogenicity | hypothetical protein FOCG_05131 | Aminotransferase class IV |
| NODE_135.g3788.t1 | PHI:3375 | FgSte50 | *Fusarium_graminearum* | loss_of_pathogenicity | hypothetical protein FOQG_00510 | Sterile alpha motif/pointed domain superfamily |
| NODE_138.g14072.t1 | PHI:8884 | aroG1 | *Ralstonia_solanacearum* | loss_of_pathogenicity | Phospho-2-dehydro-3-deoxyheptonate aldolase, tyrosine-inhibited | DHAP synthase, class 1 |
| NODE_182.g4648.t1 | PHI:3161 | Mohik5 | *Magnaporthe_oryzae* | loss_of_pathogenicity | hypothetical protein BFJ71_g2358 | Protein kinase domain-Histidine kinase, dimerisation/phosphoacceptor domain |
| NODE_294.g6342.t1 | PHI:407 | PBC1 | *Pyrenopeziza_brassicae* | loss_of_pathogenicity | cutinase | Cutinase |
| NODE_38.g1450.t1 | PHI:2387 | ACL2 | *Fusarium_graminearum* | loss_of_pathogenicity | ATP-citrate synthase subunit 2 | ATP-citrate synthase, citrate-binding domain |
| NODE_395.g7629.t1 | PHI:734 | FOW2 | *Fusarium_oxysporum* | loss_of_pathogenicity | hypothetical protein BFJ69_g5063 | Transcription factor domain, fungi |
| NODE_429.g8017.t1 | PHI:365__PHI:2267 | MLS1__Mls1 | *Parastagonospora_nodorum* | loss_of_pathogenicity | malate synthase, glyoxysomal | Malate synthase |
| NODE_54.g1909.t1 | PHI:157 | TOXF | *Bipolaris_zeicola* | loss_of_pathogenicity | Putative branched-chain-amino-acid aminotransferase TOXF | Branched-chain amino acid aminotransferase II |
| NODE_54.g1911.t1 | PHI:4475 | MoTup1 | *Magnaporthe_oryzae* | loss_of_pathogenicity | hypothetical protein FOXG_08701 | WD domain, G-beta repeat |
| NODE_71.g2345.t1 | PHI:294 | CST1 | *Colletotrichum_lagenaria* | loss_of_pathogenicity | Transcription factor steA | zinc fingers C2H2 - Ste12 |
| NODE_815.g11287.t1 | PHI:4639 | Cbp1 | *Magnaporthe_oryzae* | loss_of_pathogenicity | Bifunctional xylanase/deacetylase | NodB homology domain |
| NODE_86.g2691.t1 | PHI:5232 | MoARG1 | *Magnaporthe_oryzae* | loss_of_pathogenicity | Argininosuccinate synthase | Argininosuccinate synthase |
| NODE_887.g11755.t1 | PHI:6331 | VdCYP1 | *Verticillium_dahliae* | loss_of_pathogenicity | hypothetical protein BFJ67_g9277 | Cytochrome P450 superfamily |
| NODE_93.g2856.t1 | PHI:9357 | Fgleu1_(FGSG_09589) | *Fusarium_graminearum* | loss_of_pathogenicity | Aconitate hydratase | Aconitase, mitochondrial-like |
| DN5393_c0_g1_i1.g11683.t1 | PHI:2115__PHI:4494 | Annexin_A7__AnnA7 | *Magnaporthe_oryzae* | loss_of_pathogenicity | Annexin A11 | Annexin repeat |
| NODE_109.g12879.t1 | PHI:2145__PHI:9180 | MoSSADH__MoSSADH_(MGG_01230) | *Magnaporthe_oryzae* | loss_of_pathogenicity | Aldehyde dehydrogenase | Aldehyde dehydrogenase, C-terminal |
| NODE_708.g10527.t1 | PHI:2145__PHI:9180 | MoSSADH__MoSSADH_(MGG_01230) | *Magnaporthe_oryzae* | loss_of_pathogenicity | betaine-aldehyde dehydrogenase | Aldehyde dehydrogenase domain |
| NODE_101.g12451.t1 | PHI:5016 | Psimpa1 | *Phytophthora_sojae* | reduced_virulence | uncharacterized protein FVRRES_11925 | Importin-alpha, importin-beta-binding domain |
| NODE_103.g12546.t1 | PHI:2239 | Spe-Sdh | *Ustilago_maydis* | reduced_virulence | saccharopine dehydrogenase | Saccharopine dehydrogenase, NADP binding domain |
| NODE_105.g12650.t1 | PHI:1364 | GzC2H024 | *Fusarium_graminearum* | reduced_virulence | hypothetical protein FOC1_g10010447 | Zinc finger C2H2-type |
| NODE_112.g12973.t1 | PHI:442 | MSY1 | *Fusarium_graminearum* | reduced_virulence | Putative 5-methyltetrahydropteroyltriglutamate--homocysteine methyltransferase | Cobalamin-independent methionine synthase MetE, C-terminal/archaeal |
| NODE_114.g3331.t1 | PHI:254 | FOW1 | *Fusarium_oxysporum* | reduced_virulence | Citrate/oxoglutarate carrier protein | Mitochondrial substrate/solute carrier |
| NODE_117.g3408.t1 | PHI:2240__PHI:4586 | Srt1 | *Ustilago_maydis* | reduced_virulence | Putative glucose transporter rco-3 | Major facilitator, sugar transporter-like |
| NODE_118.g13250.t1 | PHI:9282 | mfsG_(BCIN_06g00026) | *Botrytis_cinerea* | reduced_virulence | hypothetical protein BFJ65_g8824 | Major facilitator superfamily |
| NODE_118.g3423.t1 | PHI:3914 | Sdh1 | *Parastagonospora_nodorum* | reduced_virulence | succinate-semialdehyde dehydrogenase (NADP+) | Aldehyde dehydrogenase, C-terminal |
| NODE_121.g3503.t1 | PHI:8858 | VdSSEP1_(VDAG-08100) | *Verticillium_dahliae* | reduced_virulence | Minor extracellular protease vpr | Peptidase S8/S53 domain |
| NODE_121.g3504.t1 | PHI:2920__PHI:9074 | FET3-1__fet3-1 | *Colletotrichum_graminicola* | reduced_virulence | Laccase | Multicopper oxidase, type 2 |
| NODE_122.g13452.t1 | PHI:8037 | Fvlds1 | *Fusarium_verticillioides* | reduced_virulence | hypothetical protein BFJ69_g6430 | Haem peroxidase superfamily |
| NODE_122.g3508.t1 | PHI:2117 | SPM1 | *Magnaporthe_oryzae* | reduced_virulence | Alkaline proteinase | Peptidase S8/S53 domain |
| NODE_126.g13618.t1 | PHI:9104 | FGRRES_16221 | *Fusarium_graminearum* | reduced_virulence | hypothetical protein BFJ69_g6466 | integral membrane protein |
| NODE_131.g13830.t1 | PHI:9062 | katG2_(FGSG_12369) | *Fusarium_graminearum* | reduced_virulence | catalase-peroxidase | Haem peroxidase |
| NODE_131.g13839.t1 | PHI:744 | ADE5 | *Fusarium_graminearum* | reduced_virulence | hypothetical protein FOXG_10535 | Phosphoribosylglycinamide synthetase, C-domain |
| NODE_135.g13955.t1 | PHI:179 | PELA | *Nectria_haematococca* | reduced_virulence | pectate lyase | Pectate lyase PlyH/PlyE-like |
| NODE_135.g13958.t1 | PHI:2178 | MoSFl1 | *Magnaporthe_oryzae* | reduced_virulence | hypothetical protein BFJ65_g11160 | Heat shock factor (HSF)-type, DNA-binding |
| NODE_135.g3794.t1 | PHI:5068 | MCC | *Fusarium_graminearum* | reduced_virulence | Pyruvate carboxylase | Pyruvate carboxylase |
| NODE_136.g14003.t1 | PHI:1470 | GzHMG002 | *Fusarium_graminearum* | reduced_virulence | hypothetical protein BFJ71_g7230 | High mobility group box domain |
| NODE_14.g638.t1 | PHI:180 | PELD | *Nectria_haematococca* | reduced_virulence | Putative pectate lyase F | Pectate lyase PlyH/PlyE-like |
| NODE_14.g646.t1 | PHI:5393 | GLX | *Fusarium_oxysporum* | reduced_virulence | hypothetical protein FOCG_08422 | Glyoxal oxidase, N-terminal |
| NODE_143.g14261.t1 | PHI:4491 | SHM | *Magnaporthe_oryzae* | reduced_virulence | serine hydroxymethyltransferase, mitochondrial | Serine hydroxymethyltransferase |
| NODE_146.g14329.t1 | PHI:1188 | (Sc_Sak1) | *Fusarium_graminearum* | reduced_virulence | CAMKK protein kinase | Protein kinase domain-CAMKK |
| NODE_147.g14377.t1 | PHI:1641__PHI:2434 | FgFlbA | *Fusarium_graminearum* | reduced_virulence | hypothetical protein FOXG_08482 | RGS domain |
| NODE_160.g4261.t1 | PHI:1093 | FGSG_02077 | *Fusarium_graminearum* | reduced_virulence | uncharacterized protein FMAN_11213 | CFEM domain |
| NODE_166.g14923.t1 | PHI:816 | MGG_04582 | *Magnaporthe_oryzae* | reduced_virulence | Putative beta-glucosidase btgE | Glycoside hydrolase superfamily |
| NODE_180.g4632.t1 | PHI:1180 | (Sc_Sat4) | *Fusarium_graminearum* | reduced_virulence | HAL protein kinase | Protein kinase domain-HAL |
| NODE_181.g4638.t1 | PHI:7030 | FgLAI12 | *Fusarium_graminearum* | reduced_virulence | hypothetical protein FOMG_17002 | Haem peroxidase superfamily |
| NODE_211.g5124.t1 | PHI:3019 | MoLys2 | *Magnaporthe_oryzae* | reduced_virulence | L-2-aminoadipate reductase large subunit | L-2-aminoadipate reductase |
| NODE_235.g5493.t1 | PHI:319 | SQL2 | *Ustilago_maydis* | reduced_virulence | hypothetical protein FOIG_07922 | guanine-nucleotide exchange factors catalytic domain (Ras-GTPase) |
| NODE_238.g5556.t1 | PHI:7143 | FvCYP1 | *Fusarium_verticillioides* | reduced_virulence | Peptidyl-prolyl cis-trans isomerase, mitochondrial | Cyclophilin-type peptidyl-prolyl cis-trans isomerase domain |
| NODE_244.g15881.t1 | PHI:350 | EMP1 | *Magnaporthe_oryzae* | reduced_virulence | hypothetical protein BFJ65_g720 | glycosyl-phosphatidyl-inositol-anchored membrane family (GPI) |
| NODE_26.g1114.t1 | PHI:3019 | MoLys2 | *Magnaporthe_oryzae* | reduced_virulence | hypothetical protein BFJ65_g3552 | AMP-dependent synthetase/ligase |
| NODE_260.g5879.t1 | PHI:1382 | GzC2H045 | *Fusarium_graminearum* | reduced_virulence | hypothetical protein BFJ66_g7086 | Zinc finger C2H2-type |
| NODE_262.g5900.t1 | PHI:8580 | purL_(Dda3937_03379) | *Dickeya_dadantii* | reduced_virulence | Phosphoribosylformylglycinamidine synthase | Phosphoribosylformylglycinamidine synthase, N-terminal |
| NODE_270.g6015.t1 | PHI:1090__PHI:1163__PHI:3893 | FgEBR1 | *Fusarium_graminearum* | reduced_virulence | hypothetical protein FOC1_g10012848 | Zn (2)-C6 fungal-type DNA-binding domain superfamily |
| NODE_273.g6061.t1 | PHI:1177 | nrc-2 | *Fusarium_graminearum* | reduced_virulence | AGC/RSK/RSK-UNCLASSIFIED protein kinase | Protein kinase domain-AGC |
| NODE_279.g6151.t1 | PHI:2058 | LHS1 | *Magnaporthe_oryzae* | reduced_virulence | probable heat shock protein 70 | Heat shock protein 70 family |
| NODE_300.g6429.t1 | PHI:812 | MGG_10702 | *Magnaporthe_oryzae* | reduced_virulence | hypothetical protein BFJ65_g12933 | Major facilitator superfamily |
| NODE_303.g6449.t1 | PHI:158 | UKC1 | *Ustilago_maydis* | reduced_virulence | hypothetical protein FPOA_00733 | Protein kinase domain-AGC-kinase |
| NODE_315.g6612.t1 | PHI:443 | CBL1 | *Fusarium_graminearum* | reduced_virulence | cystathionine beta-lyase | Cys/Met metabolism, pyridoxal phosphate-dependent enzyme |
| NODE_341.g6976.t1 | PHI:382__PHI:2233 | SMU1__Smu1 | *Ustilago_maydis* | reduced_virulence | STE/STE20/PAKA protein kinase | Protein kinase domain |
| NODE_348.g7058.t1 | PHI:2207 | endo-1_4-beta-xylanase_[GH10_family] | *Magnaporthe_oryzae* | reduced_virulence | endoglucanase type F | Glycoside hydrolase family 10 domain |
| NODE_36.g1409.t1 | PHI:5068 | MCC | *Fusarium_graminearum* | reduced_virulence | probable acetyl-CoA carboxylase | Acetyl-CoA carboxylase, central domain |
| NODE_364.g7273.t1 | PHI:305 | ICL1 | *Magnaporthe_oryzae* | reduced_virulence | isocitrate lyase | Isocitrate lyase |
| NODE_370.g7338.t1 | PHI:2240__PHI:4586 | Srt1 | *Ustilago_maydis* | reduced_virulence | Maltose permease MAL61 | Major facilitator, sugar transporter-like |
| NODE_407.g7776.t1 | PHI:3973 | ASP | *Colletotrichum_gloeosporioides* | reduced_virulence | hypothetical protein FOXG_16638 | Aspartic peptidase A1 family |
| NODE_407.g7784.t1 | PHI:2976 | CgOPT1 | *Colletotrichum_gloeosporioides* | reduced_virulence | Oligopeptide transporter 3 | Oligopeptide transporter, OPT superfamily |
| NODE_451.g8245.t1 | PHI:7281 | PsINV | *Puccinia_striiformis* | reduced_virulence | acid beta-fructofuranosidase precursor | Glycoside hydrolase, family 32 |
| NODE_458.g8310.t1 | PHI:7741 | FreB | *Verticillium_dahliae* | reduced_virulence | hypothetical protein FOCG_09313 | Ferric reductase, NAD binding domain |
| NODE_48.g1739.t1 | PHI:2239 | Spe-Sdh | *Ustilago_maydis* | reduced_virulence | spermidine synthase | Spermidine/spermine synthases |
| NODE_493.g8684.t1 | PHI:2240__PHI:4586 | Srt1 | *Ustilago_maydis* | reduced_virulence | Putative quinate permease | Major facilitator, sugar transporter-like |
| NODE_509.g8836.t1 | PHI:1384 | GzC2H047 | *Fusarium_graminearum* | reduced_virulence | hypothetical protein FOXG_01756 | Zinc finger C2H2-type |
| NODE_526.g8974.t1 | PHI:881 | MGG_04556 | *Magnaporthe_oryzae* | reduced_virulence | hypothetical protein BFJ69_g1507 | Polyketide synthase, enoylreductase domain |
| NODE_540.g9103.t1 | PHI:5826 | GIV4 | *Fusarium_graminearum* | reduced_virulence | hypothetical protein FOC1_g10003508 | integral membrane protein |
| NODE_544.g9152.t1 | PHI:2351__PHI:2353 | AMT1 | *Fusarium_graminearum* | reduced_virulence | probable hnRNP arginine N-methyltransferase | arginine N-methyltransferase |
| NODE_56.g1959.t1 | PHI:177 | ODC | *Parastagonospora_nodorum* | reduced_virulence | ornithine decarboxylase | Orn/DAP/Arg decarboxylase 2, C-terminal |
| NODE_566.g9354.t1 | PHI:2165 | PTH11 | *Magnaporthe_oryzae* | reduced_virulence | hypothetical protein BFJ65_g17320 | Carbohydrate-binding, CenC-like |
| NODE_592.g9595.t1 | PHI:2921__PHI:9075 | FET3-2__fet3-2 | *Colletotrichum_graminicola* | reduced_virulence | hypothetical protein FOCG_01584 | Multicopper oxidase, type 2 |
| NODE_611.g9752.t1 | PHI:1209 | FGSG_04770 | *Fusarium_graminearum* | reduced_virulence | hypothetical protein BFJ70_g4924 | Protein kinase domain |
| NODE_617.g9818.t1 | PHI:2343 | Pcipg2 | *Phytophthora_capsici* | reduced_virulence | putative exopolygalacturonase X | Glycoside hydrolase, family 28 |
| NODE_62.g2116.t1 | PHI:8646 | FgLDHL2_(FGSG_16220) | *Fusarium_graminearum* | reduced_virulence | L-lactate dehydrogenase (cytochrome) | Cytochrome b5-like heme/steroid binding domain |
| NODE_630.g9918.t1 | PHI:1217 | FGSG_00792 | *Fusarium_graminearum* | reduced_virulence | CAMK/CAMKL/KIN4 protein kinase | Protein kinase domain-CAMK/CAMKL/KIN4 protein kinase |
| NODE_661.g10166.t1 | PHI:3816 | GSN1 | *Magnaporthe_oryzae* | reduced_virulence | glycogen | Glycogen synthase |
| NODE_664.g10193.t1 | PHI:6261 | PsAAT3 | *Phytophthora_sojae* | reduced_virulence | aspartate aminotransferase, mitochondrial | Aspartate/other aminotransferase |
| NODE_684.g10350.t1 | PHI:5480 | MoPyr5 | *Magnaporthe_oryzae* | reduced_virulence | orotate phosphoribosyltransferase | Phosphoribosyltransferase domain |
| NODE_706.g10510.t1 | PHI:1422 | GzC2H090 | *Fusarium_graminearum* | reduced_virulence | hypothetical protein BFJ69_g14025 | Dual specificity protein phosphatase domain |
| NODE_74.g2420.t1 | PHI:4658 | FgHXK1 | *Fusarium_graminearum* | reduced_virulence | Hexokinase-1 | Hexokinase, N-terminal |
| NODE_74.g2421.t1 | PHI:5472 | MoDeam | *Magnaporthe_oryzae* | reduced_virulence | Glucosamine-6-phosphate isomerase 1 | Glucosamine-6-phosphate isomerase |
| NODE_801.g11185.t1 | PHI:9104 | FGRRES_16221 | *Fusarium_graminearum* | reduced_virulence | hypothetical protein FOCG_11444 | Cation/H+ exchanger |
| NODE_804.g11207.t1 | PHI:2432 | FgRgsA | *Fusarium_graminearum* | reduced_virulence | hypothetical protein BFJ71_g14823 | RGS domain superfamily |
| NODE_852.g11557.t1 | PHI:2171 | Peroxisomal_copper_amine_oxidase | *Magnaporthe_oryzae* | reduced_virulence | Peroxisomal primary amine oxidase | Copper amine oxidase, N3-terminal |
| NODE_877.g11700.t1 | PHI:2240__PHI:4586 | Srt1 | *Ustilago_maydis* | reduced_virulence | MFS transporter, SP family, general alpha glucoside:H+ symporter | Major facilitator, sugar transporter-like |
| NODE_930.g12005.t1 | PHI:7144 | FvSCP1 | *Fusarium_verticillioides* | reduced_virulence | Protein PRY1 | Allergen V5/Tpx-1-related, conserved site |
| NODE_937.g12042.t1 | PHI:9408 | VdSkn7_(VDAG_02250) | *Verticillium_dahliae* | reduced_virulence | hypothetical protein FOXG_08113 | Heat shock factor (HSF)-type, DNA-binding |
| NODE_953.g12125.t1 | PHI:7173 | HiC-15 | *Verticillium_dahliae* | reduced_virulence | hypothetical protein BFJ70_g17240 | Cytochrome P450 |
| DN10792_c0_g1_i1.g14344.t1 | PHI:8753__PHI:8806 | MoChia1_(MGG_08054)__MoChi1_(MGG_08054) | *Magnaporthe_oryzae* | reduced_virulence | chitinase | Glycoside hydrolase family 18, catalytic domain |
| DN1099_c0_g1_i3.g12518.t1 | PHI:2189 | MoGIS2 | *Magnaporthe_oryzae* | reduced_virulence | cellular nucleic acid-binding protein | Zinc finger, CCHC-type |
| DN11486_c0_g1_i1.g22387.t1 | PHI:7194 | WISH | *Magnaporthe_oryzae* | reduced_virulence | hypothetical protein BFJ71_g5446 |  |
| DN12051_c0_g1_i1.g25002.t1 | PHI:1180 | (Sc_Sat4) | *Fusarium_graminearum* | reduced_virulence | hypothetical protein BFJ68_g10659 | Protein kinase domain |
| DN12928_c0_g1_i1.g17060.t1 | PHI:179 | PELA | *Nectria_haematococca* | reduced_virulence | pectate lyase E | Pectate lyase PlyH/PlyE-like |
| DN14440_c0_g1_i1.g6409.t1 | PHI:222 | PELB | *Colletotrichum_gloeosporioides* | reduced_virulence | probable pectate lyase 1 | Pectate lyase |
| DN14830_c0_g1_i1.g1783.t1 | PHI:1641__PHI:2434 | FgFlbA | *Fusarium_graminearum* | reduced_virulence | Developmental regulator flbA | RGS domain |
| DN14875_c0_g1_i1.g1799.t1 | PHI:199 | AOX1 | *Passalora_fulva* | reduced_virulence | alcohol oxidase | Glucose-methanol-choline oxidoreductase, C-terminal |
| DN16882_c0_g1_i1.g17133.t1 | PHI:3019 | MoLys2 | *Magnaporthe_oryzae* | reduced_virulence | L-aminoadipate-semialdehyde dehydrogenase | AMP-dependent synthetase/ligase |
| DN1845_c0_g1_i1.g8290.t1 | PHI:2189 | MoGIS2 | *Magnaporthe_oryzae* | reduced_virulence | related to hexamer-binding protein HEXBP | Zinc finger, CCHC-type |
| DN21448_c0_g1_i1.g24394.t1 | PHI:7651 | FgPR-IL-4 | *Fusarium_graminearum* | reduced_virulence | hypothetical protein FOXG_09795 | Golgi-associated plant pathogenesis-related protein 1, SCP domain |
| DN22050_c0_g1_i1.g12132.t1 | PHI:2130 | MoHox4 | *Magnaporthe_oryzae* | reduced_virulence | hypothetical protein BFJ68_g12493 | PTP type protein phosphatase |
| DN240_c0_g1_i7.g53575.t1 | PHI:1347 | GzC2H007 | *Fusarium_graminearum* | reduced_virulence | hypothetical protein FOTG_08741 | Zinc finger C2H2-type |
| DN3037_c0_g1_i1.g494.t1 | PHI:2911 | Ss-pth2 | *Sclerotinia_sclerotiorum* | reduced_virulence | Putative mitochondrial carnitine O-acetyltransferase | Choline/carnitine acyltransferase domain |
| DN3427_c0_g1_i1.g22725.t1 | PHI:4602 | FDB2 | *Fusarium_graminearum* | reduced_virulence | fatty acid synthase subunit beta, fungi type | Fatty acid synthase |
| DN3858_c0_g1_i1.g24643.t1 | PHI:6594 | carA | *Pseudomonas_syringae* | reduced_virulence | Protein pyrABCN | Carbamoyl-phosphate synthase large subunit, CPSase domain |
| DN6092_c0_g1_i1.g4240.t1 | PHI:179 | PELA | *Nectria_haematococca* | reduced_virulence | hypothetical protein BFJ65_g13903 | Pectate lyase PlyH/PlyE-like |
| DN6672_c0_g1_i3.g4829.t1 | PHI:2283 | AreA | *Fusarium_oxysporum* | reduced_virulence | Nitrogen regulatory protein areA | Nitrogen regulatory protein areA, GATA-like domain |
| DN7_c0_g1_i1.g11254.t1 | PHI:356 | HEX1 | *Magnaporthe_oryzae* | reduced_virulence | hypothetical protein BFJ69_g10702 | Hex1, S1 domain |
| DN7808_c0_g1_i2.g20895.t1 | PHI:1917 | GzZC232 | *Fusarium_graminearum* | reduced_virulence | Transcriptional regulatory protein pro-1 | Zn (2)-C6 fungal-type DNA-binding domain |
| DN829_c0_g1_i3.g25626.t1 | PHI:8924 | FgPLD1_(FGSG_09917) | *Fusarium_graminearum* | reduced_virulence | Phospholipase D1 | Phospholipase D/Transphosphatidylase |
| DN8509_c0_g1_i1.g19857.t1 | PHI:3234 | MoLYS20 | *Magnaporthe_oryzae* | reduced_virulence | Homocitrate synthase, mitochondrial | Pyruvate carboxyltransferase |
| DN895_c0_g1_i1.g25451.t1 | PHI:5190 | MoPRX1 | *Magnaporthe_oryzae* | reduced_virulence | probable PRX1-mitochondrial isoform of thioredoxin peroxidase | Alkyl hydroperoxide reductase subunit C/ Thiol specific antioxidant |
| DN912_c0_g1_i1.g4102.t1 | PHI:3945 | XC_2203 | *Xanthomonas_campestris* | reduced_virulence | Nucleoside diphosphate kinase | Nucleoside diphosphate kinase |
| DN930_c1_g1_i1.g3810.t1 | PHI:5471 | MoDac | *Magnaporthe_oryzae* | reduced_virulence | Putative N-acetylglucosamine-6-phosphate deacetylase | N-acetylglucosamine-6-phosphate deacetylase |
| NODE_350.g7094.t1 | PHI:8670 | ACC_deaminase_(VDAG_10392) | *Verticillium_dahliae* | reduced_virulence | putative 1-aminocyclopropane-1-carboxylate deaminase | 1-aminocyclopropane-1-carboxylate deaminase |
| NODE_434.g8073.t1 | PHI:2315 | ChLae1 | *Bipolaris_maydis* | reduced_virulence | hypothetical protein FOXG_13466 | S-adenosyl-L-methionine-dependent methyltransferase |
| NODE_556.g9273.t1 | PHI:2364 | tom1 | *Fusarium_oxysporum* | reduced_virulence | endo-1,4-beta-xylanase C | Glycoside hydrolase family 10 domain |
| NODE_600.g9660.t1 | PHI:1335__PHI:3110__PHI:3808__PHI:4244 | GzbZIP017__FgATF1__Atf1__FgOS-2 | *Fusarium_graminearum* | reduced_virulence | hypothetical protein BFJ71_g13304 | bZIP_ATF2 |
| NODE_158.g4225.t1 | PHI:1200__PHI:4587 | (Gsk3)__Fgk3 | *Fusarium_graminearum* | reduced_virulence | Calcium-dependent protein kinase 4 | Protein kinase domain |
| NODE_208.g5090.t1 | PHI:2099 | Pmc1 | *Magnaporthe_oryzae* | reduced_virulence | Ca2+-transporting ATPase | P-type ATPase, subfamily IIB |
| NODE_592.g9596.t1 | PHI:526__PHI:1071 | GAS1__Gas1 | *Ustilago_maydis* | reduced_virulence | hypothetical protein BFJ71_g12101 | Glycoside hydrolase family 31 |
| NODE_728.g10666.t1 | PHI:300__PHI:2826 | FGB1__Fgb1 | *Fusarium_oxysporum* | reduced_virulence | guanine nucleotide-binding protein subunit beta | WD domain, G-beta repeat |
| NODE_106.g12698.t1 | PHI:6259 | DOHH | *Fusarium_graminearum* | reduced_virulence | Deoxyhypusine hydroxylase | L-lysine intermediate to form hypusine eIF-5A factor |
| NODE_136.g14014.t1 | PHI:3089__PHI:4174 | Fgac1 | *Fusarium_graminearum* | reduced_virulence | Adenylate cyclase | Adenylate cyclase G-alpha binding (cAMP) |
| NODE_234.g5487.t1 | PHI:5450 | gdh2 | *Colletotrichum_gloeosporioides* | reduced_virulence | NAD-specific glutamate dehydrogenase | Glutamate/phenylalanine/leucine/valine dehydrogenase, C-terminal |
| NODE_7.g350.t1 | PHI:8939 | ndh_(PA4538) | *Pseudomonas_aeruginosa* | reduced_virulence | hypothetical protein BFJ66_g8830 | Pyridine nucleotide-disulphide oxidoreductase |
| DN13425_c0_g1_i1.g15307.t1 | PHI:7283 | Pg1 | *Fusarium_graminearum* | reduced_virulence | galacturan 1,4-alpha-galacturonidase | Glycoside hydrolase, family 28 |
| DN6661_c0_g1_i1.g4823.t1 | PHI:7283 | Pg1 | *Fusarium_graminearum* | reduced_virulence | endopolygalacturonase PG2 | Glycoside hydrolase, family 28 |
| NODE_681.g10326.t1 | PHI:7708 | VEDA_05198 | *Verticillium_dahliae* | reduced_virulence | hypothetical protein BFJ71_g9053 | Polyketide synthase, enoylreductase domain |
| NODE_807.g11226.t1 | PHI:7706 | VEDA_05196 | *Verticillium_dahliae* | reduced_virulence | Efflux pump roqT | Major facilitator superfamily |
