# Supplementary material for: Comparative transcriptomic analysis of races 1, 2, 5 and 6 of Fusarium oxysporum f.sp. pisi in a susceptible pea host identifies differential pathogenicity profiles
Source: BMC Genomics. 2021 Oct 9;22:734. doi: 10.1186/s12864-021-08033-y (PMC8502283; doi:10.1186/s12864-021-08033-y)
Supplement: Supplementary file 8 — Additional file 8: Table S6. Differentially expressed genes in R2 that mapped to the virulence-associated genes on the PHI-base database. [file 12864_2021_8033_MOESM8_ESM.docx]

**Supplementary Table 6**

| **Unigene** | **PHI No.** | **Sequence homology to** | **Phytopathogens** | **Gene knockout result** | **Predicted protein** | **Protein domain** |
| --- | --- | --- | --- | --- | --- | --- |
| NODE_136.g4881.t1 | PHI:3216 | MoCDIP4 | *Magnaporthe_oryzae* | effector | hypothetical protein FOTG_13992 | Glycoside hydrolase, family 61 |
| NODE_172.g17916.t1 | PHI:3216 | MoCDIP4 | *Magnaporthe_oryzae* | effector | hypothetical protein BFJ69_g4370 | Glycosyl hydrolase family 61 |
| NODE_234.g19299.t1 | PHI:6831 | Vd4LysM | *Verticillium_dahliae* | effector | uncharacterized protein FMAN_13153 | LysM domain superfamily |
| NODE_253.g19575.t1 | PHI:3216 | MoCDIP4 | *Magnaporthe_oryzae* | effector | hypothetical protein FOCG_13174 | Glycoside hydrolase, family 61 |
| NODE_330.g8363.t1 | PHI:3216 | MoCDIP4 | *Magnaporthe_oryzae* | effector | hypothetical protein BFJ65_g7877 | Glycosyl hydrolase family 61 |
| NODE_388.g9183.t1 | PHI:2216 | PemG1 | *Magnaporthe_oryzae* | effector | hypothetical protein BFJ69_g6142 | Stm1-like, N-terminal |
| NODE_614.g11692.t1 | PHI:3216 | MoCDIP4 | *Magnaporthe_oryzae* | effector | hypothetical protein BFJ69_g6260 | Glycoside hydrolase, family 61 |
| NODE_108.g15256.t1 | PHI:1226 | FGSG_05845 | *Fusarium_graminearum* | lethal | AGC/RSK/RSKP70 protein kinase | Protein kinase domain-AGC-kinase, C-terminal |
| NODE_110.g4217.t1 | PHI:3280 | CoRAS1 | *Colletotrichum_orbiculare* | lethal | Ras-like protein | Small GTPase (Ras family (G)) |
| NODE_188.g18340.t1 | PHI:5271 | fabG1 | *Ralstonia_solanacearum* | lethal | beta-ketoacyl reductase | Enoyl-(Acyl carrier protein) reductase |
| NODE_284.g19913.t1 | PHI:1872 | GzZC187 | *Fusarium_graminearum* | lethal | hypothetical protein FOIG_03484 | Zn (2)-C6 fungal-type DNA-binding domain-GAL4 |
| NODE_456.g9995.t1 | PHI:1226 | FGSG_05845 | *Fusarium_graminearum* | lethal | serine/threonine protein kinase | Protein kinase domain |
| NODE_589.g11443.t1 | PHI:5271 | fabG1 | *Ralstonia_solanacearum* | lethal | hypothetical protein BFJ71_g5962 | Enoyl-(Acyl carrier protein) reductase |
| NODE_725.g12684.t1 | PHI:1450 | GzGATA008 | *Fusarium_graminearum* | lethal | hypothetical protein FOXG_08613 | Zinc finger, GATA-type |
| NODE_918.g14163.t1 | PHI:5271 | fabG1 | *Ralstonia_solanacearum* | lethal | 3-oxoacyl reductase | Enoyl-(Acyl carrier protein) reductase |
| DN308_c0_g1_i3.g15242.t1 | PHI:1976 | GzZC291 | *Fusarium_graminearum* | lethal | hypothetical protein FOMG_01413 | Zn (2)-C6 fungal-type DNA-binding domain-GAL4 |
| DN3508_c0_g1_i1.g15987.t1 | PHI:1546 | GzMyb010 | *Fusarium_graminearum* | lethal | chromatin modification | Helicase/SANT-associated domain |
| NODE_794.g13252.t1 | PHI:4660 | Chs3b | *Fusarium_graminearum* | lethal | chitin synthase | Fungal chitin synthase |
| NODE_106.g4110.t1 | PHI:8928 | MoSCAD2_(MGG_08690) | *Magnaporthe_oryzae* | loss_of_pathogenicity | Uncharacterized protein LW93_10714 | Acyl-CoA oxidase/dehydrogenase, central domain |
| NODE_155.g5271.t1 | PHI:2237 | Rbf1 | *Ustilago_maydis* | loss_of_pathogenicity | hypothetical protein BFJ69_g6108 | Zinc finger C2H2 superfamily |
| NODE_166.g5457.t1 | PHI:4475 | MoTup1 | *Magnaporthe_oryzae* | loss_of_pathogenicity | hypothetical protein FOQG_06594 | WD domain, G-beta repeat |
| NODE_179.g18095.t1 | PHI:4475 | MoTup1 | *Magnaporthe_oryzae* | loss_of_pathogenicity | hypothetical protein CDV36_014435 | WD domain, G-beta repeat |
| NODE_239.g6920.t1 | PHI:874 | MGG_00435 | *Magnaporthe_oryzae* | loss_of_pathogenicity | hypothetical protein FOIG_04191 | integral membrane protein |
| NODE_242.g19429.t1 | PHI:133 | AKT1 | *Alternaria_alternata* | loss_of_pathogenicity | 4-coumarate--CoA ligase-like | AMP-dependent synthetase/ligase |
| NODE_269.g7384.t1 | PHI:2098 | Calcium-transporting_ATPase_3 | *Magnaporthe_oryzae* | loss_of_pathogenicity | sodium/potassium-transporting ATPase | P-type ATPase |
| NODE_274.g7470.t1 | PHI:5232 | MoARG1 | *Magnaporthe_oryzae* | loss_of_pathogenicity | Argininosuccinate synthase | Argininosuccinate synthase |
| NODE_349.g8634.t1 | PHI:2731 | VdSge1 | *Verticillium_dahliae* | loss_of_pathogenicity | Efflux pump roqT | Major facilitator superfamily |
| NODE_379.g9074.t1 | PHI:734 | FOW2 | *Fusarium_oxysporum* | loss_of_pathogenicity | hypothetical protein FOPG_03948 | Zn (2)-C6 fungal-type DNA-binding domain |
| NODE_381.g9097.t1 | PHI:2858 | FvVE1 | *Fusarium_verticillioides* | loss_of_pathogenicity | hypothetical protein FOTG_05490 | Velvet factor |
| NODE_382.g9107.t1 | PHI:9359 | Fgleu4_(FGSG_12952) | *Fusarium_graminearum* | loss_of_pathogenicity | 2-isopropylmalate synthase | 2-isopropylmalate synthase LeuA, allosteric (dimerisation) domain |
| NODE_756.g12930.t1 | PHI:4639 | Cbp1 | *Magnaporthe_oryzae* | loss_of_pathogenicity | hypothetical protein FOXG_02597 | NodB homology domain |
| NODE_965.g14482.t1 | PHI:9357 | Fgleu1_(FGSG_09589) | *Fusarium_graminearum* | loss_of_pathogenicity | Aconitate hydratase | Aconitase/3-isopropylmalate dehydratase large subunit, alpha/beta/alpha domain |
| NODE_967.g14496.t1 | PHI:734 | FOW2 | *Fusarium_oxysporum* | loss_of_pathogenicity | hypothetical protein FOC1_g10011090 | Zn (2)-C6 fungal-type DNA-binding domain |
| DN1451_c0_g1_i6.g31116.t1 | PHI:8928 | MoSCAD2_(MGG_08690) | *Magnaporthe_oryzae* | loss_of_pathogenicity | Acyl-CoA dehydrogenase | Acyl-CoA dehydrogenase/oxidase C-terminal |
| DN1525_c0_g1_i2.g14118.t1 | PHI:4475 | MoTup1 | *Magnaporthe_oryzae* | loss_of_pathogenicity | Guanine nucleotide-binding protein | WD domain, G-beta repeat |
| DN19537_c0_g1_i2.g618.t1 | PHI:7323 | ralA | *Ralstonia_solanacearum* | loss_of_pathogenicity | acyl-CoA synthetase | AMP-dependent synthetase/ligase |
| DN2728_c0_g1_i1.g14680.t1 | PHI:2237 | Rbf1 | *Ustilago_maydis* | loss_of_pathogenicity | hypothetical protein BFJ72_g5893 | Zinc finger C2H2 superfamily |
| DN3395_c0_g1_i2.g16822.t1 | PHI:7331 | Vhb1 | *Verticillium_dahliae* | loss_of_pathogenicity | hypothetical protein FOCG_01077 | Homeobox domain |
| DN3403_c0_g1_i1.g10902.t1 | PHI:734 | FOW2 | *Fusarium_oxysporum* | loss_of_pathogenicity | hypothetical protein BFJ69_g5063 | Transcription factor domain, fungi |
| DN6183_c0_g1_i1.g16926.t1 | PHI:4475 | MoTup1 | *Magnaporthe_oryzae* | loss_of_pathogenicity | hypothetical protein FOTG_00827 | WD domain, G-beta repeat |
| DN868_c0_g1_i1.g16277.t1 | PHI:4475 | MoTup1 | *Magnaporthe_oryzae* | loss_of_pathogenicity | hypothetical protein FOXG_08701 | WD domain, G-beta repeat |
| DN24377_c0_g1_i1.g17952.t1 | PHI:2694 | rsmB | *Pectobacterium_atrosepticum* | loss_of_pathogenicity | 25S rRNA (cytosine(2870)-C(5))-methyltransferase | RNA (C5-cytosine) methyltransferase, NOP2 |
| DN5425_c0_g1_i1.g24486.t1 | PHI:2694 | rsmB | *Pectobacterium_atrosepticum* | loss_of_pathogenicity | hypothetical protein FOTG_07624 | RNA (C5-cytosine) methyltransferase, NOP2 |
| DN6777_c0_g1_i2.g17217.t1 | PHI:2115__PHI:4494 | Annexin_A7__AnnA7 | *Magnaporthe_oryzae* | loss_of_pathogenicity | Annexin A11 | Annexin repeat |
| NODE_604.g11596.t1 | PHI:3126__PHI:9305 | argD | *Erwinia_amylovora* | loss_of_pathogenicity | acetylornithine aminotransferase | Aminotransferase class-III |
| NODE_100.g14760.t1 | PHI:1354 | GzC2H014 | *Fusarium_graminearum* | reduced_virulence | hypothetical protein FOIG_02090 | Zinc finger C2H2 superfamily |
| NODE_103.g14978.t1 | PHI:2189 | MoGIS2 | *Magnaporthe_oryzae* | reduced_virulence | Cellular nucleic acid-binding protein | Zinc finger, CCHC-type superfamily |
| NODE_104.g15006.t1 | PHI:9476 | CoTHR4_(Cob_04177) | *Colletotrichum_orbiculare* | reduced_virulence | Threonine synthase | Threonine synthase, N-terminal |
| NODE_106.g15133.t1 | PHI:6245 | FaMyo2B | *Fusarium_graminearum* | reduced_virulence | Myosin | Myosin head, motor domain |
| NODE_107.g15180.t1 | PHI:7194 | WISH | *Magnaporthe_oryzae* | reduced_virulence | hypothetical protein FOTG_01958 | CFEM domain |
| NODE_108.g15261.t1 | PHI:7788 | AldB_(PSPTO_2673) | *Pseudomonas_syringae* | reduced_virulence | Phenylacetaldehyde dehydrogenase | Aldehyde dehydrogenase domain |
| NODE_110.g15378.t1 | PHI:7788 | AldB_(PSPTO_2673) | *Pseudomonas_syringae* | reduced_virulence | betaine-aldehyde dehydrogenase | Aldehyde dehydrogenase domain |
| NODE_119.g15854.t1 | PHI:2165 | PTH11 | *Magnaporthe_oryzae* | reduced_virulence | hypothetical protein FOXG_15891 | CFEM domain |
| NODE_120.g15890.t1 | PHI:1046 | CTB5 | *Cercospora_nicotianae* | reduced_virulence | hypothetical protein FOXG_08948 | FAD linked oxidase, N-terminal |
| NODE_123.g16030.t1 | PHI:9320 | purL | *Erwinia_amylovora* | reduced_virulence | phosphoribosylformylglycinamidine synthase | Phosphoribosylformylglycinamidine synthase, N-terminal |
| NODE_123.g16035.t1 | PHI:6317 | Hog1 | *Fusarium_oxysporum* | reduced_virulence | CMGC/MAPK/P38 protein kinase | Protein kinase domain-Mitogen-activated protein (MAP) kinase p38-like |
| NODE_124.g4616.t1 | PHI:9266 | FUM6 | *Fusarium_proliferatum* | reduced_virulence | hypothetical protein FOC1_g10006735 | Cytochrome P450 |
| NODE_125.g16134.t1 | PHI:3234 | MoLYS20 | *Magnaporthe_oryzae* | reduced_virulence | Homocitrate synthase | Pyruvate carboxyltransferase |
| NODE_128.g16246.t1 | PHI:179 | PELA | *Nectria_haematococca* | reduced_virulence | pectate lyase F | Pectate lyase PlyH/PlyE-like |
| NODE_128.g16251.t1 | PHI:6262 | FUG1 | *Fusarium_verticillioides* | reduced_virulence | hypothetical protein FOC1_g10011313 | Nitrogen regulatory protein areA, GATA-like domain |
| NODE_128.g4727.t1 | PHI:7672 | FgLetm1 | *Fusarium_graminearum* | reduced_virulence | hypothetical protein FOC4_g10001384 | Letm1 ribosome-binding domain |
| NODE_132.g16461.t1 | PHI:305 | ICL1 | *Magnaporthe_oryzae* | reduced_virulence | isocitrate lyase | Isocitrate lyase |
| NODE_132.g16462.t1 | PHI:784 | MGG_00056 | *Magnaporthe_oryzae* | reduced_virulence | glucose 1-dehydrogenase | Enoyl-(Acyl carrier protein) reductase |
| NODE_132.g4803.t1 | PHI:9282 | mfsG_(BCIN_06g00026) | *Botrytis_cinerea* | reduced_virulence | hypothetical protein FAVG1_06068 | Peptide chain release factor class I |
| NODE_134.g16552.t1 | PHI:132 | ABC1 | *Magnaporthe_oryzae* | reduced_virulence | ABC transporter | ABC transporter-like |
| NODE_134.g4850.t1 | PHI:3914 | Sdh1 | *Parastagonospora_nodorum* | reduced_virulence | succinate-semialdehyde dehydrogenase | Aldehyde dehydrogenase domain |
| NODE_138.g16699.t1 | PHI:5188 | MoHPX1 | *Magnaporthe_oryzae* | reduced_virulence | hypothetical protein FOXG_16634 | Chloroperoxidase |
| NODE_149.g17123.t1 | PHI:2189 | MoGIS2 | *Magnaporthe_oryzae* | reduced_virulence | hexamer-binding protein | Zinc finger, CCHC-type superfamily |
| NODE_149.g17146.t1 | PHI:5016 | Psimpa1 | *Phytophthora_sojae* | reduced_virulence | uncharacterized protein FVRRES_11925 | Importin subunit alpha |
| NODE_150.g5163.t1 | PHI:9282 | mfsG_(BCIN_06g00026) | *Botrytis_cinerea* | reduced_virulence | hypothetical protein FOXG_05893 | Major facilitator superfamily |
| NODE_152.g5190.t1 | PHI:2239 | Spe-Sdh | *Ustilago_maydis* | reduced_virulence | saccharopine dehydrogenase | Saccharopine dehydrogenase, NADP binding domain |
| NODE_153.g17267.t1 | PHI:2432 | FgRgsA | *Fusarium_graminearum* | reduced_virulence | hypothetical protein BFJ71_g14823 | RGS, regulator of G protein signaling domain |
| NODE_153.g5210.t1 | PHI:5353 | Famfs1 | *Fusarium_asiaticum* | reduced_virulence | hypothetical protein FOC1_g10005150 | Major facilitator superfamily |
| NODE_154.g17326.t1 | PHI:443 | CBL1 | *Fusarium_graminearum* | reduced_virulence | cystathionine beta-lyase | Cys/Met metabolism, pyridoxal phosphate-dependent enzyme |
| NODE_158.g17462.t1 | PHI:8646 | FgLDHL2_(FGSG_16220) | *Fusarium_graminearum* | reduced_virulence | cytochrome b5 | Cytochrome b5-like heme/steroid binding domain |
| NODE_162.g5386.t1 | PHI:2207 | endo-1_4-beta-xylanase_[GH10_family] | *Magnaporthe_oryzae* | reduced_virulence | endoglucanase type F | Glycoside hydrolase family 10 domain |
| NODE_170.g17818.t1 | PHI:6179 | AaSSK1 | *Alternaria_alternata* | reduced_virulence | hypothetical protein FOIG_10785 | Protein kinase domain-Histidine kinase |
| NODE_177.g18058.t1 | PHI:9062 | katG2_(FGSG_12369) | *Fusarium_graminearum* | reduced_virulence | catalase-peroxidase | Haem peroxidase |
| NODE_179.g18113.t1 | PHI:2214 | endo-1_4-beta-xylanase_I_[GH10_family] | *Magnaporthe_oryzae* | reduced_virulence | endo-1,4-beta-xylanase A | Glycosyl hydrolase family 11 |
| NODE_181.g5806.t1 | PHI:7281 | PsINV | *Puccinia_striiformis* | reduced_virulence | beta-fructofuranosidase | Glycosyl hydrolase family 32, C-terminal |
| NODE_188.g18354.t1 | PHI:7651 | FgPR-IL-4 | *Fusarium_graminearum* | reduced_virulence | hypothetical protein FOXG_09795 | SCP / Tpx-1 / Ag5 / PR-1 / Sc7 family of extracellular domains. |
| NODE_191.g18439.t1 | PHI:1366 | GzCON7 | *Fusarium_graminearum* | reduced_virulence | hypothetical protein FOIG_04164 | KR domain |
| NODE_194.g6099.t1 | PHI:1556 | GzNH001 | *Fusarium_graminearum* | reduced_virulence | hypothetical protein FOMG_06736 | Zinc finger, GATA-type |
| NODE_202.g18699.t1 | PHI:9250 | PsRPs26 | *Puccinia_striiformis* | reduced_virulence | hypothetical protein FPSE_06056 | Ribosomal protein S26e |
| NODE_211.g18893.t1 | PHI:1090__PHI:1163__PHI:3893 | FGSG_10057__ERB1__FgEBR1 | *Fusarium_graminearum* | reduced_virulence | hypothetical protein FOC1_g10012848 | Zn (2)-C6 fungal-type DNA-binding domain-GAL4 |
| NODE_213.g18917.t1 | PHI:1403 | GzC2H066 | *Fusarium_graminearum* | reduced_virulence | hypothetical protein FSPOR_8584 | Zinc finger C2H2 superfamily |
| NODE_226.g19140.t1 | PHI:2117 | SPM1 | *Magnaporthe_oryzae* | reduced_virulence | endopeptidase | Peptidase S8/S53 domain |
| NODE_229.g6704.t1 | PHI:6928 | MoBRE1 | *Magnaporthe_oryzae* | reduced_virulence | E3 ubiquitin-protein ligase | BRE1 E3 ubiquitin ligase |
| NODE_236.g19322.t1 | PHI:2964 | AsnB | *Xanthomonas_oryzae* | reduced_virulence | asparagine synthase | Asparagine synthase, glutamine-hydrolyzing |
| NODE_239.g19391.t1 | PHI:4978 | BcFRQ1 | *Botrytis_cinerea* | reduced_virulence | Frequency clock protein | Frequency clock protein |
| NODE_243.g19451.t1 | PHI:7741 | FreB | *Verticillium_dahliae* | reduced_virulence | hypothetical protein FOQG_16413 | Ferric reductase, NAD binding domain |
| NODE_268.g7375.t1 | PHI:2409 | FgMT2 | *Fusarium_graminearum* | reduced_virulence | cyclopropane-fatty-acyl-phospholipid synthase | Mycolic acid cyclopropane synthase |
| NODE_274.g7477.t1 | PHI:5185 | MoAPX1 | *Magnaporthe_oryzae* | reduced_virulence | hypothetical protein FOQG_12067 | Haem peroxidase |
| NODE_279.g19872.t1 | PHI:737__PHI:2329 | CTB4 | *Cercospora_nicotianae* | reduced_virulence | hypothetical protein FOC4_g10009741 | Major facilitator superfamily |
| NODE_282.g7615.t1 | PHI:2839 | RED1 | *Bipolaris_maydis* | reduced_virulence | hypothetical protein FVEG_11599 | Polyketide synthase, enoylreductase domain |
| NODE_285.g7654.t1 | PHI:5236 | Dnj1 | *Fusarium_oxysporum* | reduced_virulence | hypothetical protein BFJ69_g6102 | Tetratricopeptide repeat |
| NODE_287.g7680.t1 | PHI:2034 | MFP1 | *Magnaporthe_oryzae* | reduced_virulence | hypothetical protein FOC4_g10001177 | Short-chain dehydrogenase/reductase SDR |
| NODE_291.g7754.t1 | PHI:9353 | leu2A_(MGG_05223) | *Magnaporthe_oryzae* | reduced_virulence | isocitrate dehydrogenase | Isopropylmalate dehydrogenase-like domain |
| NODE_300.g7946.t1 | PHI:881 | MGG_04556 | *Magnaporthe_oryzae* | reduced_virulence | hypothetical protein FOXG_04143 | Polyketide synthase, enoylreductase domain |
| NODE_306.g8027.t1 | PHI:2920__PHI:9074 | FET3-1__fet3-1 | *Colletotrichum_graminicola* | reduced_virulence | Iron transport multicopper oxidase | Multicopper oxidase, type 2 |
| NODE_316.g20173.t1 | PHI:877 | MGG_00383 | *Magnaporthe_oryzae* | reduced_virulence | S-adenosylmethionine synthase | S-adenosylmethionine synthetase, N-terminal |
| NODE_317.g8197.t1 | PHI:1793 | GzZC108 | *Fusarium_graminearum* | reduced_virulence | hypothetical protein FOQG_01843 | Zn (2)-C6 fungal-type DNA-binding domain |
| NODE_337.g20294.t1 | PHI:4602 | FDB2 | *Fusarium_graminearum* | reduced_virulence | Fatty acid synthase subunit beta | Fatty acid synthase |
| NODE_345.g8595.t1 | PHI:2289 | BcBOA2 | *Botrytis_cinerea* | reduced_virulence | FAD-containing monooxygenase | Flavin monooxygenase-like |
| NODE_377.g9040.t1 | PHI:3816 | GSN1 | *Magnaporthe_oryzae* | reduced_virulence | glycogen synthase | Glycogen synthase |
| NODE_384.g9135.t1 | PHI:5480 | MoPyr5 | *Magnaporthe_oryzae* | reduced_virulence | orotate phosphoribosyltransferase | Phosphoribosyltransferase domain |
| NODE_394.g9258.t1 | PHI:2239 | Spe-Sdh | *Ustilago_maydis* | reduced_virulence | spermidine synthase | Spermidine/spermine synthases |
| NODE_439.g9794.t1 | PHI:179 | PELA | *Nectria_haematococca* | reduced_virulence | pectate lyase | Pectate lyase PlyH/PlyE-like |
| NODE_444.g9860.t1 | PHI:1217 | FGSG_00792 | *Fusarium_graminearum* | reduced_virulence | CAMK/CAMKL/KIN4 protein kinase | Protein kinase domain-CAMK/CAMKL/KIN4 protein kinase |
| NODE_464.g10071.t1 | PHI:6409 | FgCdc11 | *Fusarium_graminearum* | reduced_virulence | hypothetical protein FOXG_06069 | guanine nucleotide-binding (GTP) domain (Septin-type) |
| NODE_465.g10085.t1 | PHI:158 | UKC1 | *Ustilago_maydis* | reduced_virulence | hypothetical protein FPOA_00733 | Protein kinase domain- AGC |
| NODE_468.g10114.t1 | PHI:9104 | FGRRES_16221 | *Fusarium_graminearum* | reduced_virulence | hypothetical protein FOXG_13475 |  |
| NODE_468.g10115.t1 | PHI:9144 | FgAP2s_(FGSG_02015) | *Fusarium_graminearum* | reduced_virulence | hypothetical protein BFJ69_g6821 | Carboxylesterase, type B |
| NODE_476.g10224.t1 | PHI:2351__PHI:2353 | AMT1 | *Fusarium_graminearum* | reduced_virulence | arginine N-methyltransferase | S-adenosyl-L-methionine-dependent methyltransferase |
| NODE_485.g10337.t1 | PHI:7173 | HiC-15 | *Verticillium_dahliae* | reduced_virulence | Pisatin demethylase | Cytochrome P450 |
| NODE_498.g10461.t1 | PHI:1422 | GzC2H090 | *Fusarium_graminearum* | reduced_virulence | hypothetical protein BFJ69_g14025 | Dual specificity protein phosphatase domain |
| NODE_501.g10493.t1 | PHI:443 | CBL1 | *Fusarium_graminearum* | reduced_virulence | hypothetical protein BFJ68_g16085 | Cys/Met metabolism, pyridoxal phosphate-dependent enzyme |
| NODE_516.g10687.t1 | PHI:6739 | MoGls2 | *Magnaporthe_oryzae* | reduced_virulence | Alpha-xylosidase | Glycoside hydrolase family 31 |
| NODE_520.g10726.t1 | PHI:8695 | MoImd4_(MGG_03699) | *Magnaporthe_oryzae* | reduced_virulence | hypothetical protein FAVG1_01712 | IMP dehydrogenase/GMP reductase |
| NODE_524.g10773.t1 | PHI:5068 | MCC | *Fusarium_graminearum* | reduced_virulence | acetyl-CoA carboxylase | Acetyl-CoA carboxylase, central domain |
| NODE_559.g11137.t1 | PHI:2208 | endo-1_4-beta-xylanase_[GH10_family] | *Magnaporthe_oryzae* | reduced_virulence | Endo-1,4-beta-xylanase | Glycoside hydrolase family 10 domain |
| NODE_568.g11237.t1 | PHI:3308 | cnf1 | *Magnaporthe_oryzae* | reduced_virulence | hypothetical protein FOTG_09210 | Zn (2)-C6 fungal-type DNA-binding domain superfamily |
| NODE_589.g11441.t1 | PHI:382__PHI:2233 | SMU1__Smu1 | *Ustilago_maydis* | reduced_virulence | STE/STE20/PAKA protein kinase | Protein kinase domain |
| NODE_589.g11452.t1 | PHI:5082 | MoAcat1 | *Magnaporthe_oryzae* | reduced_virulence | 3-ketoacyl-CoA thiolase | acetyl-coenzyme A acetyltransferases (Thiolases) |
| NODE_618.g20824.t1 | PHI:4602 | FDB2 | *Fusarium_graminearum* | reduced_virulence | hypothetical protein FLONG3_2226 | Fatty acid synthase, meander beta sheet domain |
| NODE_636.g11917.t1 | PHI:367 | MRB1 | *Ustilago_maydis* | reduced_virulence | hypothetical protein FOXG_01767 | Mitochondrial glycoprotein |
| NODE_637.g11924.t1 | PHI:2058 | LHS1 | *Magnaporthe_oryzae* | reduced_virulence | heat shock protein 70 | Heat shock protein 70 family |
| NODE_650.g12039.t1 | PHI:7142 | FvStp1 | *Fusarium_verticillioides* | reduced_virulence | hypothetical protein FOTG_00377 | Far11/STRP, N-terminal |
| NODE_660.g12144.t1 | PHI:3315 | conx1 | *Magnaporthe_oryzae* | reduced_virulence | hypothetical protein FOTG_07898 | PAS domain |
| NODE_663.g12166.t1 | PHI:7134 | MoRab5B | *Magnaporthe_oryzae* | reduced_virulence | GTP-binding nuclear protein | Small GTPase |
| NODE_668.g12213.t1 | PHI:1188 | (Sc_Sak1) | *Fusarium_graminearum* | reduced_virulence | CAMKK protein kinase | Protein kinase domain-CaMKK |
| NODE_683.g12357.t1 | PHI:9055 | MoIVD_(MGG_02540) | *Magnaporthe_oryzae* | reduced_virulence | isovaleryl-CoA dehydrogenase | Acyl-CoA dehydrogenase/oxidase C-terminal |
| NODE_687.g12394.t1 | PHI:816 | MGG_04582 | *Magnaporthe_oryzae* | reduced_virulence | beta-glucosidase btgE | Glycoside hydrolase superfamily |
| NODE_706.g12543.t1 | PHI:4914 | ZtCBR1 | *Zymoseptoria_tritici* | reduced_virulence | hypothetical protein BFJ69_g14665 | NADH:cytochrome b5 reductase-like |
| NODE_729.g12708.t1 | PHI:2143 | MoVPR | *Magnaporthe_oryzae* | reduced_virulence | 4-aminobutyrate aminotransferase | Aminotransferase class-III |
| NODE_748.g12869.t1 | PHI:2839 | RED1 | *Bipolaris_maydis* | reduced_virulence | hypothetical protein FOMG_02164 | Polyketide synthase, enoylreductase domain |
| NODE_750.g12885.t1 | PHI:2095 | Calcium-transporting_ATPase_3 | *Magnaporthe_oryzae* | reduced_virulence | potassium/sodium efflux P-type ATPase | Cation-transporting P-type ATPase, C-terminal |
| NODE_756.g12927.t1 | PHI:4658 | FgHXK1 | *Fusarium_graminearum* | reduced_virulence | hypothetical protein FOQG_15132 | Hexokinase, N-terminal |
| NODE_757.g12934.t1 | PHI:1291 | TOP1 | *Fusarium_graminearum* | reduced_virulence | DNA topoisomerase 1 | DNA topoisomerase I, DNA binding, eukaryotic type |
| NODE_758.g12942.t1 | PHI:5187 | MoCCP1 | *Magnaporthe_oryzae* | reduced_virulence | cytochrome c peroxidase | Haem peroxidase |
| NODE_767.g13013.t1 | PHI:744 | ADE5 | *Fusarium_graminearum* | reduced_virulence | hypothetical protein FOXG_10535 | Phosphoribosylglycinamide synthetase |
| NODE_801.g13294.t1 | PHI:179 | PELA | *Nectria_haematococca* | reduced_virulence | pectate lyase E | Pectate lyase PlyH/PlyE-like |
| NODE_818.g13434.t1 | PHI:784 | MGG_00056 | *Magnaporthe_oryzae* | reduced_virulence | hypothetical protein BFJ69_g6264 | Short-chain dehydrogenase/reductase SDR |
| NODE_834.g13591.t1 | PHI:3019 | MoLys2 | *Magnaporthe_oryzae* | reduced_virulence | L-2-aminoadipate reductase | AMP-dependent synthetase/ligase |
| NODE_844.g13663.t1 | PHI:8646 | FgLDHL2_(FGSG_16220) | *Fusarium_graminearum* | reduced_virulence | lactate dehydrogenase | Cytochrome b5-like heme/steroid binding domain |
| NODE_884.g13911.t1 | PHI:1379 | GzC2H042 | *Fusarium_graminearum* | reduced_virulence | hypothetical protein FOC1_g10005228 | ZN622/Rei1/Reh1, zinc finger C2H2-type |
| NODE_898.g14012.t1 | PHI:7975 | Nop53 | *Botrytis_cinerea* | reduced_virulence | hypothetical protein BFJ70_g11791 | Ribosome biogenesis protein Nop53/GLTSCR2 |
| NODE_901.g14041.t1 | PHI:5826 | GIV4 | *Fusarium_graminearum* | reduced_virulence | hypothetical protein FOC1_g10003508 | integral membrane protein |
| NODE_935.g14284.t1 | PHI:4509 | Ss-odc2 | *Sclerotinia_sclerotiorum* | reduced_virulence | Uncharacterized protein LW93_9471 | Cupin 1 |
| NODE_942.g14333.t1 | PHI:2976 | CgOPT1 | *Colletotrichum_gloeosporioides* | reduced_virulence | Oligopeptide transporter | Oligopeptide transporter, OPT superfamily |
| NODE_959.g14441.t1 | PHI:3945 | XC_2203 | *Xanthomonas_campestris* | reduced_virulence | Nucleoside diphosphate kinase | Nucleoside diphosphate kinase |
| NODE_970.g14520.t1 | PHI:5472 | MoDeam | *Magnaporthe_oryzae* | reduced_virulence | Glucosamine-6-phosphate isomerase | Glucosamine/galactosamine-6-phosphate isomerase |
| NODE_970.g14522.t1 | PHI:5471 | MoDac | *Magnaporthe_oryzae* | reduced_virulence | N-acetylglucosamine-6-phosphate deacetylase | N-acetylglucosamine-6-phosphate deacetylase |
| NODE_977.g14566.t1 | PHI:1093 | FGSG_02077 | *Fusarium_graminearum* | reduced_virulence | uncharacterized protein FMAN_11213 | CFEM domain |
| NODE_990.g14663.t1 | PHI:7999 | putA | *Pseudomonas_aeruginosa* | reduced_virulence | Delta-1-pyrroline-5-carboxylate dehydrogenase | Aldehyde dehydrogenase domain |
| DN10125_c0_g1_i1.g33827.t1 | PHI:2117 | SPM1 | *Magnaporthe_oryzae* | reduced_virulence | hypothetical protein FOCG_16954 | Peptidase S8/S53 domain superfamily |
| DN1064_c0_g1_i1.g11752.t1 | PHI:9222 | FgPEX1_(FGSG_07104) | *Fusarium_graminearum* | reduced_virulence | hypothetical protein FOTG_06965 | ATPase, AAA-type |
| DN1184_c0_g1_i1.g34723.t1 | PHI:737__PHI:2329 | CTB4 | *Cercospora_nicotianae* | reduced_virulence | hypothetical protein FOXG_04752 | Major facilitator superfamily |
| DN1201_c0_g1_i3.g17355.t1 | PHI:9408 | VdSkn7_(VDAG_02250) | *Verticillium_dahliae* | reduced_virulence | hypothetical protein FOXG_08113 | Signal transduction response regulator, receiver domain |
| DN13233_c0_g1_i1.g3129.t1 | PHI:3697__PHI:3925 | FgABC1__FgABC3 | *Fusarium_graminearum* | reduced_virulence | ATPase | ABC-2 type transporter |
| DN1365_c0_g1_i3.g5733.t1 | PHI:9056 | FgTRR_(FGSG_00871) | *Fusarium_graminearum* | reduced_virulence | thioredoxin reductase | Pyridine nucleotide-disulphide oxidoreductase |
| DN13897_c0_g1_i1.g6245.t1 | PHI:2839 | RED1 | *Bipolaris_maydis* | reduced_virulence | hypothetical protein BFJ68_g8178 | NAD(P)-binding domain superfamily |
| DN1521_c0_g1_i3.g1970.t1 | PHI:6408__PHI:6603 | FgCdc3__FaCdc3_(FGSG_05315.3) | *Fusarium_graminearum__Fusarium_asiaticum* | reduced_virulence | hypothetical protein FPSE_02938 | Guanine nucleotide-binding (G) domain (Septin-type) |
| DN15882_c0_g1_i1.g17600.t1 | PHI:1641__PHI:2434 | FgFlbA | *Fusarium_graminearum* | reduced_virulence | regulator of G protein signaling | RGS, regulator of G protein signaling domain |
| DN16496_c0_g1_i1.g8055.t1 | PHI:179 | PELA | *Nectria_haematococca* | reduced_virulence | hypothetical protein BFJ65_g13903 | Pectate lyase PlyH/PlyE-like |
| DN19908_c0_g1_i1.g17503.t1 | PHI:8924 | FgPLD1_(FGSG_09917) | *Fusarium_graminearum* | reduced_virulence | phospholipase D | Phospholipase D/Transphosphatidylase |
| DN20848_c0_g1_i1.g28679.t1 | PHI:180 | PELD | *Nectria_haematococca* | reduced_virulence | Pectate lyase D | Pectate lyase PlyH/PlyE-like |
| DN213_c0_g2_i1.g10793.t1 | PHI:6261 | PsAAT3 | *Phytophthora_sojae* | reduced_virulence | aspartate aminotransferase | Aminotransferase, class I/classII |
| DN2340_c0_g1_i1.g3912.t1 | PHI:9308 | glmS | *Erwinia_amylovora* | reduced_virulence | glucosamine--fructose-6-phosphate transaminase | Sugar isomerase (SIS) |
| DN23785_c0_g1_i1.g11082.t1 | PHI:2240__PHI:4586 | Srt1 | *Ustilago_maydis* | reduced_virulence | Maltose permease | Major facilitator, sugar transporter-like |
| DN25373_c0_g1_i1.g18002.t1 | PHI:9144 | FgAP2s_(FGSG_02015) | *Fusarium_graminearum* | reduced_virulence | Para-nitrobenzyl esterase | Carboxylesterase, type B |
| DN2784_c0_g1_i2.g16586.t1 | PHI:1078 | Mes1 | *Fusarium_graminearum* | reduced_virulence | hypothetical protein FOTG_01069 | Arf3-interacting protein 1, N-terminal domain |
| DN290_c0_g1_i9.g9764.t1 | PHI:5236 | Dnj1 | *Fusarium_oxysporum* | reduced_virulence | hypothetical protein FOPG_11896 | Heat shock protein DnaJ, cysteine-rich domain |
| DN4217_c0_g1_i1.g14839.t1 | PHI:7788 | AldB_(PSPTO_2673) | *Pseudomonas_syringae* | reduced_virulence | Aldehyde dehydrogenase | Aldehyde dehydrogenase domain |
| DN53_c0_g1_i3.g8821.t1 | PHI:1793 | GzZC108 | *Fusarium_graminearum* | reduced_virulence | hypothetical protein BFJ69_g2667 | Zn (2)-C6 fungal-type DNA-binding domain-GAL4 |
| DN53_c0_g1_i4.g8819.t1 | PHI:3019 | MoLys2 | *Magnaporthe_oryzae* | reduced_virulence | L-aminoadipate-semialdehyde dehydrogenase | Amino acid adenylation domain |
| DN5496_c0_g1_i2.g3026.t1 | PHI:9266 | FUM6 | *Fusarium_proliferatum* | reduced_virulence | Bifunctional cytochrome P450/NADPH--P450 reductase | Cytochrome P450 |
| DN5860_c0_g1_i1.g18735.t1 | PHI:9055 | MoIVD_(MGG_02540) | *Magnaporthe_oryzae* | reduced_virulence | hypothetical protein BFJ69_g6500 | Acyl-CoA dehydrogenase/oxidase C-terminal |
| DN7065_c0_g1_i6.g2427.t1 | PHI:9317 | purF | *Erwinia_amylovora* | reduced_virulence | Amidophosphoribosyltransferase | Glutamine amidotransferase type 2 domain |
| DN7571_c0_g1_i2.g9423.t1 | PHI:7194 | WISH | *Magnaporthe_oryzae* | reduced_virulence | hypothetical protein FOC1_g10004629 | CFEM domain |
| DN793_c0_g1_i1.g15501.t1 | PHI:4491 | SHM | *Magnaporthe_oryzae* | reduced_virulence | Serine hydroxymethyltransferase | Serine hydroxymethyltransferase |
| DN802_c0_g1_i6.g22965.t1 | PHI:7562 | CAL1 | *Alternaria_alternata* | reduced_virulence | phosphoprotein phosphatase | Tetratricopeptide repeat |
| DN8090_c0_g1_i1.g12385.t1 | PHI:3315 | conx1 | *Magnaporthe_oryzae* | reduced_virulence | hypothetical protein FOC1_g10010125 | PAS domain |
| DN940_c0_g1_i1.g32092.t1 | PHI:8924 | FgPLD1_(FGSG_09917) | *Fusarium_graminearum* | reduced_virulence | Phospholipase D1 | Phospholipase D/Transphosphatidylase |
| DN992_c0_g1_i1.g6532.t1 | PHI:1090__PHI:1163__PHI:3893 | FGSG_10057__ERB1__FgEBR1 | *Fusarium_graminearum* | reduced_virulence | hypothetical protein FOC4_g10013861 | Zn (2)-C6 fungal-type DNA-binding domain-GAL4 |
| NODE_820.g13450.t1 | PHI:2315 | ChLae1 | *Bipolaris_maydis* | reduced_virulence | hypothetical protein FOC1_g10009903 | S-adenosyl-L-methionine-dependent methyltransferase |
| NODE_93.g3709.t1 | PHI:2315 | ChLae1 | *Bipolaris_maydis* | reduced_virulence | hypothetical protein FOC1_g10008850 | Methyltransferase domain |
| DN1260_c0_g1_i4.g7767.t1 | PHI:2315 | ChLae1 | *Bipolaris_maydis* | reduced_virulence | hypothetical protein FOXG_10681 | S-adenosyl-L-methionine-dependent methyltransferase |
| NODE_212.g6413.t1 | PHI:2202__PHI:7327 | PdeH__MopdeH | *Magnaporthe_oryzae* | reduced_virulence | hypothetical protein BFJ69_g12817 | 3'5'-cyclic nucleotide phosphodiesterase, catalytic domain |
| DN20343_c0_g1_i1.g13963.t1 | PHI:2099 | Pmc1 | *Magnaporthe_oryzae* | reduced_virulence | Calcium-transporting ATPase 2 | P-type ATPase, cytoplasmic domain N |
| NODE_155.g17355.t1 | PHI:6259 | DOHH | *Fusarium_graminearum* | reduced_virulence | Deoxyhypusine hydroxylase | Deoxyhypusine hydroxylase |
| NODE_287.g7685.t1 | PHI:9245 | Fghyd5_(FGSG_01831) | *Fusarium_graminearum* | reduced_virulence | hydrophobin | Cerato-ulmin hydrophobin family |
| NODE_415.g9525.t1 | PHI:7283 | Pg1 | *Fusarium_graminearum* | reduced_virulence | endopolygalacturonase | Glycosyl hydrolases family 28 |
| NODE_841.g13643.t1 | PHI:7283 | Pg1 | *Fusarium_graminearum* | reduced_virulence | 1,4-alpha-galacturonidase | Glycoside hydrolase, family 28 |
| DN1610_c0_g1_i3.g26909.t1 | PHI:7704 | VEDA_05194 | *Verticillium_dahliae* | reduced_virulence | hypothetical protein FOQG_12898 | NmrA-like domain |
| NODE_153.g17268.t1 | PHI:7708 | VEDA_05198 | *Verticillium_dahliae* | reduced_virulence | hypothetical protein FOMG_10877 | Polyketide synthase, enoylreductase domain |
| NODE_948.g14367.t1 | PHI:7708 | VEDA_05198 | *Verticillium_dahliae* | reduced_virulence | hypothetical protein FOC1_g10009461 | Polyketide synthase, enoylreductase domain |
| DN3846_c0_g1_i1.g4083.t1 | PHI:548__PHI:2305 | BcPIC5__BcFKBP12 | *Botrytis_cinerea* | reduced_virulence | prolyl cis-trans isomerase | FKBP-type peptidyl-prolyl cis-trans isomerase domain |
