# Supplementary material for: Comparative transcriptomic analysis of races 1, 2, 5 and 6 of Fusarium oxysporum f.sp. pisi in a susceptible pea host identifies differential pathogenicity profiles
Source: BMC Genomics. 2021 Oct 9;22:734. doi: 10.1186/s12864-021-08033-y (PMC8502283; doi:10.1186/s12864-021-08033-y)
Supplement: Supplementary file 9 — Additional file 9: Table S7. Differentially expressed genes in R5 that mapped to the virulence-associated genes on the PHI-base database. [file 12864_2021_8033_MOESM9_ESM.docx]

**Supplementary Table 7**

| **Unigenes** | **PHI No.** | **Sequence homology to** | **Phytopathogens** | **Gene knockout result** | **Predicted protein** | **Protein domain** |
| --- | --- | --- | --- | --- | --- | --- |
| NODE_104.g15115.t1 | PHI:2975 | ipx10 | *Pseudomonas_syringae* | effector | LysM peptidoglycan-binding domain-containing protein | Transglycosylase SLT domain 1 |
| NODE_154.g9633.t1 | PHI:1650 | GzWing022 | *Fusarium_graminearum* | lethal | hypothetical protein FOTG_02891 | Fork head domain |
| NODE_233.g11578.t1 | PHI:1546 | GzMyb010 | *Fusarium_graminearum* | lethal | chromatin modification-like protein VID21 | Myb-like DNA-binding domain |
| NODE_618.g14560.t1 | PHI:3280 | CoRAS1 | *Colletotrichum_orbiculare* | lethal | Ras-like protein | GTPase (Ras type) |
| NODE_62.g5622.t1 | PHI:1226 | FGSG_05845 | *Fusarium_graminearum* | lethal | AGC/RSK/RSKP70 protein kinase | Protein kinase domain AGC-kinase, C-terminal |
| NODE_63.g5636.t1 | PHI:1226 | FGSG_05845 | *Fusarium_graminearum* | lethal | related to serine/threonine protein kinase | Protein kinase domain |
| NODE_66.g5822.t1 | PHI:1235 | FGSG_08731 | *Fusarium_graminearum* | lethal | CK1/CK1/CK1-D protein kinase | Protein kinase domain |
| NODE_9.g1253.t1 | PHI:1228 | FGSG_06502 | *Fusarium_graminearum* | lethal | RIO kinase 1 | Protein kinase-like domain-RIO kinase |
| DN1004_c0_g1_i5.g38759.t1 | PHI:1650 | GzWing022 | *Fusarium_graminearum* | lethal | hypothetical protein FOTG_02891 | Fork head domain |
| DN14012_c0_g1_i1.g7831.t1 | PHI:1226 | FGSG_05845 | *Fusarium_graminearum* | lethal | related to serine/threonine protein kinase | Protein kinase domain |
| DN1468_c0_g1_i6.g700.t1 | PHI:1450 | GzGATA008 | *Fusarium_graminearum* | lethal | hypothetical protein FOXG_08613 | Zinc finger, GATA-type |
| DN18411_c0_g1_i1.g10312.t1 | PHI:5271 | fabG1 | *Ralstonia_solanacearum* | lethal | hypothetical protein BFJ71_g5962 | Enoyl-(Acyl carrier protein) reductase |
| DN197_c0_g1_i2.g8746.t1 | PHI:1517 | GzHOMEL013 | *Fusarium_graminearum* | lethal | hypothetical protein FOTG_02327 | Phosphatidate cytidylyltransferase |
| DN3600_c0_g1_i1.g49894.t1 | PHI:1546 | GzMyb010 | *Fusarium_graminearum* | lethal | chromatin modification-like protein VID21 | Helicase/SANT-associated domain |
| DN938_c0_g1_i1.g14801.t1 | PHI:1235 | FGSG_08731 | *Fusarium_graminearum* | lethal | CK1/CK1/CK1-D protein kinase | Protein kinase domain |
| DN938_c0_g1_i6.g14802.t1 | PHI:1235 | FGSG_08731 | *Fusarium_graminearum* | lethal | hypothetical protein FPOA_04401 | Protein kinase domain |
| NODE_102.g7716.t1 | PHI:8884 | aroG1 | *Ralstonia_solanacearum* | loss_of_pathogenicity | Phospho-2-dehydro-3-deoxyheptonate aldolase, tyrosine-inhibited | DAHP synthetase I/KDSA |
| NODE_121.g8531.t1 | PHI:4750 | ChPMA2 | *Colletotrichum_higginsianum* | loss_of_pathogenicity | plasma membrane ATPase | Cation-transporting P-type ATPase, N-terminal |
| NODE_122.g8573.t1 | PHI:3161 | Mohik5 | *Magnaporthe_oryzae* | loss_of_pathogenicity | hypothetical protein FOTG_10371 | Protein kinase domain-Histidine kinase |
| NODE_128.g8779.t1 | PHI:9357 | Fgleu1_(FGSG_09589) | *Fusarium_graminearum* | loss_of_pathogenicity | aconitate hydratase, mitochondrial | Aconitase/3-isopropylmalate dehydratase large subunit, alpha/beta/alpha domain |
| NODE_146.g9429.t1 | PHI:3162 | Mohik8 | *Magnaporthe_oryzae* | loss_of_pathogenicity | hypothetical protein FOTG_06316 | Protein kinase domain-Histidine kinase/HSP90-like ATPase |
| NODE_153.g9611.t1 | PHI:8734 | MoLeu1_(MGG_01553)__leu1_(MGG_01553) | *Magnaporthe_oryzae* | loss_of_pathogenicity | homoaconitase, mitochondrial | Aconitase/3-isopropylmalate dehydratase large subunit, alpha/beta/alpha domain |
| NODE_156.g9695.t1 | PHI:4475 | MoTup1 | *Magnaporthe_oryzae* | loss_of_pathogenicity | hypothetical protein FOTG_00827 | WD domain, G-beta repeat |
| NODE_196.g10754.t1 | PHI:9359 | Fgleu4_(FGSG_12952) | *Fusarium_graminearum* | loss_of_pathogenicity | 2-isopropylmalate synthase | 2-isopropylmalate synthase LeuA, allosteric (dimerisation) domain |
| NODE_21.g2558.t1 | PHI:2145__PHI:9180 | MoSSADH__MoSSADH_(MGG_01230) | *Magnaporthe_oryzae* | loss_of_pathogenicity | succinate-semialdehyde dehydrogenase | Aldehyde dehydrogenase, C-terminal |
| NODE_210.g11102.t1 | PHI:365__PHI:2267 | MLS1__Mls1 | *Parastagonospora_nodorum* | loss_of_pathogenicity | Malate synthase, glyoxysomal | Malate synthase |
| NODE_238.g11673.t1 | PHI:9357 | Fgleu1_(FGSG_09589) | *Fusarium_graminearum* | loss_of_pathogenicity | Aconitate hydratase | Aconitase/3-isopropylmalate dehydratase large subunit, alpha/beta/alpha domain |
| NODE_250.g11888.t1 | PHI:734 | FOW2 | *Fusarium_oxysporum* | loss_of_pathogenicity | hypothetical protein FOC1_g10011090 | Zn (2)-C6 fungal-type DNA-binding domain-GAL4 |
| NODE_261.g12055.t1 | PHI:4475 | MoTup1 | *Magnaporthe_oryzae* | loss_of_pathogenicity | Periodic tryptophan protein 2 | WD domain, G-beta repeat |
| NODE_268.g12184.t1 | PHI:4475 | MoTup1 | *Magnaporthe_oryzae* | loss_of_pathogenicity | hypothetical protein FOTG_02856 | U3 small nucleolar RNA-associated protein 15 |
| NODE_327.g12972.t1 | PHI:5232 | MoARG1 | *Magnaporthe_oryzae* | loss_of_pathogenicity | Argininosuccinate synthase | Argininosuccinate synthase |
| NODE_34.g3697.t1 | PHI:4475 | MoTup1 | *Magnaporthe_oryzae* | loss_of_pathogenicity | hypothetical protein XA68_10480 | WD domain, G-beta repeat |
| NODE_422.g13801.t1 | PHI:9357 | Fgleu1_(FGSG_09589) | *Fusarium_graminearum* | loss_of_pathogenicity | 3-isopropylmalate dehydratase | Aconitase/3-isopropylmalate dehydratase large subunit, alpha/beta/alpha domain |
| NODE_44.g4431.t1 | PHI:268 | MST12 | *Magnaporthe_oryzae* | loss_of_pathogenicity | DNA-binding protein creA | Zinc finger C2H2-type |
| NODE_47.g4692.t1 | PHI:685__PHI:2022 | BUF__BUF1 | *Magnaporthe_oryzae* | loss_of_pathogenicity | hypothetical protein FOTG_03263 | Short-chain dehydrogenase/reductase, conserved site |
| NODE_52.g4941.t1 | PHI:3162 | Mohik8 | *Magnaporthe_oryzae* | loss_of_pathogenicity | hypothetical protein FOTG_01372 | Protein kinase domain histidine kinase |
| NODE_562.g14432.t1 | PHI:3975 | MoIlv2 | *Magnaporthe_oryzae* | loss_of_pathogenicity | acetolactate synthase I/II/III large subunit | Thiamine pyrophosphate enzyme, central domain |
| NODE_63.g5661.t1 | PHI:407 | PBC1 | *Pyrenopeziza_brassicae* | loss_of_pathogenicity | cutinase | Cutinase |
| NODE_87.g6997.t1 | PHI:508 | AFT1 | *Alternaria_alternata* | loss_of_pathogenicity | acetyl-coenzyme A synthetase | AMP-dependent synthetase/ligase |
| DN10_c0_g1_i1.g4247.t1 | PHI:8884 | aroG1 | *Ralstonia_solanacearum* | loss_of_pathogenicity | Phospho-2-dehydro-3-deoxyheptonate aldolase, tyrosine-inhibited | DAHP synthetase I/KDSA |
| DN10917_c0_g1_i1.g49587.t1 | PHI:4475 | MoTup1 | *Magnaporthe_oryzae* | loss_of_pathogenicity | hypothetical protein FOTG_00827 | U3 small nucleolar RNA-associated SSU processome protein (Utp13 protein) |
| DN11070_c0_g1_i1.g11576.t1 | PHI:2098 | Calcium-transporting_ATPase_3 | *Magnaporthe_oryzae* | loss_of_pathogenicity | Calcium-transporting ATPase 2 | Cation-transporting P-type ATPase, C-terminal |
| DN11799_c0_g1_i1.g7097.t1 | PHI:9357 | Fgleu1_(FGSG_09589) | *Fusarium_graminearum* | loss_of_pathogenicity | aconitate hydratase, mitochondrial | Aconitase A/isopropylmalate dehydratase small subunit, swivel domain |
| DN16121_c0_g1_i1.g5348.t1 | PHI:365__PHI:2267 | MLS1__Mls1 | *Parastagonospora_nodorum* | loss_of_pathogenicity | Malate synthase, glyoxysomal | Malate synthase |
| DN16389_c0_g1_i1.g15321.t1 | PHI:2145__PHI:9180 | MoSSADH__MoSSADH_(MGG_01230) | *Magnaporthe_oryzae* | loss_of_pathogenicity | betaine-aldehyde dehydrogenase | Aldehyde dehydrogenase domain |
| DN19110_c0_g1_i1.g21604.t1 | PHI:734 | FOW2 | *Fusarium_oxysporum* | loss_of_pathogenicity | hypothetical protein FOTG_08988 | Zn (2)-C6 fungal-type DNA-binding domain-GAL4 |
| DN20630_c0_g1_i1.g9653.t1 | PHI:9357 | Fgleu1_(FGSG_09589) | *Fusarium_graminearum* | loss_of_pathogenicity | Aconitate hydratase | Aconitase A/isopropylmalate dehydratase small subunit, swivel domain |
| DN2074_c0_g1_i1.g16922.t1 | PHI:4475 | MoTup1 | *Magnaporthe_oryzae* | loss_of_pathogenicity | probable CPC2 protein | WD domain, G-beta repeat |
| DN20813_c0_g1_i1.g15595.t1 | PHI:4475 | MoTup1 | *Magnaporthe_oryzae* | loss_of_pathogenicity | hypothetical protein FOTG_02856 | U3 small nucleolar RNA-associated protein 15, C-terminal |
| DN2498_c0_g1_i1.g13874.t1 | PHI:9357 | Fgleu1_(FGSG_09589) | *Fusarium_graminearum* | loss_of_pathogenicity | homoaconitase, mitochondrial | Aconitase/3-isopropylmalate dehydratase large subunit, alpha/beta/alpha domain |
| DN2974_c0_g1_i2.g18543.t1 | PHI:9357 | Fgleu1_(FGSG_09589) | *Fusarium_graminearum* | loss_of_pathogenicity | 3-isopropylmalate dehydratase | Aconitase/3-isopropylmalate dehydratase large subunit, alpha/beta/alpha domain |
| DN44_c0_g1_i3.g4329.t1 | PHI:4750 | ChPMA2 | *Colletotrichum_higginsianum* | loss_of_pathogenicity | plasma membrane ATPase | Cation-transporting P-type ATPase, N-terminal |
| DN447_c0_g1_i8.g29247.t1 | PHI:4475 | MoTup1 | *Magnaporthe_oryzae* | loss_of_pathogenicity | Periodic tryptophan protein 2 | WD domain, G-beta repeat |
| DN4549_c0_g1_i1.g3897.t1 | PHI:3307__PHI:3313 | gpf1 | *Magnaporthe_oryzae* | loss_of_pathogenicity | hypothetical protein FOMG_13855 | Zn (2)-C6 fungal-type DNA-binding domain |
| DN4723_c0_g1_i1.g19045.t1 | PHI:3162 | Mohik8 | *Magnaporthe_oryzae* | loss_of_pathogenicity | hypothetical protein FOTG_06316 | Protein kinase domain-Histidine kinase/HSP90-like ATPase |
| DN4917_c0_g1_i2.g6265.t1 | PHI:685__PHI:2022 | BUF__BUF1 | *Magnaporthe_oryzae* | loss_of_pathogenicity | hypothetical protein FOTG_03263 | Short-chain dehydrogenase/reductase SDR |
| DN572_c0_g1_i1.g10913.t1 | PHI:415 | IPMDH | *Parastagonospora_nodorum* | loss_of_pathogenicity | probable isocitrate dehydrogenase (NAD) | Isopropylmalate dehydrogenase-like domain |
| DN6161_c0_g1_i1.g15573.t1 | PHI:4475 | MoTup1 | *Magnaporthe_oryzae* | loss_of_pathogenicity | Putative WD repeat-containing protein C3D6.12 | U3 small nucleolar RNA-associated SSU processome protein (Utp12) |
| NODE_111.g8134.t1 | PHI:2694 | rsmB | *Pectobacterium_atrosepticum* | loss_of_pathogenicity | hypothetical protein FOTG_07624 | SAM-dependent methyltransferase RsmB/NOP2-type |
| DN13894_c0_g1_i1.g9576.t1 | PHI:2694 | rsmB | *Pectobacterium_atrosepticum* | loss_of_pathogenicity | hypothetical protein FOTG_07624 | SAM-dependent methyltransferase RsmB/NOP2-type |
| DN4235_c0_g1_i2.g13109.t1 | PHI:2115__PHI:4494 | Annexin_A7__AnnA7 | *Magnaporthe_oryzae* | loss_of_pathogenicity | Annexin A11 | Annexin repeat |
| NODE_10.g1401.t1 | PHI:6121 | FGSG_03243 | *Fusarium_graminearum* | reduced_virulence | hypothetical protein BFJ69_g6347 | Carboxylesterase, type B |
| NODE_10.g1421.t1 | PHI:7712 | CgRGS1 | *Colletotrichum_gloeosporioides* | reduced_virulence | Developmental regulator flbA | RGS domain, Regulator of G protein |
| NODE_101.g7666.t1 | PHI:2896 | BEC1005 | *Blumeria_graminis* | reduced_virulence | murein transglycosylase | Glycoside hydrolase family 17 |
| NODE_101.g7674.t1 | PHI:6594 | carA | *Pseudomonas_syringae* | reduced_virulence | Protein pyrABCN | Carbamoyl-phosphate synthetase large subunit-like, ATP-binding domain |
| NODE_104.g7816.t1 | PHI:2239 | Spe-Sdh | *Ustilago_maydis* | reduced_virulence | spermidine synthase | Spermidine synthase, tetramerisation domain |
| NODE_11.g1535.t1 | PHI:2240__PHI:4586 | Srt1 | *Ustilago_maydis* | reduced_virulence | MFS transporter, SP family, general alpha glucoside:H+ symporter | Major facilitator, sugar transporter-like |
| NODE_112.g8174.t1 | PHI:6261 | PsAAT3 | *Phytophthora_sojae* | reduced_virulence | aspartate aminotransferase, mitochondrial | Aminotransferase, class I/classII |
| NODE_112.g8177.t1 | PHI:9321 | trpB | *Erwinia_amylovora* | reduced_virulence | probable tryptophan synthase | Tryptophan synthase, alpha chain |
| NODE_115.g8315.t1 | PHI:2189 | MoGIS2 | *Magnaporthe_oryzae* | reduced_virulence | Cellular nucleic acid-binding protein like protein | Zinc finger, CCHC-type |
| NODE_119.g8464.t1 | PHI:9517 | FgEch1_(FGSG_13111) | *Fusarium_graminearum* | reduced_virulence | enoyl-CoA hydratase | Enoyl-CoA hydratase/isomerase |
| NODE_121.g8518.t1 | PHI:2130 | MoHox4 | *Magnaporthe_oryzae* | reduced_virulence | hypothetical protein FOTG_05526 |  |
| NODE_123.g8584.t1 | PHI:877 | MGG_00383 | *Magnaporthe_oryzae* | reduced_virulence | S-adenosylmethionine synthase | S-adenosylmethionine synthetase, C-terminal |
| NODE_127.g8757.t1 | PHI:3257 | MoIlv1 | *Magnaporthe_oryzae* | reduced_virulence | threonine ammonia-lyase, biosynthetic | Threonine dehydratase, ACT-like domain |
| NODE_127.g8760.t1 | PHI:3234 | MoLYS20 | *Magnaporthe_oryzae* | reduced_virulence | homocitrate synthase, mitochondrial | Pyruvate carboxyltransferase |
| NODE_127.g8770.t1 | PHI:3234 | MoLYS20 | *Magnaporthe_oryzae* | reduced_virulence | homocitrate synthase | Pyruvate carboxyltransferase |
| NODE_130.g8873.t1 | PHI:251 | FGA1 | *Fusarium_oxysporum* | reduced_virulence | predicted protein | Guanine nucleotide binding protein (G-protein), alpha subunit |
| NODE_135.g9012.t1 | PHI:9517 | FgEch1_(FGSG_13111) | *Fusarium_graminearum* | reduced_virulence | hypothetical protein FOTG_00564 | Enoyl-CoA hydratase/isomerase, HIBYL-CoA-H type |
| NODE_141.g9234.t1 | PHI:2174 | NMR2 | *Magnaporthe_oryzae* | reduced_virulence | hypothetical protein FOC1_g10006917 | NmrA-like domain |
| NODE_145.g9367.t1 | PHI:2058 | LHS1 | *Magnaporthe_oryzae* | reduced_virulence | heat shock 70kDa protein 4 | Heat shock protein 70 family |
| NODE_145.g9398.t1 | PHI:2911 | Ss-pth2 | *Sclerotinia_sclerotiorum* | reduced_virulence | carnitine O-acetyltransferase | Choline/carnitine acyltransferase domain |
| NODE_146.g9415.t1 | PHI:9056 | FgTRR_(FGSG_00871) | *Fusarium_graminearum* | reduced_virulence | thioredoxin reductase (NADPH) | Thioredoxin reductase |
| NODE_146.g9423.t1 | PHI:8695 | MoImd4_(MGG_03699) | *Magnaporthe_oryzae* | reduced_virulence | hypothetical protein FAVG1_01712 | IMP dehydrogenase/GMP reductase |
| NODE_151.g9572.t1 | PHI:1188 | (Sc_Sak1) | *Fusarium_graminearum* | reduced_virulence | hypothetical protein BFJ69_g5697 | Protein kinase domain |
| NODE_154.g9648.t1 | PHI:1090__PHI:1163__PHI:3893 | FGSG_10057__ERB1__FgEBR1 | *Fusarium_graminearum* | reduced_virulence | hypothetical protein FOC1_g10012848 | Zn (2)-C6 fungal-type DNA-binding domain |
| NODE_156.g9700.t1 | PHI:1211 | (Sc_Ctk1) | *Fusarium_graminearum* | reduced_virulence | CTD kinase subunit alpha | Protein kinase domain |
| NODE_157.g9711.t1 | PHI:6634__PHI:9301 | MoDnm1__Modnm1_(MGG_06361) | *Magnaporthe_oryzae* | reduced_virulence | dynamin GTPase | Dynamin superfamily |
| NODE_161.g9821.t1 | PHI:3914 | Sdh1 | *Parastagonospora_nodorum* | reduced_virulence | succinate-semialdehyde dehydrogenase (NADP+) | Aldehyde dehydrogenase domain |
| NODE_163.g9881.t1 | PHI:2058 | LHS1 | *Magnaporthe_oryzae* | reduced_virulence | probable heat shock protein 70 | Heat shock protein 70 family |
| NODE_164.g9927.t1 | PHI:8753__PHI:8806 | MoChia1_(MGG_08054)__MoChi1_(MGG_08054) | *Magnaporthe_oryzae* | reduced_virulence | hypothetical protein BFJ69_g14497 | Glycoside hydrolase family 18, catalytic domain |
| NODE_169.g10058.t1 | PHI:6395 | MoALR2 | *Magnaporthe_oryzae* | reduced_virulence | Putative metal ion transporter C27B12.12c | CorA-like Mg2+ transporter protein |
| NODE_170.g10064.t1 | PHI:1379 | GzC2H042 | *Fusarium_graminearum* | reduced_virulence | Cytoplasmic 60S subunit biogenesis factor REI1 | ZN622/Rei1/Reh1, zinc finger C2H2-type |
| NODE_177.g10257.t1 | PHI:9353 | leu2A_(MGG_05223) | *Magnaporthe_oryzae* | reduced_virulence | probable isocitrate dehydrogenase (NAD) | Isopropylmalate dehydrogenase-like domain |
| NODE_179.g10323.t1 | PHI:6135__PHI:6636 | PEF1__MoMdv1 | *Magnaporthe_oryzae* | reduced_virulence | F-box and WD-40 domain-containing protein CDC4 | WD domain, G-beta repeat |
| NODE_181.g10353.t1 | PHI:9191 | Fvset2_(FVEG_06937) | *Fusarium_verticillioides* | reduced_virulence | histone-lysine N-methyltransferase, H3 lysine-4 specific | Histone-lysine N-methyltransferase, H3 lysine-4 specific |
| NODE_186.g10496.t1 | PHI:3796 | Pst-Lov | *Pseudomonas_syringae* | reduced_virulence | hypothetical protein FOTG_04178 | PAS domain |
| NODE_194.g10701.t1 | PHI:2178 | MoSFl1 | *Magnaporthe_oryzae* | reduced_virulence | hypothetical protein FOXG_12803 | Heat shock factor (HSF)-type, DNA-binding |
| NODE_197.g10798.t1 | PHI:319 | SQL2 | *Ustilago_maydis* | reduced_virulence | hypothetical protein FOIG_07922 | guanine-nucleotide exchange factors catalytic domain (Ras) |
| NODE_206.g11003.t1 | PHI:1198 | (Sc_Ssk2/Ssk22) | *Fusarium_graminearum* | reduced_virulence | MAP kinase kinase kinase wis4 | Protein kinase domain-Mitogen-activated protein (MAP) kinase kinase kinase Ssk2/Ssk22 |
| NODE_21.g2559.t1 | PHI:2143 | MoVPR | *Magnaporthe_oryzae* | reduced_virulence | 4-aminobutyrate aminotransferase | Aminotransferase class-III |
| NODE_218.g11285.t1 | PHI:316__PHI:4606 | PiGPB1 | *Phytophthora_infestans* | reduced_virulence | hypothetical protein BFJ71_g3260 | WD domain, G-beta repeat |
| NODE_221.g11341.t1 | PHI:6928 | MoBRE1 | *Magnaporthe_oryzae* | reduced_virulence | E3 ubiquitin-protein ligase BRE1 | BRE1 E3 ubiquitin ligase |
| NODE_222.g11364.t1 | PHI:2911 | Ss-pth2 | *Sclerotinia_sclerotiorum* | reduced_virulence | Putative mitochondrial carnitine O-acetyltransferase | Choline/carnitine acyltransferase domain |
| NODE_23.g2719.t1 | PHI:881 | MGG_04556 | *Magnaporthe_oryzae* | reduced_virulence | hypothetical protein FOTG_08258 | Alcohol dehydrogenase, zinc-type, conserved site |
| NODE_237.g11658.t1 | PHI:4914 | ZtCBR1 | *Zymoseptoria_tritici* | reduced_virulence | NADH-cytochrome b5 reductase 1, partial | Flavoprotein pyridine nucleotide cytochrome reductase-like |
| NODE_251.g11898.t1 | PHI:744 | ADE5 | *Fusarium_graminearum* | reduced_virulence | hypothetical protein FOXG_10535 | Phosphoribosylglycinamide synthetase, ATP-grasp (A) domain |
| NODE_254.g11958.t1 | PHI:1441 | GzDDT | *Fusarium_graminearum* | reduced_virulence | Imitation switch two complex protein 1 | WSTF/Acf1/Cbp146 |
| NODE_26.g3041.t1 | PHI:9282 | mfsG_(BCIN_06g00026) | *Botrytis_cinerea* | reduced_virulence | hypothetical protein BFJ69_g13977 | Major facilitator superfamily |
| NODE_26.g3065.t1 | PHI:1384 | GzC2H047 | *Fusarium_graminearum* | reduced_virulence | hypothetical protein FOCG_02355 | Zinc finger C2H2-type |
| NODE_27.g3166.t1 | PHI:1422 | GzC2H090 | *Fusarium_graminearum* | reduced_virulence | hypothetical protein BFJ69_g14025 | Dual specificity protein phosphatase domain |
| NODE_28.g3177.t1 | PHI:177 | ODC | *Parastagonospora_nodorum* | reduced_virulence | ornithine decarboxylase | Orn/DAP/Arg decarboxylase 2 |
| NODE_285.g12429.t1 | PHI:2058 | LHS1 | *Magnaporthe_oryzae* | reduced_virulence | hypothetical protein FOIG_02155 | Heat shock protein 70 family |
| NODE_29.g3267.t1 | PHI:9408 | VdSkn7_(VDAG_02250) | *Verticillium_dahliae* | reduced_virulence | hypothetical protein FOXG_08113 | Heat shock factor (HSF)-type, DNA-binding |
| NODE_300.g12648.t1 | PHI:158 | UKC1 | *Ustilago_maydis* | reduced_virulence | hypothetical protein FPOA_00733 | Protein kinase domain |
| NODE_302.g12674.t1 | PHI:888 | MGG_01707 | *Magnaporthe_oryzae* | reduced_virulence | uncharacterized protein FFUJ_04877 | Mitochondrial protein |
| NODE_304.g12706.t1 | PHI:1093 | FGSG_02077 | *Fusarium_graminearum* | reduced_virulence | uncharacterized protein FMAN_11213 | CFEM domain |
| NODE_306.g12739.t1 | PHI:4491 | SHM | *Magnaporthe_oryzae* | reduced_virulence | serine hydroxymethyltransferase, cytosolic | Serine hydroxymethyltransferase-like domain |
| NODE_311.g12797.t1 | PHI:3019 | MoLys2 | *Magnaporthe_oryzae* | reduced_virulence | L-2-aminoadipate reductase large subunit | AMP-dependent synthetase/ligase |
| NODE_326.g12963.t1 | PHI:9320 | purL | *Erwinia_amylovora* | reduced_virulence | phosphoribosylformylglycinamidine synthase | Phosphoribosylformylglycinamidine synthase, N-terminal |
| NODE_33.g3603.t1 | PHI:5068 | MCC | *Fusarium_graminearum* | reduced_virulence | amidase | Amidase signature domain |
| NODE_33.g3610.t1 | PHI:5083 | MoAcat2 | *Magnaporthe_oryzae* | reduced_virulence | 3-ketoacyl-CoA thiolase B, peroxisomal | acetyl-coenzyme A acetyltransferases (Thiolases) |
| NODE_34.g3672.t1 | PHI:305 | ICL1 | *Magnaporthe_oryzae* | reduced_virulence | Isocitrate lyase | Isocitrate lyase |
| NODE_345.g13188.t1 | PHI:1291 | TOP1 | *Fusarium_graminearum* | reduced_virulence | DNA topoisomerase 1 | DNA topoisomerase I, DNA binding, eukaryotic type |
| NODE_35.g3765.t1 | PHI:8037 | Fvlds1 | *Fusarium_verticillioides* | reduced_virulence | hypothetical protein FOQG_10790 | Haem peroxidase superfamily |
| NODE_36.g3847.t1 | PHI:816 | MGG_04582 | *Magnaporthe_oryzae* | reduced_virulence | Putative beta-glucosidase btgE | Glycoside hydrolase superfamily |
| NODE_36.g3853.t1 | PHI:5187 | MoCCP1 | *Magnaporthe_oryzae* | reduced_virulence | cytochrome c peroxidase, mitochondrial | Haem peroxidase |
| NODE_364.g13359.t1 | PHI:2283 | AreA | *Fusarium_oxysporum* | reduced_virulence | nitrogen regulatory protein areA | Zinc finger, GATA-type |
| NODE_369.g13392.t1 | PHI:3308 | cnf1 | *Magnaporthe_oryzae* | reduced_virulence | hypothetical protein FOTG_09210 | Zn (2)-C6 fungal-type DNA-binding domain |
| NODE_37.g3925.t1 | PHI:4602 | FDB2 | *Fusarium_graminearum* | reduced_virulence | Fatty acid synthase subunit beta | Fatty acid synthase |
| NODE_392.g13595.t1 | PHI:132 | ABC1 | *Magnaporthe_oryzae* | reduced_virulence | ABC transporter CDR4 | ABC transporter-like |
| NODE_394.g13608.t1 | PHI:9222 | FgPEX1_(FGSG_07104) | *Fusarium_graminearum* | reduced_virulence | hypothetical protein FOTG_06965 | ATPase, AAA-type, core |
| NODE_409.g13718.t1 | PHI:1556 | GzNH001 | *Fusarium_graminearum* | reduced_virulence | hypothetical protein FOMG_06736 | Zinc finger, GATA-type |
| NODE_41.g4183.t1 | PHI:9317 | purF | *Erwinia_amylovora* | reduced_virulence | amidophosphoribosyltransferase | Amidophosphoribosyltransferase |
| NODE_41.g4238.t1 | PHI:737__PHI:2329 | CTB4 | *Cercospora_nicotianae* | reduced_virulence | hypothetical protein FOC1_g10010154 | Major facilitator superfamily |
| NODE_44.g4442.t1 | PHI:3315 | conx1 | *Magnaporthe_oryzae* | reduced_virulence | hypothetical protein FOTG_07898 |  |
| NODE_44.g4455.t1 | PHI:6261 | PsAAT3 | *Phytophthora_sojae* | reduced_virulence | Aspartate aminotransferase, cytoplasmic | Aminotransferases, class-I |
| NODE_44.g4459.t1 | PHI:7788 | AldB_(PSPTO_2673) | *Pseudomonas_syringae* | reduced_virulence | Phenylacetaldehyde dehydrogenase | Aldehyde dehydrogenase, C-terminal |
| NODE_44.g4482.t1 | PHI:5236 | Dnj1 | *Fusarium_oxysporum* | reduced_virulence | molecular chaperone DnaJ | Heat shock protein DnaJ, cysteine-rich domain |
| NODE_45.g4539.t1 | PHI:1366 | GzCON7 | *Fusarium_graminearum* | reduced_virulence | hypothetical protein FOIG_04164 |  |
| NODE_45.g4558.t1 | PHI:9302 | Mofzo1_(MGG_05209) | *Magnaporthe_oryzae* | reduced_virulence | mitofusin | P-loop containing nucleoside triphosphate hydrolase |
| NODE_49.g4771.t1 | PHI:1217 | FGSG_00792 | *Fusarium_graminearum* | reduced_virulence | CAMK/CAMKL/KIN4 protein kinase | Protein kinase domain |
| NODE_51.g4879.t1 | PHI:2189 | MoGIS2 | *Magnaporthe_oryzae* | reduced_virulence | related to hexamer-binding protein HEXBP | Zinc finger, CCHC-type |
| NODE_53.g5025.t1 | PHI:2839 | RED1 | *Bipolaris_maydis* | reduced_virulence | hypothetical protein FVEG_11599 | Polyketide synthase, enoylreductase domain |
| NODE_537.g14359.t1 | PHI:3941 | XC_0481 | *Xanthomonas_campestris* | reduced_virulence | aminodeoxychorismate synthase | Glutamine amidotransferase |
| NODE_55.g5156.t1 | PHI:243 | CHIP6 | *Colletotrichum_gloeosporioides* | reduced_virulence | hypothetical protein FOTG_13023 | Glycosyltransferase family 28 |
| NODE_551.g14401.t1 | PHI:2432 | FgRgsA | *Fusarium_graminearum* | reduced_virulence | hypothetical protein BFJ71_g14823 | RGS domain, regulator of G protein signaling |
| NODE_57.g5262.t1 | PHI:6927 | MoRAD6 | *Magnaporthe_oryzae* | reduced_virulence | Ubiquitin-conjugating enzyme | Ubiquitin-conjugating enzyme E2 |
| NODE_59.g5421.t1 | PHI:2240__PHI:4586 | Srt1 | *Ustilago_maydis* | reduced_virulence | MFS transporter, SP family, general alpha glucoside:H+ symporter | Major facilitator, sugar transporter-like |
| NODE_6.g951.t1 | PHI:9144 | FgAP2s_(FGSG_02015) | *Fusarium_graminearum* | reduced_virulence | hypothetical protein BFJ69_g6821 | Alpha/beta hydrolase fold-1 |
| NODE_61.g5514.t1 | PHI:1193 | (Sc_Sky1) | *Fusarium_graminearum* | reduced_virulence | hypothetical protein BFJ70_g11808 | Protein kinase domain |
| NODE_61.g5524.t1 | PHI:7975 | Nop53 | *Botrytis_cinerea* | reduced_virulence | hypothetical protein BFJ70_g11791 | Ribosome biogenesis protein Nop53/GLTSCR2 |
| NODE_63.g5649.t1 | PHI:1403 | GzC2H066 | *Fusarium_graminearum* | reduced_virulence | hypothetical protein FSPOR_8584 | Zinc finger C2H2-type |
| NODE_63.g5663.t1 | PHI:5471 | MoDac | *Magnaporthe_oryzae* | reduced_virulence | Putative N-acetylglucosamine-6-phosphate deacetylase | N-acetylglucosamine-6-phosphate deacetylase |
| NODE_64.g5701.t1 | PHI:7672 | FgLetm1 | *Fusarium_graminearum* | reduced_virulence | hypothetical protein FOC4_g10001384 | Letm1 ribosome-binding domain |
| NODE_649.g14609.t1 | PHI:7787 | AldA_(PSPTO_0092) | *Pseudomonas_syringae* | reduced_virulence | Betaine aldehyde dehydrogenase | Aldehyde dehydrogenase domain |
| NODE_66.g5818.t1 | PHI:3941 | XC_0481 | *Xanthomonas_campestris* | reduced_virulence | probable GMP synthase | GMP synthase, C-terminal |
| NODE_69.g5995.t1 | PHI:3816 | GSN1 | *Magnaporthe_oryzae* | reduced_virulence | glycogen | Glycogen synthase |
| NODE_70.g6068.t1 | PHI:2171 | Peroxisomal_copper_amine_oxidase | *Magnaporthe_oryzae* | reduced_virulence | Peroxisomal primary amine oxidase | Copper amine oxidase, catalytic domain |
| NODE_70.g6070.t1 | PHI:7788 | AldB_(PSPTO_2673) | *Pseudomonas_syringae* | reduced_virulence | Aldehyde dehydrogenase | Aldehyde dehydrogenase domain |
| NODE_71.g6100.t1 | PHI:881 | MGG_04556 | *Magnaporthe_oryzae* | reduced_virulence | hypothetical protein FOXG_04143 | Polyketide synthase, enoylreductase domain |
| NODE_72.g6184.t1 | PHI:9353 | leu2A_(MGG_05223) | *Magnaporthe_oryzae* | reduced_virulence | Putative tartrate dehydrogenase/decarboxylase ttuC | Isocitrate/isopropylmalate dehydrogenase |
| NODE_74.g6266.t1 | PHI:9476 | CoTHR4_(Cob_04177) | *Colletotrichum_orbiculare* | reduced_virulence | Threonine synthase | Threonine synthase, N-terminal |
| NODE_74.g6303.t1 | PHI:9319 | leuB | *Erwinia_amylovora* | reduced_virulence | probable isocitrate dehydrogenase | Isocitrate/isopropylmalate dehydrogenase |
| NODE_75.g6336.t1 | PHI:1641__PHI:2434 | FgFlbA | *Fusarium_graminearum* | reduced_virulence | hypothetical protein FOXG_08482 | RGS domain, Regulator of G protein signaling |
| NODE_76.g6409.t1 | PHI:5826 | GIV4 | *Fusarium_graminearum* | reduced_virulence | hypothetical protein FOC1_g10006143 |  |
| NODE_78.g6500.t1 | PHI:5236 | Dnj1 | *Fusarium_oxysporum* | reduced_virulence | hypothetical protein BFJ69_g6102 | Chaperone J-domain superfamily |
| NODE_81.g6696.t1 | PHI:5068 | MCC | *Fusarium_graminearum* | reduced_virulence | Pyruvate carboxylase | Carbamoyl-phosphate synthetase large subunit-like, ATP-binding domain |
| NODE_84.g6859.t1 | PHI:5353 | Famfs1 | *Fusarium_asiaticum* | reduced_virulence | hypothetical protein BFJ69_g21 | Major facilitator superfamily |
| NODE_86.g6952.t1 | PHI:5017 | MoPEX11A | *Magnaporthe_oryzae* | reduced_virulence | hypothetical protein FAVG1_03363 | Peroxisomal biogenesis factor 11 |
| NODE_9.g1263.t1 | PHI:7172 | Clp-1 | *Verticillium_dahliae* | reduced_virulence | hypothetical protein FOXG_02452 | Peptidase C2, calpain, catalytic domain |
| NODE_9.g1334.t1 | PHI:5068 | MCC | *Fusarium_graminearum* | reduced_virulence | probable acetyl-CoA carboxylase | Acetyl-CoA carboxylase, central domain |
| NODE_97.g7532.t1 | PHI:6927 | MoRAD6 | *Magnaporthe_oryzae* | reduced_virulence | probable ubiquitin-conjugating enzyme CDC34 | Ubiquitin-conjugating enzyme E2 |
| DN10030_c0_g1_i1.g15705.t1 | PHI:2058 | LHS1 | *Magnaporthe_oryzae* | reduced_virulence | probable heat shock protein 70 | Heat shock protein 70 family |
| DN10165_c0_g1_i1.g13244.t1 | PHI:2130 | MoHox4 | *Magnaporthe_oryzae* | reduced_virulence | hypothetical protein FOTG_05294 | PTP type protein phosphatase |
| DN10249_c0_g1_i1.g12851.t1 | PHI:8753__PHI:8806 | MoChia1_(MGG_08054)__MoChi1_(MGG_08054) | *Magnaporthe_oryzae* | reduced_virulence | hypothetical protein BFJ69_g14497 | Glycoside hydrolase superfamily |
| DN1045_c0_g1_i1.g13326.t1 | PHI:1180 | (Sc_Sat4) | *Fusarium_graminearum* | reduced_virulence | Serine/threonine-protein kinase oca2 | Protein kinase domain |
| DN106_c0_g1_i1.g1562.t1 | PHI:8695 | MoImd4_(MGG_03699) | *Magnaporthe_oryzae* | reduced_virulence | hypothetical protein FAVG1_01712 | IMP dehydrogenase/GMP reductase |
| DN1069_c0_g1_i1.g39362.t1 | PHI:9144 | FgAP2s_(FGSG_02015) | *Fusarium_graminearum* | reduced_virulence | hypothetical protein BFJ69_g6821 | Carboxylesterase, type B |
| DN1081_c0_g1_i2.g13439.t1 | PHI:2405 | MoSSK1 | *Magnaporthe_oryzae* | reduced_virulence | hypothetical protein FOTG_14309 | Protein kinase domain-Histidine kinase/HSP90-like ATPase |
| DN10864_c0_g1_i1.g14247.t1 | PHI:812 | MGG_10702 | *Magnaporthe_oryzae* | reduced_virulence | hypothetical protein BFJ69_g13977 | Major facilitator superfamily |
| DN1111_c0_g1_i1.g17654.t1 | PHI:2911 | Ss-pth2 | *Sclerotinia_sclerotiorum* | reduced_virulence | carnitine O-acetyltransferase | Choline/carnitine acyltransferase domain |
| DN11110_c0_g1_i1.g49980.t1 | PHI:8753__PHI:8806 | MoChia1_(MGG_08054)__MoChi1_(MGG_08054) | *Magnaporthe_oryzae* | reduced_virulence | hypothetical protein BFJ69_g14497 | Glycoside hydrolase family 18, catalytic domain |
| DN11394_c0_g1_i1.g11540.t1 | PHI:9302 | Mofzo1_(MGG_05209) | *Magnaporthe_oryzae* | reduced_virulence | mitofusin | Dynamin superfamily |
| DN11417_c0_g1_i2.g3786.t1 | PHI:5236 | Dnj1 | *Fusarium_oxysporum* | reduced_virulence | molecular chaperone DnaJ | Chaperone DnaJ, C-terminal |
| DN11460_c0_g1_i1.g3756.t1 | PHI:5068 | MCC | *Fusarium_graminearum* | reduced_virulence | probable acetyl-CoA carboxylase | Carbamoyl-phosphate synthetase large subunit-like, ATP-binding domain |
| DN11577_c0_g1_i1.g184.t1 | PHI:6928 | MoBRE1 | *Magnaporthe_oryzae* | reduced_virulence | E3 ubiquitin-protein ligase BRE1 | Zinc finger, RING-type |
| DN12007_c0_g1_i1.g11786.t1 | PHI:5353 | Famfs1 | *Fusarium_asiaticum* | reduced_virulence | related to E. coli tetracycline resistance protein TCR1 |  |
| DN12141_c0_g1_i1.g13216.t1 | PHI:4491 | SHM | *Magnaporthe_oryzae* | reduced_virulence | serine hydroxymethyltransferase, mitochondrial | Serine hydroxymethyltransferase-like domain |
| DN12593_c0_g1_i1.g17215.t1 | PHI:7712 | CgRGS1 | *Colletotrichum_gloeosporioides* | reduced_virulence | Developmental regulator flbA | Winged helix DNA-binding domain superfamily |
| DN13637_c0_g1_i1.g35173.t1 | PHI:5353 | Famfs1 | *Fusarium_asiaticum* | reduced_virulence | hypothetical protein BFJ69_g21 | Major facilitator superfamily |
| DN1370_c0_g1_i2.g14575.t1 | PHI:2839 | RED1 | *Bipolaris_maydis* | reduced_virulence | hypothetical protein FOMG_02164 | Polyketide synthase, enoylreductase domain |
| DN13702_c0_g1_i1.g4207.t1 | PHI:6121 | FGSG_03243 | *Fusarium_graminearum* | reduced_virulence | hypothetical protein BFJ69_g6347 | Carboxylesterase, type B |
| DN13775_c0_g1_i1.g395.t1 | PHI:5471 | MoDac | *Magnaporthe_oryzae* | reduced_virulence | Putative N-acetylglucosamine-6-phosphate deacetylase | N-acetylglucosamine-6-phosphate deacetylase |
| DN1382_c0_g1_i16.g45825.t1 | PHI:2174 | NMR2 | *Magnaporthe_oryzae* | reduced_virulence | hypothetical protein FOC1_g10006917 | NAD(P)H-binding |
| DN14032_c0_g1_i1.g7815.t1 | PHI:9222 | FgPEX1_(FGSG_07104) | *Fusarium_graminearum* | reduced_virulence | hypothetical protein FOMG_08604 | ATPase, AAA-type, core |
| DN1438_c0_g1_i2.g868.t1 | PHI:4491 | SHM | *Magnaporthe_oryzae* | reduced_virulence | serine hydroxymethyltransferase, cytosolic | Serine hydroxymethyltransferase-like domain |
| DN1451_c0_g1_i3.g647.t1 | PHI:2058 | LHS1 | *Magnaporthe_oryzae* | reduced_virulence | heat shock 70kDa protein 4 | Heat shock protein 70 family |
| DN1452_c0_g1_i2.g771.t1 | PHI:8924 | FgPLD1_(FGSG_09917) | *Fusarium_graminearum* | reduced_virulence | Phospholipase D1 | Phospholipase D/Transphosphatidylase |
| DN14739_c0_g1_i1.g44771.t1 | PHI:2171 | Peroxisomal_copper_amine_oxidase | *Magnaporthe_oryzae* | reduced_virulence | Peroxisomal primary amine oxidase | Copper amine oxidase, catalytic domain |
| DN155_c0_g1_i1.g1611.t1 | PHI:2239 | Spe-Sdh | *Ustilago_maydis* | reduced_virulence | spermidine synthase | Spermidine synthase, tetramerisation domain |
| DN1556_c0_g1_i2.g3491.t1 | PHI:6135__PHI:6636 | PEF1__MoMdv1 | *Magnaporthe_oryzae* | reduced_virulence | F-box and WD-40 domain-containing protein CDC4 | WD domain, G-beta repeat |
| DN15940_c0_g1_i1.g6898.t1 | PHI:8733 | BcKDM1_(Bcin07g03050) | *Botrytis_cinerea* | reduced_virulence | hypothetical protein BFJ69_g2823 |  |
| DN1606_c0_g1_i3.g7933.t1 | PHI:382__PHI:2233 | SMU1__Smu1 | *Ustilago_maydis* | reduced_virulence | Serine/threonine-protein kinase MST20 | Protein kinase domain |
| DN1623_c0_g1_i1.g8015.t1 | PHI:3315 | conx1 | *Magnaporthe_oryzae* | reduced_virulence | hypothetical protein FOTG_07898 | Zn (2)-C6 fungal-type DNA-binding domain |
| DN16275_c0_g1_i1.g14954.t1 | PHI:6927 | MoRAD6 | *Magnaporthe_oryzae* | reduced_virulence | probable ubiquitin-conjugating enzyme CDC34 | Ubiquitin-conjugating enzyme E2 |
| DN16286_c0_g1_i1.g14965.t1 | PHI:1384 | GzC2H047 | *Fusarium_graminearum* | reduced_virulence | hypothetical protein FOCG_02355 | Zinc finger C2H2 superfamily |
| DN16335_c0_g1_i1.g15298.t1 | PHI:9476 | CoTHR4_(Cob_04177) | *Colletotrichum_orbiculare* | reduced_virulence | Threonine synthase | Threonine synthase, N-terminal |
| DN16383_c0_g1_i1.g17142.t1 | PHI:9282 | mfsG_(BCIN_06g00026) | *Botrytis_cinerea* | reduced_virulence | hypothetical protein FOXG_05893 | Major facilitator superfamily |
| DN16407_c0_g1_i1.g5259.t1 | PHI:6261 | PsAAT3 | *Phytophthora_sojae* | reduced_virulence | Aspartate aminotransferase, cytoplasmic | Aminotransferase, class I/classII |
| DN16522_c0_g1_i2.g14898.t1 | PHI:881 | MGG_04556 | *Magnaporthe_oryzae* | reduced_virulence | hypothetical protein BFJ69_g3297 | Alcohol dehydrogenase, N-terminal |
| DN16593_c0_g1_i1.g14900.t1 | PHI:7672 | FgLetm1 | *Fusarium_graminearum* | reduced_virulence | hypothetical protein FOC4_g10001384 | Letm1 ribosome-binding domain |
| DN17093_c0_g1_i1.g21692.t1 | PHI:7194 | WISH | *Magnaporthe_oryzae* | reduced_virulence | hypothetical protein BFJ69_g6466 | integral membrane protein |
| DN1757_c0_g1_i1.g48161.t1 | PHI:881 | MGG_04556 | *Magnaporthe_oryzae* | reduced_virulence | hypothetical protein FOXG_04143 | Polyketide synthase, enoylreductase domain |
| DN17699_c0_g1_i1.g35513.t1 | PHI:881 | MGG_04556 | *Magnaporthe_oryzae* | reduced_virulence | hypothetical protein BFJ65_g6361 | Alcohol dehydrogenase, C-terminal |
| DN18082_c0_g1_i1.g18682.t1 | PHI:2178 | MoSFl1 | *Magnaporthe_oryzae* | reduced_virulence | hypothetical protein FOXG_12803 | Heat shock factor (HSF)-type, DNA-binding |
| DN18264_c0_g1_i1.g11644.t1 | PHI:6262 | FUG1 | *Fusarium_verticillioides* | reduced_virulence | hypothetical protein FOC1_g10011313 | Nitrogen regulatory protein areA, GATA-like domain |
| DN18485_c0_g1_i1.g10335.t1 | PHI:132 | ABC1 | *Magnaporthe_oryzae* | reduced_virulence | ABC transporter CDR4 | ABC-2 type transporter |
| DN18491_c0_g1_i1.g10313.t1 | PHI:737__PHI:2329 | CTB4 | *Cercospora_nicotianae* | reduced_virulence | hypothetical protein FOC1_g10010154 | Major facilitator superfamily |
| DN1919_c0_g1_i1.g11242.t1 | PHI:9320 | purL | *Erwinia_amylovora* | reduced_virulence | Phosphoribosylformylglycinamidine synthase | Glutamine amidotransferase domain |
| DN19198_c0_g1_i1.g21594.t1 | PHI:2240__PHI:4586 | Srt1 | *Ustilago_maydis* | reduced_virulence | Maltose permease MAL31 | Major facilitator, sugar transporter-like |
| DN1931_c0_g1_i1.g11154.t1 | PHI:2351__PHI:2353 | AMT1 | *Fusarium_graminearum* | reduced_virulence | probable hnRNP arginine N-methyltransferase | Ribosomal protein L11 |
| DN1995_c0_g1_i3.g41605.t1 | PHI:9476 | CoTHR4_(Cob_04177) | *Colletotrichum_orbiculare* | reduced_virulence | Threonine synthase | Threonine synthase, N-terminal |
| DN20417_c0_g1_i1.g3153.t1 | PHI:3796 | Pst-Lov | *Pseudomonas_syringae* | reduced_virulence | hypothetical protein FOTG_04178 | PAS domain |
| DN20547_c0_g1_i1.g1037.t1 | PHI:9353 | leu2A_(MGG_05223) | *Magnaporthe_oryzae* | reduced_virulence | Putative tartrate dehydrogenase/decarboxylase ttuC | Isopropylmalate dehydrogenase-like domain |
| DN2090_c0_g1_i1.g16870.t1 | PHI:3941 | XC_0481 | *Xanthomonas_campestris* | reduced_virulence | probable GMP synthase | GMP synthase, C-terminal |
| DN2097_c0_g1_i11.g19443.t1 | PHI:319 | SQL2 | *Ustilago_maydis* | reduced_virulence | hypothetical protein FOIG_07922 | Guanine nucleotide exchange factor (Ras-like) |
| DN21024_c0_g1_i1.g91.t1 | PHI:258 | GPABC1 | *Fusarium_sambucinum* | reduced_virulence | ABC transporter G family member 14 | ABC transporter-like |
| DN2103_c0_g1_i1.g6211.t1 | PHI:1422 | GzC2H090 | *Fusarium_graminearum* | reduced_virulence | hypothetical protein BFJ69_g14025 | Zinc finger C2H2 superfamily |
| DN21429_c0_g1_i1.g387.t1 | PHI:5083 | MoAcat2 | *Magnaporthe_oryzae* | reduced_virulence | 3-ketoacyl-CoA thiolase B, peroxisomal | acetyl-coenzyme A acetyltransferases (Thiolases) |
| DN21461_c0_g1_i1.g393.t1 | PHI:305 | ICL1 | *Magnaporthe_oryzae* | reduced_virulence | isocitrate lyase | Isocitrate lyase |
| DN21682_c0_g1_i1.g10236.t1 | PHI:3234 | MoLYS20 | *Magnaporthe_oryzae* | reduced_virulence | Homocitrate synthase, mitochondrial | Pyruvate carboxyltransferase |
| DN2199_c0_g1_i1.g6339.t1 | PHI:1090__PHI:1163__PHI:3893 | FGSG_10057__ERB1__FgEBR1 | *Fusarium_graminearum* | reduced_virulence | hypothetical protein FOC1_g10012848 | Zn (2)-C6 fungal-type DNA-binding domain |
| DN2230_c0_g1_i2.g25695.t1 | PHI:1641__PHI:2434 | FgFlbA | *Fusarium_graminearum* | reduced_virulence | Developmental regulator flbA | RGS domain, Regulator of G protein signaling domain |
| DN231_c0_g1_i10.g2281.t1 | PHI:550 | BOS1 | *Botrytis_cinerea* | reduced_virulence | NIK-1 nonidentical kinase-1 | Protein kinase domain-histidine kinase, dimerisation/phosphoacceptor domain |
| DN2365_c0_g1_i5.g40631.t1 | PHI:9191 | Fvset2_(FVEG_06937) | *Fusarium_verticillioides* | reduced_virulence | histone-lysine N-methyltransferase, H3 lysine-4 specific | Histone lysine methyltransferase SET associated |
| DN2372_c0_g1_i1.g12369.t1 | PHI:6395 | MoALR2 | *Magnaporthe_oryzae* | reduced_virulence | Putative metal ion transporter C27B12.12c | CorA-like Mg2+ transporter protein |
| DN2440_c0_g1_i3.g11200.t1 | PHI:9321 | trpB | *Erwinia_amylovora* | reduced_virulence | probable tryptophan synthase | Tryptophan synthase, alpha chain |
| DN2675_c0_g1_i1.g6109.t1 | PHI:3308 | cnf1 | *Magnaporthe_oryzae* | reduced_virulence | hypothetical protein FOTG_09210 | Zn (2)-C6 fungal-type DNA-binding domain |
| DN2677_c0_g1_i1.g6022.t1 | PHI:744 | ADE5 | *Fusarium_graminearum* | reduced_virulence | hypothetical protein FOXG_10535 | Phosphoribosylglycinamide synthetase, ATP-grasp (A) domain |
| DN2688_c0_g1_i1.g5996.t1 | PHI:1188 | (Sc_Sak1) | *Fusarium_graminearum* | reduced_virulence | hypothetical protein BFJ69_g5697 | Protein kinase domain |
| DN2721_c0_g1_i2.g15133.t1 | PHI:3958 | XC_0423 | *Xanthomonas_campestris* | reduced_virulence | 1,4-alpha-glucan-branching enzyme | Glycoside hydrolase, family 13, N-terminal |
| DN2793_c0_g1_i2.g15202.t1 | PHI:305 | ICL1 | *Magnaporthe_oryzae* | reduced_virulence | Isocitrate lyase | Isocitrate lyase |
| DN2871_c0_g1_i2.g15416.t1 | PHI:243 | CHIP6 | *Colletotrichum_gloeosporioides* | reduced_virulence | hypothetical protein FOPG_03657 | Glycosyltransferase family 28, N-terminal domain |
| DN2947_c0_g1_i2.g18540.t1 | PHI:2158 | MoCMK1 | *Magnaporthe_oryzae* | reduced_virulence | serine/threonine protein kinase | Protein kinase domain |
| DN2982_c0_g1_i2.g11935.t1 | PHI:1538__PHI:4209__PHI:4236 | GzMyb002__MYT3 | *Fusarium_graminearum* | reduced_virulence | hypothetical protein FOTG_06235 | Homeobox-like domain superfamily |
| DN3045_c0_g1_i3.g1207.t1 | PHI:2911 | Ss-pth2 | *Sclerotinia_sclerotiorum* | reduced_virulence | Putative mitochondrial carnitine O-acetyltransferase | Choline/carnitine acyltransferase domain |
| DN3050_c0_g1_i2.g1227.t1 | PHI:9319 | leuB | *Erwinia_amylovora* | reduced_virulence | probable isocitrate dehydrogenase | Isopropylmalate dehydrogenase-like domain |
| DN3086_c0_g1_i1.g1208.t1 | PHI:1641__PHI:2434 | FgFlbA | *Fusarium_graminearum* | reduced_virulence | hypothetical protein FOXG_08482 | RGS domain, regulator of G protein |
| DN3210_c0_g1_i1.g5401.t1 | PHI:2189 | MoGIS2 | *Magnaporthe_oryzae* | reduced_virulence | related to hexamer-binding protein HEXBP | Zinc finger, CCHC-type |
| DN3271_c0_g1_i1.g50347.t1 | PHI:9222 | FgPEX1_(FGSG_07104) | *Fusarium_graminearum* | reduced_virulence | hypothetical protein FOTG_06965 | ATPase, AAA-type, core |
| DN3302_c0_g1_i1.g8338.t1 | PHI:5353 | Famfs1 | *Fusarium_asiaticum* | reduced_virulence | hypothetical protein FOIG_08082 | Major facilitator superfamily |
| DN3453_c0_g1_i1.g18923.t1 | PHI:4602 | FDB2 | *Fusarium_graminearum* | reduced_virulence | Fatty acid synthase subunit beta | Fatty acid synthase subunit beta, N-terminal domain |
| DN3623_c0_g1_i1.g5084.t1 | PHI:2334 | BcNma | *Botrytis_cinerea* | reduced_virulence | hypothetical protein FOC4_g10011984 | Peptidase S1, PA clan |
| DN3635_c0_g1_i2.g5108.t1 | PHI:9282 | mfsG_(BCIN_06g00026) | *Botrytis_cinerea* | reduced_virulence | hypothetical protein FOTG_12210 | Major facilitator superfamily |
| DN368_c0_g1_i11.g32940.t1 | PHI:1366 | GzCON7 | *Fusarium_graminearum* | reduced_virulence | hypothetical protein FOIG_04164 | Zinc finger C2H2-type |
| DN3784_c0_g1_i2.g14552.t1 | PHI:1211 | (Sc_Ctk1) | *Fusarium_graminearum* | reduced_virulence | CTD kinase subunit alpha | Protein kinase domain |
| DN399_c0_g1_i16.g33912.t1 | PHI:9517 | FgEch1_(FGSG_13111) | *Fusarium_graminearum* | reduced_virulence | hypothetical protein FOTG_00564 | Enoyl-CoA hydratase/isomerase, HIBYL-CoA-H type |
| DN4562_c0_g1_i1.g3902.t1 | PHI:9320 | purL | *Erwinia_amylovora* | reduced_virulence | Phosphoribosylformylglycinamidine synthase | Phosphoribosylformylglycinamidine synthase, N-terminal |
| DN4605_c0_g1_i1.g9384.t1 | PHI:3019 | MoLys2 | *Magnaporthe_oryzae* | reduced_virulence | L-aminoadipate-semialdehyde dehydrogenase | AMP-dependent synthetase/ligase |
| DN461_c0_g1_i5.g2294.t1 | PHI:5017 | MoPEX11A | *Magnaporthe_oryzae* | reduced_virulence | hypothetical protein FAVG1_03363 | Peroxisomal biogenesis factor 11 |
| DN4640_c0_g1_i1.g9380.t1 | PHI:7172 | Clp-1 | *Verticillium_dahliae* | reduced_virulence | hypothetical protein FOXG_02452 | Peptidase C2, calpain, catalytic domain |
| DN4742_c0_g1_i2.g19060.t1 | PHI:8645 | FgLDHL1_(FGSG_00145) | *Fusarium_graminearum* | reduced_virulence | L-lactate dehydrogenase (cytochrome) | FMN-dependent dehydrogenase |
| DN4811_c0_g1_i1.g50753.t1 | PHI:5826 | GIV4 | *Fusarium_graminearum* | reduced_virulence | hypothetical protein FOC1_g10006143 |  |
| DN5081_c0_g1_i1.g7665.t1 | PHI:3914 | Sdh1 | *Parastagonospora_nodorum* | reduced_virulence | succinate-semialdehyde dehydrogenase (NADP+) | Aldehyde dehydrogenase domain |
| DN5098_c0_g1_i2.g7627.t1 | PHI:3816 | GSN1 | *Magnaporthe_oryzae* | reduced_virulence | glycogen | Glycogen synthase |
| DN5515_c0_g1_i1.g4908.t1 | PHI:8646 | FgLDHL2_(FGSG_16220) | *Fusarium_graminearum* | reduced_virulence | probable CYB2-lactate dehydrogenase cytochrome b2 | FMN-dependent dehydrogenase |
| DN582_c0_g1_i13.g29701.t1 | PHI:2130 | MoHox4 | *Magnaporthe_oryzae* | reduced_virulence | hypothetical protein FOTG_05526 |  |
| DN5860_c0_g1_i1.g47731.t1 | PHI:2198__PHI:8110 | MoRgs7__MoRgs7_(MGG_11693) | *Magnaporthe_oryzae* | reduced_virulence | hypothetical protein FOC1_g10003033 | RGS domain superfamily |
| DN5878_c0_g1_i1.g11732.t1 | PHI:9320 | purL | *Erwinia_amylovora* | reduced_virulence | Phosphoribosylformylglycinamidine synthase | PurM-like, N-terminal domain superfamily |
| DN6219_c0_g1_i1.g6630.t1 | PHI:1193 | (Sc_Sky1) | *Fusarium_graminearum* | reduced_virulence | hypothetical protein BFJ70_g11808 | Protein kinase domain |
| DN7097_c0_g1_i3.g267.t1 | PHI:8733 | BcKDM1_(Bcin07g03050) | *Botrytis_cinerea* | reduced_virulence | hypothetical protein FOTG_05352 | JmjC domain |
| DN71_c0_g1_i3.g4434.t1 | PHI:1533 | GzLam002 | *Fusarium_graminearum* | reduced_virulence | multiprotein-bridging factor 1 | Multiprotein bridging factor 1 |
| DN742_c0_g1_i2.g18229.t1 | PHI:7788 | AldB_(PSPTO_2673) | *Pseudomonas_syringae* | reduced_virulence | Phenylacetaldehyde dehydrogenase | Aldehyde dehydrogenase domain |
| DN7590_c0_g1_i1.g4782.t1 | PHI:6594 | carA | *Pseudomonas_syringae* | reduced_virulence | Protein pyrABCN | Carbamoyl-phosphate synthase small subunit, N-terminal domain |
| DN886_c0_g1_i9.g13928.t1 | PHI:806 | MGG_04137 | *Magnaporthe_oryzae* | reduced_virulence | related to LisH motif-containing protein | LIS1 homology motif |
| NODE_98.g7565.t1 | PHI:1335__PHI:3110__PHI:3808__PHI:4244 | GzbZIP017__FgATF1__Atf1__FgOS-2 | *Fusarium_graminearum* | reduced_virulence | hypothetical protein FOTG_03037 | bZIP |
| DN3387_c0_g1_i1.g8311.t1 | PHI:1335__PHI:3110__PHI:3808__PHI:4244 | GzbZIP017__FgATF1__Atf1__FgOS-2 | *Fusarium_graminearum* | reduced_virulence | hypothetical protein FOTG_03037 | Transcription factor Aft1, HRR domain |
| NODE_178.g10284.t1 | PHI:2100__PHI:9482 | Spf1__Spf1_(MGG_12005) | *Magnaporthe_oryzae* | reduced_virulence | ATPase | P-type ATPase, subfamily V |
| NODE_226.g11460.t1 | PHI:2099 | Pmc1 | *Magnaporthe_oryzae* | reduced_virulence | Calcium-transporting ATPase 2 | Cation-transporting P-type ATPase, C-terminal |
| DN1559_c0_g1_i2.g3498.t1 | PHI:2100__PHI:9482 | Spf1__Spf1_(MGG_12005) | *Magnaporthe_oryzae* | reduced_virulence | ATPase | P-type ATPase, subfamily V |
| DN18308_c0_g1_i1.g17298.t1 | PHI:2099 | Pmc1 | *Magnaporthe_oryzae* | reduced_virulence | Calcium-transporting ATPase 2 | P-type ATPase, cytoplasmic domain N |
| DN20781_c0_g1_i1.g14644.t1 | PHI:2099 | Pmc1 | *Magnaporthe_oryzae* | reduced_virulence | Calcium-transporting ATPase 2 | Haloacid dehalogenase-like hydrolase |
| DN2330_c2_g1_i1.g12195.t1 | PHI:2099 | Pmc1 | *Magnaporthe_oryzae* | reduced_virulence | Calcium-transporting ATPase 2 | Cation-transporting P-type ATPase, C-terminal |
| NODE_126.g8714.t1 | PHI:2746 | treZ | *Pseudomonas_aeruginosa* | reduced_virulence | 1,4-alpha-glucan-branching enzyme | Glycoside hydrolase, family 13, N-terminal |
| NODE_21.g2629.t1 | PHI:8939 | ndh_(PA4538) | *Pseudomonas_aeruginosa* | reduced_virulence | hypothetical protein BFJ66_g8830 | Pyridine nucleotide-disulphide oxidoreductase |
| NODE_44.g4491.t1 | PHI:6259 | DOHH | *Fusarium_graminearum* | reduced_virulence | Deoxyhypusine hydroxylase | Armadillo-like helical |
| NODE_45.g4555.t1 | PHI:5450 | gdh2 | *Colletotrichum_gloeosporioides* | reduced_virulence | NAD-specific glutamate dehydrogenase | Glutamate/phenylalanine/leucine/valine dehydrogenase, C-terminal |
| DN18268_c0_g1_i1.g11691.t1 | PHI:5450 | gdh2 | *Colletotrichum_gloeosporioides* | reduced_virulence | NAD-specific glutamate dehydrogenase | Glutamate/phenylalanine/leucine/valine dehydrogenase, C-terminal |
| DN3026_c0_g1_i3.g1193.t1 | PHI:2994__PHI:5604 | MGG_06355.6__FZC14 | *Magnaporthe_oryzae* | reduced_virulence | Activator of stress proteins 1 | Zn (2)-C6 fungal-type DNA-binding domain |
| DN73_c0_g1_i2.g4429.t1 | PHI:6259 | DOHH | *Fusarium_graminearum* | reduced_virulence | Deoxyhypusine hydroxylase | Armadillo-type fold |
| NODE_222.g11363.t1 | PHI:7706 | VEDA_05196 | *Verticillium_dahliae* | reduced_virulence | hypothetical protein BFJ68_g14319 | Major facilitator superfamily |
| NODE_315.g12847.t1 | PHI:7708 | VEDA_05198 | *Verticillium_dahliae* | reduced_virulence | hypothetical protein FOMG_10877 | Polyketide synthase, enoylreductase domain |
| DN11506_c0_g1_i1.g203.t1 | PHI:7708 | VEDA_05198 | *Verticillium_dahliae* | reduced_virulence | hypothetical protein FOC1_g10009461 | Polyketide synthase, enoylreductase domain |
| DN1429_c0_g1_i3.g17486.t1 | PHI:548__PHI:2305 | BcPIC5__BcFKBP12 | *Botrytis_cinerea* | reduced_virulence | probable FPR3-prolyl cis-trans isomerase | FKBP-type peptidyl-prolyl cis-trans isomerase domain |
| DN1718_c0_g1_i3.g11929.t1 | PHI:7708 | VEDA_05198 | *Verticillium_dahliae* | reduced_virulence | hypothetical protein FOMG_10877 | Polyketide synthase, enoylreductase domain |
| DN2885_c0_g1_i1.g15478.t1 | PHI:7706 | VEDA_05196 | *Verticillium_dahliae* | reduced_virulence | Putative MFS-type transporter C16A3.17c | Major facilitator superfamily |
