# Supplementary material for: Comparative transcriptomic analysis of races 1, 2, 5 and 6 of Fusarium oxysporum f.sp. pisi in a susceptible pea host identifies differential pathogenicity profiles
Source: BMC Genomics. 2021 Oct 9;22:734. doi: 10.1186/s12864-021-08033-y (PMC8502283; doi:10.1186/s12864-021-08033-y)
Supplement: Supplementary file 10 — Additional file 10: Table S8. Differentially expressed genes in R6 that mapped to the virulence-associated genes on the PHI-base database. [file 12864_2021_8033_MOESM10_ESM.docx]

**Supplementary Table 8**

| **Unigenes** | **PHI No.** | **Sequence homology to** | **Phytopathogens** | **Gene knockout result** | **Predicted protein** | **Protein domain** |
| --- | --- | --- | --- | --- | --- | --- |
| NODE_101.g12446.t1 | PHI:5482 | FTF2 | *Fusarium_oxysporum* | reduced_virulence | hypothetical protein FOTG_10844 | Zn (II)2Cys6 (or C6 zinc) binuclear cluster DNA-binding domainGAL4-like |
| NODE_102.g12456.t1 | PHI:9517 | FgEch1_(FGSG_13111) | *Fusarium_graminearum* | reduced_virulence | enoyl-CoA hydratase | Enoyl-CoA hydratase/isomerase |
| NODE_103.g12541.t1 | PHI:9282 | mfsG_(BCIN_06g00026) | *Botrytis_cinerea* | reduced_virulence | hypothetical protein FOXG_05893 | Major facilitator superfamily |
| NODE_105.g12630.t1 | PHI:4138 | xrp14 | *Xanthomonas_oryzae* | reduced_virulence | cytochrome P450 55A1 | Cytochrome P450 |
| NODE_154.g3940.t1 | PHI:4509 | Ss-odc2 | *Sclerotinia_sclerotiorum* | reduced_virulence | Oxalate decarboxylase oxdC | Cupin 1 |
| NODE_185.g15460.t1 | PHI:8753__PHI:8806 | MoChia1_(MGG_08054)__MoChi1_(MGG_08054) | *Magnaporthe_oryzae* | reduced_virulence | hypothetical protein BFJ69_g14497 | Glycoside hydrolase family 18, catalytic domain |
| NODE_190.g4595.t1 | PHI:5189 | MoLDS1 | *Magnaporthe_oryzae* | reduced_virulence | prostaglandin-endoperoxide synthase 1 | Haem peroxidase superfamily |
| NODE_191.g15592.t1 | PHI:7281 | PsINV | *Puccinia_striiformis* | reduced_virulence | Beta-fructofuranosidase, insoluble isoenzyme 3 | Glycoside hydrolase, family 32 |
| NODE_239.g16199.t1 | PHI:7283 | Pg1 | *Fusarium_graminearum* | reduced_virulence | Polygalacturonase | Glycoside hydrolase, family 28 |
| NODE_24.g963.t1 | PHI:2240__PHI:4586 | Srt1 | *Ustilago_maydis* | reduced_virulence | hypothetical protein FOCG_16099 | Major facilitator, sugar transporter-like |
| NODE_259.g5657.t1 | PHI:2174 | NMR2 | *Magnaporthe_oryzae* | reduced_virulence | hypothetical protein FOC1_g10006917 | NmrA-like domain |
| NODE_267.g5777.t1 | PHI:2207 | endo-1_4-beta-xylanase_[GH10_family] | *Magnaporthe_oryzae* | reduced_virulence | Endoglucanase 3 | Glycoside hydrolase, family 5 |
| NODE_285.g6031.t1 | PHI:305 | ICL1 | *Magnaporthe_oryzae* | reduced_virulence | Isocitrate lyase | Isocitrate lyase |
| NODE_308.g6325.t1 | PHI:3703 | fvtox1 | *Fusarium_virguliforme* | reduced_virulence | hypothetical protein FOTG_03521 |  |
| NODE_378.g7204.t1 | PHI:2240__PHI:4586 | Srt1 | *Ustilago_maydis* | reduced_virulence | hypothetical protein BFJ69_g14850 | Major facilitator, sugar transporter-like |
| NODE_387.g7304.t1 | PHI:9104 | FGRRES_16221 | *Fusarium_graminearum* | reduced_virulence | hypothetical protein FOCG_11444 | Cation/H+ exchanger |
| NODE_42.g1469.t1 | PHI:9241 | Fghyd2_(FGSG_01764) | *Fusarium_graminearum* | reduced_virulence | hypothetical protein FOXG_10949 |  |
| NODE_42.g1470.t1 | PHI:9241 | Fghyd2_(FGSG_01764) | *Fusarium_graminearum* | reduced_virulence | hypothetical protein FOC4_g10007522 |  |
| NODE_432.g7810.t1 | PHI:2240__PHI:4586 | Srt1 | *Ustilago_maydis* | reduced_virulence | MFS transporter, SP family, general alpha glucoside:H+ symporter | Major facilitator, sugar transporter-like |
| NODE_452.g8030.t1 | PHI:8646 | FgLDHL2_(FGSG_16220) | *Fusarium_graminearum* | reduced_virulence | probable CYB2-lactate dehydrogenase cytochrome b2 | Alpha-hydroxy acid dehydrogenase, FMN-dependent |
| NODE_5.g241.t1 | PHI:7173 | HiC-15 | *Verticillium_dahliae* | reduced_virulence | Pisatin demethylase | Cytochrome P450 |
| NODE_538.g8878.t1 | PHI:2240__PHI:4586 | Srt1 | *Ustilago_maydis* | reduced_virulence | Sugar transporter STL1 | Sugar transporter, conserved site |
| NODE_57.g1857.t1 | PHI:4231 | MoFLP1 | *Magnaporthe_oryzae* | reduced_virulence | hypothetical protein BFJ69_g7592 | Fasciclin domain |
| NODE_677.g10118.t1 | PHI:2240__PHI:4586 | Srt1 | *Ustilago_maydis* | reduced_virulence | hypothetical protein FOTG_16328 | Major facilitator, sugar transporter-like |
| NODE_730.g10532.t1 | PHI:7787 | AldA_(PSPTO_0092) | *Pseudomonas_syringae* | reduced_virulence | Aldehyde dehydrogenase | Aldehyde dehydrogenase, C-terminal |
| DN17490_c0_g1_i1.g18966.t1 | PHI:2099 | Pmc1 | *Magnaporthe_oryzae* | reduced_virulence | Ca2+-transporting ATPase | P-type ATPase, cytoplasmic domain N |
| DN1784_c0_g1_i1.g19985.t1 | PHI:3315 | conx1 | *Magnaporthe_oryzae* | reduced_virulence | hypothetical protein FOTG_07898 | Zn (2)-C6 fungal-type DNA-binding domain-GAL4 |
| DN3822_c0_g1_i1.g17164.t1 | PHI:9241 | Fghyd2_(FGSG_01764) | *Fusarium_graminearum* | reduced_virulence | hypothetical protein FOTG_14648 |  |
| DN7520_c0_g1_i1.g3441.t1 | PHI:4231 | MoFLP1 | *Magnaporthe_oryzae* | reduced_virulence | hypothetical protein FOQG_15399 | Fasciclin domain |
