# Supplementary material for: Comparative transcriptomic analysis of races 1, 2, 5 and 6 of Fusarium oxysporum f.sp. pisi in a susceptible pea host identifies differential pathogenicity profiles
Source: BMC Genomics. 2021 Oct 9;22:734. doi: 10.1186/s12864-021-08033-y (PMC8502283; doi:10.1186/s12864-021-08033-y)
Supplement: Supplementary file 11 — Additional file 11: Table S9. CAZyme prediction of the differentially expressed genes in R1. [file 12864_2021_8033_MOESM11_ESM.docx]

**Supplementary Table 9**

| **Unigene** | **Predicted protein/Protein domain** | **HMMER** | **Hotpep** | **DIAMOND** | **Signalp** | **# of Tools** |
| --- | --- | --- | --- | --- | --- | --- |
| NODE_107.g3183.t1 | Putative endo-beta-1,4-glucanase D | AA9(7-210) | AA9+CBM1 | AA9+CBM1 | Y | 3 |
| NODE_108.g12808.t1 | Pectate lyase | PL1 | PL1 | CBM1+PL1_4 | Y | 3 |
| NODE_115.g13120.t1 | alpha-N-arabinofuranosidase B | GH54(22-336) +CBM42(354-494) | GH54+CBM13+CBM42 | CBM42+GH54 | N | 3 |
| NODE_118.g13251.t1 | Pectin lyase fold | PL9_3(20-377) | PL9 | PL9_3 | Y | 3 |
| NODE_121.g3504.t1 | Laccase | AA1_3(97-416) | AA1 | AA1_3 | N | 3 |
| NODE_135.g13955.t1 | pectate lyase | PL3_2(26-212) | PL3 | PL3_2 | Y | 3 |
| NODE_136.g14009.t1 | Xyloglucanase | GH74(82-179) | GH74+CBM1+CBM2 | GH74 | Y | 3 |
| NODE_14.g638.t1 | Putative pectate lyase F | PL3_2(30-217) | PL3 | PL3_2 | Y | 3 |
| NODE_14.g646.t1 | Glyoxal oxidase | AA5(390-895) | AA5 | AA5_1 | Y | 3 |
| NODE_15.g691.t1 | murein transglycosylase | AA9(9-229) | AA9+CBM1 | AA9 | Y | 3 |
| NODE_15.g706.t1 | hypothetical protein FOXG_05669 | CE12(22-235) | CE12 | CE12 | Y | 3 |
| NODE_162.g4293.t1 | Galactose oxidase | AA5_2(60-677) | AA5+CBM13+CBM32 | AA5_2+CBM32 | Y | 3 |
| NODE_164.g14881.t1 | Beta-glucosidase | GH132(137-438) | GH132 | GH132 | N | 3 |
| NODE_193.g4836.t1 | Pectinesterase | CE8(27-306) | CE8 | CE8 | Y | 3 |
| NODE_200.g4940.t1 | Cellulose-binding | AA16(17-183) | AA16 | AA16 | Y | 3 |
| NODE_27.g1127.t1 | Beta-glucosidase 1A | GH1(30-497) | GH1 | GH1 | N | 3 |
| NODE_294.g6342.t1 | cutinase | CE5(52-228) | CE5 | CE5 | Y | 3 |
| NODE_3.g133.t1 | alpha-L-arabinofuranosidase II precursor | GH43_26(21-306) | GH43 | GH43_26 | N | 3 |
| NODE_344.g7009.t1 | hypothetical protein BFJ68_g15668 | CBM38(34-162) +GH32(220-516) | GH32 | CBM38+GH32 | Y | 3 |
| NODE_348.g7058.t1 | endoglucanase type F | CBM1(22-49) +GH10(84-383) | GH10+CBM1+CBM2 | CBM1+GH10 | Y | 3 |
| NODE_370.g7336.t1 | Pectin lyase fold | PL1_2(53-228) | PL1 | PL1_2 | Y | 3 |
| NODE_556.g9273.t1 | β-1,4-endoxylanase | GH10(29-326) | GH10+CBM1+CBM2+CBM22 | GH10 | Y | 3 |
| NODE_592.g9595.t1 | Multicopper oxidase | AA1_3(64-379) | AA1 | AA1_3 | N | 3 |
| NODE_592.g9596.t1 | hypothetical protein BFJ71_g12101 | GH31(361-882) | GH31 | GH31 | N | 3 |
| NODE_614.g9784.t1 | Unsaturated rhamnogalacturonyl hydrolase yteR | GH105(32-378) | GH105 | GH105 | N | 3 |
| NODE_617.g9818.t1 | putative exopolygalacturonase X | GH28 | GH28 | GH28 | Y | 3 |
| NODE_644.g10030.t1 | cellulose-binding-like domain (Expansin) | CBM63(232-302) | CBM63 | CBM63 | Y | 3 |
| NODE_661.g10166.t1 | glycogen synthase | GT3(18-655) | GT3 | GT3 | N | 3 |
| NODE_688.g10378.t1 | choline dehydrogenase | AA3_2(43-668) | AA3 | AA3 | Y | 3 |
| NODE_708.g10526.t1 | related to cellobiose dehydrogenase | AA3_2(13-532) | AA3 | AA3_2 | N | 3 |
| NODE_726.g10654.t1 | Glucose-methanol-choline oxidoreductase | AA3_2(23-636) | AA3 | AA3 | N | 3 |
| NODE_815.g11287.t1 | Bifunctional xylanase/deacetylase | CE4(32-155) | CE4+CBM18 | CE4 | Y | 3 |
| NODE_86.g2693.t1 | Pectinesterase | CE8(408-673) +CE8(823-1096) | CE8 | CE8 | Y | 3 |
| DN10699_c0_g1_i1.g19153.t1 | hypothetical protein FOC4_g10012713 | AA11(18-206) | AA11 | AA11 | Y | 3 |
| DN10792_c0_g1_i1.g14344.t1 | chitinase | GH18(21-423) | CBM18 | CBM18+GH18 | N | 3 |
| DN1155_c0_g1_i1.g23823.t1 | Cellulose/chitin-binding protein | AA16(17-183) | AA16 | AA16 | Y | 3 |
| DN12928_c0_g1_i1.g17060.t1 | pectate lyase E | PL3_2(43-204) | PL3+CBM13 | PL3_2 | Y | 3 |
| DN13425_c0_g1_i1.g15307.t1 | α-1,4-galacturonidase | GH28(56-407) | GH28 | GH28 | N | 3 |
| DN14440_c0_g1_i1.g6409.t1 | probable pectate lyase 1 | PL1_7(80-260) | PL1 | PL1_7 | Y | 3 |
| DN2059_c0_g1_i2.g9743.t1 | hypothetical protein BFJ65_g9754 | GH131(35-290) | GH131 | GH131 | Y | 3 |
| DN21523_c0_g1_i1.g109.t1 | hypothetical protein BFJ67_g12805 | GH18(500-854) | GH18+CBM18 | CBM18+CBM50+GH18 | N | 3 |
| DN3782_c0_g1_i2.g11889.t1 | hypothetical protein BFJ69_g1977 | CBM63(131-201) | CBM63 | CBM63 | N | 3 |
| DN5768_c0_g1_i1.g19843.t1 | hypothetical protein BFJ69_g6260 | AA9(3-151) | AA9+CBM1 | AA9 | N | 3 |
| DN6661_c0_g1_i1.g4823.t1 | endopolygalacturonase PG2 | GH28 | GH28 | GH28 | N | 3 |
| DN930_c1_g1_i1.g3810.t1 | Putative N-acetylglucosamine-6-phosphate deacetylase | CE9(17-410) | CE9 | CE9 | N | 3 |
| DN9396_c0_g1_i2.g1442.t1 | Putative rhamnogalacturonate lyase A | PL4 | PL4 | PL4_1 | N | 3 |
