# Supplementary material for: Comparative transcriptomic analysis of races 1, 2, 5 and 6 of Fusarium oxysporum f.sp. pisi in a susceptible pea host identifies differential pathogenicity profiles
Source: BMC Genomics. 2021 Oct 9;22:734. doi: 10.1186/s12864-021-08033-y (PMC8502283; doi:10.1186/s12864-021-08033-y)
Supplement: Supplementary file 12 — Additional file 12: Table S10. CAZyme prediction of the differentially expressed genes in R2. [file 12864_2021_8033_MOESM12_ESM.docx]

**Supplementary Table 10**

| **Unigene** | **Predicted protein/Protein domain** | **HMMER** | **Hotpep** | **DIAMOND** | **Signalp** | **# of Tools** |
| --- | --- | --- | --- | --- | --- | --- |
| NODE_102.g14860.t1 | Pectin lyase fold | PL9_3(20-377) | PL9 | PL9_3 | N | 3 |
| NODE_122.g4568.t1 | beta-galactosidase | GH35(45-390) | GH35 | GH35 | Y | 3 |
| NODE_128.g16246.t1 | Putative pectate lyase F | PL3_2(26-212) | PL3 | PL3_2 | Y | 3 |
| NODE_129.g16326.t1 | putative unsaturated glucuronyl hydrolase | GH105(48-369) | GH105 | GH105 | N | 3 |
| NODE_129.g4731.t1 | Cellobiose dehydrogenase | AA8(11-758) | AA3+AA8 | AA3_1+AA8 | Y | 3 |
| NODE_129.g4732.t1 | SGNH hydrolase-type esterase domain | CE12(22-235) | CE12 | CE12 | Y | 3 |
| NODE_129.g4747.t1 | murein transglycosylase | AA9(9-229) | AA9+CBM1 | AA9 | Y | 3 |
| NODE_136.g4881.t1 | Glycoside hydrolase | AA9(8-228) | AA9 | AA9 | Y | 3 |
| NODE_138.g16693.t1 | Glucose-methanol-choline oxidoreductase | AA3_2(18-599) | AA3 | AA3_2 | N | 3 |
| NODE_142.g16893.t1 | Cellulose-binding domain | CBM63(131-201) | CBM63 | CBM63 | N | 3 |
| NODE_149.g17145.t1 | glucan 1,6-alpha-glucosidase | GH13_40(33-392) | GH13 | GH13_40 | N | 3 |
| NODE_162.g5386.t1 | Putative endoglucanase type F | GH10(84-383) | GH10+CBM1+CBM2 | CBM1+GH10 | Y | 3 |
| NODE_172.g17916.t1 | Glycosyl hydrolase | AA9(9-225) | AA9 | AA9 | Y | 3 |
| NODE_179.g18113.t1 | endo-1,4-beta-xylanase A | GH11(54-228) | GH11+CBM1+CBM36 | GH11 | Y | 3 |
| NODE_180.g18139.t1 | alcohol oxidase | AA3_3(1-532) | AA3 | AA3_3 | N | 3 |
| NODE_189.g18371.t1 | Lytic polysaccharide monooxygenase | AA16(17-183) | AA16 | AA16 | Y | 3 |
| NODE_189.g5988.t1 | alpha-mannosidase | GH125(70-497) | GH125 | GH125 | N | 3 |
| NODE_250.g19542.t1 | Glycosyl hydrolase | GH105(32-378) | GH105 | GH105 | N | 3 |
| NODE_253.g19575.t1 | Glycoside hydrolase | AA9(9-227) | AA9+CBM1 | AA9 | N | 3 |
| NODE_274.g7472.t1 | Pectinesterase | CE8(27-306) | CE8 | CE8 | Y | 3 |
| NODE_281.g7597.t1 | Pectin lyase | PL1_4(114-297) | PL1 | PL1_4 | Y | 3 |
| NODE_306.g8027.t1 | Multicopper oxidase | AA1_2(43-379) | AA1 | AA1_2 | Y | 3 |
| NODE_321.g8245.t1 | Putative exoglucanase type C | GH7(19-451) | GH7+CBM1 | CBM1+GH7 | Y | 3 |
| NODE_330.g8363.t1 | Glycosyl hydrolase | AA9(7-222) | AA9 | AA9 | Y | 3 |
| NODE_349.g8644.t1 | Glycosyl hydrolase | GH81(173-847) | GH81 | GH81 | Y | 3 |
| NODE_377.g9040.t1 | Glycogen synthase | GT3(18-655) | GT3 | GT3 | N | 3 |
| NODE_379.g9073.t1 | Bifunctional xylanase/deacetylase | CE4(32-155) | CE4+CBM18 | CE4 | Y | 3 |
| NODE_415.g9525.t1 | endopolygalacturonase 1 | GH28(40-353) | GH28 | GH28 | Y | 3 |
| NODE_439.g9794.t1 | pectate lyase | PL3_2(44-233) | PL3+CBM13 | PL3_2 | Y | 3 |
| NODE_478.g10252.t1 | Glycoside hydrolase | AA9(6-230) | AA9+CBM1 | AA9 | Y | 3 |
| NODE_516.g10687.t1 | Alpha-xylosidase | GH31(258-698) | GH31 | GH31 | N | 3 |
| NODE_544.g10979.t1 | Glycosyl hydrolase | GH13_40(34-391) | GH13 | GH13_40 | N | 3 |
| NODE_559.g11137.t1 | Endo-1,4-beta-xylanase | GH10(34-351) | GH10+CBM1+CBM2 | GH10 | Y | 3 |
| NODE_614.g11692.t1 | Glycoside hydrolase | AA9(6-230) | AA9+CBM1 | AA9 | Y | 3 |
| NODE_614.g11696.t1 | endoglucanase type B | GH6(130-422) | GH6+CBM1 | CBM1+GH6 | Y | 3 |
| NODE_620.g11759.t1 | Glycoside hydrolase | GH3(37-248) | GH3 | GH3 | N | 3 |
| NODE_703.g12511.t1 | Choline dehydrogenase | AA3_2(2-457) | AA3 | AA3_2 | N | 3 |
| NODE_793.g13245.t1 | Cellulose-binding domain | CE1(42-235) | CE1+CBM1 | CBM1+CE1 | Y | 3 |
| NODE_794.g13252.t1 | chitin synthase | GT2_Chitin_synth_1(213-381) | GT2 | GT2 | N | 3 |
| NODE_801.g13294.t1 | pectate lyase E | PL3_2(15-200) | PL3+CBM13 | PL3_2 | N | 3 |
| NODE_815.g13411.t1 | xyloglucanase | GH74(12-103) | GH74+CBM1+CBM2 | GH74 | N | 3 |
| NODE_841.g13643.t1 | galacturan 1,4-alpha-galacturonidase | GH28(74-425) | GH28 | GH28 | N | 3 |
| NODE_868.g13817.t1 | Glycoside hydrolase | GH43_36(30-300) | GH43 | GH43_36 | Y | 3 |
| NODE_912.g14121.t1 | alpha-N-arabinofuranosidase C | GH51(14-505) | GH51 | GH51 | N | 3 |
| NODE_970.g14522.t1 | Putative N-acetylglucosamine-6-phosphate deacetylase | CE9(17-410) | CE9 | CE9 | N | 3 |
| NODE_995.g14687.t1 | Six-hairpin glycosidase-like | GH88(88-480) | GH88 | GH88 | N | 3 |
| DN1048_c0_g1_i1.g12018.t1 | beta-glucosidase | GH1(1-359) | GH1 | GH1 | N | 3 |
| DN10804_c0_g1_i1.g28875.t1 | Galactose oxidase | AA5_2(1-236) | AA5+CBM32 | AA5_2+CBM32 | N | 3 |
| DN38_c1_g1_i2.g8917.t1 | hypothetical protein BFJ69_g14063 | AA11(19-212) | AA11 | AA11 | N | 3 |
| DN6468_c0_g1_i1.g13420.t1 | Carbohydrate esterase 2 | CE2(100-311) | CE2 | CE2 | N | 3 |
