# Supplementary material for: Comparative transcriptomic analysis of races 1, 2, 5 and 6 of Fusarium oxysporum f.sp. pisi in a susceptible pea host identifies differential pathogenicity profiles
Source: BMC Genomics. 2021 Oct 9;22:734. doi: 10.1186/s12864-021-08033-y (PMC8502283; doi:10.1186/s12864-021-08033-y)
Supplement: Supplementary file 13 — Additional file 13: Table S11. CAZyme prediction of the differentially expressed genes in R5. [file 12864_2021_8033_MOESM13_ESM.docx]

**Supplementary Table 11**

| **Unigene** | **Predicted protein/Protein domain** | **HMMER** | **Hotpep** | **DIAMOND** | **Signalp** | **#of Tools** |
| --- | --- | --- | --- | --- | --- | --- |
| NODE_104.g15115.t1 | Glycoside hydrolase | GH23(205-330) | GH23 | CBM50+GH23 | Y | 3 |
| NODE_11.g1534.t1 | pectate lyase E | PL3 | PL3 | PL3 | N | 3 |
| NODE_122.g8541.t1 | Pectinesterase | CE8(407-672) +CE8(822-1095) | CE8 | CE8 | Y | 3 |
| NODE_126.g8714.t1 | 1,4-alpha-glucan-branching enzyme | GH13_8(250-543) | GH13+CBM48 | CBM48+GH13_8 | N | 3 |
| NODE_161.g9832.t1 | alpha-L-arabinofuranosidase | GH43_26(21-306) | GH43 | GH43_26 | N | 3 |
| NODE_163.g9887.t1 | Laccase | AA1_3(97-416) | AA1 | AA1_3 | N | 3 |
| NODE_218.g11285.t1 | alpha-N-arabinofuranosidase | GH54(22-336) +CBM42(354-494) | GH54+CBM13+CBM42 | CBM42+GH54 | N | 3 |
| NODE_280.g12348.t1 | Glucose-methanol-choline oxidoreductase | AA3_1(18-545) | AA3+AA8 | AA3_1 | Y | 3 |
| NODE_306.g12739.t1 | endo-xylogalacturonan hydrolase | GH28 | GH28 | GH28 | N | 3 |
| NODE_31.g3462.t1 | pectate lyase E | PL3 | PL3 | PL3 | N | 3 |
| NODE_39.g4038.t1 | hypothetical protein FOC4_g10009379 | GT15(118-425) | GT15 | GT15 | N | 3 |
| NODE_422.g13801.t1 | beta-galactosidase | GH35(45-390) | GH35 | GH35 | N | 3 |
| NODE_55.g5156.t1 | hypothetical protein FOTG_13023 | GT1(115-519) | GT1 | GT1 | N | 3 |
| NODE_63.g5661.t1 | Cutinase | CE5(52-228) | CE5 | CE5 | Y | 3 |
| NODE_63.g5663.t1 | Putative N-acetylglucosamine-6-phosphate deacetylase | CE9(17-410) | CE9 | CE9 | N | 3 |
| NODE_649.g14610.t1 | choline dehydrogenase | AA3_2(13-532) | AA3 | AA3_2 | N | 3 |
| NODE_65.g5772.t1 | Cellulose-binding-like domain (Expansin) | CBM63(131-201) | CBM63 | CBM63 | N | 3 |
| NODE_70.g6030.t1 | beta-fructofuranosidase | GH32(45-354) | GH32+CBM38 | GH32 | Y | 3 |
| NODE_82.g6756.t1 | Glycoside hydrolase | AA9(18-208) | AA9 | AA9 | Y | 3 |
| DN1062_c0_g1_i5.g39219.t1 | hypothetical protein BFJ65_g11565 | GT90(320-605) | GT90 | GT90 | N | 3 |
| DN11110_c0_g1_i1.g49980.t1 | hypothetical protein BFJ69_g14497 | GH18(21-423) | CBM18 | CBM18+GH18 | N | 3 |
| DN1195_c0_g1_i1.g17770.t1 | Glycoside hydrolase | AA11(18-208) | AA11 | AA11 | Y | 3 |
| DN13775_c0_g1_i1.g395.t1 | Putative N-acetylglucosamine-6-phosphate deacetylase | CE9(17-410) | CE9 | CE9 | N | 3 |
| DN19557_c0_g1_i1.g49473.t1 | Invertase 2 | GH32(1-242) | GH32 | GH32 | N | 3 |
| DN2721_c0_g1_i2.g15133.t1 | 1,4-alpha-glucan-branching enzyme | GH13_8(250-543) | GH13+CBM48 | CBM48+GH13_8 | N | 3 |
| DN2871_c0_g1_i2.g15416.t1 | hypothetical protein FOPG_03657 | GT1(218-533) | GT1 | GT1 | N | 3 |
| DN3219_c0_g1_i1.g50367.t1 | Glycoside hydrolase | AA9(18-208) | AA9 | AA9 | Y | 3 |
| DN5098_c0_g1_i2.g7627.t1 | Glycogen synthase | GT3(18-655) | GT3 | GT3 | N | 3 |
| DN7970_c0_g1_i2.g13011.t1 | Cellulose-binding-like domain (Expansin) | CBM63(131-201) | CBM63 | CBM63 | N | 3 |
