# Supplementary material for: Comparative transcriptomic analysis of races 1, 2, 5 and 6 of Fusarium oxysporum f.sp. pisi in a susceptible pea host identifies differential pathogenicity profiles
Source: BMC Genomics. 2021 Oct 9;22:734. doi: 10.1186/s12864-021-08033-y (PMC8502283; doi:10.1186/s12864-021-08033-y)
Supplement: Supplementary file 14 — Additional file 14: Table S12. CAZyme prediction of the differentially expressed genes in R6. [file 12864_2021_8033_MOESM14_ESM.docx]

**Supplementary Table 12**

|  | **Predicted protein/Protein domain** | **HMMER** | **Hotpep** | **DIAMOND** | **Signalp** | **# of Tools** |
| --- | --- | --- | --- | --- | --- | --- |
| NODE_139.g3662.t1 | arabinogalactan endo-1,4-beta-galactosidase | GH53(21-345) | GH53 | GH53 | Y | 3 |
| NODE_166.g4160.t1 | Galactose oxidase | AA5_2(60-677) | AA5+CBM13+CBM32 | AA5_2+CBM32 | Y | 3 |
| NODE_174.g15209.t1 | alcohol oxidase | AA3_3(1-534) | AA3 | AA3_3 | N | 3 |
| NODE_185.g15460.t1 | hypothetical protein BFJ69_g14497 | GH18(21-423) | CBM18 | CBM18+GH18 | N | 3 |
| NODE_214.g4970.t1 | cellulose-binding-like domain (Expansin) | CBM63(131-201) | CBM63 | CBM63 | N | 3 |
| NODE_224.g5124.t1 | murein transglycosylase | AA9(9-229) | AA9+CBM1 | AA9 | Y | 3 |
| NODE_239.g16199.t1 | Polygalacturonase | GH28(37-346) | GH28 | GH28 | Y | 3 |
| NODE_267.g5777.t1 | Endoglucanase 3 | GH5_5(84-360) | GH5+CBM1 | CBM1+GH5_5 | Y | 3 |
| NODE_63.g2016.t1 | choline dehydrogenase | AA3_2(23-636) | AA3 | AA3 | Y | 3 |
| NODE_97.g2804.t1 | Pectinesterase | CE8(27-306) | CE8 | CE8 | Y | 3 |
| DN10613_c0_g1_i1.g23.t1 | Pectinesterase | CE8(27-243) | CE8 | CE8 | Y | 3 |
